# Disentangling the impact of cerebrospinal fluid formation and neuronal activity on solute clearance from the brain

**DOI:** 10.1186/s12987-023-00443-2

**Published:** 2023-06-14

**Authors:** Martin Segeroth, Lydia Wachsmuth, Mathias Gagel, Franziska Albers, Andreas Hess, Cornelius Faber

**Affiliations:** 1 Translational Research Imaging Center (TRIC), Clinic of Radiology, University of Münster, Albert-Schweitzer-Campus 1, Gebäude A16, 48149 Münster, Germany; 2 Department of Radiology, University Hospital Basel, Basel, Switzerland; 3 Department of Experimental and Clinical Pharmacology and Toxicology, Friedrich-Alexander-University of Erlangen-Nürnberg, Erlangen, Germany; 4 Institute of Neuroradiology, University Hospital Erlangen, Friedrich-Alexander-University Erlangen-Nürnberg (FAU), Erlangen, Germany; 5 FAU NeW, Research Center for New Bioactive Compounds, Nikolaus-Fiebiger-Str. 10, 91058 Erlangen, Germany

**Keywords:** Glymphatic system, Solute clearance, Kinetic modeling, DCE MRI, DWI, Brain state, CSF production, Ca^2+^ recordings

## Abstract

**Background:**

Despite recent attention, pathways and mechanisms of fluid transposition in the brain are still a matter of intense discussion and driving forces underlying waste clearance in the brain remain elusive. Consensus exists that net solute transport is a prerequisite for efficient clearance. The individual impact of neuronal activity and cerebrospinal fluid (CSF) formation, which both vary with brain state and anesthesia, remain unclear.

**Methods:**

To separate conditions with high and low neuronal activity and high and low CSF formation, different anesthetic regimens in naive rat were established, using Isoflurane (ISO), Medetomidine (MED), acetazolamide or combinations thereof. With dynamic contrast-enhanced MRI, after application of low molecular weight contrast agent (CA) Gadobutrol to cisterna magna, tracer distribution was monitored as surrogate for solute clearance. Simultaneous fiber-based Ca^2+^-recordings informed about the state of neuronal activity under different anesthetic regimen. T2-weighted MRI and diffusion-weighted MRI (DWI) provided size of subarachnoidal space and aqueductal flow as surrogates for CSF formation. Finally, a pathway and mechanism-independent two-compartment model was introduced to provide a measure of efficiency for solute clearance from the brain.

**Results:**

Anatomical imaging, DWI and Ca^2+^-recordings confirmed that conditions with distinct levels of neuronal activity and CSF formation were achieved. A sleep-resembling condition, with reduced neuronal activity and enhanced CSF formation was achieved using ISO+MED and an awake-like condition with high neuronal activity using MED alone. CA distribution in the brain correlated with the rate of CSF formation. The cortical brain state had major influence on tracer diffusion. Under conditions with low neuronal activity, higher diffusivity suggested enlargement of extracellular space, facilitating a deeper permeation of solutes into brain parenchyma. Under conditions with high neuronal activity, diffusion of solutes into parenchyma was hindered and clearance along paravascular pathways facilitated. Exclusively based on the measured time signal curves, the two-compartment model provided net exchange ratios, which were significantly larger for the sleep-resembling condition than for the awake-like condition.

**Conclusions:**

Efficiency of solute clearance in brain changes with alterations in both state of neuronal activity and CSF formation. Our clearance pathway and mechanism agnostic kinetic model informs about net solute transport, solely based on the measured time signal curves. This rather simplifying approach largely accords with preclinical and clinical findings.

**Supplementary Information:**

The online version contains supplementary material available at 10.1186/s12987-023-00443-2.

## Background

Fluid circulation in the brain is of longstanding research interest given its importance for brain homoeostasis, supply of energy and oxygen, waste removal and its peculiarities, namely the existence of a blood brain barrier and the long-adopted conception of absence of a classical lymphatic system. The historic view postulated diffusion as dominant mechanism for flow and molecular transport within brain’s extracellular space [1]. Cserr et al. [2, 3] challenged the early assumption showing that tracers moved much faster and along vessels through the brain and concluded that molecular-size independent transport was driven primarily by advection.

The proposal of a brain-wide pathway of fluid transport, the glymphatic system in 2012 [4], the connection of this pathway with a major role of sleep for efficient glymphatic clearance one year later [5], together with the discovery of a brain lymphatic system [6] massively propelled scientific efforts to elucidate the mechanisms underlying waste clearance in the brain which is of utmost importance in the context of several neurodegenerative diseases like Alzheimer’s disease. The originally proposed uni-directional pathway consisted of para-arterial influx of subarachnoid cerebrospinal fluid (CSF) into brain interstitium and para-venous clearance of interstitial fluid (ISF), with astrocytic endfeet AQP4 channel-gated interstitial bulk flow in between. The debate about mechanisms of solute transport within interstitium is ongoing. Some groups questioned bulk flow as underlying mechanism, arguing that unphysiologically strong forces would be necessary to build up a sufficiently large gradient to transport CSF through the interstitial space. Especially arterial pulsations, as suggested by Iliff et al. [4], would be too weak to create the required forces [7–9]. Some authors suggested diffusion [10–12], others a combination of both diffusion and convection as underlying mechanism [7, 12–14]. It has also been suggested that influx of CSF occurs along the basement membranes of glial and pial cells, and efflux follows intramural para-arterial drainage pathways along basement membranes of the smooth muscle layer [15, 16]. Further, the existence of interconnected para-vascular compartments from arterioles to veins was demonstrated, which may provide a route for fluid flow as well as transport of molecules [17]. Several recent reviews in detail summarize the current state of knowledge and controversies [18–21].

Evidence supporting the different hypothesis comes from different experimental techniques, all exhibiting unique strengths and limitations. Among a number of applied imaging methods, two-photon microscopy [4, 5] for example offers high spatial resolution but limited field of view and does not allow for observing fluid’s dynamics across the entire brain. Dynamic contrast-enhanced (DCE) MRI [22, 23] on the other hand, provides the whole brain view at a lower spatial and temporal resolution. Both methods rely on distribution of tracers of various molecular sizes as surrogate for CSF flow in the brain. However, a recent MRI approach using isotopically enriched H_2_^17^O directly resolved water distribution in the brain [24]. Post-mortem histology provides excellent spatial resolution and sensitivity, but alteration of tissue microstructure at time of death as well as dehydration and fixation may hamper interpretation of results.

A potential role of sleep for fluid dynamics in the brain had been brought into play early [5] and subsequent studies on the glymphatic system were performed in the awake or sleep state, or under various anesthetic regimens selected to resemble these conditions. The state of brain activity is usually monitored by electroencephalogram (EEG) or (in animals) local field potential (LFP) recordings. The unique sleep signature with slow alternation of neuronal membrane potentials between hyperpolarized down-states with neuronal quiescence and depolarized up-states with action potentials, has been characterized in seminal papers by Steriade et al. [25–27]. The so-called slow waves are consistently found during non-rapid eye-movement (NREM) sleep across species and in the brain state under Isoflurane (and several other anesthetics). Such bistability is not seen in sedated or awake animals. Under higher levels of isoflurane down-states become longer and more frequent [28]. Conversely, at low doses of isoflurane, suppression periods are nearly abolished, with the EEG being characterized by nearly persistent up-states [29–31]. Several studies have shown that both brain states can be distinguished by LFP and calcium recordings [30–32]. Reduced cerebral glucose (by 30–50%) and oxygen (by 5–25%) metabolism in animal and men [33, 34] accompanying the decrease in neuronal firing rate and change in firing pattern during NREM sleep have been reported, whereas, during rapid eye movement (REM) sleep, the level of brain metabolism was similar to that of wakefulness. Additional evidence for a reduction in cerebral activity and metabolism during NREM sleep is the decrease of brain lactate levels during slow wave sleep [35].

Sleep/awake state or anesthetic regimen, however, not only modulate neuronal activity but also CSF formation [5, 36–38]. Consequently, multiple factors may exert their effects on fluid dynamics and thereby impact results and interpretation. We hypothesize that CSF formation modulates CSF distribution along paravascular and subarachnoidal spaces, and that the state of neuronal arousal has major impact on the interstitial distribution.

In the current study, we selected three anesthetic regimens resembling different states of brain activity. Medetomidine (MED), an α_2_-adrenergic receptor agonist was used to achieve a state of sedation with relatively high neuronal activity [1, 39–41]. Isoflurane (ISO), acting as a modulator of GABA_A_ chloride channels was used to achieve reduced neuronal activity [29, 30]. The effect of the two compounds on CSF formation is opposite, with high CSF formation under MED and lower CSF formation under ISO [29, 30]. The combination of both anesthetics (MED-ISO) was used to resemble a state of low neuronal activity with higher CSF formation. To further suppress CSF formation, we additionally administered Acetazolamide (AZE), a carboanhydrase inhibitor [42], to all three regimens. We measured brain state, tissue microstructure and contrast agent distribution using optical Ca^2+^-recordings, DWI MRI and DCE-MRI. Finally, we fed DCE data into a simplistic two-compartment kinetic model in order to quantitatively assess the efficiency of net solute transport.

## Materials and methods

### Animals

Experiments were performed with 95 female Fisher rats (F344) aged 3 to 5 months (122–203 g, 171 ± 17.5 g body weight). Animals were obtained from Charles River Laboratories (Erkrat, Germany) or bred in the central animal facility of the university of Muenster. Animals received from Charles River were housed at least 1 week before experiments were performed, animals from the central animal facility were housed at least for 1 day before experiments. Rats were kept in groups of 2–3 animals under 12-h light/12-h dark cycle with ad libitum access to food and water. All surgeries were performed during the light phase to rule out possible effects of the circadian cycle. Experiments were conducted according to the German Tierschutzgesetz and were approved by local authorities (Landesamt für Natur, Umwelt- und Verbraucherschutz Nordrhein-Westfalen, Germany, 84-02.04.2016.A135).

### Study design

Two sets of experiments with different animal preparations were performed. One subset of animals (n = 49) received a contrast agent (CA) administration via cisterna magna and underwent repeated 3D T1 weighted (T1w) whole brain MRI over 6 h (Table 1) under six different anesthetic regimens. Data from this group was used to study contrast agent distribution in the brain and to model solute clearance. In some of these animals simultaneous optical Ca^2+^-recordings were performed. The other group (n = 37) was ventilated using a tracheal tubus and a series of MRI examinations were first performed under isoflurane, and then repeatedly after switching to one of the six anesthetic regimens. In this group, size of arteries [Time Of Flight angiography (TOF)], subarachnoidal space volume (T2w MRI), aqueductal flow (DWI), and apparent diffusion coefficient [ADC, diffusion tensor imaging (DTI)] in neocortex were measured (Table 1).

**Table 1:** Animal numbers used for experiments under different anesthetic protocols (column 1) with the set of techniques applied (column 2 and 3: DCE-MRI only or with simultaneous Ca^2+^-recordings; column 4: bench Ca^2+^-recordings, column 5: T2w MRI/DWI/DTI/TOF performed in ventilated animals

Anesthetic protocol	3D T1w		Bench Ca^2+^-recordings	T2w MRI/DWI/DTI/TOF (ventilated animals)
	Only	With simultaneous Ca^2+^-recordings		
ISO	5	3		6
MED	4	3		6
ISO+MED	3	5		6
MED+AZE	8		3	6
ISO+AZE	10		3	6
ISO+MED+AZE	8		3	7

Animal preparation was generally performed under isoflurane anesthesia (ISO, see dosing details below) (Forene, Abbott, Wiesbaden, Germany). For MRI, animals were placed in a warmed animal cradle and fixed with bite bar and ear plugs. Animals were supplied with a gas mixture of 75% air and 25% oxygen during MRI. Small animal MRI was performed at 9.4 T in a 94/20 Biospec interfaced to a Bruker Avance console controlled by Paravision 5.1 software (Bruker BioSpin, Ettlingen, Germany). For all experiments, a surface receive three-element array coil (Rapid Biomedical, Rimpar, Germany) was positioned above the head of the animal and inserted in a quadrature volume transmit coil (Bruker). Respiratory rate (Additional file 1) and rectal temperature (mean ± standard deviation: 37 ± 0.5 °C) were continuously monitored. Temperature was kept in the physiological range by adjusting the cradle temperature. In a subset of animals, we also monitored the heart rate by ECG recordings (Additional file 2).

Six different anesthetic regimens were applied. For the ISO condition, animals were anesthetized with 1.5–1.7% ISO (1.5% contrast agent injection experiments and 1.7% for intubated animals). The MED condition consisted of continuous subcutaneous (s.c.) Medetomidine (Orion Corporation, Espoo, Finnland) application (40 µg/kg bolus, followed by infusion of 50 µg/kg/h, s.c.). The ISO+MED condition supplemented continuous s.c. MED application with additional ISO (0.8%). All three conditions were performed both with and without intraperitoneal injection of Acetazolamide (AZE, Diamox 500 mg, Vifor SA, Villars-sur-Glane, France, bolus 55.5 mg/kg of Acetazolamide diluted in 0.9% saline).

### Catheter implantation and DCE

For catheter implantation animals were anesthetized with ISO (initiation 5%, continuous dose 2.5% ISO in 100% oxygen) and received buprenorphine (bolus s.c. 0.05 mg/kg, Temgesic, Reckitt Benckiser Healthcare UK Ltd., Hull, UK) 30 min before surgery. Animals were positioned in a stereotactic frame (Stoelting, Dublin, Ireland) on a feedback-controlled heating pad to keep a temperature of 37 °C. Absence of pain was verified by pedal and corneal reflex testing.

The atlanto-occipital membrane was exposed via midline neck incision and blunt removal of neck musculature. Puncture of the atlanto-occipital membrane was performed with a modified standard venous catheter (Terumo, Surflow-W, 26G, Eschborn, Germany) which limited catheter penetration to a depth of 1 mm. The catheter was fixed via tissue glue (Histoacryl, B Braun, Melsungen, Germany) and remaining dead volume in the catheter was cautiously filled with NaCl 0.9%. A PE20 line was attached to the catheter and to a 5 ml syringe in a syringe pump (World Precision Instruments, Sarasota, USA, AL-300). Both contained the MR Contrast agent (CA) Gadobutrol (Gadovist, Bayer, Schering, Leverkusen, Germany, 83 mM) diluted in 0.9% NaCl. Skin incision was sewed to minimize movement of catheter during animal positioning.

After surgery the animal was fixed in prone position in the MRI cradle via custom-made ear plugs and bite bar, and anesthesia was switched to ISO (1.5%). A baseline 3D T1w Fast Low Angle Shot (FLASH) sequence was performed (TE = 3.83 ms, TR = 15 s, scan time 3 min 4 s, flip angle = 15°, FOV = 30 × 30 × 32 mm^3^, spatial resolution = 0.117 × 0.234 × 0.25 mm^3^, matrix = 256 × 128 × 128). Then, anesthesia was switched to one of the six anesthetic regimens and maintained at least for 40 min to ensure habituation to the respective anesthetic condition. Either 80 µl 21 mM Gd-BT-DO3A, or 20 µl 83 mM Gd-BT-DO3A were delivered intrathecally via syringe pump at an infusion rate of 1.6 µl or 0.4 µl/min, respectively (total infusion time of 50 min). Given the small amount of CA injected, no substantial impact on osmolarity of CSF is expected. 3D T1w FLASH scans of the whole brain were continuously acquired during infusion and during the following 5 h, resulting in a total of 40 scans.

#### Fiber-based calcium recordings

Twenty animals (Table 1) additionally received an optic fiber implantation to record calcium release as a surrogate parameter for the brain state, which was measured during the MRI experiments in a subgroup (n = 11). For conditions containing AZE, Ca^2+^-recordings were performed on the bench (n = 9). These animals had received an intracranial injection of the viral construct encoding for the calcium indicator GCaMP6f (pAAV.Syn.GCaMP6f.WPRE.SV40 was a gift from Douglas Kim & GENIE Project (Addgene plasmid # 100837) at least 4 weeks earlier, as described previously [43]. Briefly, rats were anesthetized with Isoflurane (2.5%) and received the analgesic buprenorphine (bolus s.c. 0.05 mg/kg). After fixation in a stereotactic frame via ear and bite bars the skull was exposed and a small craniotomy was accomplished via a dental drill (Ultimate XL-F, NSK, Trier Germany, and VS1/4HP/005, Meisinger, Neuss, Germany). 1 µl AAV1.Syn.GCaMP6f.WPRE.SV40 was injected via a glass capillary into S1FL (anterio-posterior (AP) 0.0 mm, medio-lateral (ML) + 3.0 mm, dorso-ventral (DV) − 1.2 mm) at an 35° angle from medial. At the day of DCE a craniotomy (AP + 0.2 mm, ML + 3.3 mm) was performed as described before and a 200 μm optic fiber (Thorlabs, Newton, NJ, USA) was implanted perpendicular to the dura at DV − 300 μm, above the GCaMP6f expressing region [43]. After verifying detection of calcium signal upon spontaneous brain activity in the stereotactic frame, the fiber was fixed to the skull with UV glue (Polytec, PT GmbH, Waldbrunn, Germany). The amount of the UV glue was reduced to a minimum to avoid MR image distortions.

### Anatomical, TOF, and diffusion MRI

A subset of 37 rats was artificially ventilated for MRI examinations (MRI-1 ventilator, CWE Inc., Ardmore, PA, USA). Following anesthetic induction (see above), animals shortly (minutes) received 3.5% ISO in 100% oxygen to ensure full relaxation of the animal during intubation. Subsequently, the animal was placed in the MRI cradle with the head fixed as described above and the tracheal tubus (Introcan Safety-W, 14G, B Braun Melsungen, Germany) was connected to a ventilator (MRI-1 Ventilator, CWE Inc., Ardmore, PA, USA, 1.8–2.2 ml tidal volume, 53 breaths/min) delivering 1.7% ISO in a mixture of 75% air and 25% oxygen. CO_2_ concentration in the expired air was measured continuously (CapStar-100 CO_2_ Analyzer, CWE Inc., Ardmore, PA, USA) and kept stable by adjusting tidal volume. The fact that the animals did not breath against the machine confirmed that the ventilator settings were appropriate. After positioning the animal in the scanner and performing localizer scans, shimming was performed using MAPSHIM (Bruker). Four different scan protocols were run under ISO anesthesia:

1. TOF to assess vascular volume (3D T1wFLASH, TE = 2.58 ms, TR = 1.5 s, scan time = 12 min 17 s, flip angle = 30°, FOV = 26 × 26 × 28 mm^3^, spatial resolution = 102 × 102 × 109 µm^3^, matrix = 256 × 256 × 256),
2. T2w Rapid Acquisition with Relaxation Enhancement (RARE) to measure the size of the subarachnoidal space (TE = 9 ms, RARE factor = 16, TR = 1500 ms, scan time = 6 min 24 s, flip angle = 180°, FOV = 30 × 30 × 8 mm^3^, spatial resolution = 117 × 234 × 250 µm^3^, matrix = 256 × 128 × 32),
3. DWI to estimate aqueductal flow (TE = 30.045 ms, TR = 5000.001 s, scan time = 23 min 20 s, b-value = 1000 s/mm^2^, FOV = 34 × 28 mm^2^, spatial resolution = 133 × 109 µm^2^, matrix = 256 × 256 × 20).
4. Axial DTI, to measure the apparent diffusion coefficient in neocortex (DTI-EPI, TE = 42.8 ms, TR = 4250 ms, scan time = 7 min 56 s, b-values = [10 200 400 600 800 1000 1200 1400 1500] s/mm^2^, segments = 4, FOV = 30 × 30 mm^2^, spatial resolution = 0.156 × 0.156 mm^2^, slice thickness = 1 mm, matrix = 192 × 192 × 17).

Subsequently, anesthesia was switched to one of the six anesthetic conditions. After a waiting period of 40 min, all scan protocols were acquired again.

### Postprocessing

For each experiment, brains were segmented from 3D T1w FLASH, and all images were registered to the initially acquired scan of its measurement series, to correct for animal movement. Next, data were registered on the Waxholm Space rat atlas [44] with 79 brain regions (from the 76 brain regions of the publication the central canal is omitted and the hippocampal formation is divided into cornu ammonis regions 1–3, the fasciola cinereum, and the dentate gyrus) via ANTS [Advanced Normalization ToolS (RRID:SCR_004757)] [45]. Mean signal intensities were retrieved for each brain region at each time point. Time signal curves (TSC), showing relative signal changes from baseline (BL) for each brain region were calculated by region-wise normalization to the mean intensity in the pre-contrast scan. In order to quantitatively characterize contrast agent distribution, the following parameters were calculated region-wise for statistical analysis and voxel-wise for 3D visualization using MATLAB (MATLAB 2021a, The MathWorks, Inc., Natick, Massachusetts, United States). Data are presented both region-wise and averaged over all brain regions: (1) arrival time *t*_*a*_, in accordance with previous work [46], was estimated by finding the time point where signal increased for at least three successive measurements, (2) maximum signal and time to maximum *t*_*max*_, (3) area under the curve (AUC) was calculated as the integral of the TSC from a scan before CA injection (t = 0) to 6 h after injection, as.

1 $$\text{AUC}= \int\nolimits_{0}^{{6}\,{\text{h}}}\text{TSC}(t)\text{dt},$$

(4) Signal decay rate b was calculated by fitting a mono-exponential decay (exp) to the TSC.

2 $$exp=\text{a}\cdot {e}^{-\text{b}\cdot t}$$

where a is an amplitude factor. For region-wise analysis, MR signal was averaged over each of the 79 brain regions and fitting was performed over the time period between 90 min after maximum of the specific TSC, *t*_*max*_, and end of data acquisition. Data were discarded if *t*_*max*_ was reached less than 90 min before the end of data acquisition, or if b was not positive.

**Fig. 1:**
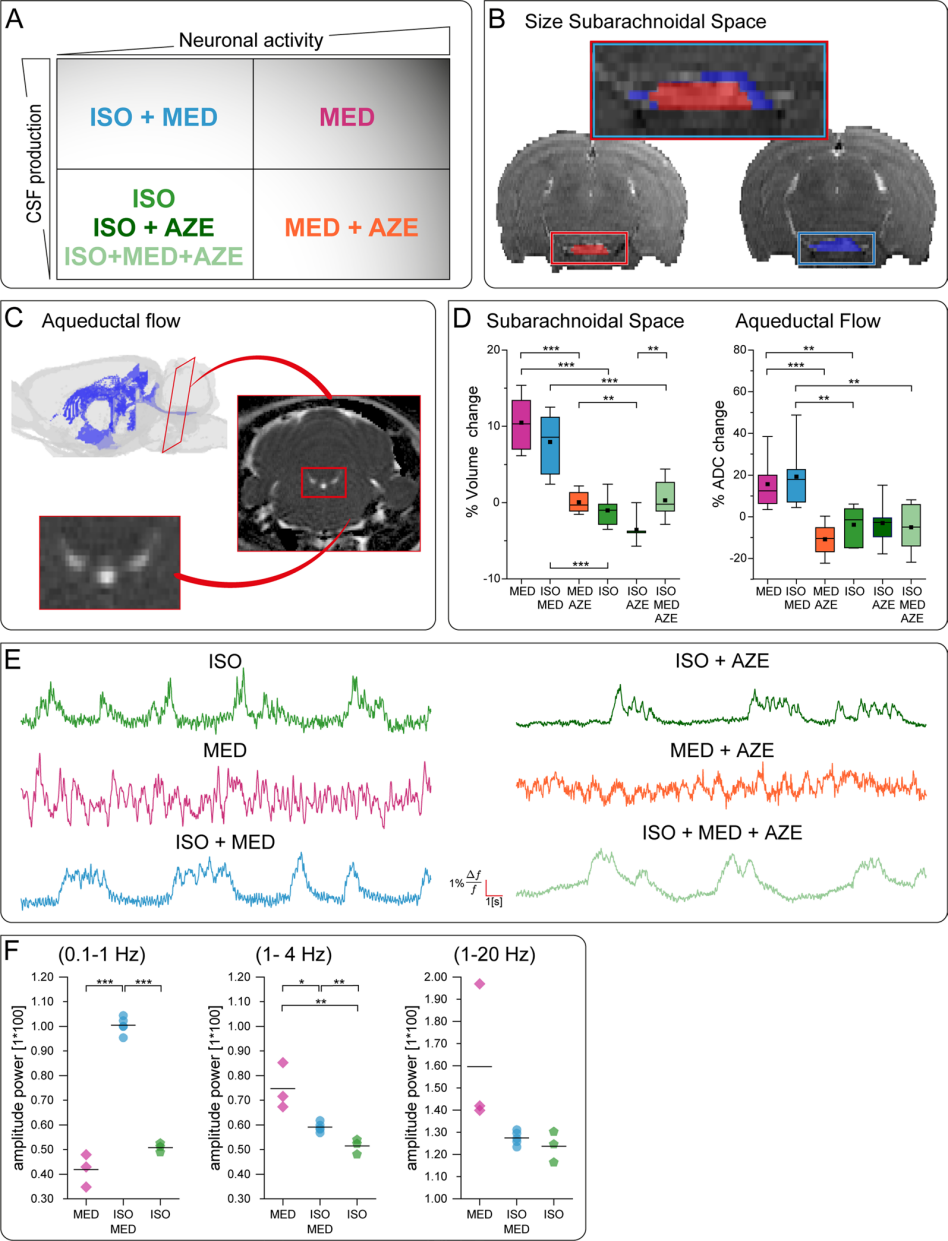
Validation of pharmacological modulation of CSF formation and neuronal activity.** A** Four conditions with high and low CSF formation combined with high and low neuronal activity were achieved by different anesthesia protocols. **B** Representative axial T2w images depict subarachnoidal space under ISO (red) and MED (blue). Inset shows superposition of volumes under the two conditions. **C** 3D visualization indicates position of the slice for DWI (red) and shows the fluid-filled volume (blue) in the rat brain derived from anatomical images. DWI (right) was used for measuring ADC in the aqueduct (central bright structure in the zoomed image) as surrogate for CSF flow. **D** Boxplots display percent change of volume of subarachnoidal space or of aqueductal flow. Kruskal–Wallis test indicated an effect of experimental condition on volume of subarachnoidal space (p < 0.001). ANOVA test showed an effect of experimental condition on aqueductal flow (p < 0.001). Significant differences between groups determined by post-hoc analysis with Mann–Whitney-U and Student’s t-test, respectively, are indicated in the graphs (ISO n = 6; MED n = 6; ISO+MED n = 6; ISO+AZE n = 6; MED+AZE n = 6; ISO+MED+AZE n = 7). **E** Exemplary traces show optical Ca^2+^-recordings of spontaneous brain activity in neocortex. **F** Analysis of power spectra of Ca^2+^-recordings under MED, ISO+MED and ISO for the frequency ranges of 0.1–1 Hz, 1–4 Hz and 1–20 Hz respectively (MED N = 3 animals; ISO+MED N = 5 animals; ISO N = 3 animals; each averaged over 30 spectra per animal). ANOVA test indicated effect of experimental condition for 0.1–1 Hz (p < 0.001) and for 1–4 Hz (p < 0.01). Significant differences between groups determined by post-hoc analysis with Student’s t-test are indicated in the graphs.* p < 0.05, ** p < 0.01, *** p < 0.001

ADC maps from scans before and after switch of anesthesia condition were calculated from DTI data using Paravision 5.1 by applying a mono-exponential fit. For ROI-analysis neocortex was manually segmented in one axial slice, matching the region selected for Ca^2+^-recordings. Similarly, for aqueductal flow, ADC maps were calculated from DWI data and aqueduct was manually segmented in the slice depicting the aqueduct. In order to determine the size of the subarachnoidal space, the basal cistern was manually segmented in axial T2w scans before and after anesthetic switch (Fig. 1). Resulting values were divided by the initial value (ISO condition), to reveal relative changes from BL.

### Power spectra of calcium traces

High resolution calcium traces (sampling rate 2 kHz) were analyzed using a customized MATLAB script. First data were smoothed using a lowpass filter (sampling rate 60 Hz; upper bound 200 Hz; lower bound 20 Hz; damping 40 Hz). To detect possible time dependent changes, every other 6 min, a 2-min trace was extracted from the original data, resulting in 30 spectra per animal. For each of these cropped traces a power spectrum analysis was performed and the frequency ranges 0.1–1 Hz, 1–4 Hz and 1–20 Hz were summed up, were analyzed separately and subsequently averaged per animal.

### Mechanism-independent two-compartment model

We used a two-compartment model to quantify efficiency of solute clearance. We choose neocortex (as defined by the Waxholm Rat atlas) because of the decent size of this brain region and the fact, that Ca^2+^-recordings, ADC data and TSCs were available from this brain region. Neocortex was subdivided into two compartments, one coherent outer compartment that completely surrounded one inner compartment.

The following differential equation describes the model, where $\widetilde {{I}_{in}}$ is the signal of the inner compartment and $\widetilde {{I}_{out}}$ is the signal of the outer compartment. *k*_1_ and *k*_2_ denote the exchange rates between these compartments. Thus, ${k}_{1}\cdot \widetilde {{I}_{out}}(t)$ is the signal amount entering the inner compartment from the outer compartment at each time and ${k}_{2}\cdot \widetilde {{I}_{in}}(t)$ is the signal amount leaving the inner compartment entering the outer compartment at each time.

3 $$\frac{d}{dt} {\widetilde{{I}_{in}}}(t)={k}_{1}\cdot {\widetilde {{I}_{out}}}(t)-{k}_{2}\cdot {\widetilde {{I}_{in}}}(t)$$

With a given $\widetilde {{I}_{out}}$ and the exchange rates *k*_1_ and *k*_2_, $\widetilde {{I}_{in}}$ can be determined. In this case a time signal curve of the outer compartment can serve as an initial condition being equal to $\widetilde {{I}_{out}}$, but the exchange rates *k*_1_ and *k*_2_ are unknown and thus need to be estimated from the known sampled functions *I*_*out*_(t) and *I*_*in*_(t) from the experiments.

The differential equation was solved via Laplace transformation.

4 $$\widetilde {{I}_{in}}(t)={k}_{1}\cdot \left[{e}^{-{k}_{2}\cdot t}\star \widetilde {{I}_{out}}(t)\right]={k}_{1}\cdot \int\nolimits_{-{\infty }}^{{\infty }}{e}^{-{k}_{2}\cdot t}\cdot \widetilde {{I}_{out}}(t-l)\text{dl}$$

where $\star$ represents a convolution, over the time parameter and is performed as shown in the integral. Since $\widetilde{{I}_{in}}$ and $\widetilde{{I}_{out}}$ are given by the measured TSCs from the inner and outer compartment, they can be replaced by *I*_*out*_(t) and *I*_*in*_(t), two unknown parameters ${k}_{1},{k}_{2}$ remain. Optimal parameters ${k}_{1},{k}_{2}$ in $\mathbb{R}$ were found by minimizing the difference between the *I*_*in*_ from the TSC and the Laplace transform of Eq. (3).

5 $$\text{optimal}\left({k}_{1},{k}_{2}\right)=\underset{{k}_{1},{k}_{2}\in R}{min}\left\| {I}_{in}\left(t\right)-\stackrel{\sim}{{I}_{in}}(t)\right\|_{{L}^{2}}$$

where the L^2^-norm is the Euclidean norm of the function values and used to determine the distance between the measured *I*_*in*_(t) and the calculated $\widetilde{{I}_{in}}$ using (4) with the measured *I*_*out*_(t). To find the optimal size of the inner and outer compartment we performed this procedure for each animal by stepwise changing the thickness from 1 to 17 voxels of the outer compartment and calculated the difference between the *I*_*in*_ from the TSC and the optimal one from Eq. (4), denoted as ${I}_{in}^{\text{sol}}(t)$, for each thickness. ${I}_{in}^{\text{sol}}(t)$ is calculated using the determined k_1_ and k_2_ from (4).

6 $$\text{optimals}=\underset{s\in 1,\ldots ,18}{min}\left\| {I}_{in}\left(t\right)-{I}_{in}^{\text{sol}}(t)\right\|_{{L}^{2}}$$

Via multiplication of the TSC with the exchange rates, the exchanged volumes at every time point were calculated. As the exchange rates are constant the signal intensity changes over time. An exponential decay may be part of the signal intensity but is not assumed using this approach.

7 $${k}_{1}\cdot {I}_{out}(t), {k}_{2}\cdot {I}_{in}(t)$$

Since it was assumed that ${k}_{1},{k}_{2}$ do not change over time, these functions are multiples of ${I}_{out}(t)\;\text{and}\; {I}_{in}(t)$. The total volumes are defined by their area under the curves:

8 $${V}_{out}=\int\nolimits_{0}^{end\;of\;experiment}{I}_{out}(t)\text{dt}$$

9 $${V}_{in}=\int\nolimits_{0}^{end\;of\;experiment}{I}_{in}(t)\text{dt}$$

Consequently, the exchanged volumes during the total experiment over time are:

10 $${V}_{out\to in}=\int\nolimits_{0}^{end\;of\;experiment}{k}_{1}{I}_{out}(t)\text{dt}={k}_{1}\cdot {V}_{out}$$

11 $${V}_{in\to out}=\int\nolimits_{0}^{end\;of\;experiment}{k}_{2}{I}_{in}(t)\text{dt}={k}_{2}\cdot {V}_{in}$$

With ${V}_{out\to in}$ being the volume that is exchanged from the outer to the inner compartment and ${V}_{in\to out}$ the volume that is exchanged from the inner to the outer compartment.

The net exchange ratio (NER) is defined by the ratio of ${V}_{out\to in}$ and ${V}_{in\to out}$,

12 $$NER=\frac{{V}_{in\to out}}{{V}_{out\to in}}.$$

### Statistical analysis

Continuous variables were reported as mean ± standard deviation (SD) when normally distributed and as medians (interquartile range) otherwise. Normal distribution was assessed using a Kolmogorov–Smirnov test. A parameter was regarded as normally distributed, if normal distribution was observed for all anesthetic conditions and for all brain regions. To determine whether there was an effect of anesthetic condition, we applied an ANOVA test or a Kruskal–Wallis test depending on normality. Post-hoc comparisons between groups were performed for continuous variables, using a 2-tailed unpaired Student’s t-test or a Mann–Whitney-U test depending on normality. The power spectra of calcium traces were analyzed using linear mixed effect model with time and anesthetic condition as parameters. Hypothesis testing was two-tailed. All p-values < 0.05 were considered statistically significant. Data were presented as boxplots: the central line indicates median, the central mark indicates mean, bottom and top edges of the box indicate the 25th and 75th percentiles, respectively, whiskers indicate the 5th and 95th percentiles. Significant differences are marked with one asterisk if p < 0.05, two asterisks if p < 0.01 and three if p < 0.001. All statistical analyses were performed using MATLAB 2020b.

## Results

### Validation of pharmacological modulation of CSF formation and neuronal activity

In a first set of MRI experiments, we verified that the different pharmacological regimens correctly modeled conditions of high and low CSF formation in combination with high and low brain activity (Fig. 1A). To validate the effect of anesthetic condition on CSF formation, changes in the size of the basal cisterns in T2w MRI images (Fig. 1B) and ADC in the aqueduct (Fig. 1C) were measured. Under the anesthetic conditions MED and ISO+MED, a significant enlargement of the basal cisterns and a significant increase of aqueductal flow were observed, compared to ISO anesthesia (Fig. 1D). With application of AZE, significantly lower values of both subarachnoidal space and aqueductal flow were observed with MED and ISO+MED. Although not being significant, aqueductal flow seemed to be further reduced when AZE was applied under ISO anesthesia.

These data show that both conditions MED and ISO+MED resulted in enhanced CSF formation, while ISO and all conditions containing AZE resulted in low CSF formation.

To validate the effect of anesthetic conditions on brain state, we monitored spontaneous neuronal activity in neocortex via optical Ca^2+^-recordings during MRI measurements (Fig. 1E). We observed two different brain states, silent periods with burst-like phases during ISO anesthesia and persistent neuronal activity with MED (Fig. 1E), in accordance with previous observations [29, 30]. The combination of both anesthetics revealed suppressed activity with burst-like phases comparable to the ISO anesthesia. Since the burst-like phases during ISO and ISO+MED anesthesia occurred at a frequency of less than 1 Hz, we analyzed the power spectra of Ca^2+^-recordings below 1 Hz. In each animal 30 power spectra were sampled over the total time of dynamic contrast-enhanced imaging of 6 h to detect a time dependent effect. A linear mixed effect model analysis demonstrated no time dependent change for any of the frequency bands inspected (0.1–1 Hz, p = 0.41; 1–4 Hz, p = 0.99; 1–20 Hz, p = 0.75), whereas the anesthetic condition demonstrated an effect (all frequency bands, p < 0.001). We averaged power spectra per animal and performed group comparisons. ANOVA showed a significant effect of condition for 0.1–1 Hz (p < 0.001) and 1–4 Hz (p < 0.01). At frequencies of 0.1–1 Hz we observed higher power with ISO+MED (Student’s t-test p < 0.001) and a trend towards higher power with ISO (Student’s t-test p = 0.09) compared to MED. Analyzing the delta frequency band (1–4 Hz) a higher power with MED compared to ISO (Student’s t-test p < 0.05) and compared to ISO+MED (Student’s t-test p < 0.01) was detected, in accordance with previous studies [38]. The power was also higher with ISO-MED compared to ISO (Student’s t-test p < 0.01).

These data show that MED sedation generated a state of higher neuronal activity compared to ISO and ISO+MED. We were, therefore, able to generate all combinations of high and low CSF formation with high and low neuronal activity by combining ISO, MED and AZE (Fig. 1A). MED induced high CSF formation and high neuronal activity; ISO+MED induced high CSF formation and lower neuronal activity; the application of AZE led to lower CSF formation for MED, ISO+MED and ISO.

### CSF formation drives CSF distribution in the brain

In the next set of experiments, we investigated whether enhanced CSF formation influenced CSF distribution, which is a precondition for permeation of brain parenchyma.

**Fig. 2:**
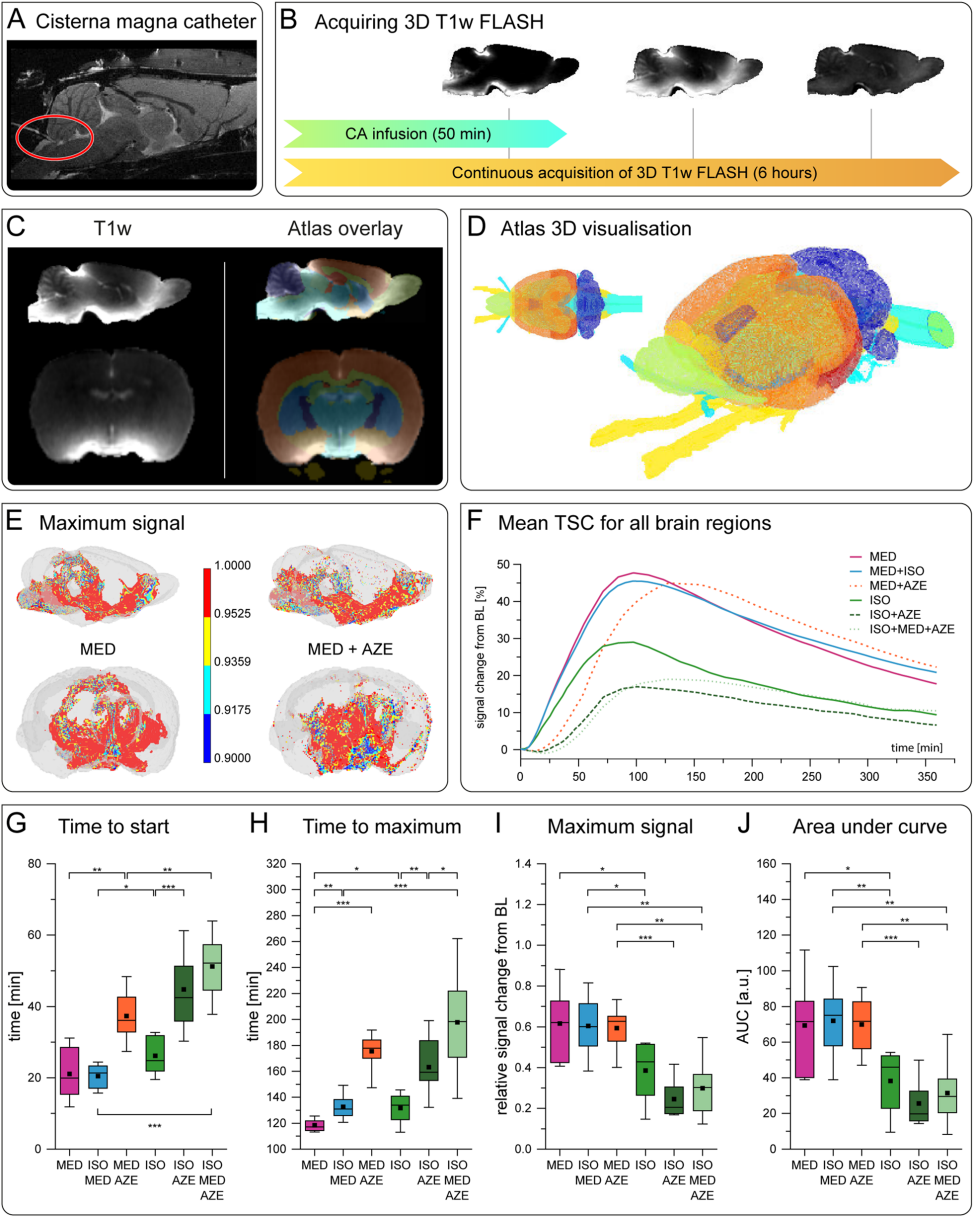
Solute distribution in the brain assessed by DCE MRI. **A** Catheter placement (red circle) for CA injection in the cisterna magna is indicated in a representative sagittal anatomical image. **B** 3D T1w MRIs using a FLASH sequence were continuously acquired before, during and after injection of CA into the cisterna magna for a total of 6 h. **C** Registration of high-resolution 3D T1w MRI data onto the Waxholm space atlas. Some exemplary brain regions are colored: orange = cortex, purple = cerebellum, light green = olfactory bulb, green = corpus callosum, light blue = striatum, dark blue = hypothalamus, cyan = thalamus). **D** 3D visualization of some exemplary brain regions of the Waxholm space atlas orange = cortex, yellow = optic nerve, purple = cerebellum, light green = olfactory bulb, blue = spinal cord). **E** 3D visualization of relative maximum signal after contrast agent application under MED (left), and MED+AZE (right). **F** Mean time signal curves (TSC) for the different anesthetic regimens group-averaged over all brain regions. Boxplots of TSC parameters time to start (**G**), time to maximum (**H**), maximum signal (**I**), and AUC (**J**) averaged over all 79 brain regions from all animals (ISO n = 8; MED n = 7; ISO+MED n = 8; ISO+AZE n = 10; MED+AZE n = 8; ISO+MED+AZE n = 8). Kruskal–Wallis test indicated significant differences between groups for all TSC parameters (p < 0.001), the post-hoc analysis was performed using Mann–Whitney-U test (asterisks indicate p-values in the graphs). * p < 0.05, ** p < 0.01, *** p < 0.001

For this purpose, we injected the CA Gadobutrol into the cisterna magna (Fig. 2A) and measured its distribution in the brain by repeated 3D T1w MRI over 6 h under each of the six conditions (Fig. 2B).

High resolution 3D T1w MRI data were mapped onto the Waxholm space atlas (Fig. 2C, D), and time signal curves (TSCs) were obtained for whole brain (Fig. 2F) and 79 separate brain regions (Additional file 3).

Within the first 2 h, the injected tracer spread from the cisterna magna along the basal arteries (Fig. 2B, E) to the circle of Willis, and from there into the brain parenchyma [23, 47]. Signal increased after some delay, reached a maximum and then decreased again (Fig. 2F and Additional file 3). To assess the distribution kinetics quantitatively, we measured time to start, time to maximum, area under the curve (AUC), and maximum signal. We compared parameters averaged over all brain regions. Kruskal–Wallis test indicated an effect of experimental condition regarding the distribution parameters time to start (p < 0.001), time to maximum (p < 0.001), area under the curve (p < 0.001) and maximum signal (p < 0.001). For the three anesthetic conditions containing AZE, we observed a significantly later time to start and time to maximum (Fig. 2G, H). CA arrived later when comparing MED+AZE with MED (Mann–Whitney-U test p < 0.01), ISO+MED+AZE with ISO+MED (Mann–Whitney-U test p < 0.001), and under ISO+AZE than under ISO (Mann–Whitney-U test p < 0.001). Additionally, TSC showed similar or lower maximum amplitudes under MED+AZE compared to MED (not significant) (Fig. 2I), and under ISO+MED+AZE compared to ISO+MED (Mann–Whitney-U test p < 0.01). There was no significant difference in AUC between MED and MED+AZE. Under ISO+MED and under MED all brain regions showed larger AUC than under ISO (Mann–Whitney-U test p < 0.01, p < 0.05, respectively) (Fig. 2J). In general, when CSF formation was high, CA reached most regions earlier and higher maximum signal was observed. Vice versa, reduced CSF formation decelerated the distribution of CA in the brain.

**Fig. 3:**
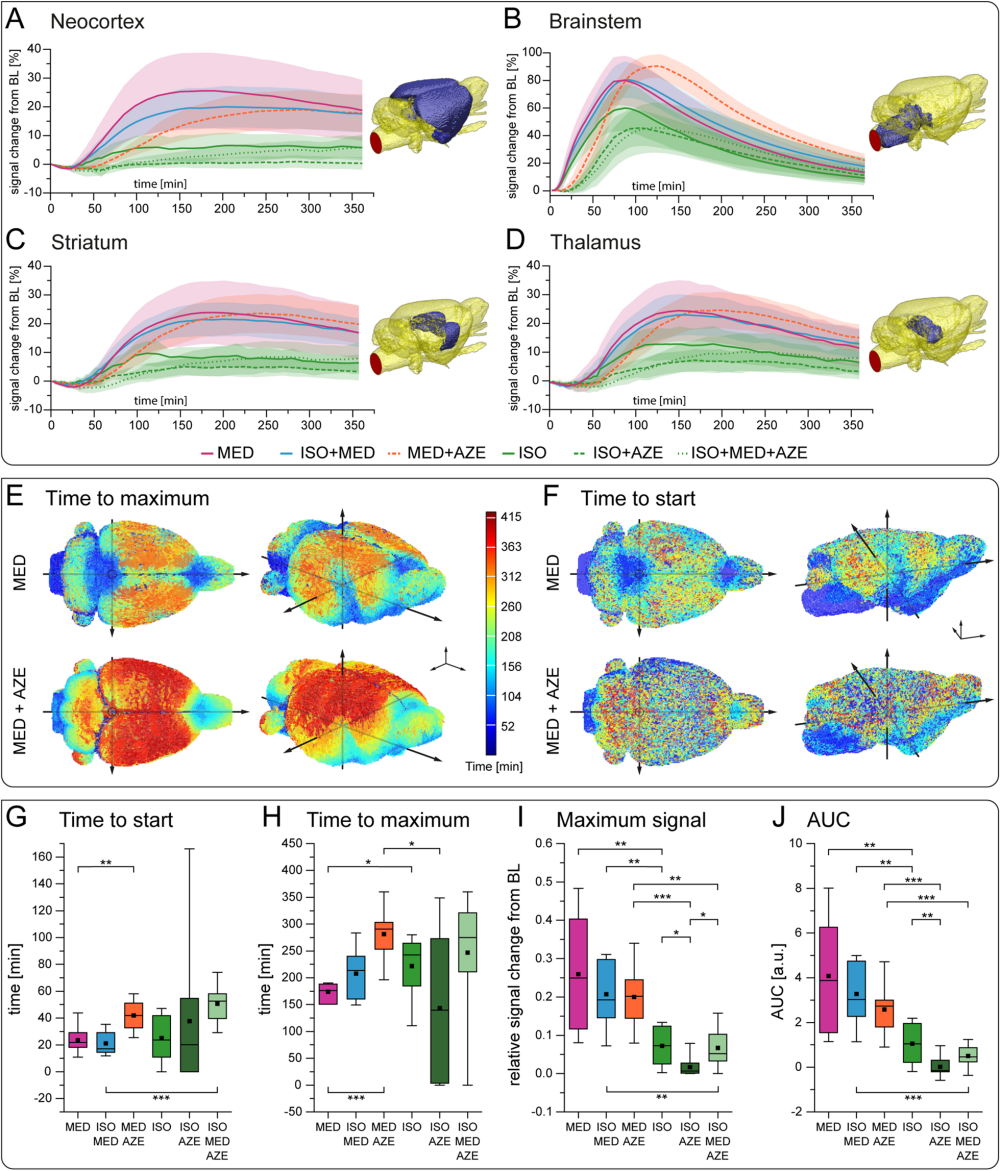
Brain region-wise analysis of tracer distribution. TSCs for all anesthetic conditions represented by mean (solid or broken lines) and confidence interval (shaded area) in neocortex (**A**), brainstem (**B**), striatum (**C**) and thalamus (**D**). Brain region location is indicated in blue in volume renderings. **E** 3D visualization of time to maximum, **F** 3D visualization of time to start for MED and MED+AZE. Boxplots display time to start (**G**), time to maximum (**H**), maximum signal (**I**), and AUC (**J**) of neocortex. Animal numbers for plots in **A**–**D** and **G**–**J** were ISO n = 8; MED n = 7; ISO+MED n = 8; ISO+AZE n = 10; MED+AZE n = 8; ISO+MED+AZE n = 8. Kruskal–Wallis test showed an effect of experimental condition for all TSC parameters (time to start: p = 0.011; time to maximum: p = 0.007; maximum signal: p < 0.001; area under the curve: p < 0.001), the post-hoc analysis was performed using Mann–Whitney-U test. Significant differences are indicated in the graphs. BL: baseline. * p < 0.05, ** p < 0.01, *** p < 0.001

Region-specific analysis (Fig. 3A–D, Additional files 3, 4) showed that in all individual brain regions signal initially increased and, after reaching a maximum, decreased again. However, comparing different brain regions demonstrated substantial differences in distribution kinetics regarding maximum signal, time to start and time to maximum. Exemplarily, brain stem (Fig. 3B), which is located close to cisterna magna, showed an earlier signal increase than neocortex, striatum or thalamus (Fig. 3A, C, D), which are located more remote from the tracer injection site. Under conditions with reduced CSF formation (ISO, MED+AZE, ISO+AZE, ISO+MED+AZE), brainstem showed a higher signal increase than other brain regions. 3D visualization of the time to start and time to maximum (Fig. 3E, F) showed that tracer remained longer in the basal cistern when CSF production was low. Consequently, higher signal increase was observed in the brainstem, compared to the neocortex (Fig. 3A).

Comparing different anesthetic regimens in each brain region, it became evident that under MED and ISO+MED onset and slope of TSCs were similar, while reduced CSF formation resulted in delayed onset or reduced maximum signal. Quantification is exemplarily shown for neocortex (Fig. 3G–J).

Under different conditions, the tracer reached neocortex at different speed and to various spatial extent. Though, generally, signal enhancement was first observed in the periphery and then centrally. Reduced CSF formation by application of AZE, resulted in a significantly delayed tracer distribution as illustrated in 3D brain depictions (Fig. 3E, F). Accordingly, time to start was shorter under high CSF formation conditions (MED, Mann–Whitney U test, p < 0.01 and MED+ISO, Mann Whitney U test p < 0.001) compared to low CSF formation conditions with AZE (Fig. 3G). Time to maximum, was significantly delayed under low CSF formation conditions when comparing MED to MED+AZE (Mann Whitney U test, p < 0.001) and ISO (Mann Whitney U test, p < 0.05; Fig. 3H). Both parameters, maximum signal amplitude and AUC, showed higher values under high CSF formation conditions (MED and MED+ISO) and MED+AZE than under low CSF formation conditions (Fig. 3I, J).

In summary, these data demonstrate that reduced CSF formation resulted in delayed onset and maximum signal change as well as equal or lower signal amplitude. The two conditions with enhanced CSF formation (MED, MED+ISO) showed similar distribution of tracer into the brain, despite different states of neuronal activity.

### Solute clearance depends on water diffusivity in the brain

**Fig. 4:**
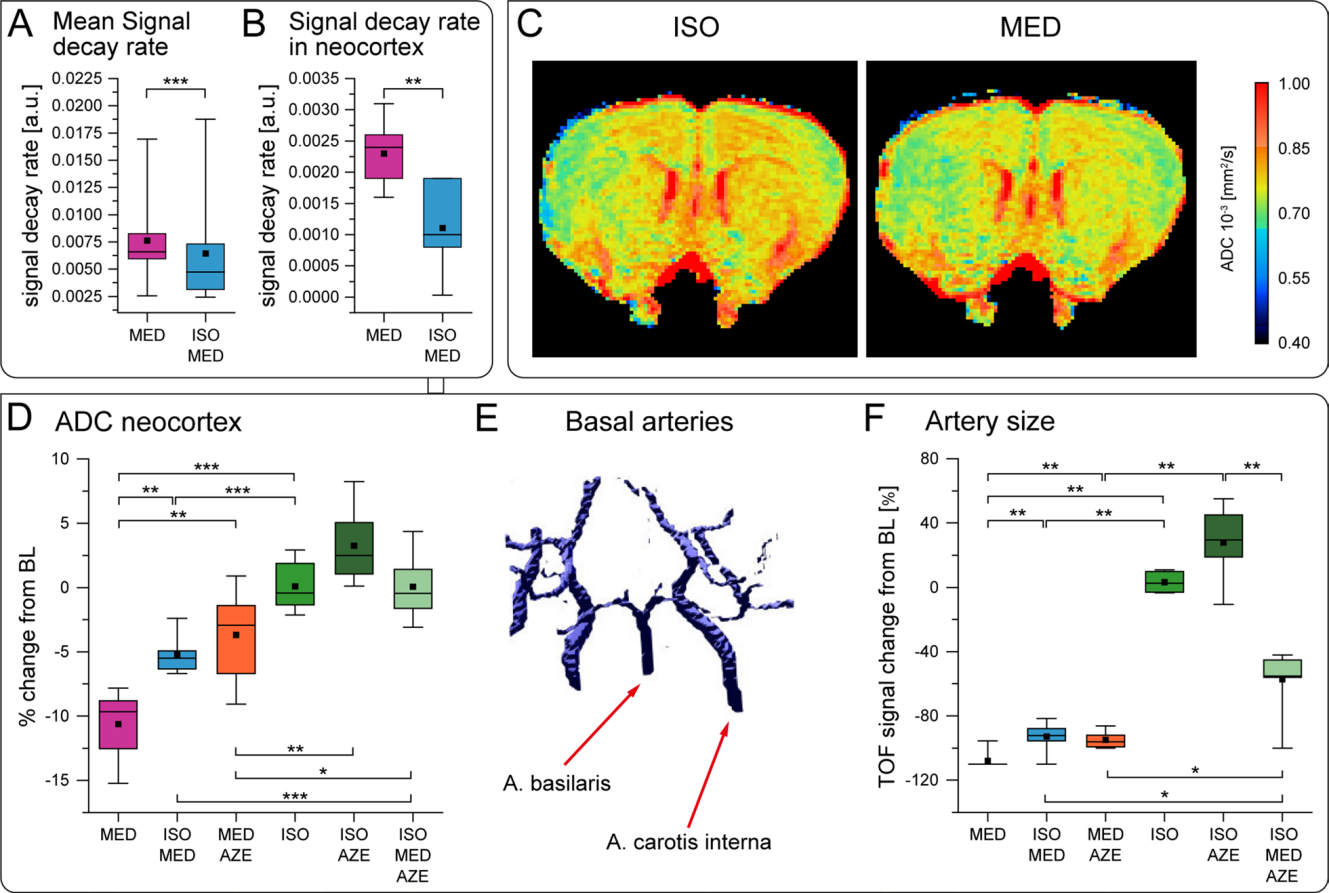
Solute clearance and water diffusivity in the brain. **A** Signal decay rate of TSCs under MED and ISO+MED for all brain regions, calculated by mono-exponentially fitting of individual region-specific TSCs over the time period between 90 min after TSC maximum and end of data acquisition (Mann–Whitney-U test). **B** Signal decay rate of TSC in neocortex under MED and ISO+MED (MED n = 7; ISO+MED n = 8) (Mann–Whitney-U test). **C** ADC maps of an exemplary axial slice of the rat brain under ISO and after switching to MED. **D** Boxplot displays % ADC change from baseline. ANOVA test indicated effect of experimental condition on diffusivity (p < 0.001). Post-hoc analysis was performed using Student’s t-test. **E** 3D maximum intensity projection of an exemplary TOF-MRI depicting the basal arteries under ISO. **F** Boxplot showing the % change of TOF signal (Kruskal–Wallis-Test showed significant difference p < 0.001). Post-hoc analysis was performed using Mann–Whitney-U test. Animal numbers for boxplots shown in **D** and **F** were ISO n = 6; MED n = 6; ISO+MED n = 6; ISO+AZE n = 6; MED+AZE n = 6; ISO+MED+AZE n = 7. * p < 0.05, ** p < 0.01, *** p < 0.001

In contrast to tracer distribution into the brain, clearance of the tracer, observed as decay of the TSCs was faster under MED with high neuronal activity, compared to ISO+MED (Fig. 4A, B, Additional files 3, 5). It must be noted that mono-exponential fitting of the decay was only possible for TSCs exhibiting a (sufficient) maximum signal amplitude followed by a signal decrease during the 6 h of observation. For conditions with AZE and most brain regions under ISO the signal maximum was either delayed or had low amplitude, and fitting of a signal decay rate was not possible.

The efficiency of solute clearance in the brain has previously been related to the brain state, which has been related to volume of the extracellular space [5, 18, 21], which, in turn, has been related to diffusivity [48–50]. We therefore performed DWI MRI under the different anesthetic conditions, and measured ADC in neocortex (Fig. 4C, D), where also neuronal activity had been monitored by Ca^2+^-recordings.

Interestingly, ADC in neocortex was higher under all three conditions with AZE when compared to the respective conditions without AZE. Conditions with high neuronal activity (MED, MED+AZE) showed significantly lower ADC than the corresponding conditions with low neuronal activity (ISO+MED, ISO+MED+AZE; Fig. 4D).

To verify that vasoconstrictive or dilative effects did not have a major influence on ADC, artery volume was assessed using time of flight (TOF) MRI angiography as surrogate (Fig. 4E). The most prominent observation was a reduced artery volume under all conditions with MED, confirming its vasoconstrictive effects [30, 51] as opposed to the vasodilatory effect of ISO [52] (Fig. 4F). The observed vasoconstriction under MED excludes a compression effect by enlarged vasculature as major cause of ADC reduction during high neuronal activity. Adding AZE resulted in bigger artery volumes compared to the respective condition without AZE. A vasodilatory effect of carboanhydrase inhibitors has previously been reported [53].

In summary, we conclude that under conditions with high neuronal activity, and reduced diffusivity, the tracer is removed from the brain faster, most likely still being in the PVS and not within interstitial brain parenchyma. This conclusion is in line with previous observations that solutes permeate deeper into brain parenchyma when diffusivity is high due to larger interstitial spaces [5]. Providing such conditions, we assume that low neuronal activity allows for more efficient solute clearance from the brain parenchyma since fluids diffuse more efficiently into interstitial spaces [5, 38].

### Modeling solute clearance

**Fig. 5:**
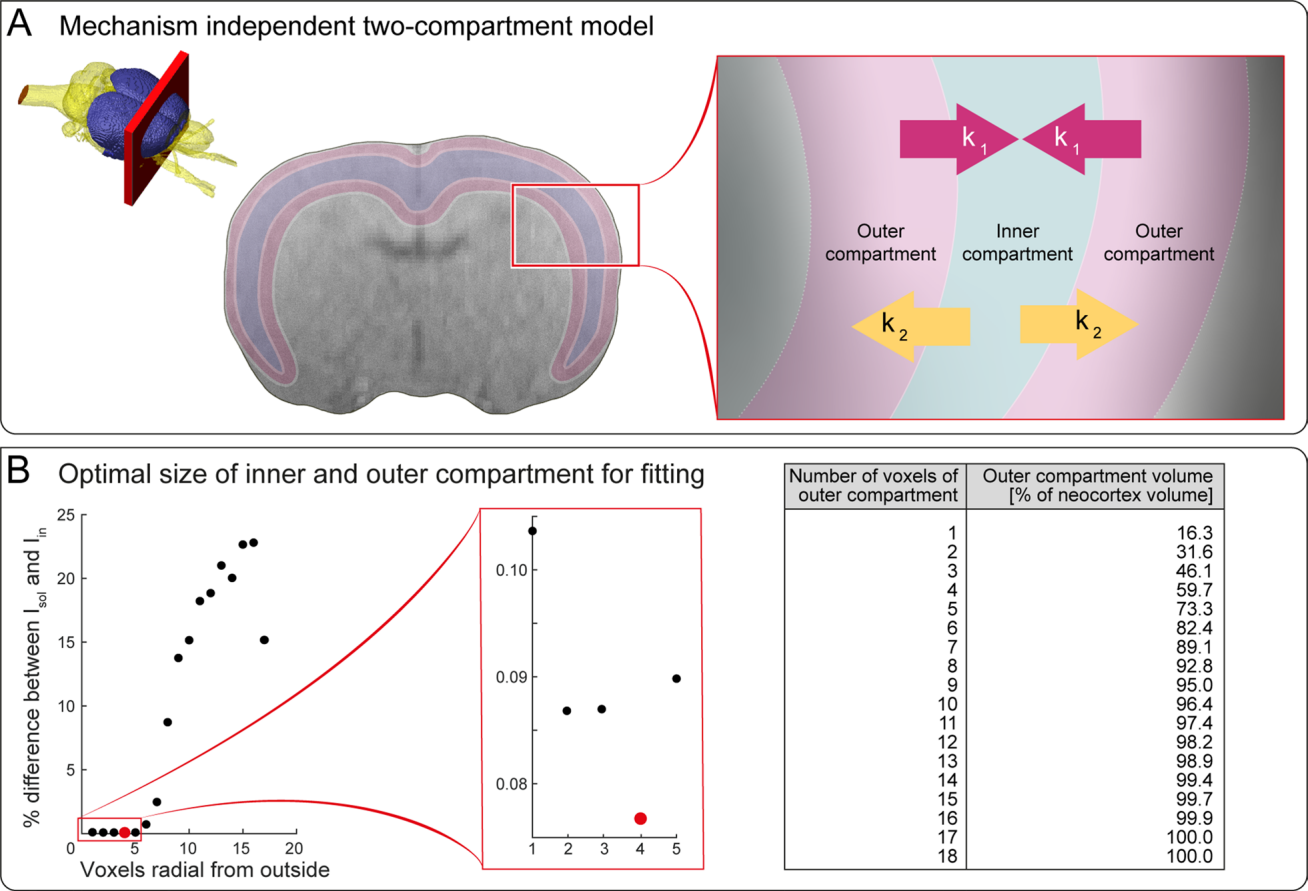
The mechanism-independent two-compartment model. **A** Scheme shows neocortex overlaid on an exemplary precontrast T1w axial MRI. The close-up illustrates exchange rates of *k*_1_, *k*_2_ between the two compartments, while CSF has to pass the outer regions of cortex to reach the inner regions and then has to pass the outer region again before being cleared from the brain. **B** % Difference between *I*_*in*_^*sol*^ and *I*_*in*_ as a function of thickness of outer compartment. Each data point was calculated using the average TSC from n = 49 animals. Minimum difference (indicated in red) was observed with a thickness of 4 voxels for the outer compartment. Table shows relation between number of voxels and compartment volume relative to total neocortex volume

In the final step, we aimed to provide a measure of how efficiently solutes are cleared from the brain. We hypothesize that clearance can be characterized by a net exchange ratio. For this purpose, we developed a pathway and mechanism-independent two-compartment model (MITCM) (Fig. 5A, B). The MITCM makes grossly simplifying assumptions, describing the exchange between two compartments independent of a fluid pathway or a physical mechanism. The underlying rationale is that for efficient solute clearance, CSF has to pass outer parts of a brain region to reach inner parts, and has to pass the outer part again before being cleared from the brain (Fig. 5A). Due to the fact that MRI does not provide microscopic resolution, the two compartments cannot be directly assigned to an anatomical equivalent. We applied the MITCM to neocortex, and subdivided the brain region into an inner compartment that was entirely surrounded by an outer compartment, which was independent from the anatomical cortical layer structure. The outer compartment was defined in atlas-registered MRI data with a constant (voxel) thickness from the outer edges of neocortex, resulting in a closed envelope with even thickness on the dorsal/lateral and ventral/medial side (Fig. 5A). The remaining, enclosed volume of neocortex was assigned to the inner compartment. For both compartments, the specific TSCs *I*_*out*_ and *I*_*in*_ were obtained by summing MRI signals over all voxels of inner and outer compartment for each animal. This procedure was repeated for different compartment thicknesses (i.e. volume fractions) (Fig. 5B).

Using these data, solute exchange in the MITCM can be described by differential Eq. (3).

By solving Eq. (3) for each single animal, we obtained *k*_1_, the exchange rate from outer to inner compartment and *k*_2_, the exchange rate from inner to outer compartment. Equation (4) allowed for predicting the TSC of the inner compartment *I*_*in*_^*sol*^ on the basis of *k*_1_, *k*_2_ and *I*_*out*_. Ideally, the solution *I*_*in*_^*sol*^ matches the measured *I*_*in*_. However, since the two compartments are an artificial construct, and *k*_1_ and *k*_2_ strongly depended on *I*_*out*_ and *I*_*in*_, which again depended on the volume fraction of the inner and outer compartment, we solved Eq. (3) for all possible volume fractions (Additional file 6). The smallest deviation between *I*_*in*_^*sol*^ and *I*_*in*_ was obtained for an outer compartment with a thickness of four voxels, which corresponded to 60% of total neocortex (Fig. 5B). Therefore, this definition of outer and inner compartment was used to calculate volume of tracer in outer compartment *V*_*out*_ and inner compartment *V*_*in*_, and exchange rates.

**Fig. 6:**
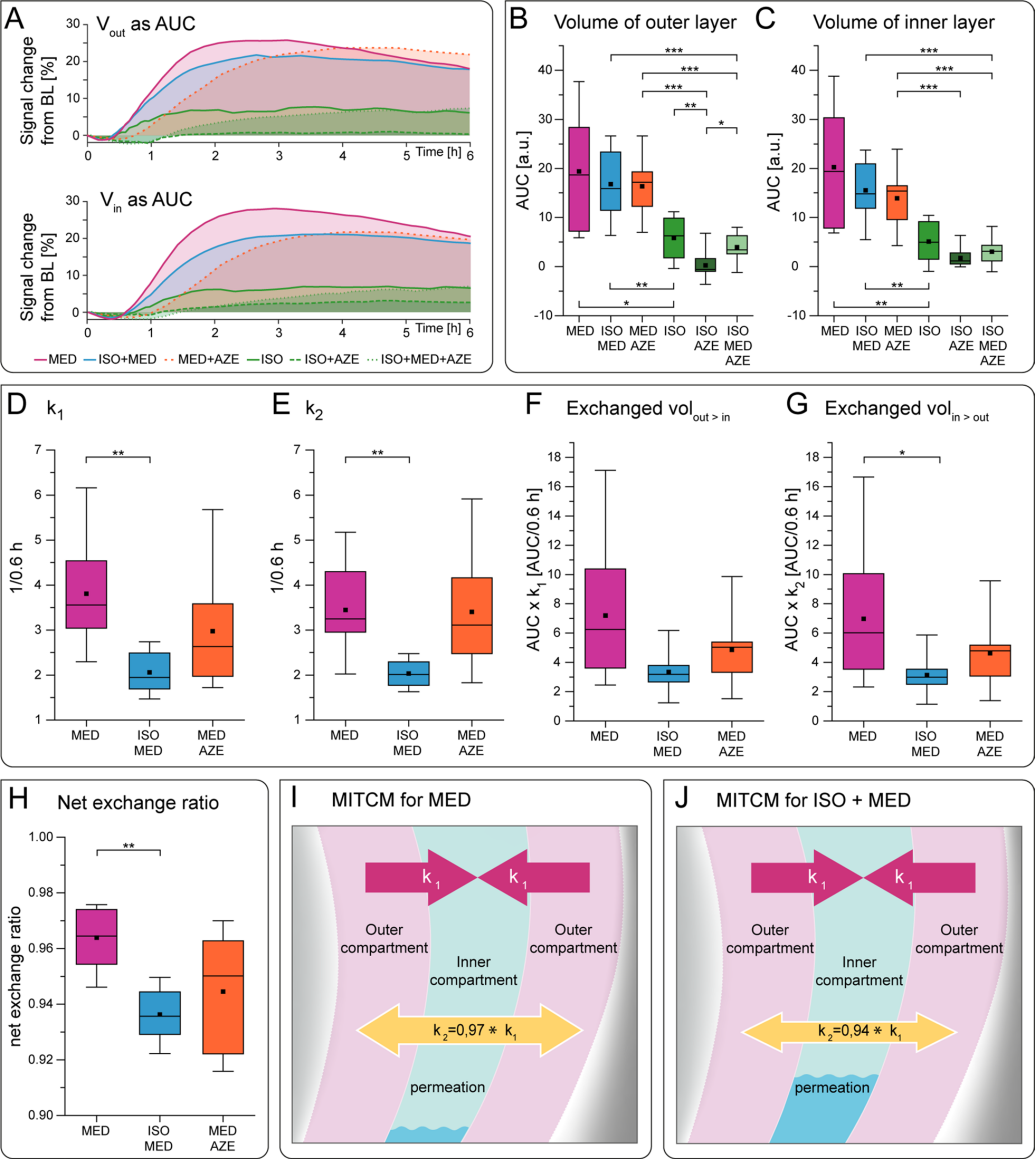
Modeling solute clearance. **A** Mean TSCs of outer and inner compartment for each group; AUCs represent CSF volumes. **B**, **C** Calculated CSF volumes of inner and outer compartment (Kruskal–Wallis test indicated an effect of experimental condition for both p < 0.001, post-hoc analysis was performed using Mann–Whitney-U test). **D**, **E** Exchange parameters ${k}_{1},{k}_{2}$ obtained by solving the differential Eq. (3), describing the exchange between outer and inner compartment (ANOVA test indicated an effect of experimental condition for both p = 0.018 for k_1_ and p = 0.016 for k_2_, post-hoc analysis was performed using Student’s t-test). **F**, **G** Exchanged volumes from outer to inner compartment (ANOVA test indicated no significant difference p = 0.10, post-hoc analysis was performed using Student’s t-test) or from inner to outer compartment (ANOVA test showed no significant effect of experimental condition p = 0.09, post-hoc analysis was performed using Student’s t-test) obtained by multiplying the exchange rates with the corresponding volume. **H** Net exchange ratio between the inner and outer volumes (ANOVA test showed an effect of experimental condition p = 0.007, post-hoc analysis was performed using Student’s t-test). **I** Schematic illustration of the two-compartment model for MED with almost similar exchange between inner and outer compartment. **J** Schematic illustration of the two-compartment model for ISO+MED with lower exchange ratio and higher permeation when compared to MED. Animal numbers used for all plots were ISO n = 8; MED n = 7; ISO+MED n = 8; ISO+AZE n = 10; MED+AZE n = 8; ISO+MED+AZE n = 8; a. u.: arbitrary units. * p < 0.05, ** p < 0.01, *** p < 0.001

For the anesthetic conditions ISO, ISO+AZE, ISO+MED+AZE, all with low CSF production, both tracer volumes *V*_*out*_ and *V*_*in*_ were small, and did not provide reliable results in the following calculations (Fig. 6A–C), and were therefore not considered further. The exchange rates *k*_1_ and *k*_2_ were significantly larger for MED compared to ISO+MED. For MED+AZE no significant differences were observed (Fig. 6D, E).

To characterize exchange between the two compartments, the amount of tracer *V*_*out→in*_ that was exchanged from outer to inner compartment was obtained by multiplying the exchange rate *k*_1_ with the amount of tracer of the outer compartment *V*_*out*_, and accordingly *V*_*in→out*_ by multiplying *k*_2_ with *V*_*in*_. There was no significant difference of *V*_*out→in*_ between the three conditions (Fig. 6F), indicating that a similar amount of CSF volume moved from outer to inner compartment. In contrast, *V*_*in→out*_ was significantly lower for ISO+MED compared to MED, but similar when compared to MED+AZE (Fig. 6G). This indicated that under ISO+MED more CSF tracer remained in the inner compartment, compared to MED.

To quantify tracer displacement, the net exchange ratio, defined as the ratio of *V*_*out→in*_ and *V*_*in→out*_ was calculated. For MED, the net exchange ratio was 0.964 ± 0.011 (Fig. 6H), indicating an almost balanced fluid exchange between both compartments. The net exchange ratio under ISO+MED was 0.936 ± 0.010 and significantly (Student’s t-test p < 0.01) lower compared to MED, indicating that more CSF tracer, relative to total tracer flux, remained in the inner compartment with ISO+MED compared to MED (Fig. 6H). For MED+AZE no significant difference compared to the other two regimens was observed (Fig. 6H).

These results were obtained by feeding the MITCM solely with TSCs measured in neocortex, without inferring any pathway of solute displacement, nor any underlying mechanisms such as diffusion or transport. Yet, these findings corroborate the notion that under conditions with low neuronal activity (and therefore enlarged extracellular space) solutes permeate deeper into brain parenchyma, while under conditions with high neuronal activity, solutes do not leave paravascular spaces and are cleared from the brain faster.

## Discussion

Triggered by formulation of the glymphatic hypothesis by the Nedergaard group [4], research on solute or waste clearance from the brain has been intensified and advanced our understanding of this complex physiological process. While most mechanistic details, such as for example exact clearance pathways and mechanisms of fluid transposition or driving forces are still subject to intense debates, there seems to be consensus on a general picture [18, 19, 21]. Net transport, requiring efficient solute exchange, is most relevant for waste clearance, irrespective of the underlying pathways, mechanisms or driving forces [19]. Physiological parameters that have recently been discussed as pivotal for efficiency of waste clearance from the brain are CSF formation, volume of extracellular space and state of neuronal activity. While sometimes discussed separately [37, 38], it is well established that neuronal activity is tightly linked to extracellular volume [5, 48, 50], leaving CSF formation and neuronal activity as key factors. However, in animal experiments anesthesia, as well as awake or sleep conditions, influence these two parameters differently, hindering elucidation of their individual contribution. Therefore, we have selected experimental conditions that allow separating the individual effects of CSF formation and neuronal activity on efficiency of solute clearance from the brain. To assess this efficiency, we have devised a mechanism and pathway agnostic model that yields the net exchange ratio between an inner and an outer brain tissue compartment. We postulate that this parameter is suitable to estimate efficiency of solute clearance from the brain.

### Establishing experimental conditions that allow for disentangling the impact of CSF formation and neuronal activity

Sleep drives glymphatic clearance from the brain as shown in humans [54–57] and in animal studies [5, 58]. Enhanced solute clearance under various anesthetic regimens compared to the awake condition has also been reported [38]. The vast variety of anesthetics used in animal studies, their different modes of action, together with the fact that different classes of anesthetics differently or even opposedly affect different physiological processes, make comparison of studies difficult and add to the controversies about the glymphatic pathway and underlying mechanisms of solute clearance. In order to disentangle the impact of CSF formation and neuronal activity on the glymphatic clearance we established anesthetic regimens, resembling distinct conditions with high and low CSF formation and high and low brain activity, respectively. In humans, high CSF formation has been demonstrated during sleep, when parasympathetic activity is superior to sympathetic activity [59]. The administration of MED increases CSF formation due to an inhibitory effect on sympathetic neuronal activity [39–41], which stimulates CSF formation in plexus [1]. Under MED and ISO+MED, our experiments confirmed high CSF formation by measuring a larger volume of the basal cisterns and high aqueductal flow compared to the condition with ISO alone. Although our measurements could not exclude contributions from oscillatory CSF flow in aqueduct, our results are in accordance with previous flow measurements [59]. Enlargement of basal cisterns has also been reported by Ozturk et al. [60]. Para-arterial conduits, which are main CSF influx routes for glymphatic transport, were enlarged under MED in that study as well, and were explained by the blockade of norepinephrine in the preoptic area [36]. With ISO alone, a GABA_A_ chloride channel modulator, we achieved lower CSF formation together with low neuronal activity [29, 30]. Adding the carboanhydrase inhibitor AZE [42, 61, 62] reduced CSF formation under all three anesthetic regimens, MED, ISO-MED and ISO. Of the six experimental conditions used in our study, ISO+MED anesthesia induced a condition, which most likely resembled sleep, characterized by high CSF formation and low neuronal activity.

### The role of CSF formation

Several clinical studies support the importance of CSF formation and clearance [54, 63]. CSF formation is reduced in elderly humans [40], which might contribute to the higher beta-amyloid deposition in Alzheimer’s disease [39]. In patients with normal pressure hydrocephalus and expected CSF circulation failure, low Gadobutrol clearance has been observed and has been interpreted as reduced solute clearance [41]. Vice versa, utilizing the CSF formation-enhancing effect of Dexmedetomidine for improving intrathecal drug distribution has been experimentally shown [64].

In our study, CSF formation under conditions with MED obviously promoted CA distribution in the brain. TSCs clearly indicated that most regions were reached by CA earlier and achieved higher signal under conditions with high CSF formation (MED and ISO+MED) in comparison to conditions with low CSF formation (ISO or any condition containing AZE). More effective CSF distribution under MED is in line with previous findings of enhanced glymphatic activity under Dexmedetomidine-ISO compared to ISO [36, 38]. Neuromodulatory signaling or better the lack thereof has been identified as a potential driver of glymphatic clearance based on studies in mice that experimentally decreased norepinephrine (NE) release by application of α2-antagonists to the cisterna magna or directly to the cortical surface [5] or by systemic anesthesia comprising α2-agonists Xylazine [5, 38] or Dexmedetomidine (the pharmacologically active racemate of medetomidine) [36].

Adverse systemic effects of the drugs used in the current study, for example impact on the breathing rate, need to be considered. The documented respiration rates in spontaneously breathing rats (Additional file 1) suggests highest end-expiratory CO_2_ values under ISO-MED, followed by MED, and then ISO. However, no effect of potential hypercapnia (in particular under ISO-MED) on CSF formation was observed. Subarachnoidal space and aqueductal flow were highest under MED and ISO-MED, followed by ISO. Lowering of heart rate, is another known adverse effect of MED, which could in turn lower CSF pulsatillity. Indeed, lower heart rates under conditions with MED were observed. However, CSF formation was still higher compared to conditions without MED. AZE reduced CSF formation in the current study, as expected [58]. A recent paper [42] provided experimental evidence that AZE exerts direct action on the choroid plexus and not indirectly via the systemic action of AZE on renal and vascular processes, irrespective of the mode of drug administration and level of anesthesia. However, we cannot fully exclude AZE side effects that potentially, secondarily impacted fluid dynamics. Inhibition of brain and blood carbonic anhydrase increases cerebral blood flow by acidifying cerebral extracellular fluid [65]. We observed increased vessel diameters after application of AZE, and an increase in cerebral blood flow and arterial vessel diameter have been reported in dogs [53]. We also observed higher ADC values in all three anesthetic conditions when adding AZE, potentially indicating dampening of neuronal activity. Elevated neurotransmitter release as a consequence of pH changes have been linked to the long-known anticonvulsant properties of AZE [66]. Indeed, by administration of CO_2_ in one of our previous studies [30] we also found reduced excitability of pyramidal neurons and decreased firing of neurons in vivo and in slice experiments. In the current study, though, we did not see compensatory hyperventilation (as suspected by Dahan et al. [67]) in spontaneously breathing animals (Additional file 1).

We therefore attribute differences in CSF distribution under these conditions to the different level of CSF formation. Our conclusion is supported by Hornkjol et al. [63] who interrogated the role of CSF flow in the ventricular system and subarachnoidal space. Their simulations based on human DCE data identified CSF flow in the subarachnoidal space as a major player in solute clearance. In line with our observations under conditions with high CSF formation, enhanced turnover of CSF limited the influx and facilitated clearance also within brain parenchyma.

### The role of neuronal activity

Already Xie et al. [5] demonstrated via real-time iontophoresis in mice that during sleep and Ketamin-Xylazine anesthesia interstitial space volume was increased and accompanied by striking increase of tracer distribution into brain parenchyma, resulting in a more efficient removal of β-Amyloid plaques. An increase in interstitial volume fraction was also reported in that study upon direct application of receptor antagonists to the exposed cortical surface of mice. The authors concluded that reduction of NE signaling has a major impact on solute transport. We have observed faster washout kinetics and slower parenchymal diffusion under MED compared to ISO-MED, both conditions supposed to systemically reduce NE signaling (lower heart rates were in fact apparent in all conditions with MED) but with different neuronal activity, providing evidence that neuronal activity itself may influence solute transport in addition to NE signaling. Hablitz et al. [38] associated low neuronal activity with high glymphatic efficiency in mice under anesthesia, and others have observed a pronounced effect of anesthetics on solute transport [36, 37]. In humans it has been shown that ADC increases during sleep in the cerebellum and left temporal lobe [49]. In mice, Gakuba et al. [37] did not observe differences in ADC in awake mice compared to mice under ISO anesthesia. The seemingly conflicting results most likely depend on the specific experimental design. ADC measurements were repeatedly performed under alternating anesthetic conditions, without allowing more than 30 min between measurements to adapt to the new condition [68].

In our study, Ca^2+^-recordings during diffusion-weighted MRI experiments under the different anesthetic conditions confirmed that low neuronal activity goes along with high diffusivity in neocortex. This is in line with the notion that diffusivity in the brain is mostly affected by cell swelling due to neuronal activity and correspondingly altered size of the interstitial space [48, 50]. Early optical studies in brain slices [69] already demonstrated activity-dependent increases of light transmittance reflecting neuronal and astrocytic swelling, and suggested the technique to be most useful in monitoring excessive neuronal discharge during epileptiform and excitotoxic states. More recently, Pan et al. [70] demonstrated with intrinsic optical signal recordings in isoflurane-anesthetized rats altered scattering and absorption in response to the up-states of spontaneous neuronal activity in cortical or subcortical structures, with a strong correlation to local field potentials. The authors related their observations to the same source of changes as observed with diffusion-weighted fMRI (dw-fMRI) [71] and line scanning diffusion MRI [72], namely cell volume changes and extracellular space shrinkage. This possibly explains increased permeation due to lower mechanical hindrance in the interstitial space during reduced neuronal activity [1, 5, 48, 49]. In our experiments, MED that induced high neuronal activity and low diffusivity, resulted in faster signal decay in TSCs, indicative of a faster clearance of the tracer as compared to conditions with higher diffusivity. This accelerated clearance is in line with the notion that CA remained confined to paravascular spaces of the brain and presumably exited the subarachnoid space by transport through the lymphatic system to the systemic circulation [73]. In contrast, under ISO+MED which induced lower neuronal activity and higher diffusion, TSCs showed slower signal decay. This is in line with a better permeation of the interstitial space, facilitating increased efficiency of solute removal from the brain parenchyma.

### A pathway and mechanism independent two-compartment model (MITCM) to estimate glymphatic efficiency

Modelling solute clearance from the brain suffers from poorly defined boundary conditions, such as velocities of water in the interstitial and paravascular space and microstructural details that define barrier functions and driving forces [21]. This problem is underlined by a recent sensitivity analysis on a network model of glymphatic flow [74]. That analysis pointed out how largely unknown or naturally varying input parameters impact model performance and limit relevance of the obtained conclusions for practical applications. MRI has an intrinsically low resolution, when compared to intra-vital fluorescence microscopy, and therefore cannot provide the missing links. Our model makes the strongly simplifying assumption that a brain region under consideration can be represented by two layer-shaped compartments, an outer compartment and an inner compartment. We have applied these assumptions only to neocortex, which is of considerable volume and where compartments are homogeneous with respect to the orthogonal arrangement of vessels. The MITCM further assumes that efficiency of solute clearance is defined by the amount of fluid passing through the inner compartment. Sole input parameter is the measured TSC. Output parameter is the net exchange ratio between the outer and inner compartment. For neocortex, for low neuronal activity (MED+ISO) we found lower net exchange ratio, than for the condition of high neuronal activity (MED).

Our results suggest that in a state of high neuronal activity (MED) CSF remains in the paravascular space and is washed out fast, while in a state of low neuronal activity (MED+ISO) more CSF reaches the interstitial space. Based on quantifying the efficiency of net solute transport, we can differentiate the sleep-resembling condition, with reduced neuronal activity and enhanced CSF formation from awake-like condition with high neuronal activity.

It must be noted that there remain limitations to our study. First, we did not detect fluid motion directly, but observed distribution of a low molecular weight MRI contrast agent as tracer substance. Yet, low molecular contrast agents are widely accepted as surrogate marker to study solute clearance [36, 37, 75–77], although not representing clearance of large molecular complexes [12, 23]. Further, ADC in aqueduct and parenchyma only indicates the magnitude but not the directionality of diffusion or flow. Second, the MITCM makes the strongly simplifying assumption of two exchanging compartments that, as introduced here, do not have a direct anatomical equivalent. Third, we focus on net transport rather than considering specific mechanisms (diffusion, flow or advection) or pathways (para or peri venous or arterial) or including input parameters for potential driving forces (pressure or osmotic gradients, pulsatile motion). Albeit the simplicity of our approach, our conclusions support the notion that solute clearance from the brain is more efficient during sleep [54] or anesthesia that reduces neuronal activity but does not reduce CSF formation [5, 36, 38]. This is not disregarding the value of more sophisticated models. Their continuous improvement with new information and their validation with artificial and experimental data will help to identify incorrect or insufficiently precise assumptions and to better understand the complexity of solute clearance. Kinetic models relying solely on measured transport of tracer solution as a surrogate for CSF-ISF flow and exchange have been used to study solute clearance in animal disease models. Mortensen et al. [78] applied a one-tissue compartment model to brain and CSF time-concentration curves and found reduced global solute influx and efflux rates in hypertensive rats using quantitative DCE-MRI data acquired during and after the infusion of gadoteric acid (Gd-DOTA) into the cisterna magna. Davoodi-Bojd et al. [46] proposed a two-compartment-model with a local input function to quantify DCE data from a diabetes rat model. The estimated parameters indicated reduced solute clearance in diabetic rats.

## Conclusion

The precise pathways and mechanisms of solute clearance from the brain remain elusive. Here, we have isolated two of the contributing factors, CSF formation and state of neuronal activity. Establishing conditions with distinct levels of neuronal activity and CSF formation enabled connecting widespread tracer distribution to states with high CSF formation and limited tracer permeation to states with a high level of neuronal activity. A simple kinetic model that is agnostic for clearance pathways and driving forces can provide an estimate of the efficiency of solute clearance by calculating a net solute transport, solely based on the analysis of the time signal curves obtained from DCE MRI. This rather simplifying approach yielded results that were in accordance with previous preclinical and clinical findings. The model may be applied to any measured tracer kinetics and provide an estimate of efficiency of solute clearance from the brain.

## Supplementary Information

**Additionalfile 1.** Respiratory rate.

**Additionalfile 2.** Heart rate.

**Additionalfile 3.** Brain region specific analysis.

**Additionalfile 4.** Table of group comparison between the different anesthetic conditions for every brain region and for the four parameters (time to start; time to maximum; maximal signal; area under the curve) using Kruskal–Wallis test.

**Additionalfile 5.** Signal decay rate of TSCs under MED and ISO+MED.

**Additionalfile 6.** Solution of the differential equation.

## Data Availability

The datasets used and/or analyzed during the current study are available from the corresponding author on reasonable request.

## Abbreviations

ADC: Apparent diffusion coefficient
AUC: Area under the curve
AZE: Acetazolamide
BL: Baseline
CA: Contrast agent
CSF: Cerebrospinal fluid
DCE: Dynamic contrast-enhanced
DTI: Diffusion tensor imaging
DWI: Diffusion weighted imaging
ISF: Interstitial fluid
ISO: Isoflurane
MED: Medetomidine
MITCM: Pathway and mechanism-independent two-compartment model
MRI: Magnetic resonance imaging
TOF: Time of flight
TSC: Time signal curve
