# Supplementary material for: Disentangling the impact of cerebrospinal fluid formation and neuronal activity on solute clearance from the brain
Source: Fluids Barriers CNS. 2023 Jun 14;20:43. doi: 10.1186/s12987-023-00443-2 (PMC10265831; doi:10.1186/s12987-023-00443-2)
Supplement: Supplementary file 1 — Additionalfile 1. Respiratory rate. [file 12987_2023_443_MOESM1_ESM.docx]

Additional file 1 – Respiratory Rate


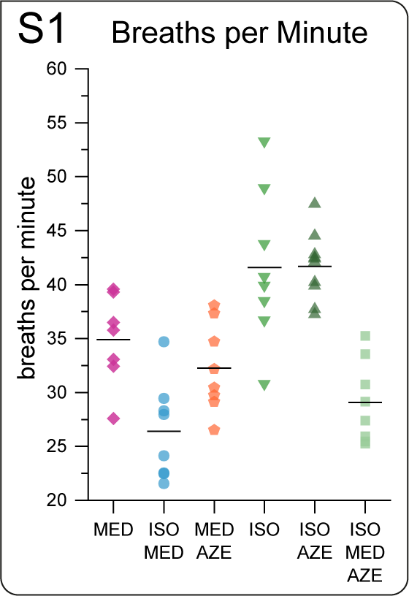


Respiratory rate displayed as breaths per minute for every anesthetic condition as mean over the whole experiment for each animal. The gray line gives the mean over all animals in the respective anesthetic condition. (ISO n = 8; MED n = 7; ISO+MED n = 8; ISO+AZE n = 10; MED+AZE n = 8; ISO+MED+AZE n = 8).
