# Supplementary material for: Disentangling the impact of cerebrospinal fluid formation and neuronal activity on solute clearance from the brain
Source: Fluids Barriers CNS. 2023 Jun 14;20:43. doi: 10.1186/s12987-023-00443-2 (PMC10265831; doi:10.1186/s12987-023-00443-2)
Supplement: Supplementary file 2 — Additionalfile 2. Heart rate. [file 12987_2023_443_MOESM2_ESM.docx]

Additional file 2 – Heart Rate


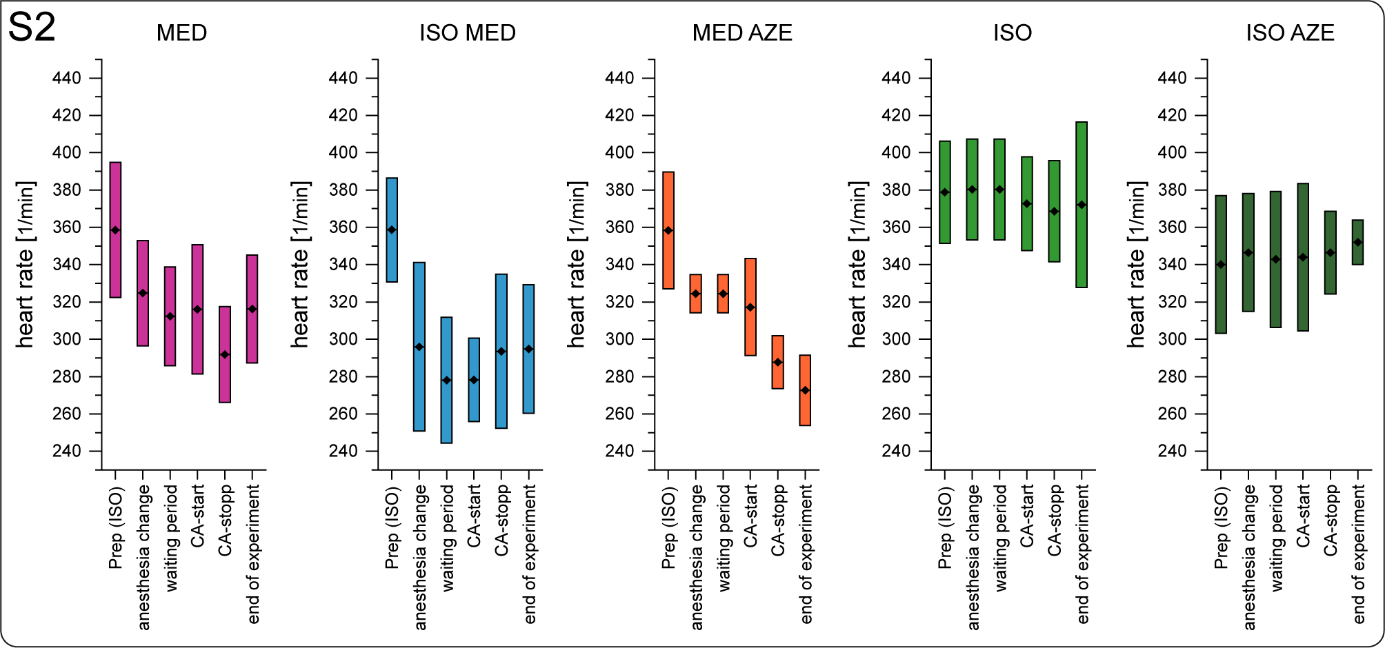


Exemplary heart rate values displayed as mean with boxes indicating standard deviation for the anesthetic conditions (MED, ISO+MED, MED+AZE, ISO, ISO+AZE) at specific timepoints during the experiment (Preparation (Prep) under ISO; anesthesia change; waiting period; CA-start; CA-stop; end of experiment). (ISO n = 3; MED n = 10; ISO+MED n = 7; ISO+AZE n = 7; MED+AZE n = 4)
