# Supplementary material for: Disentangling the impact of cerebrospinal fluid formation and neuronal activity on solute clearance from the brain
Source: Fluids Barriers CNS. 2023 Jun 14;20:43. doi: 10.1186/s12987-023-00443-2 (PMC10265831; doi:10.1186/s12987-023-00443-2)

Additional file 3 – Brain region specific analysis

Illustrations of time signal curves, and boxplots for time to start, time to maximum, maximal signal and area under the curve for every anesthetic condition and every of 79 analyzed brain regions. The specific brain region is the respective title of the illustration and is illustrated in the 3D rendering. Time signal curves are represented in percent signal change from baseline and time in minutes for each anesthetic condition and each animal (thin curves) and mean (thick curves) (ISO n = 8; MED n = 7; ISO+MED n = 8; ISO+AZE n = 10; MED+AZE n = 8; ISO+MED+AZE n = 8). Concerning the Boxplots the central mark indicates the median, the bottom and top edges of the box indicate the 25th and 75th percentiles, respectively. The whiskers extend to the most extreme data points not considered outliers. Significant differences are marked with one star if p < 0.05, two stars if p < 0.01 and three if p < 0.001.

Gray matter
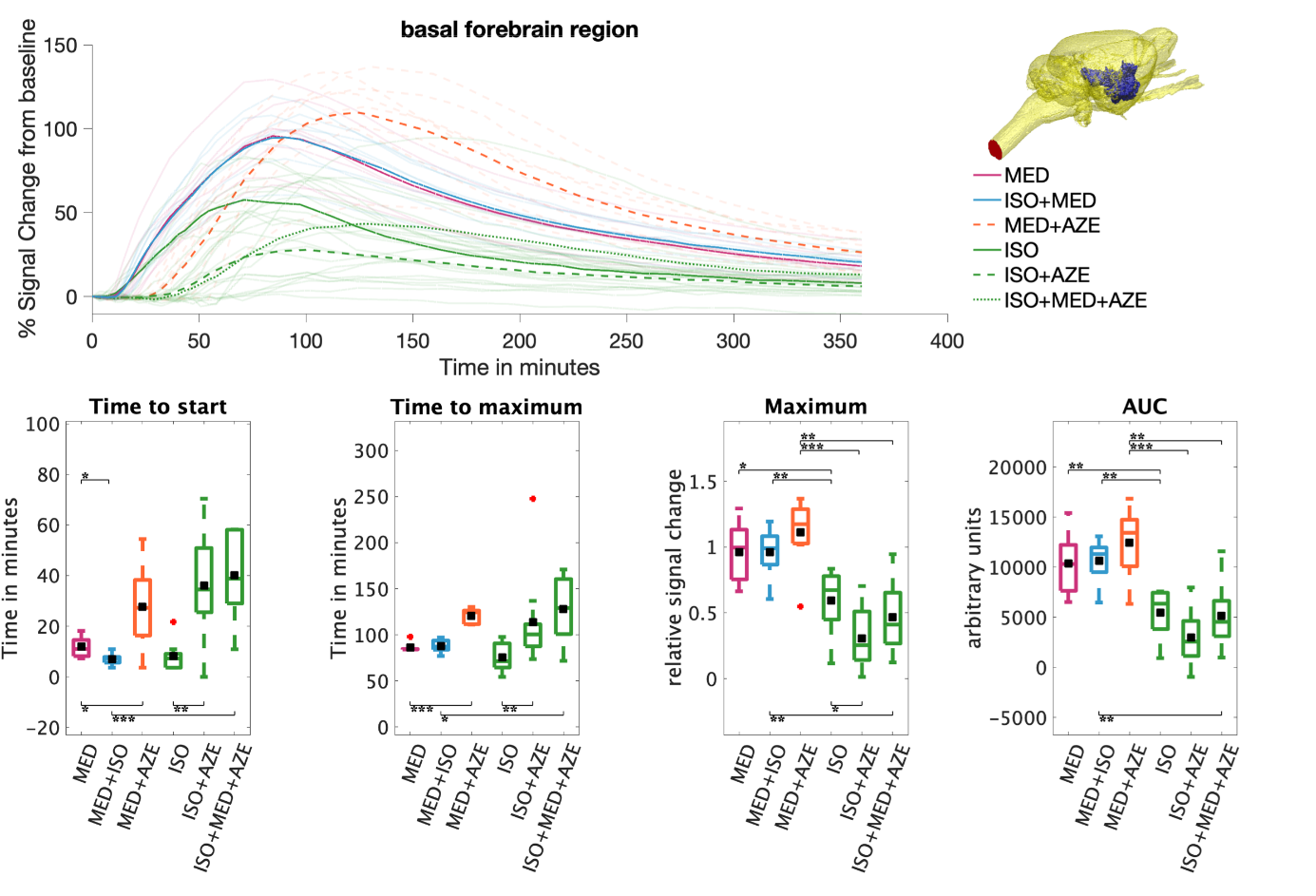

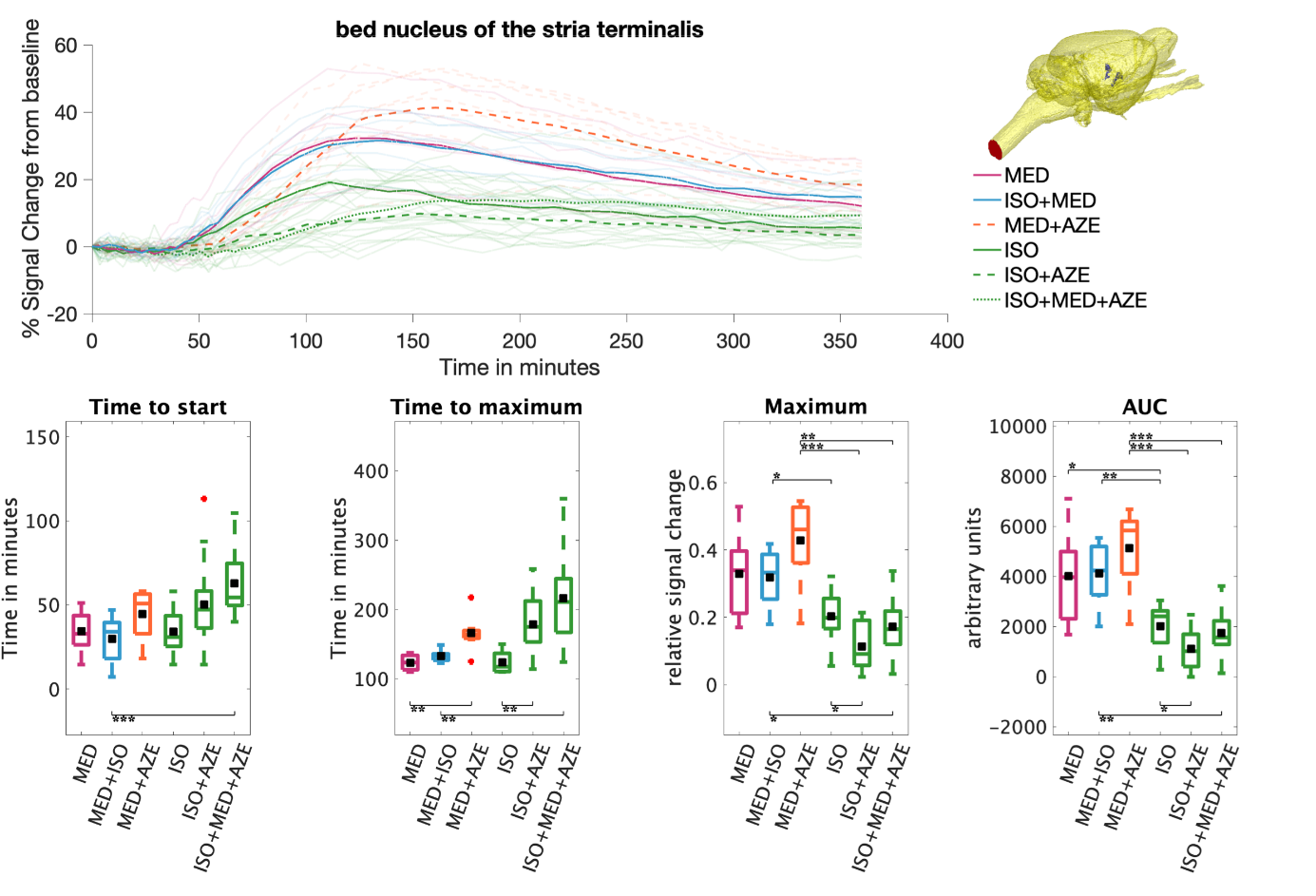

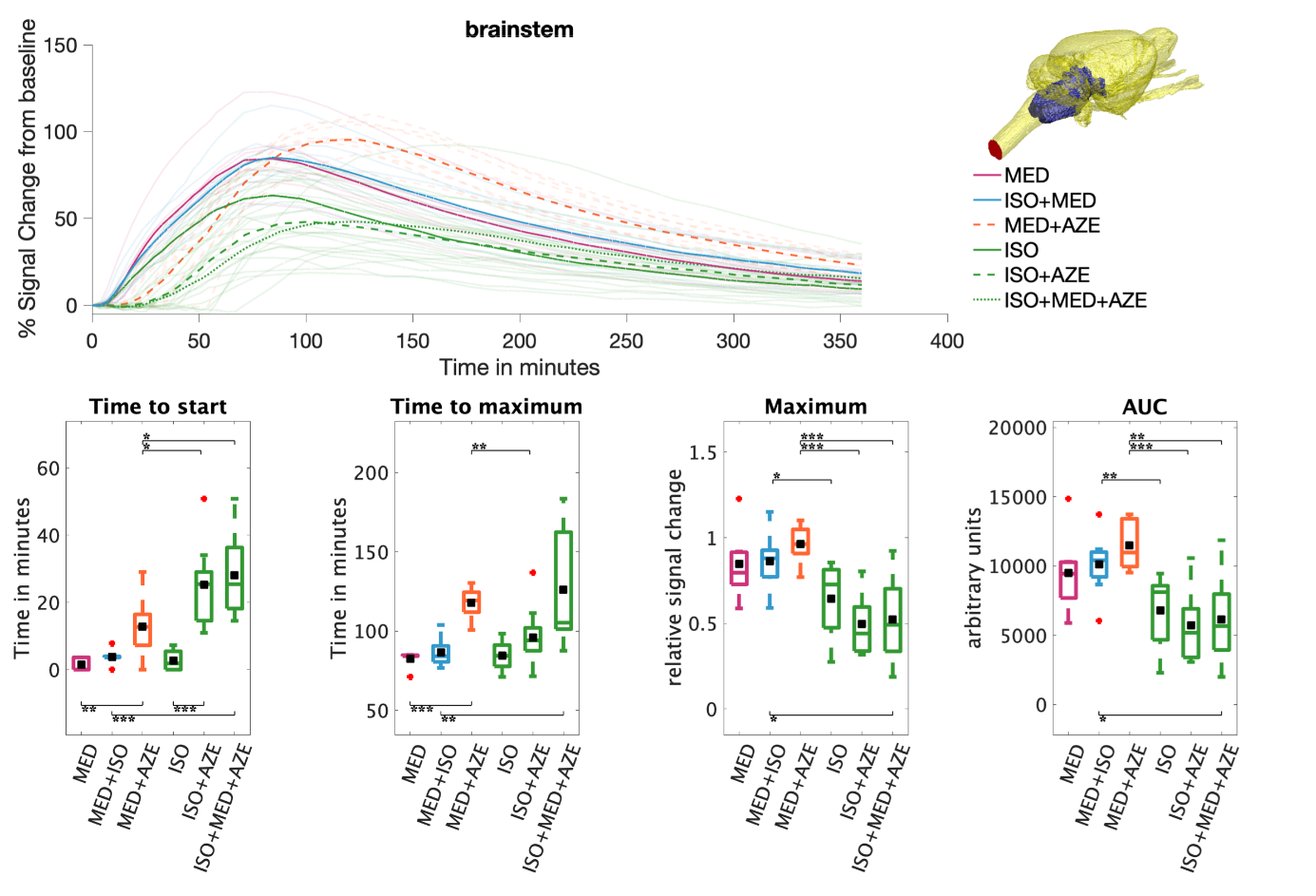

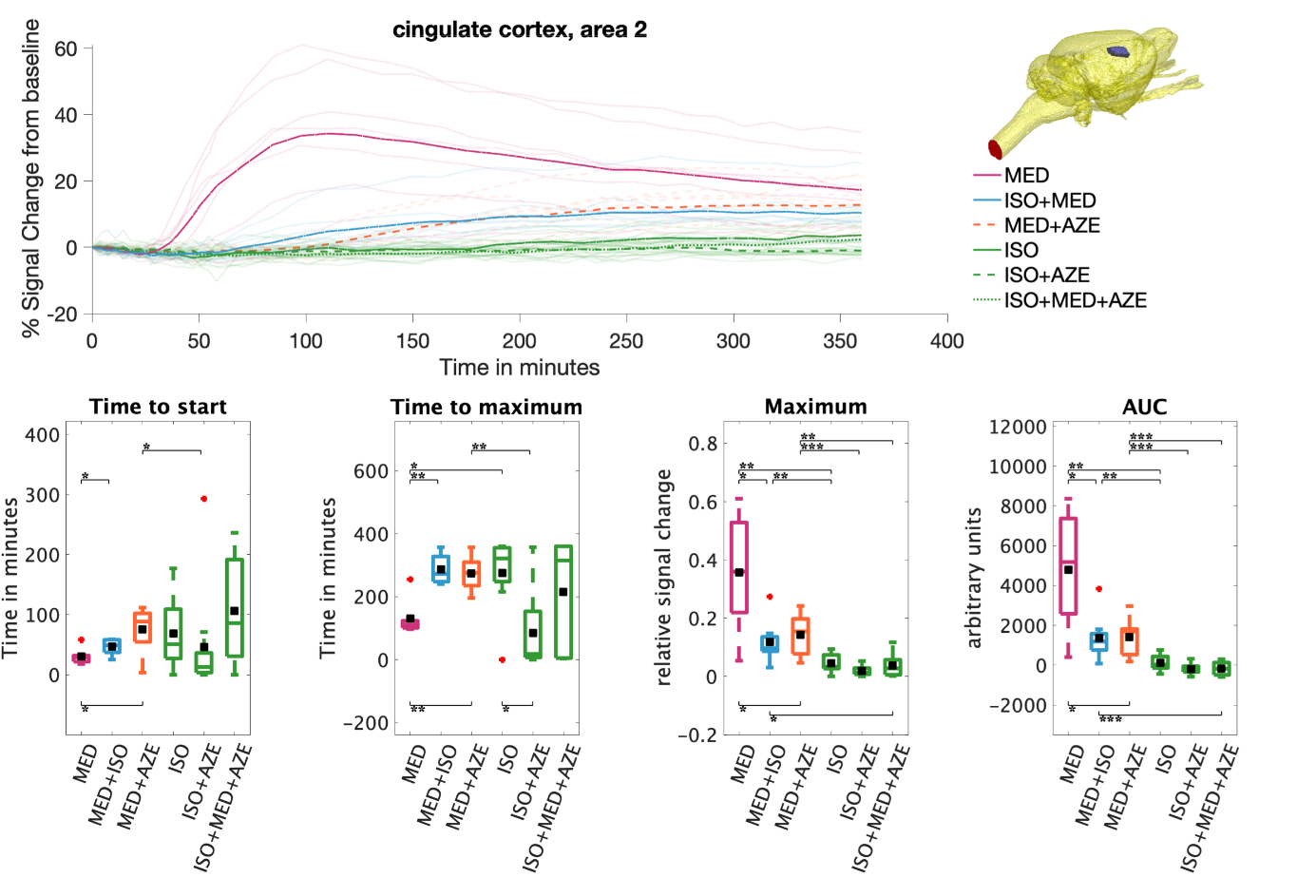

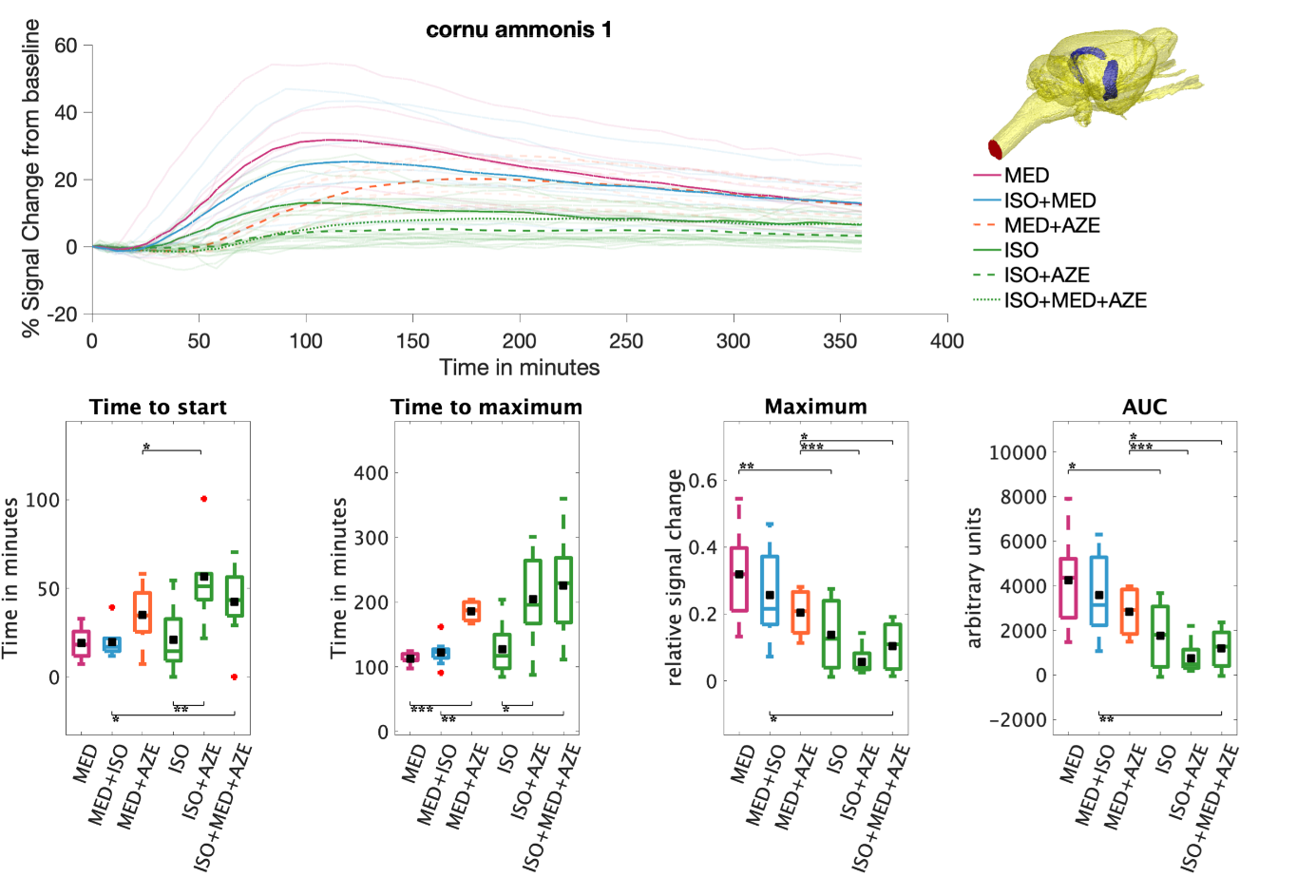

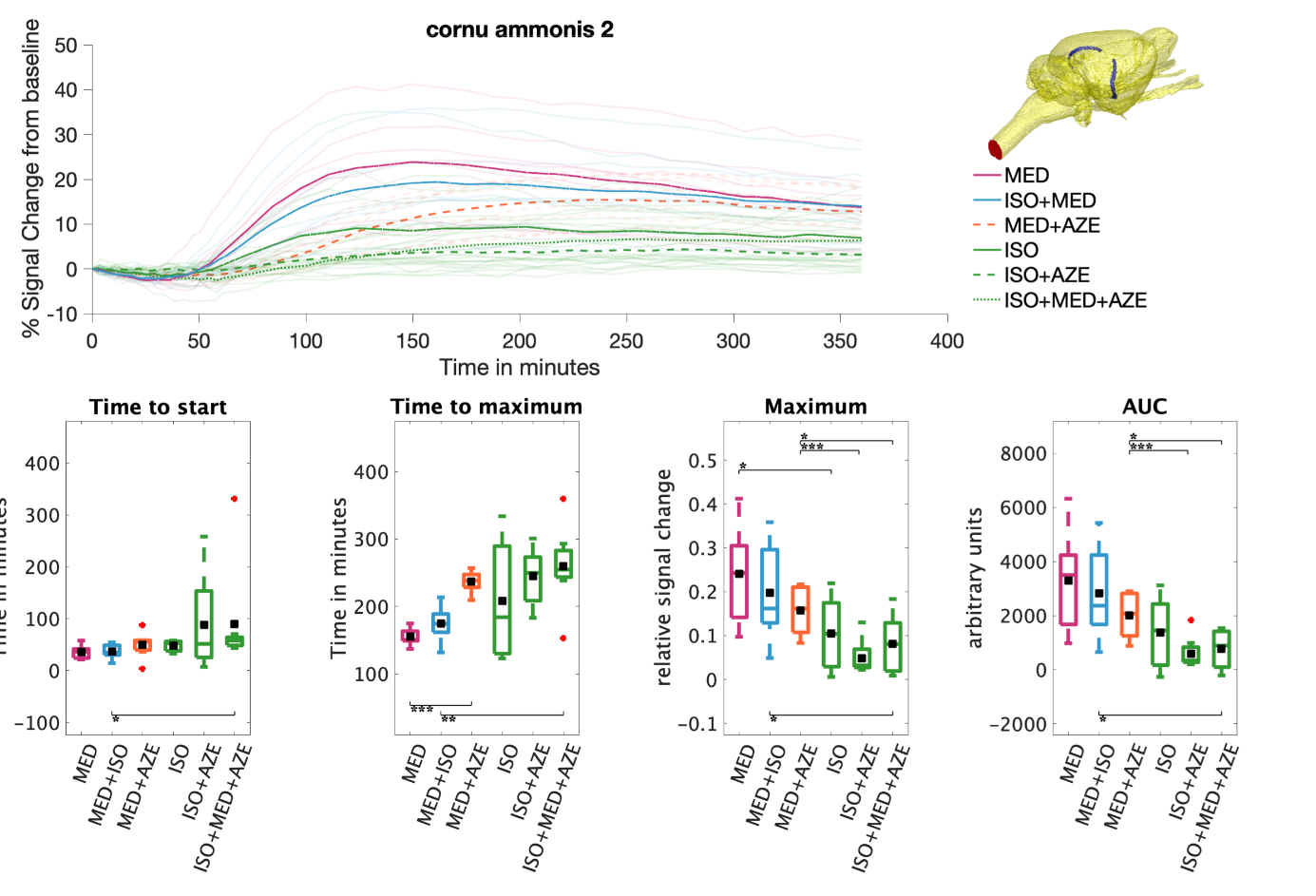

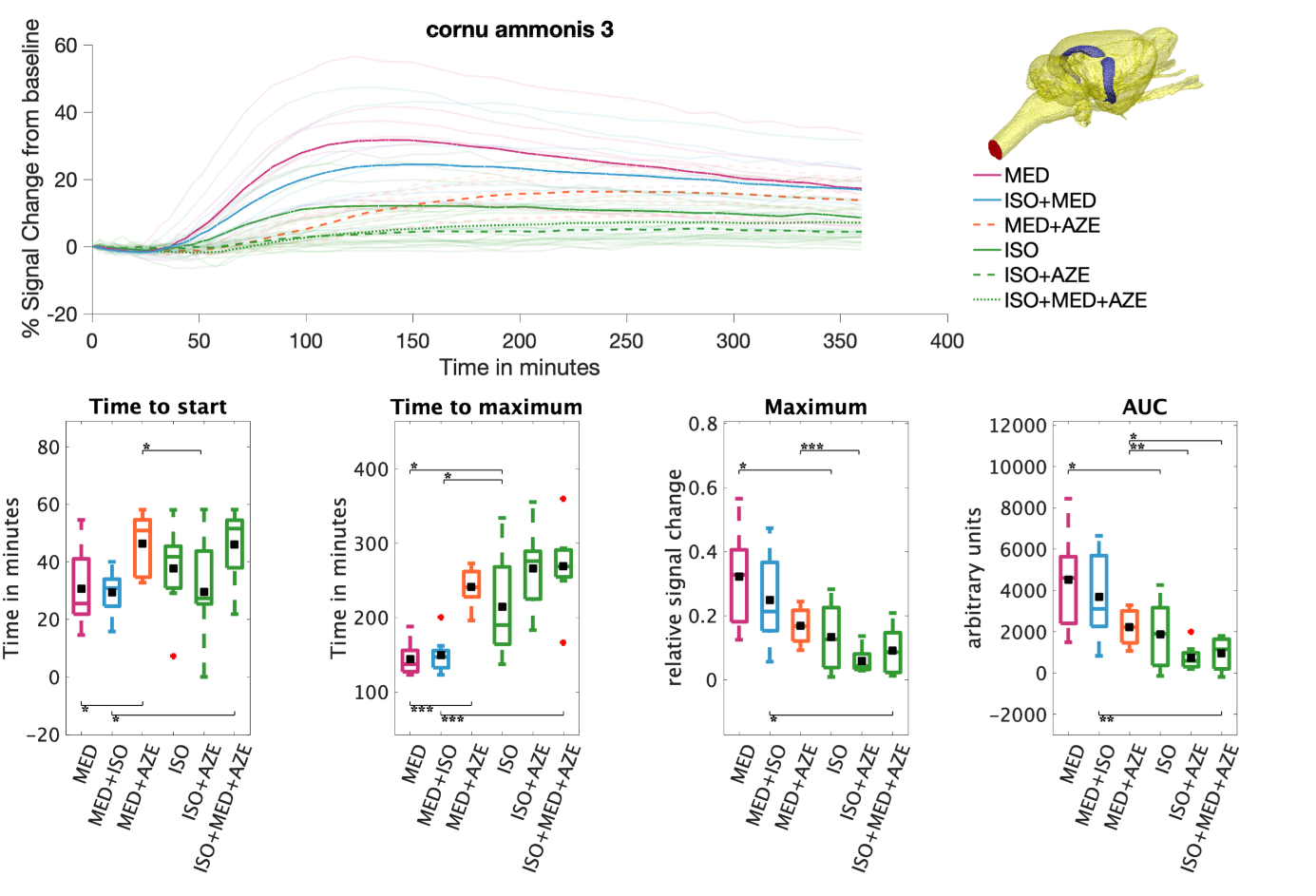

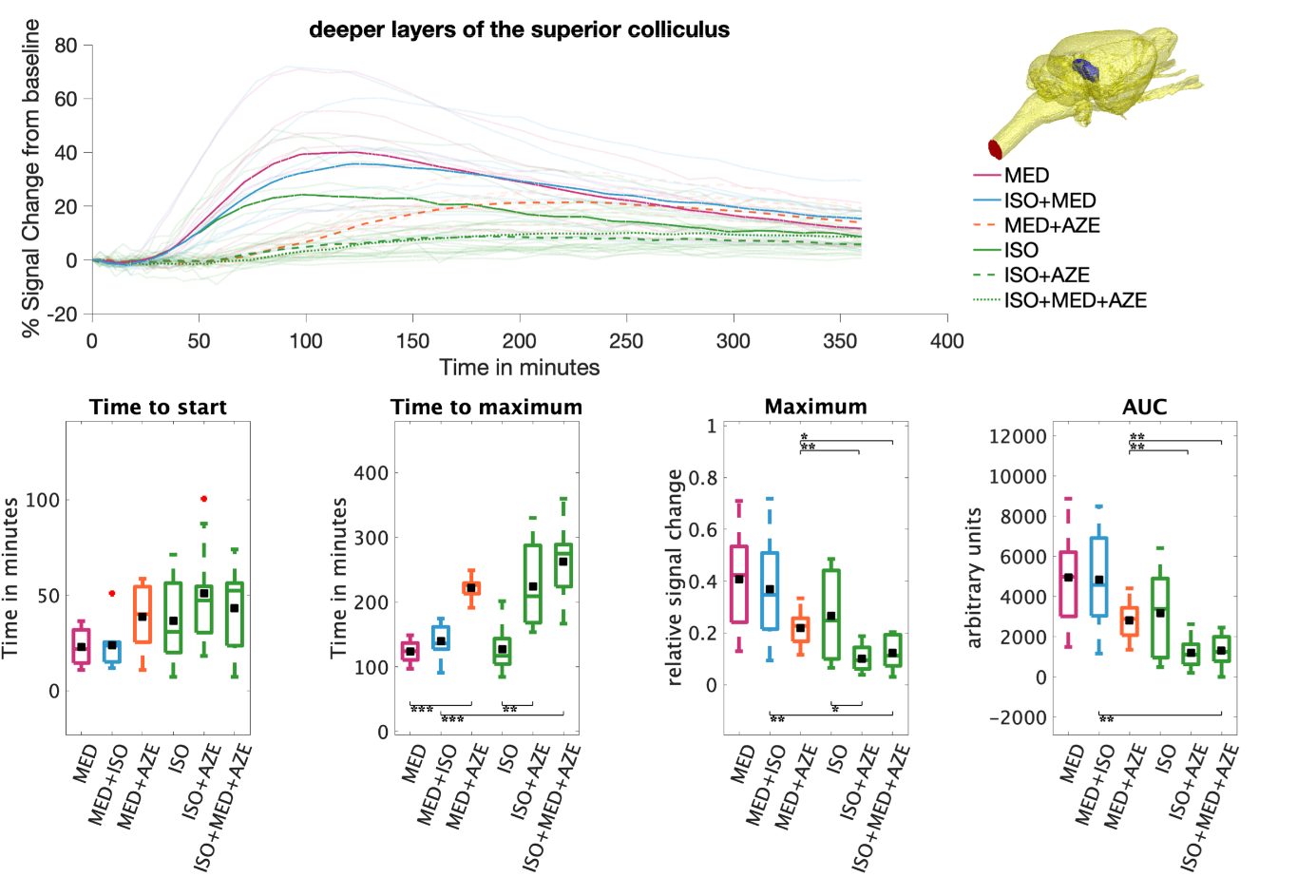

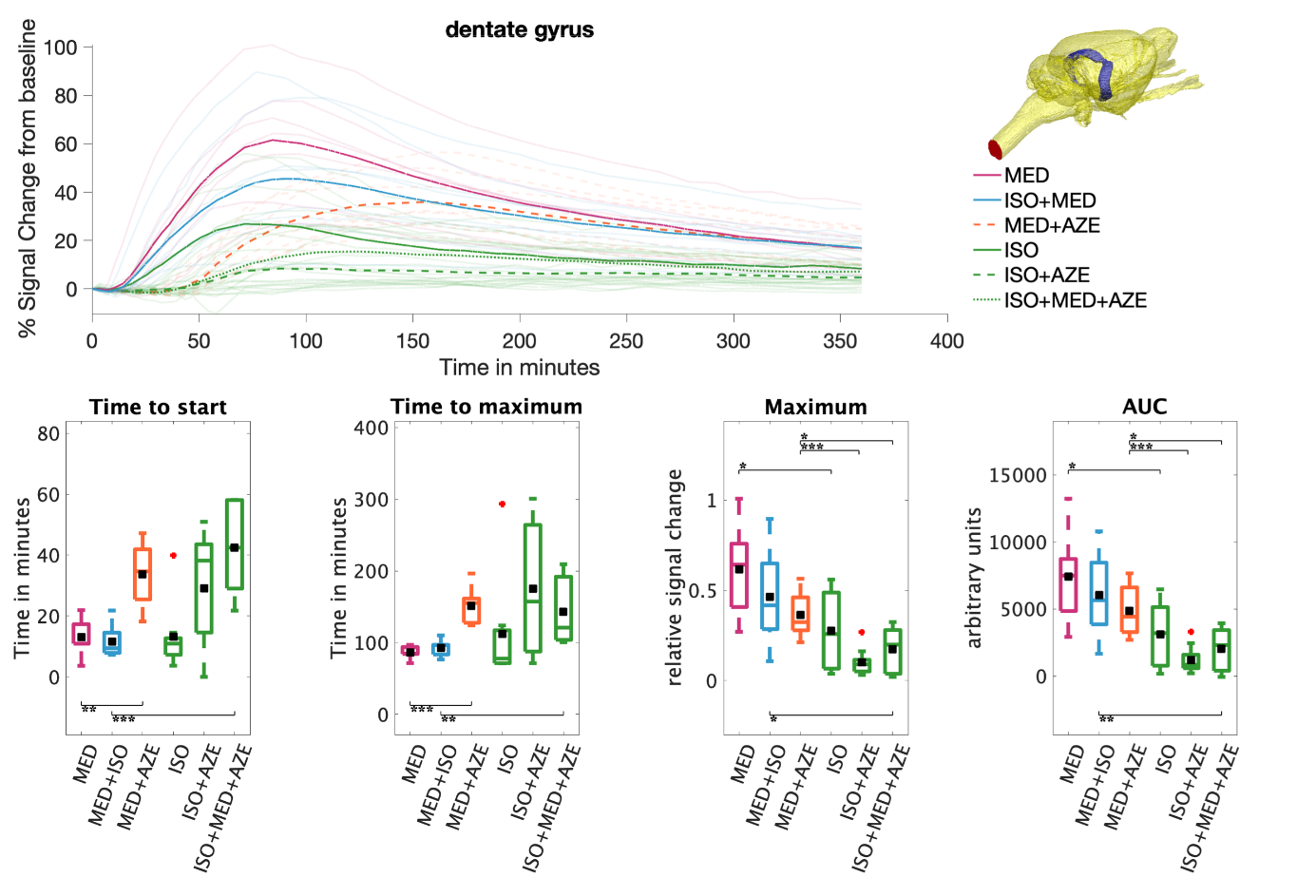

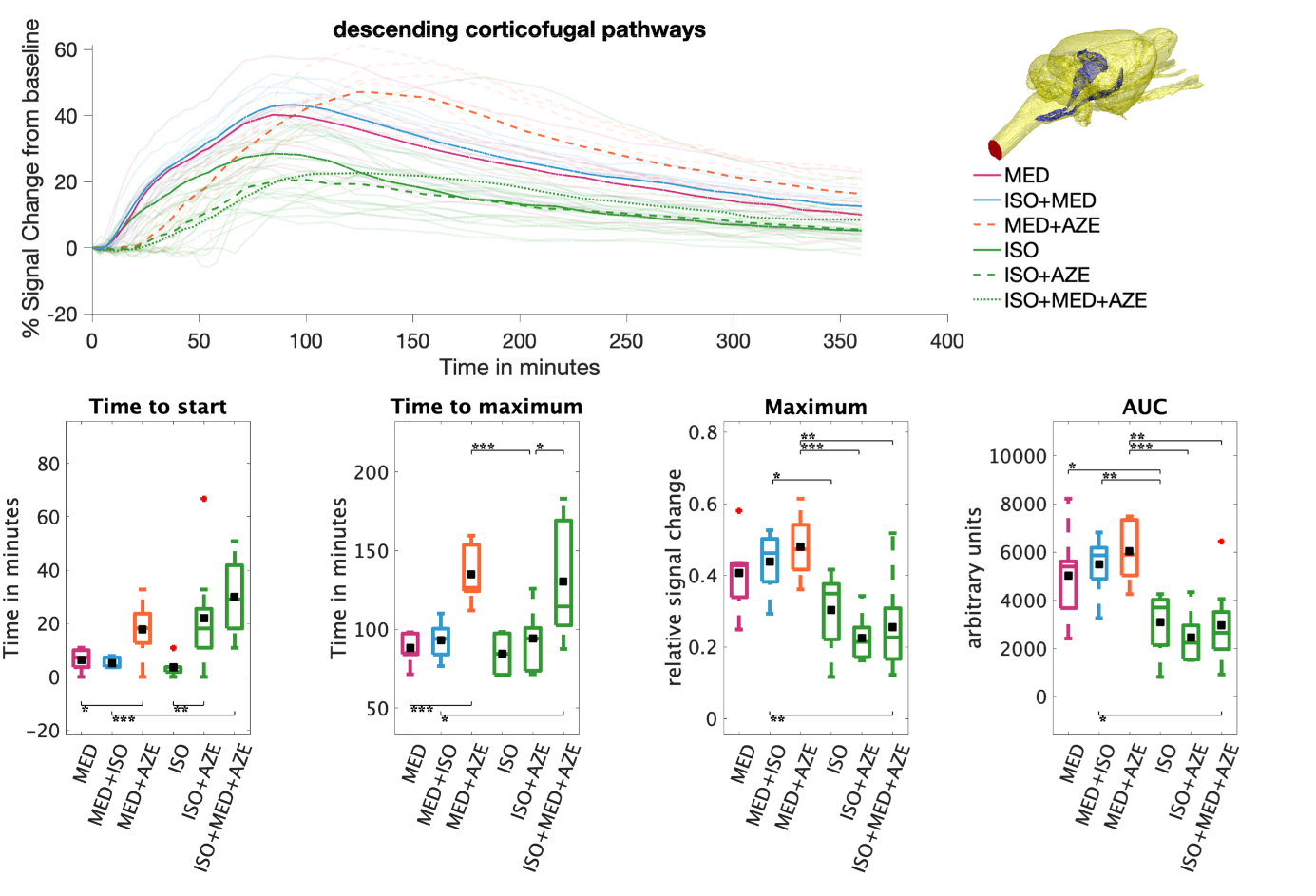

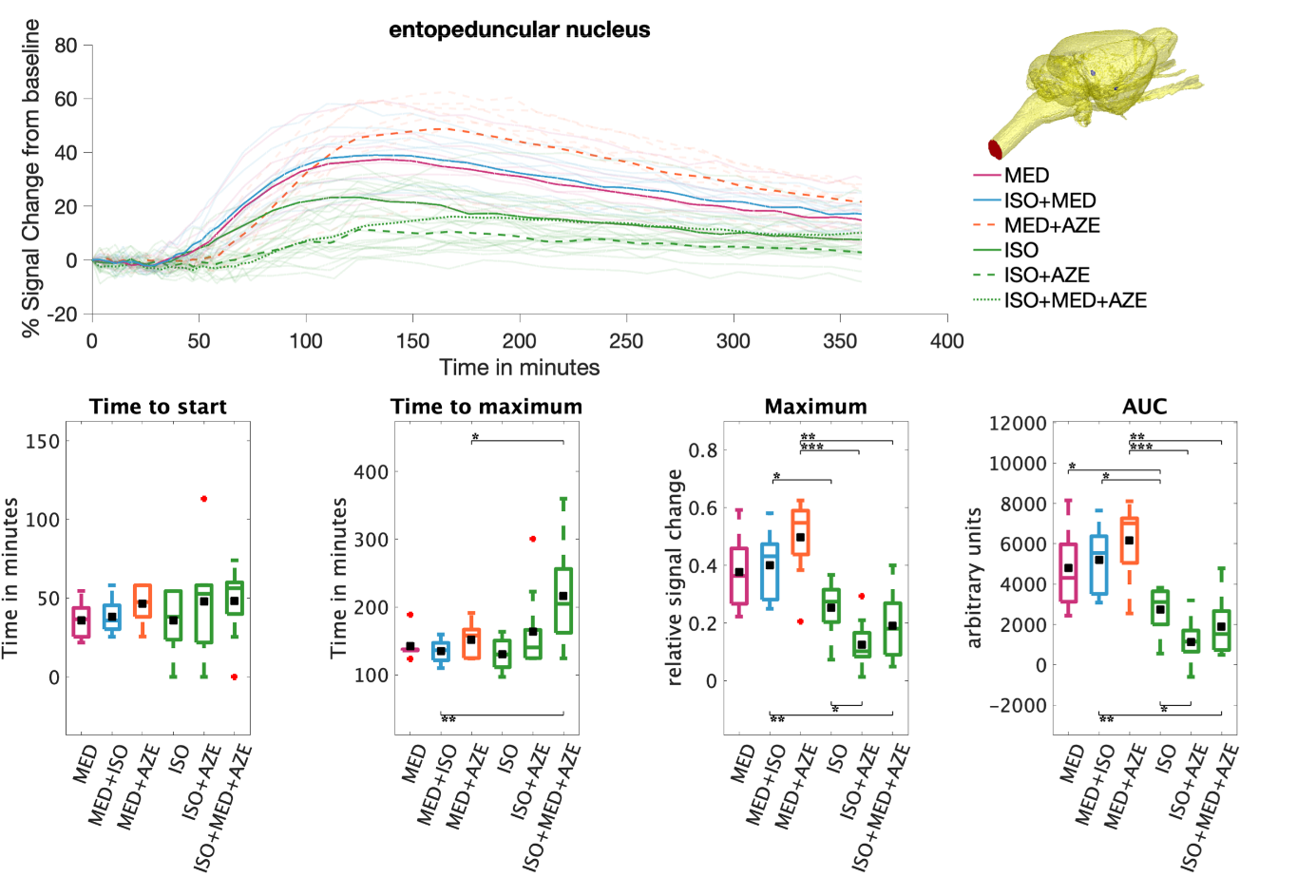

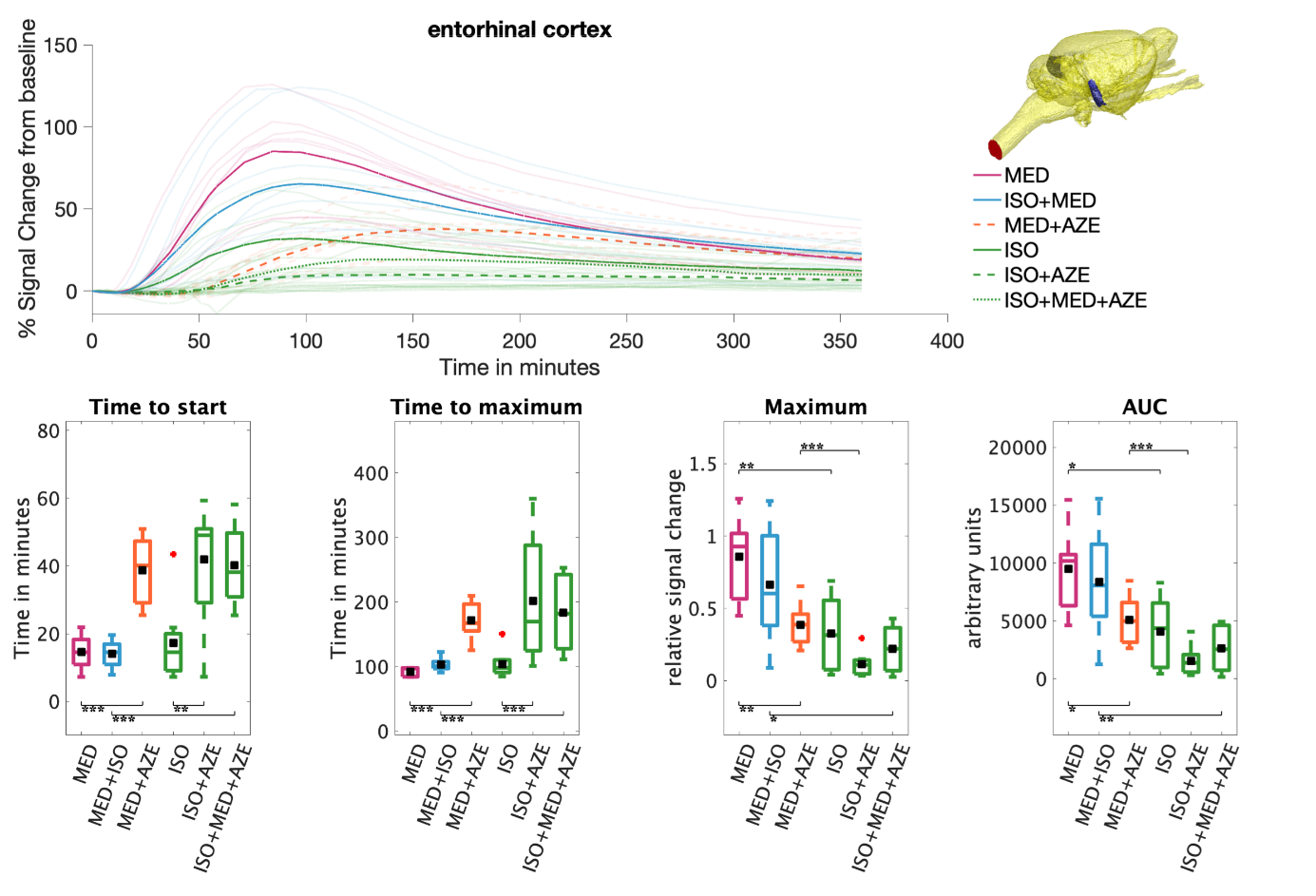

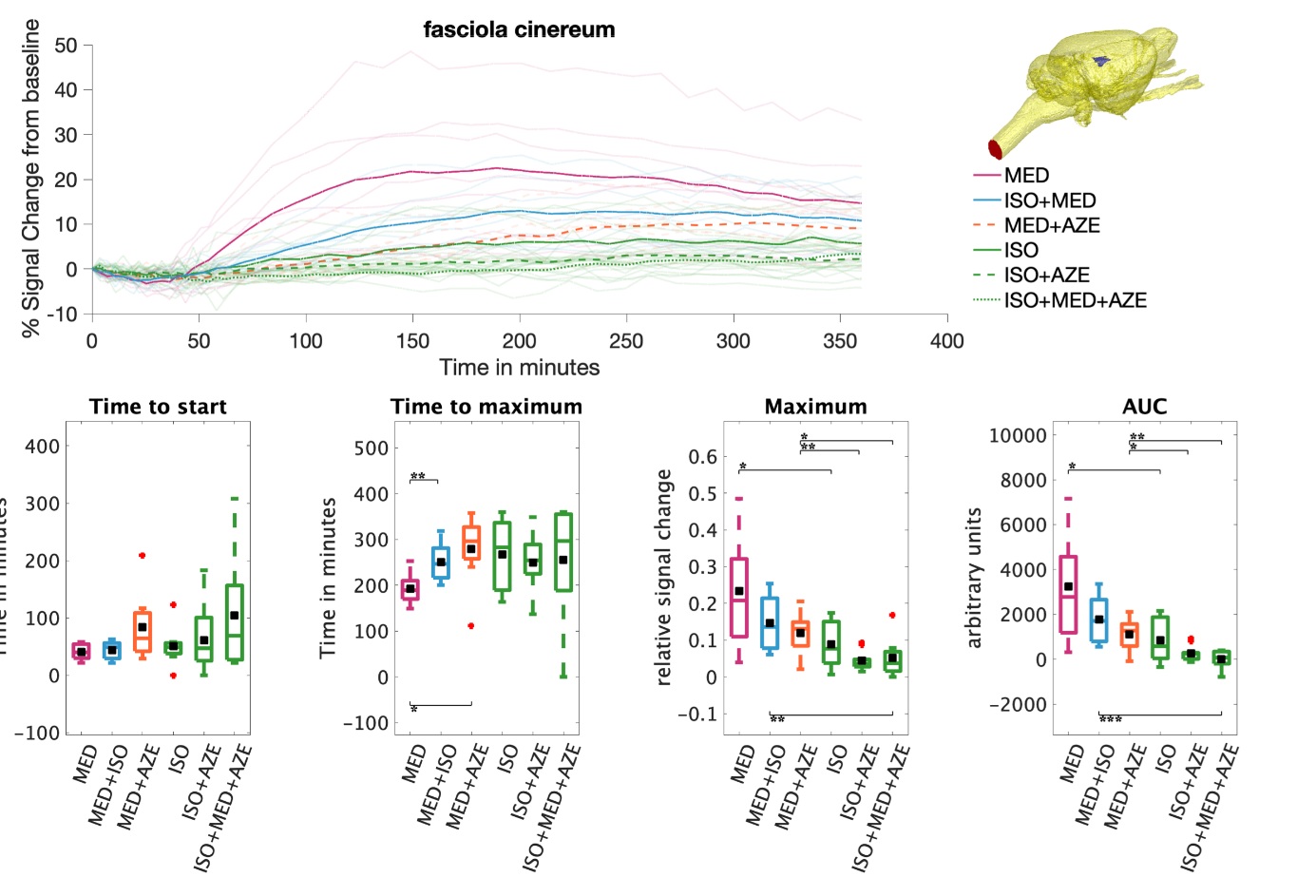

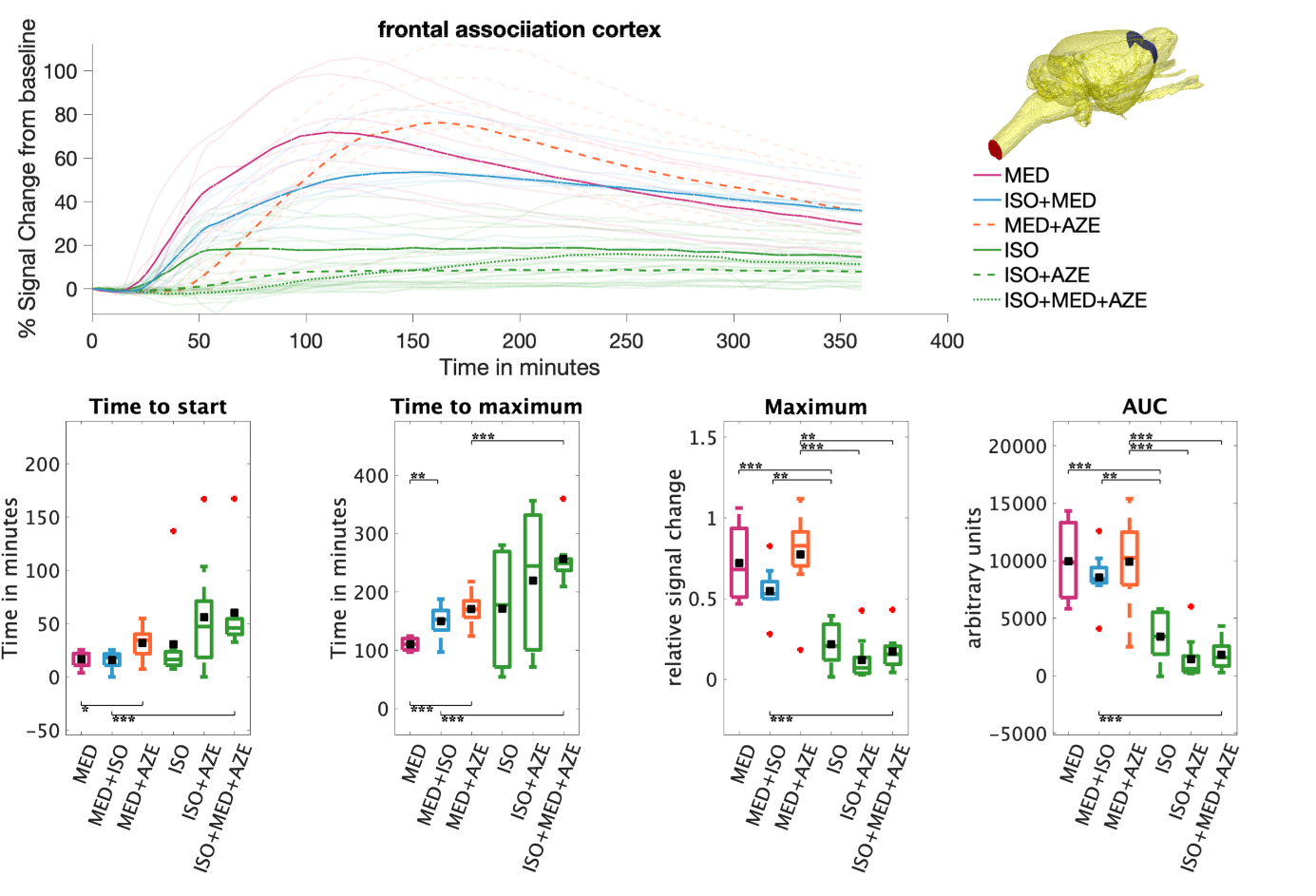

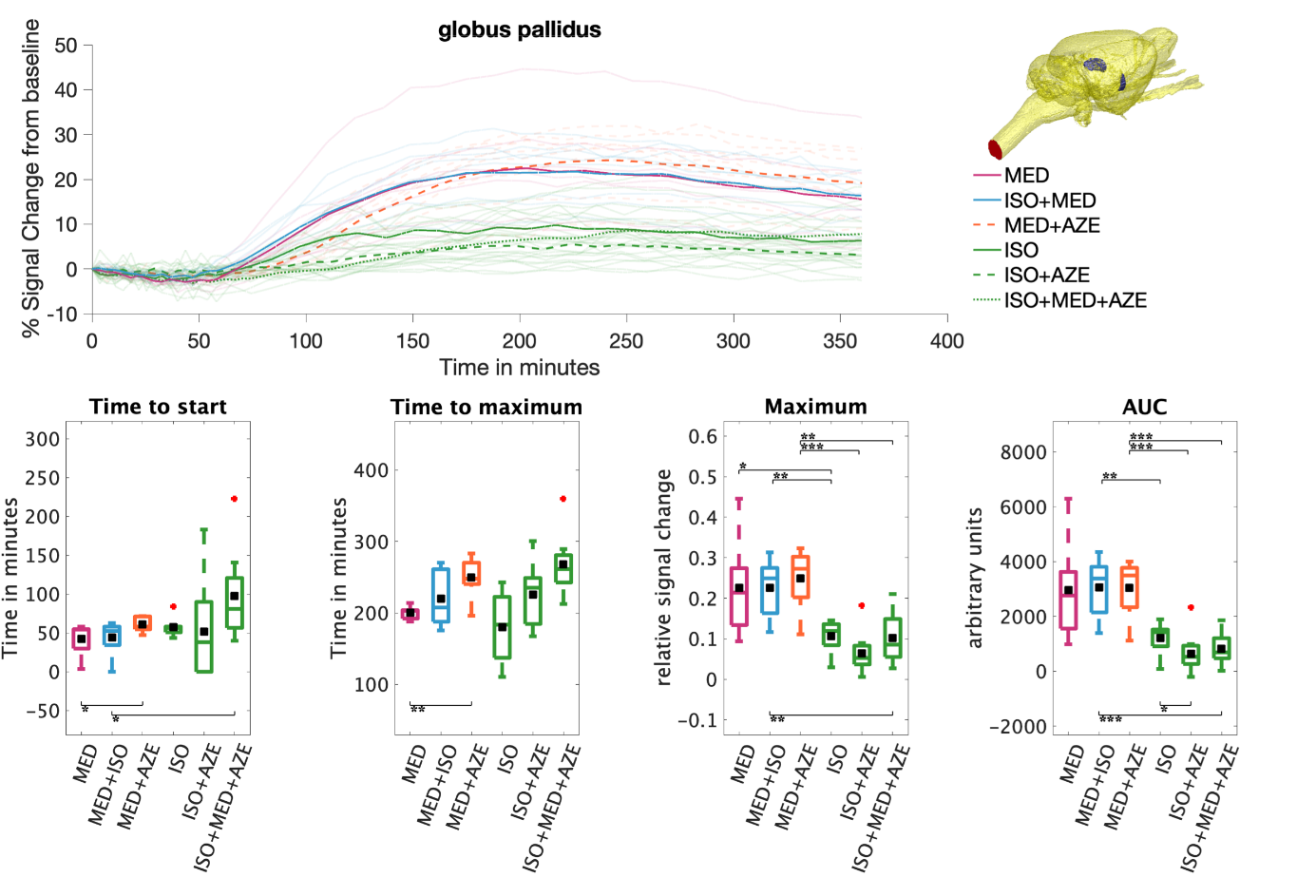

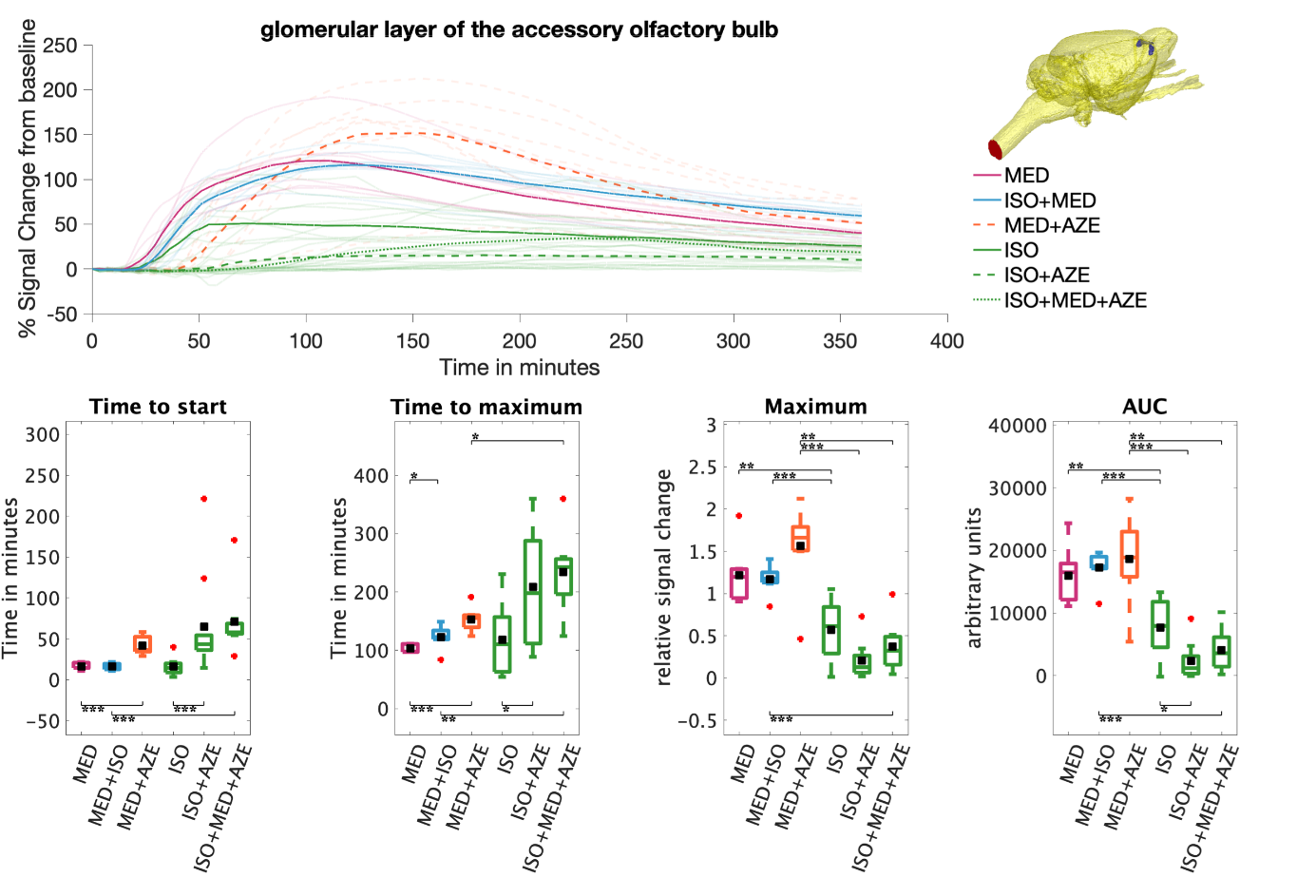

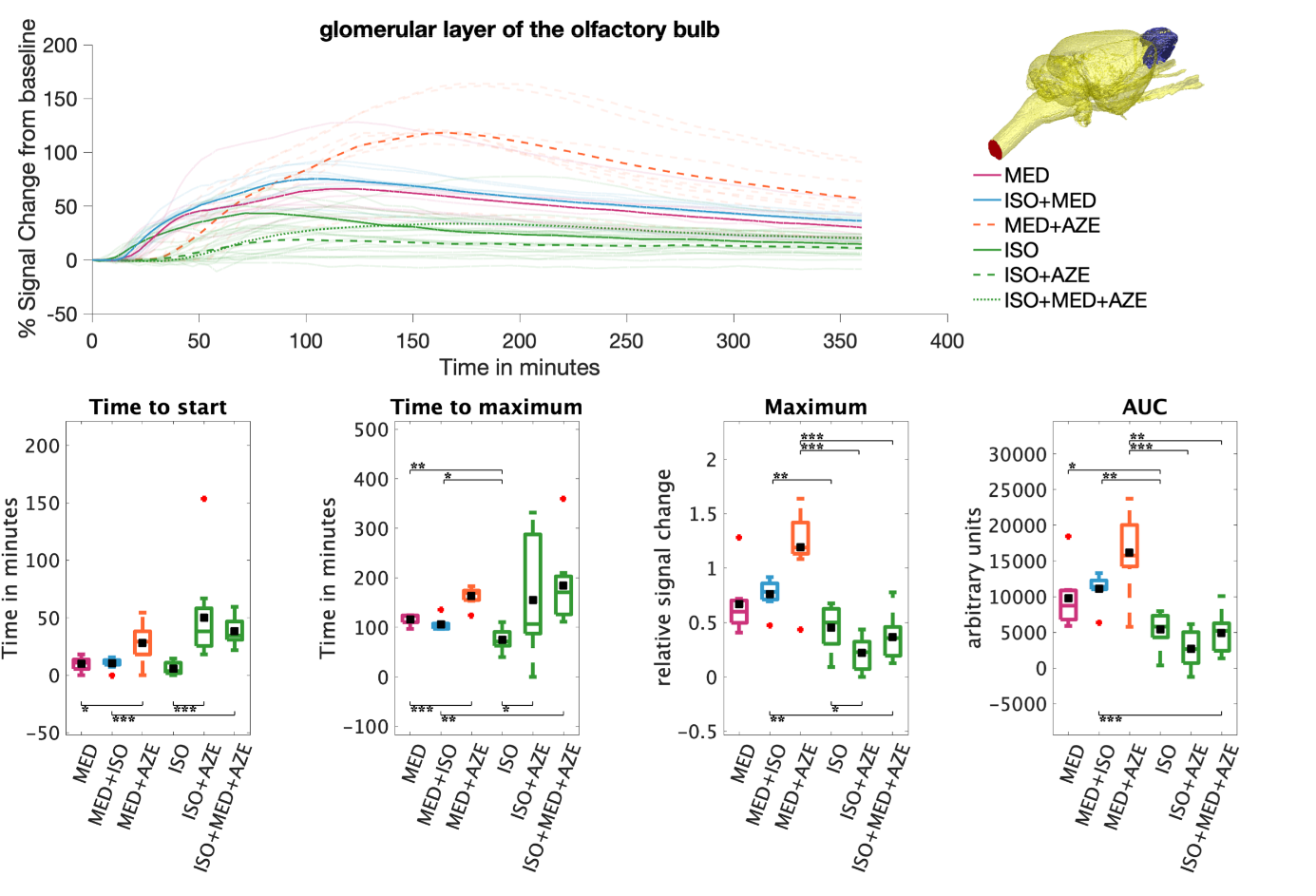

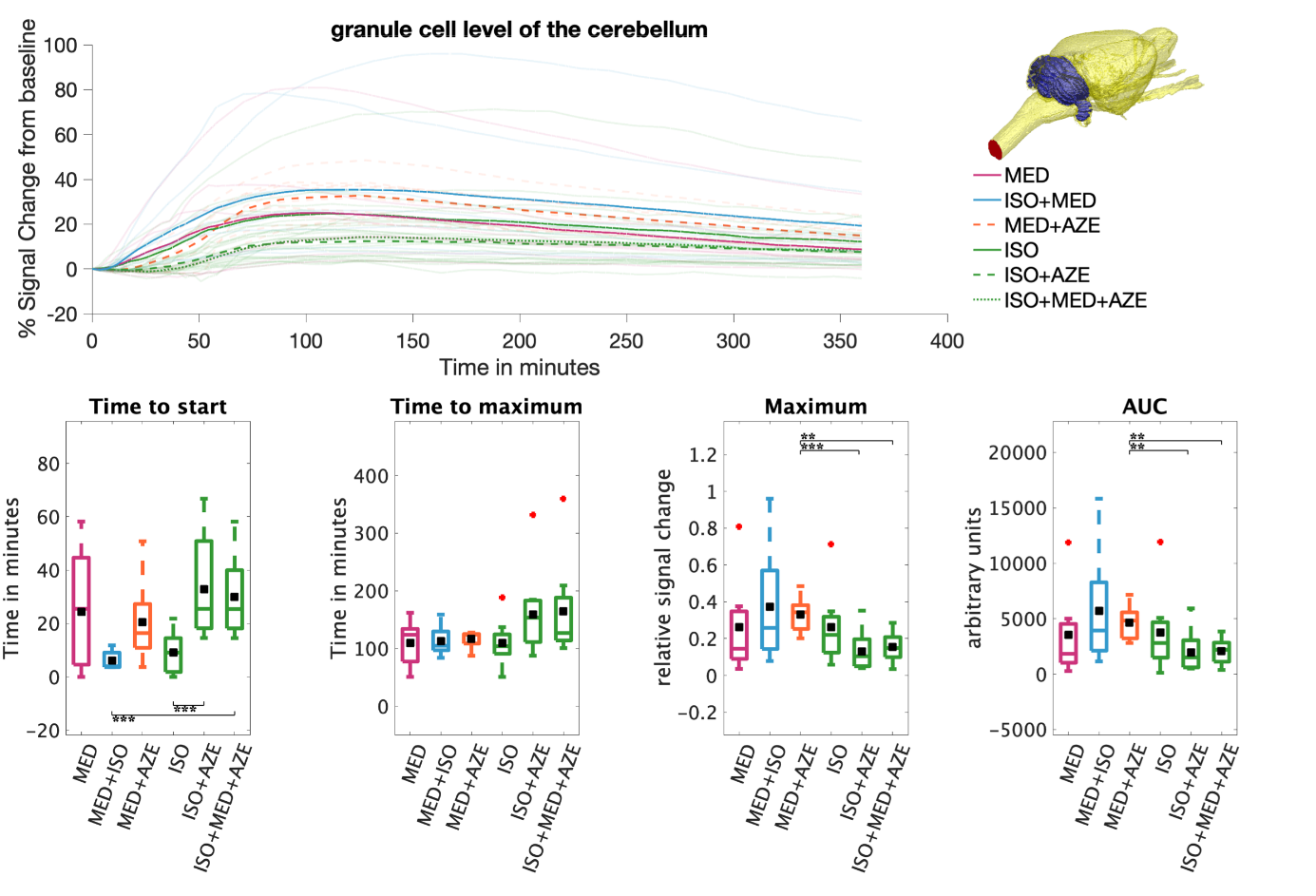

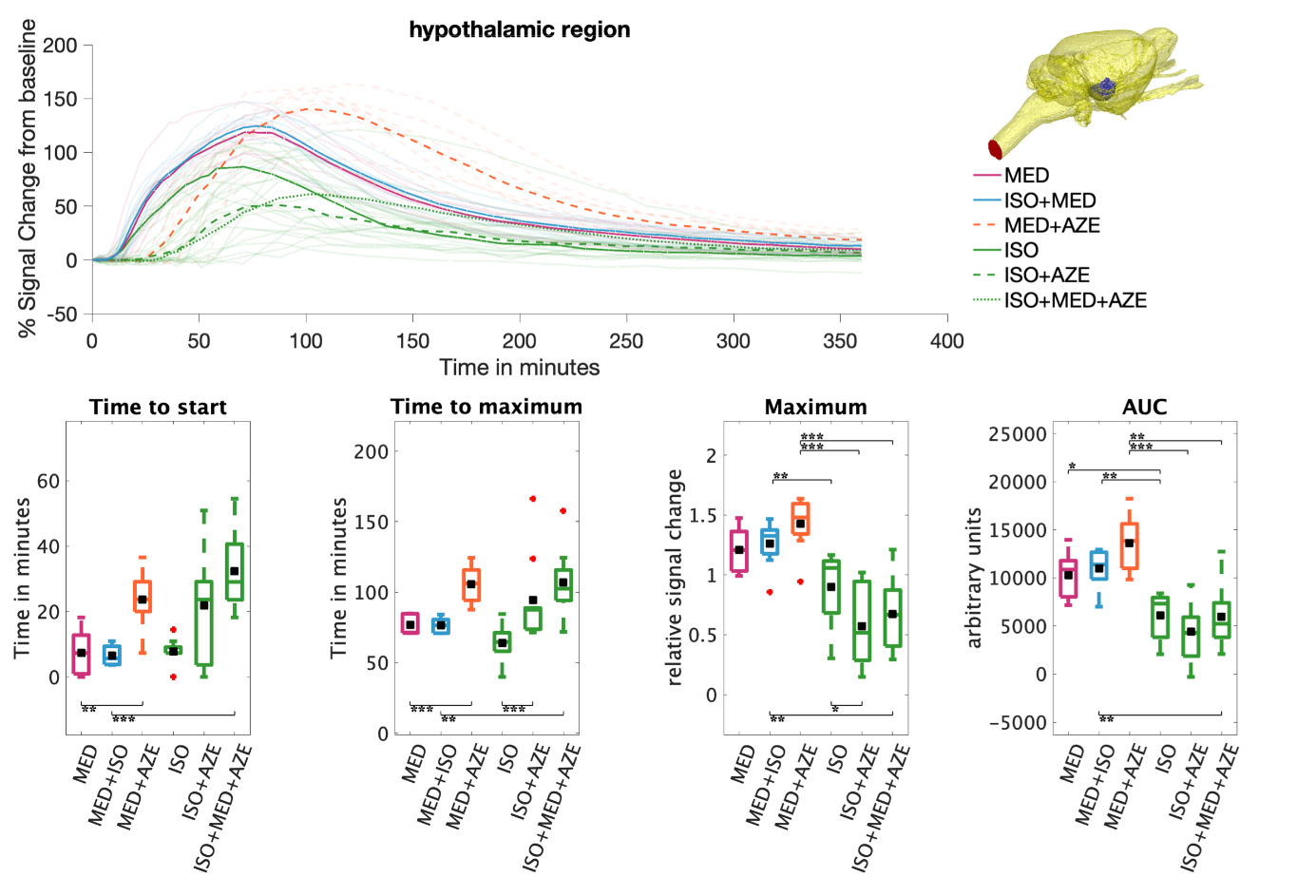

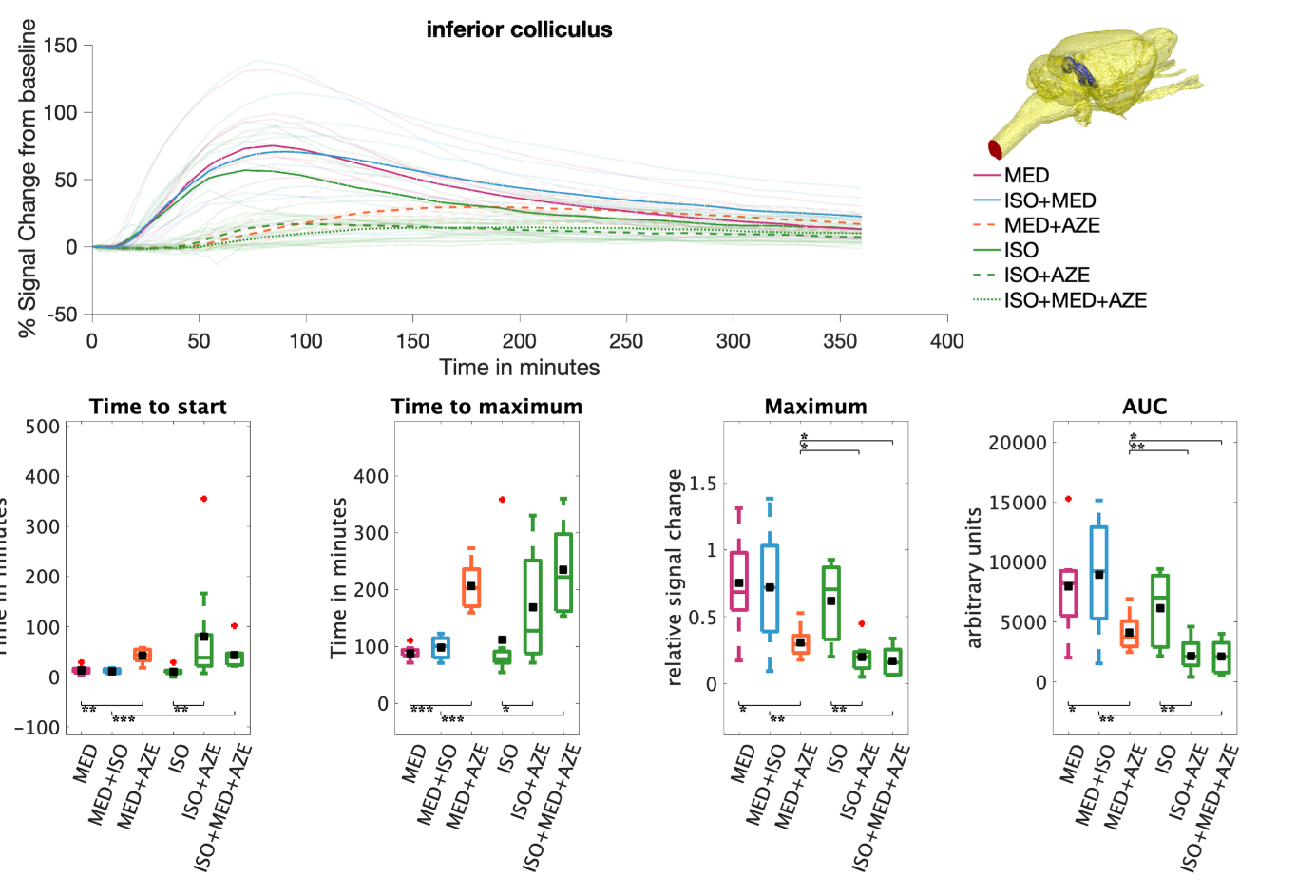

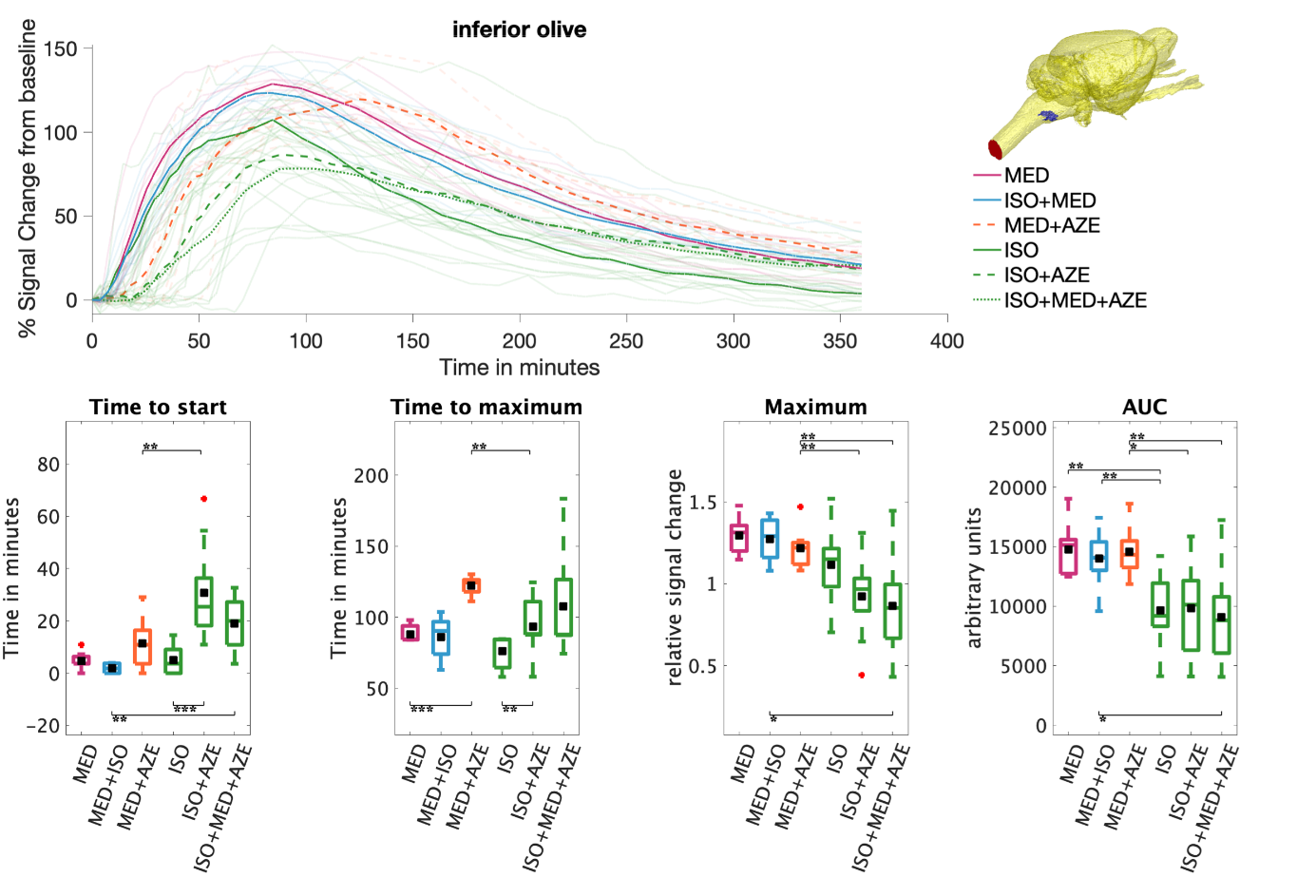

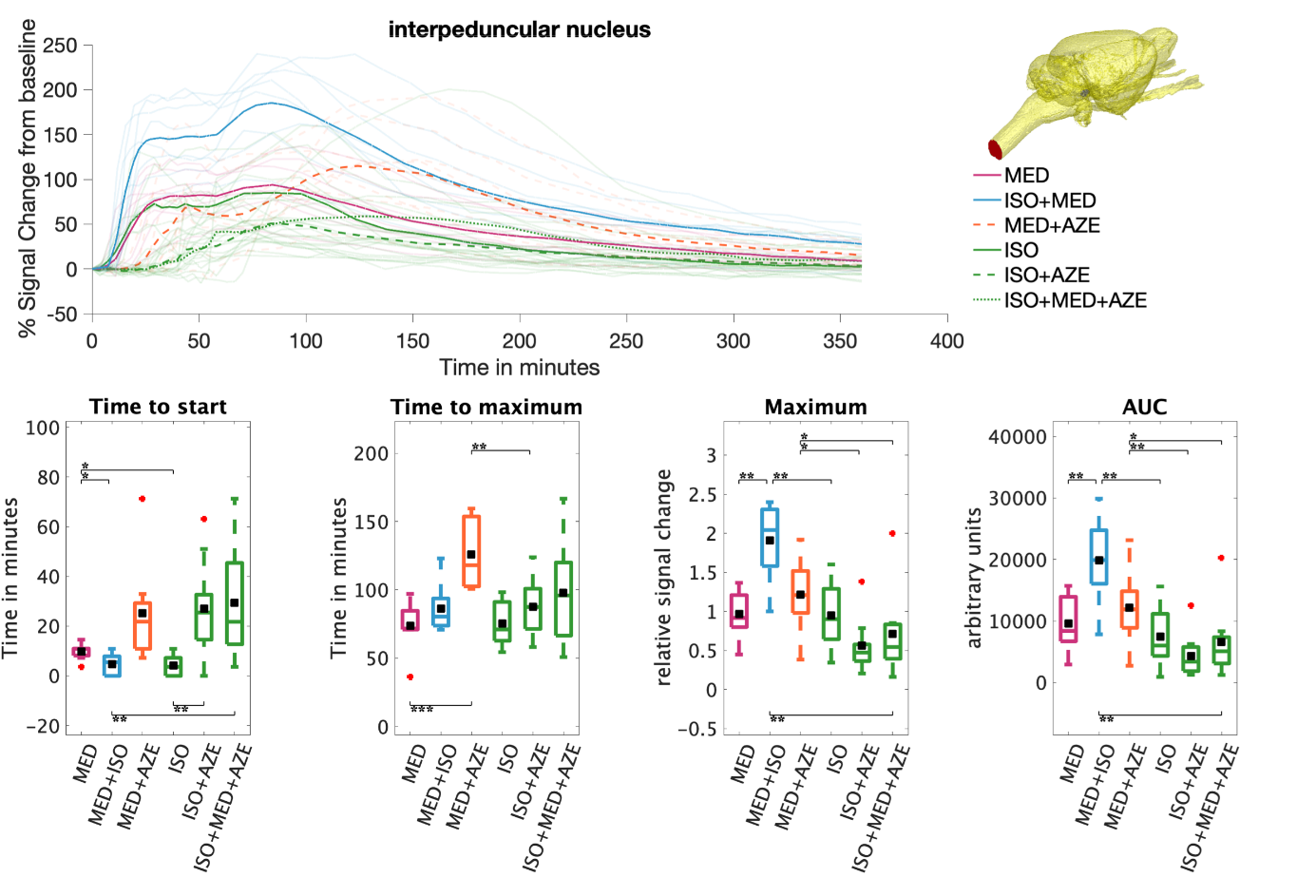

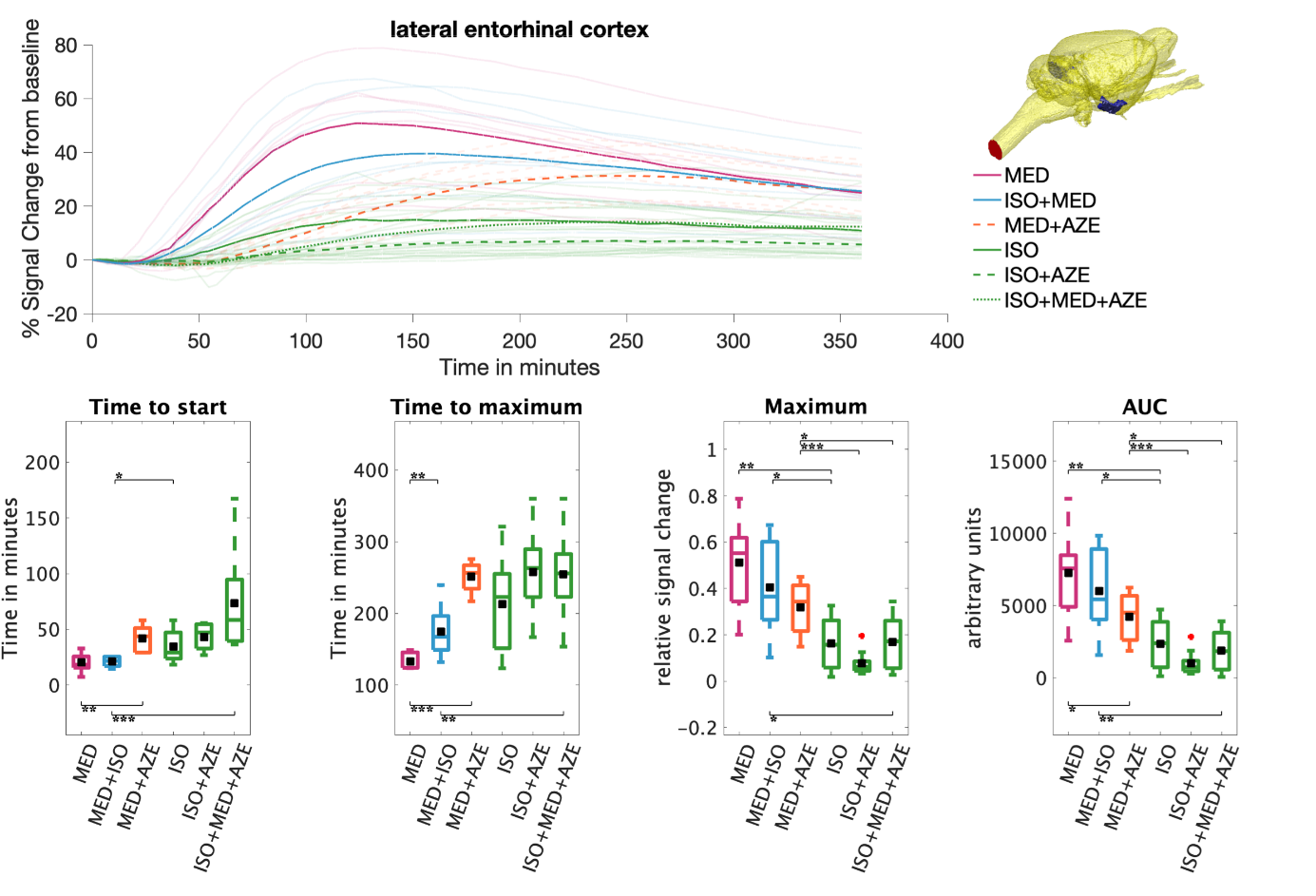

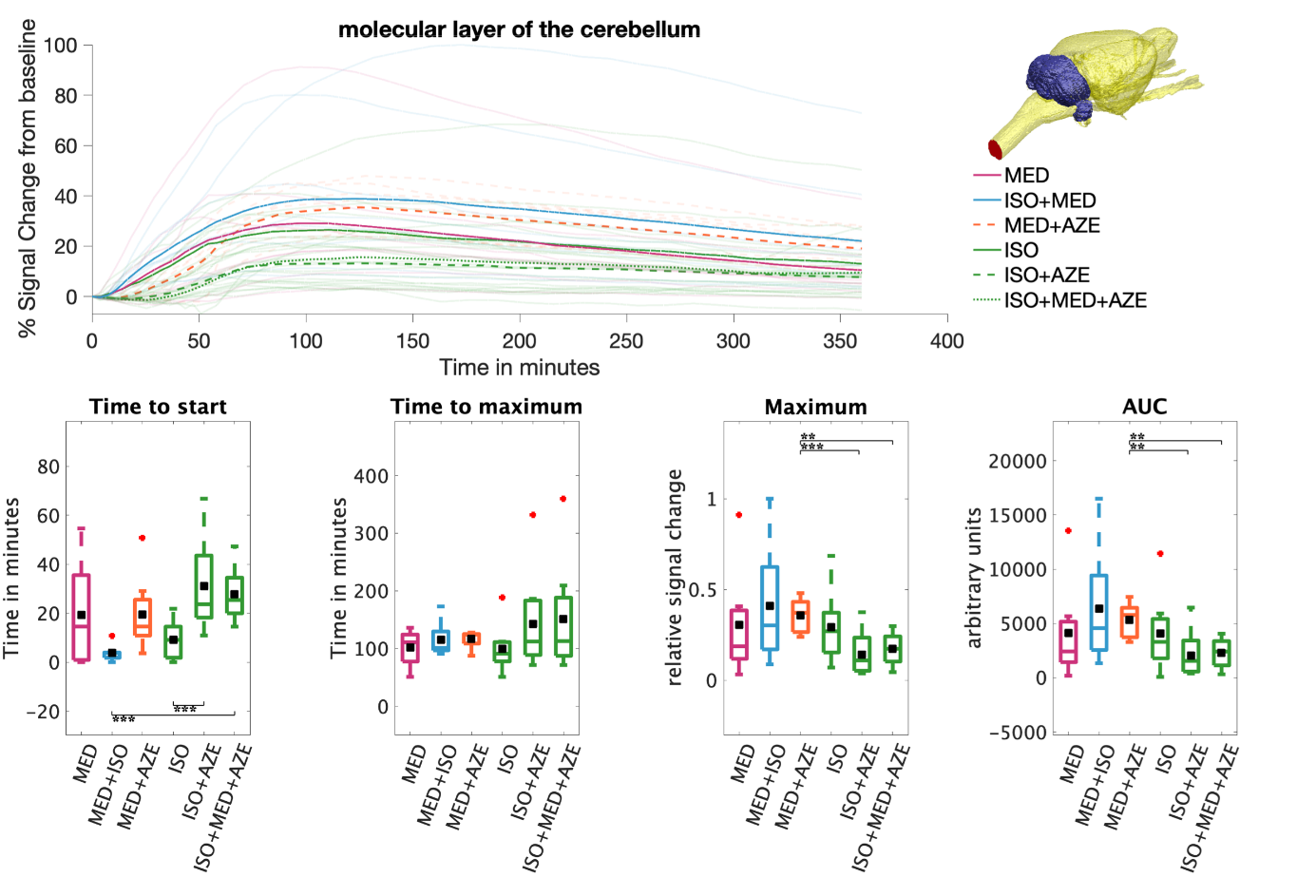

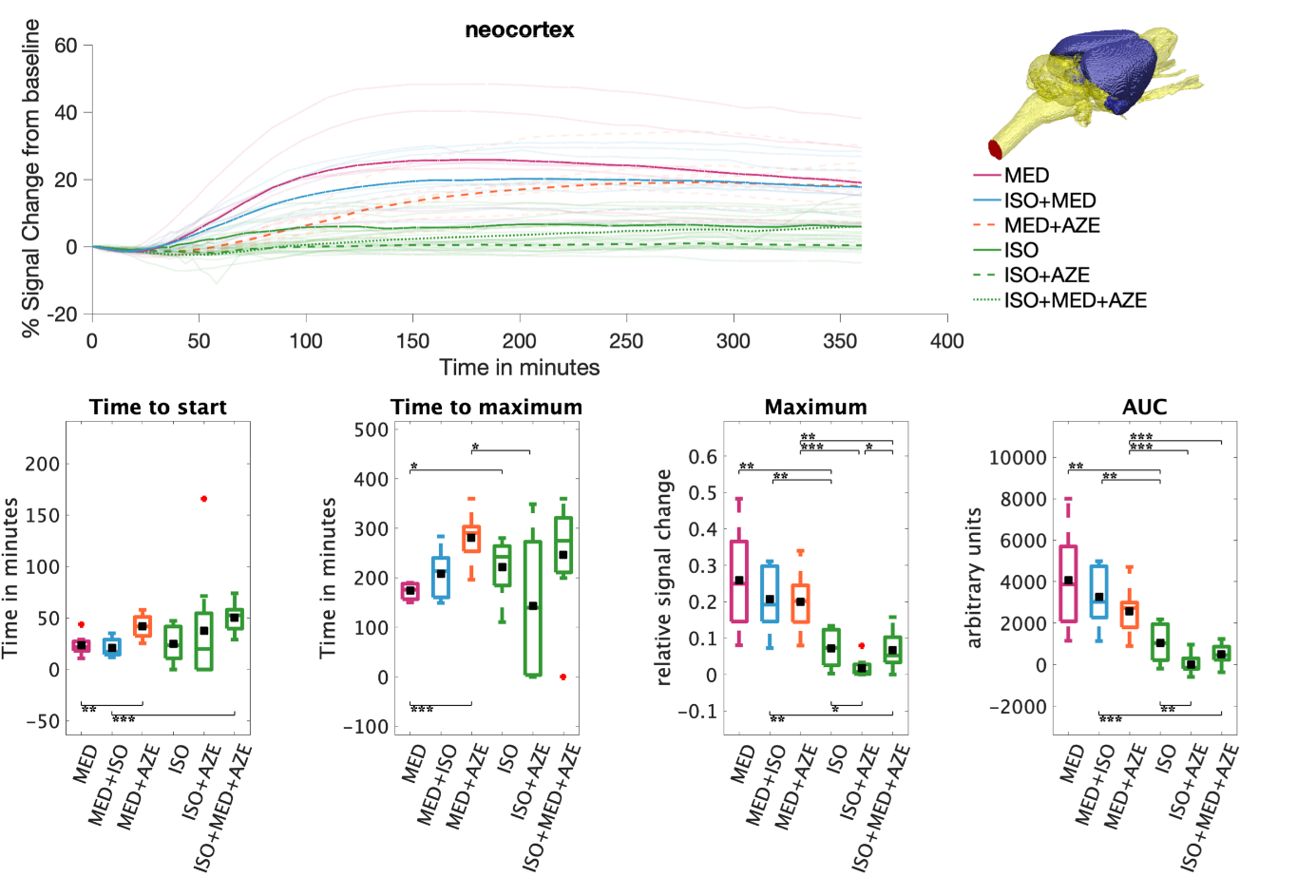

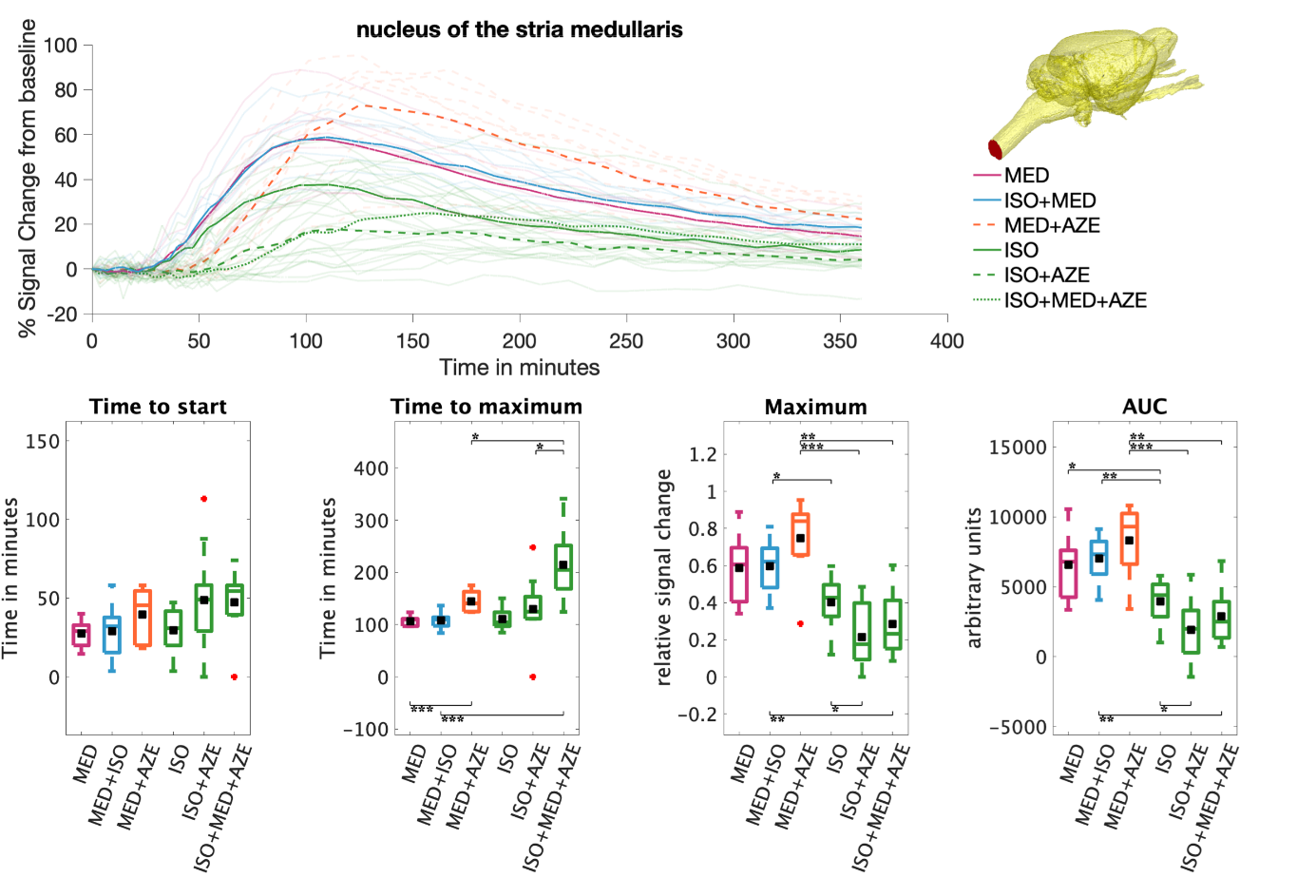

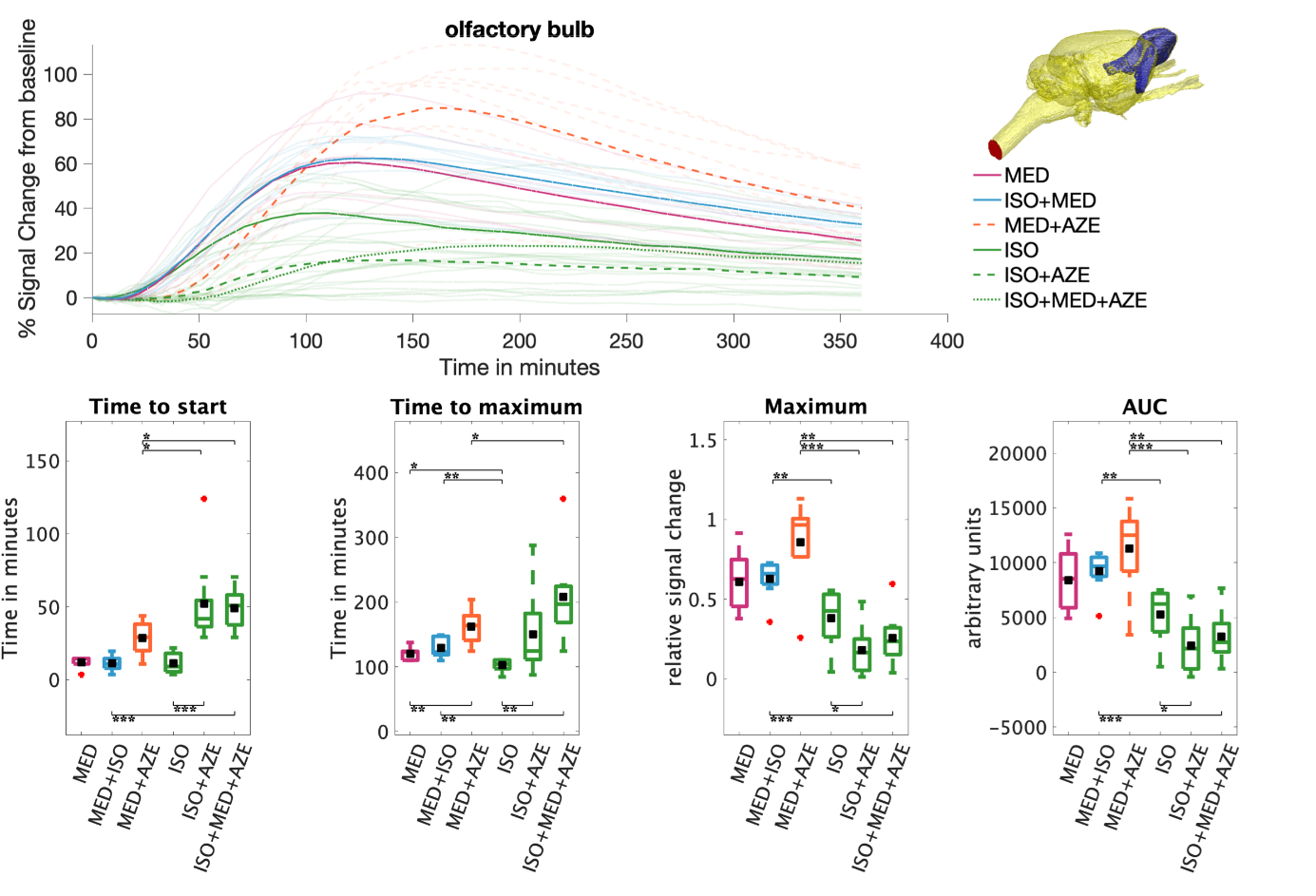

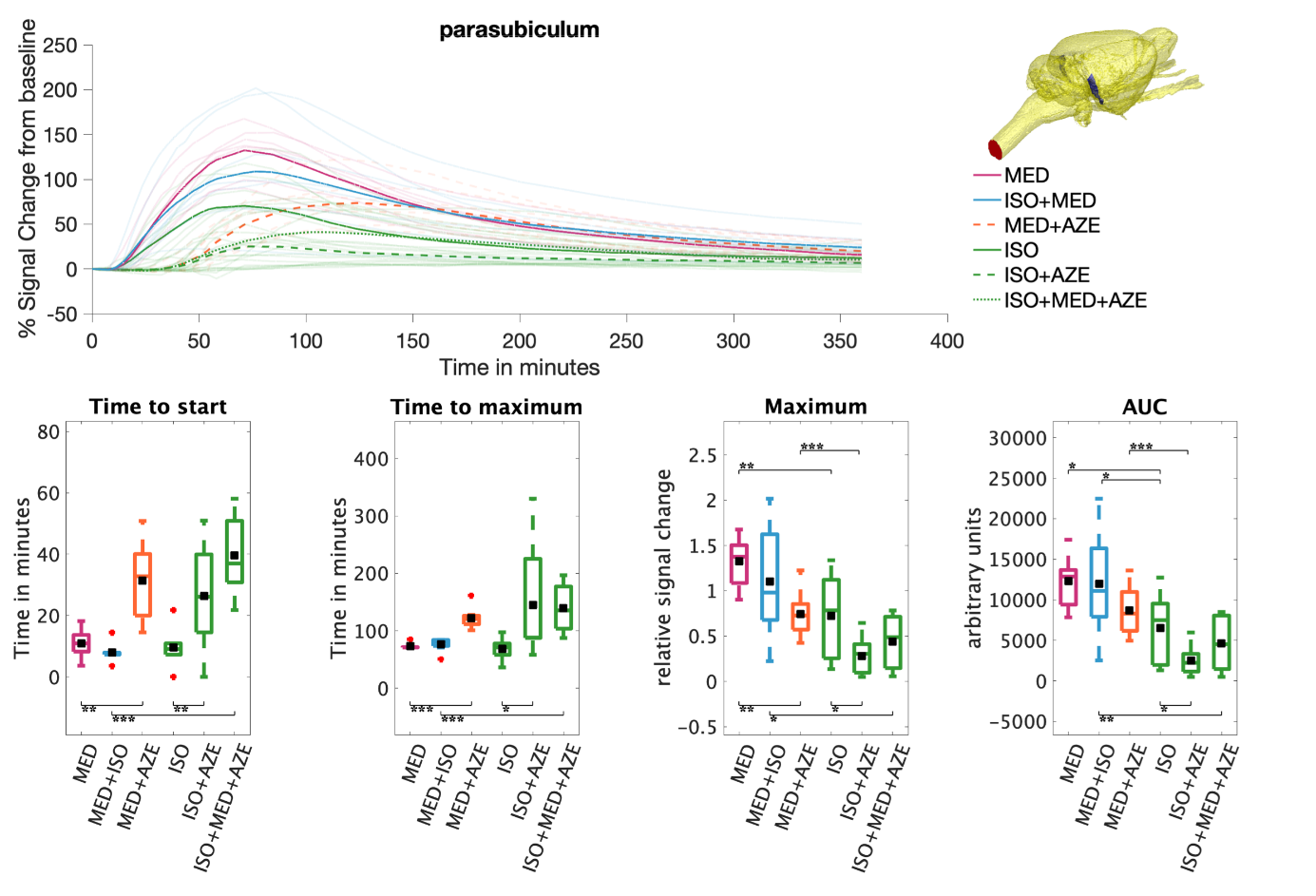

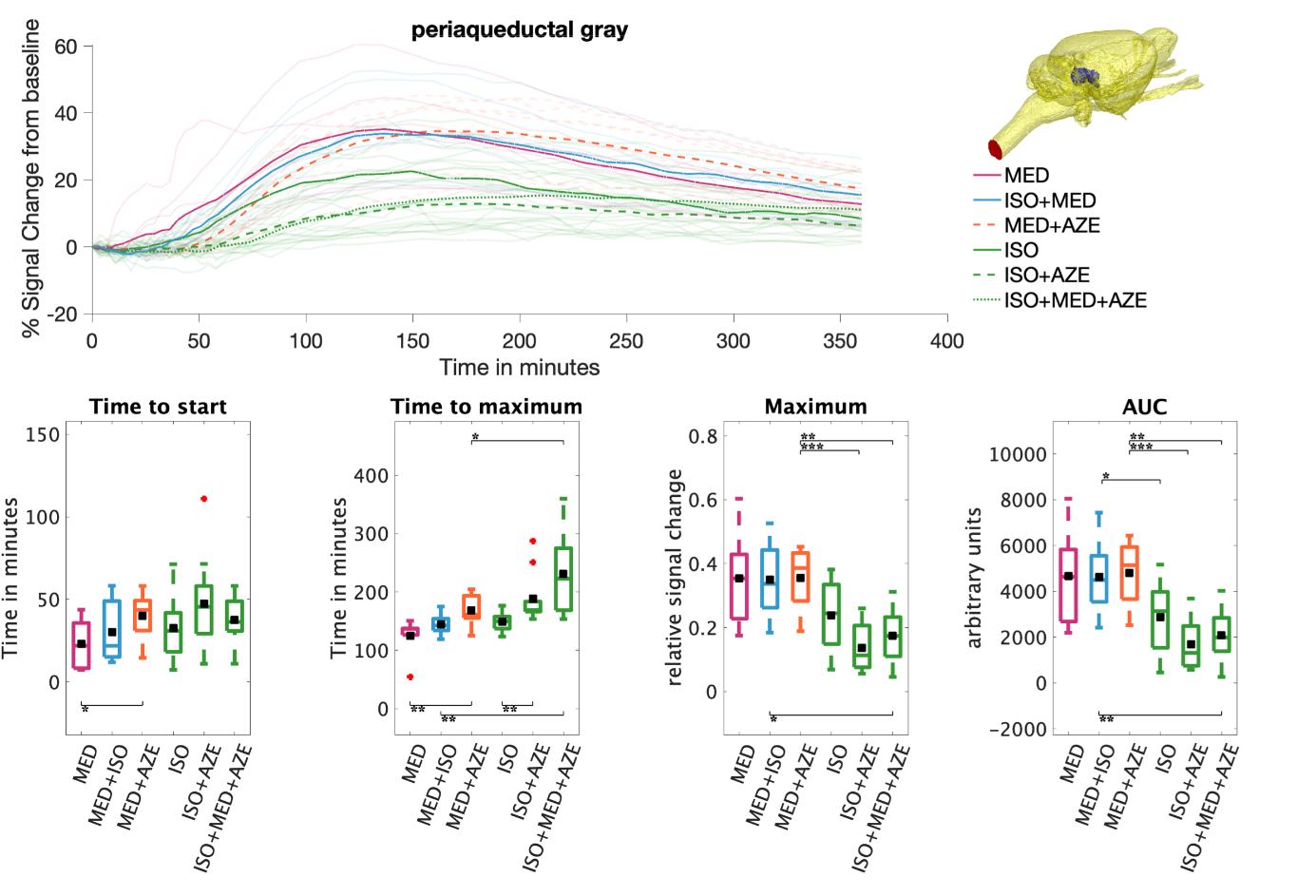

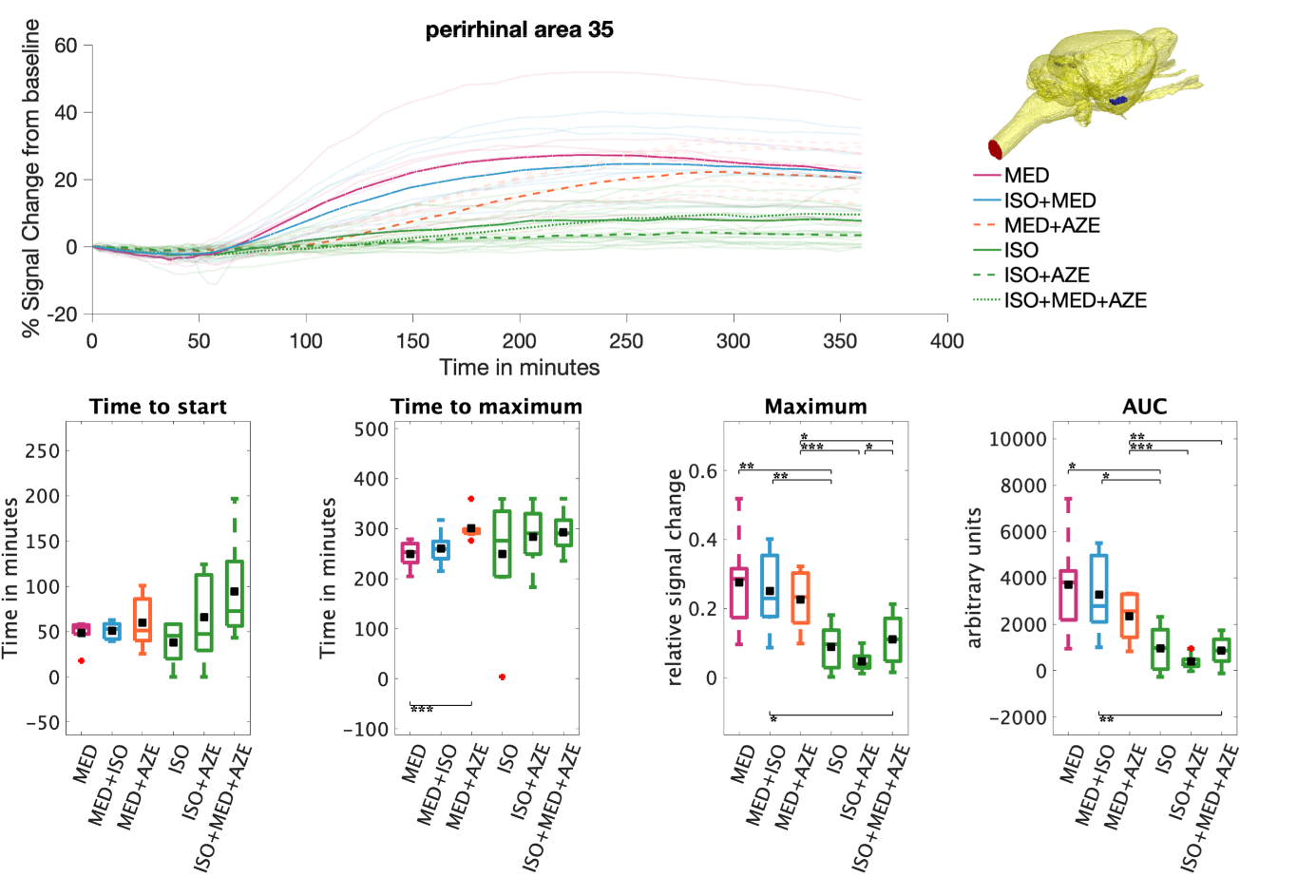

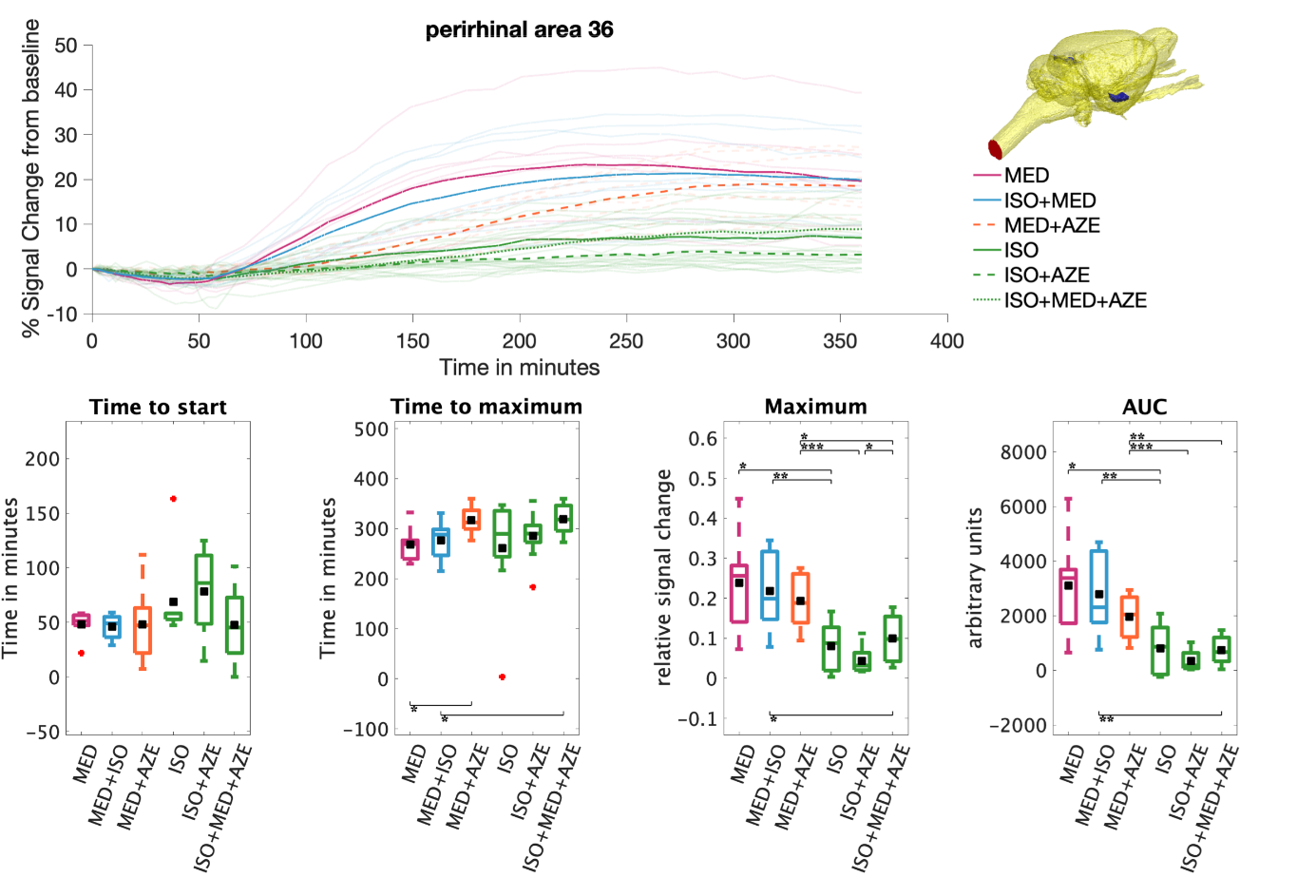

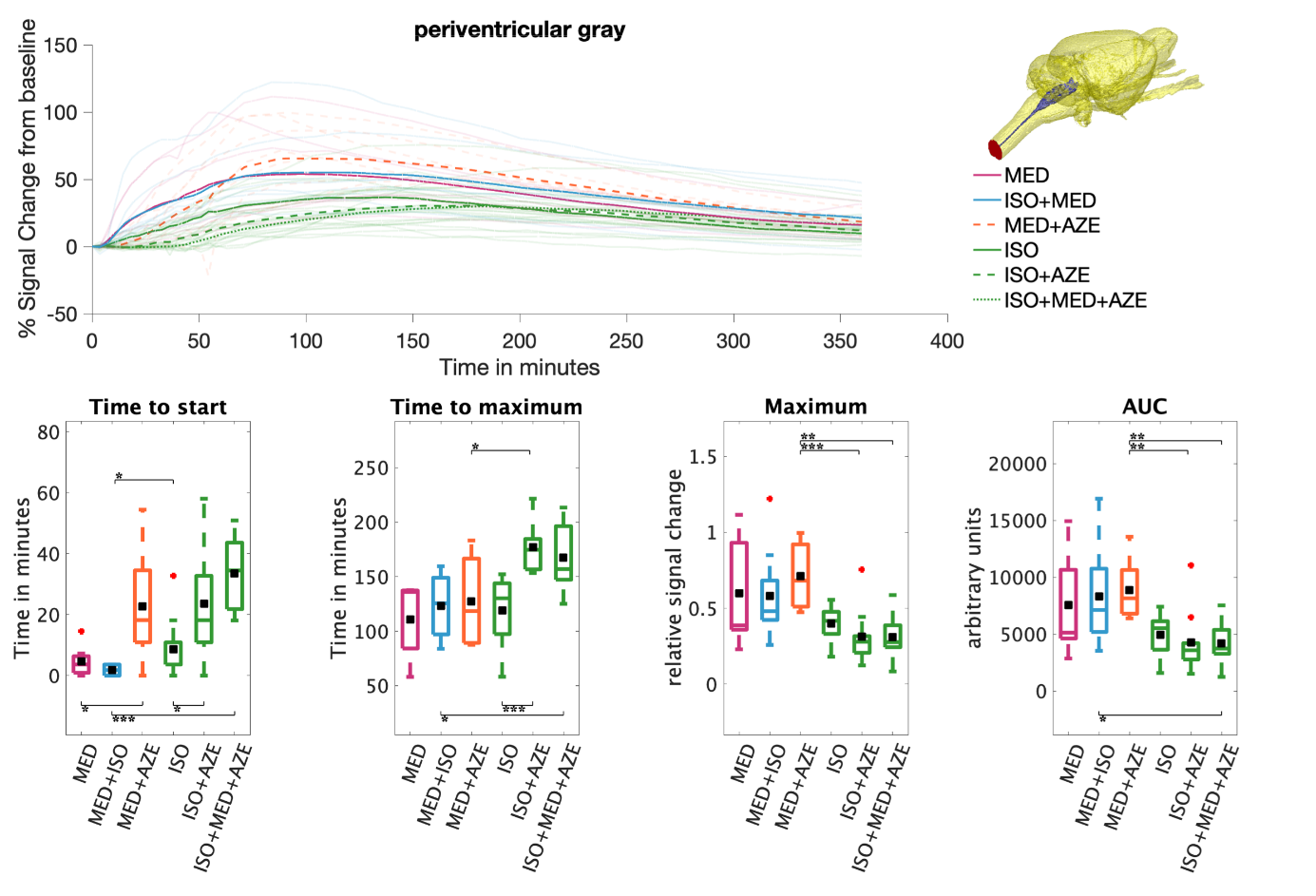

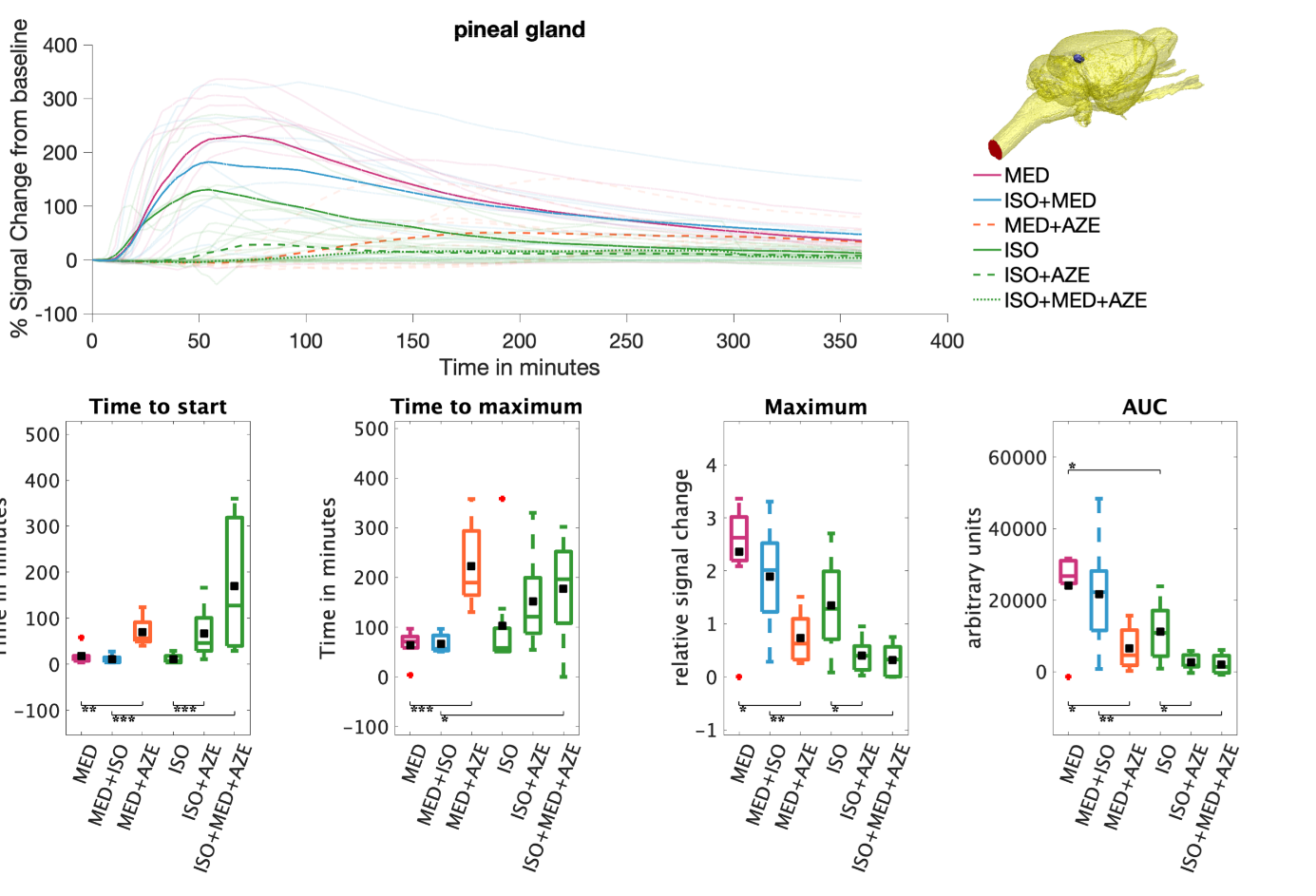

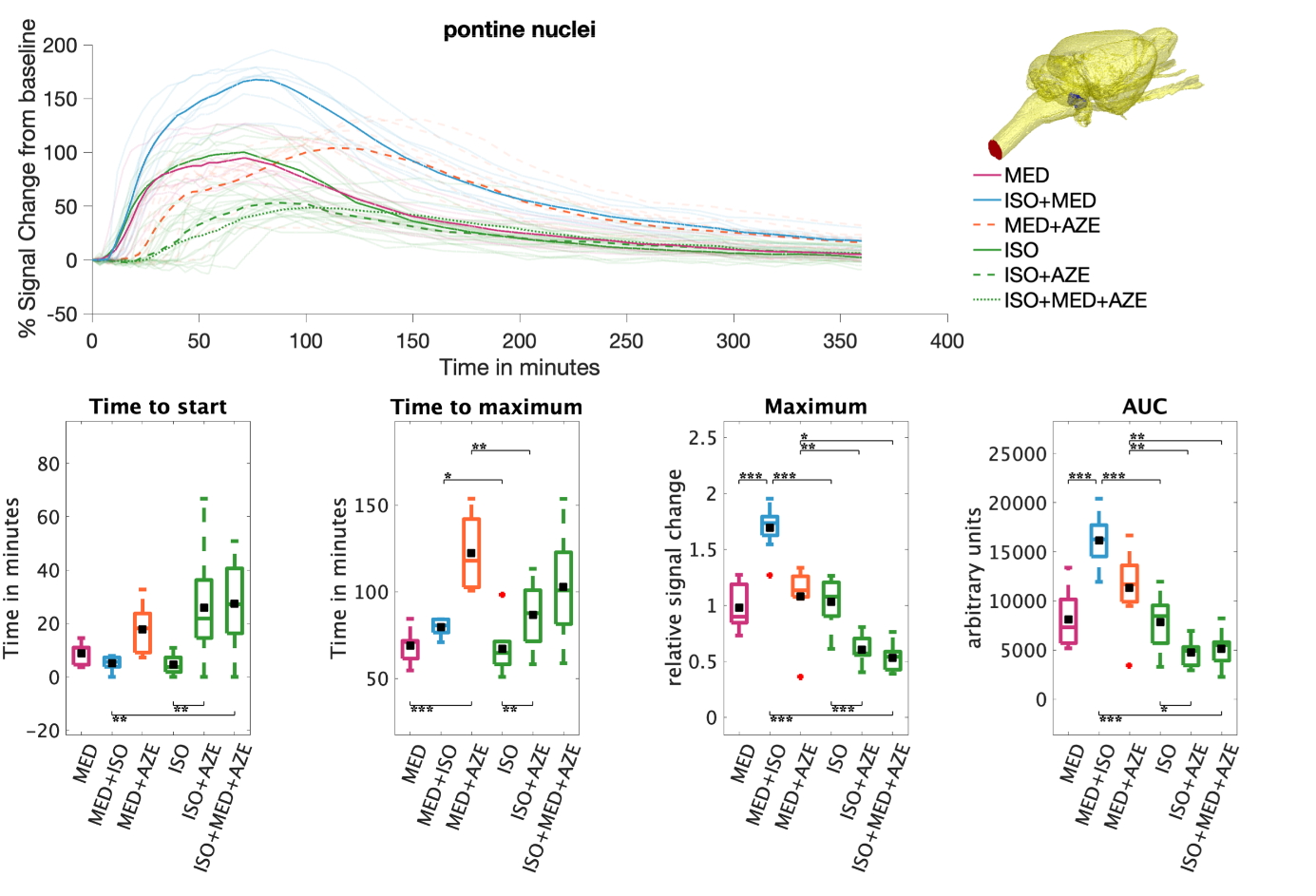

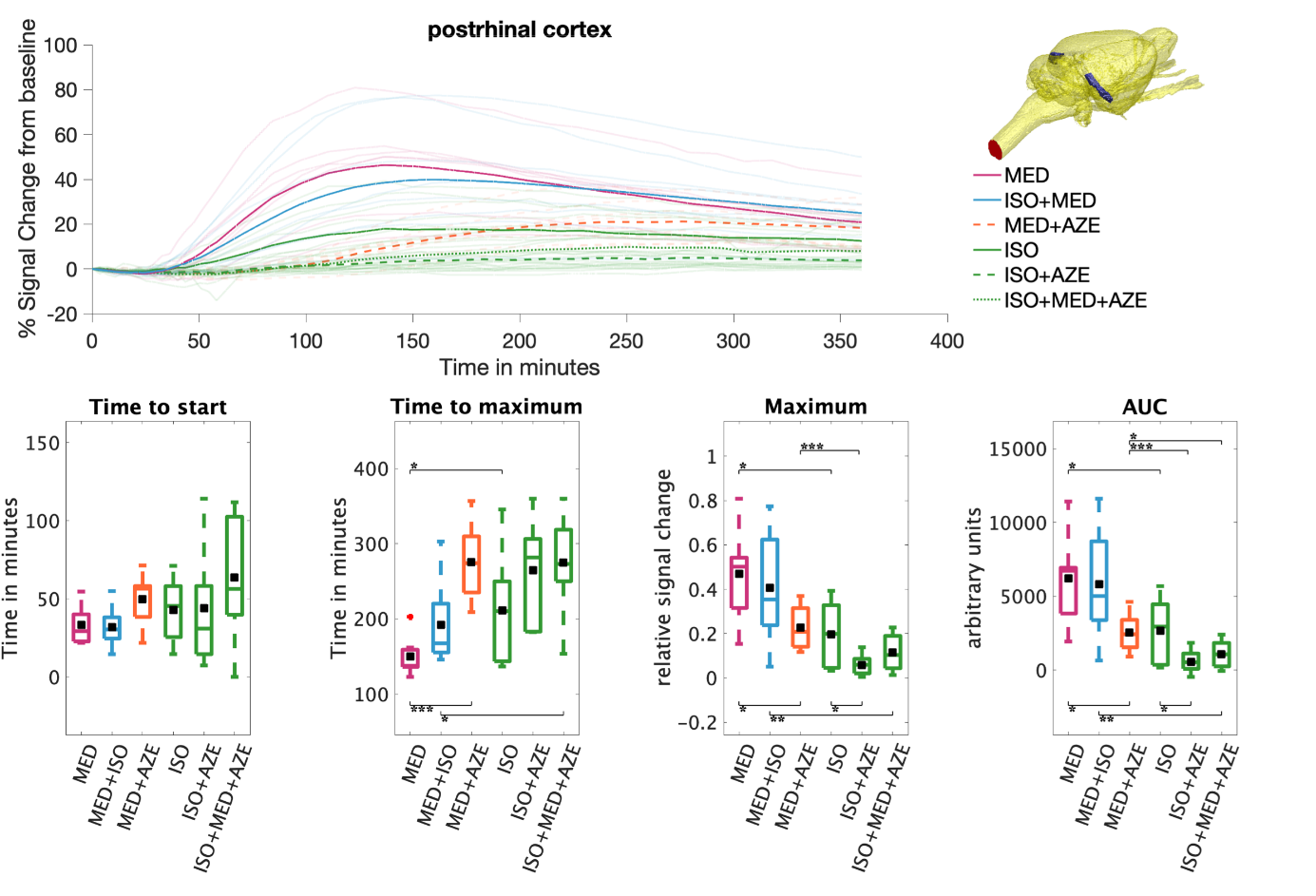

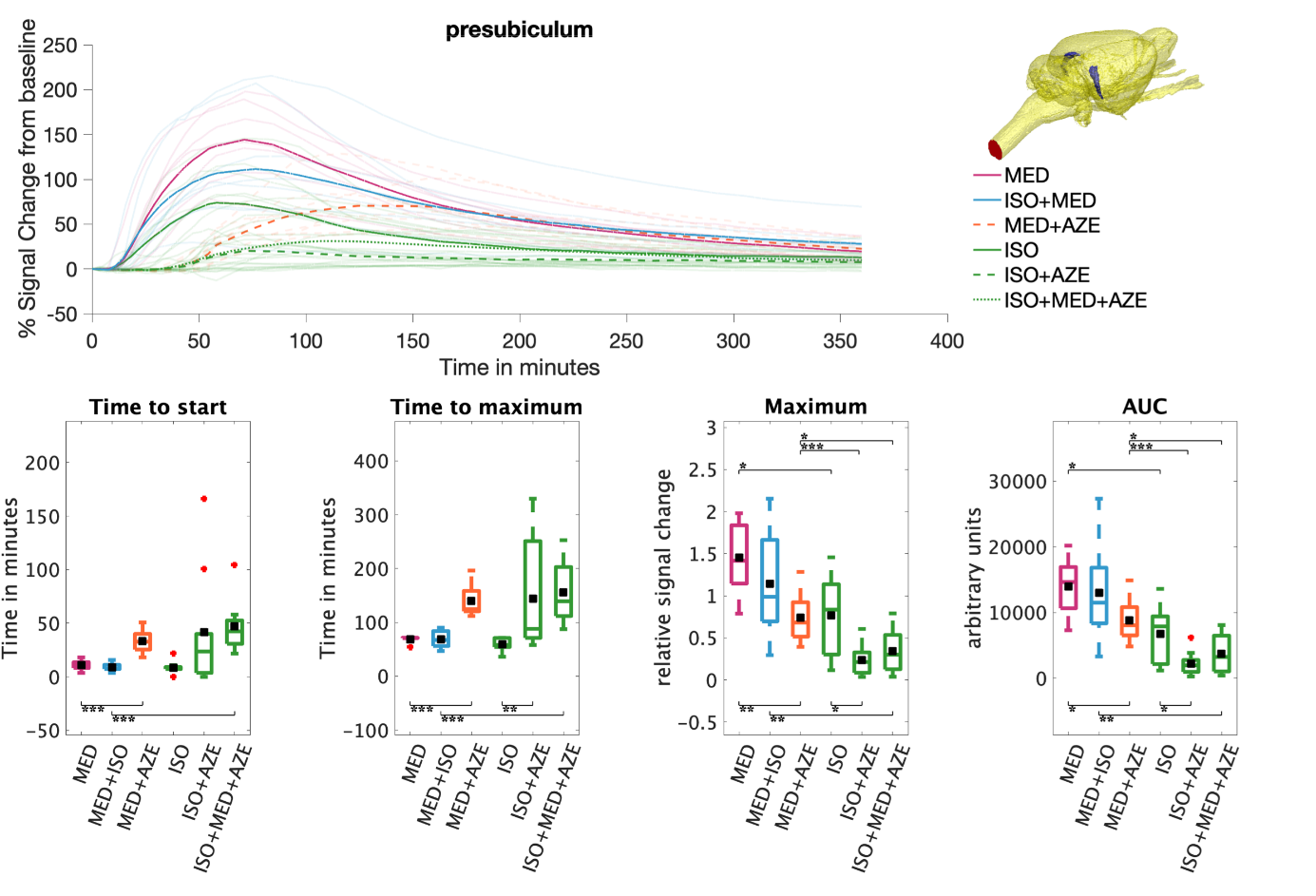

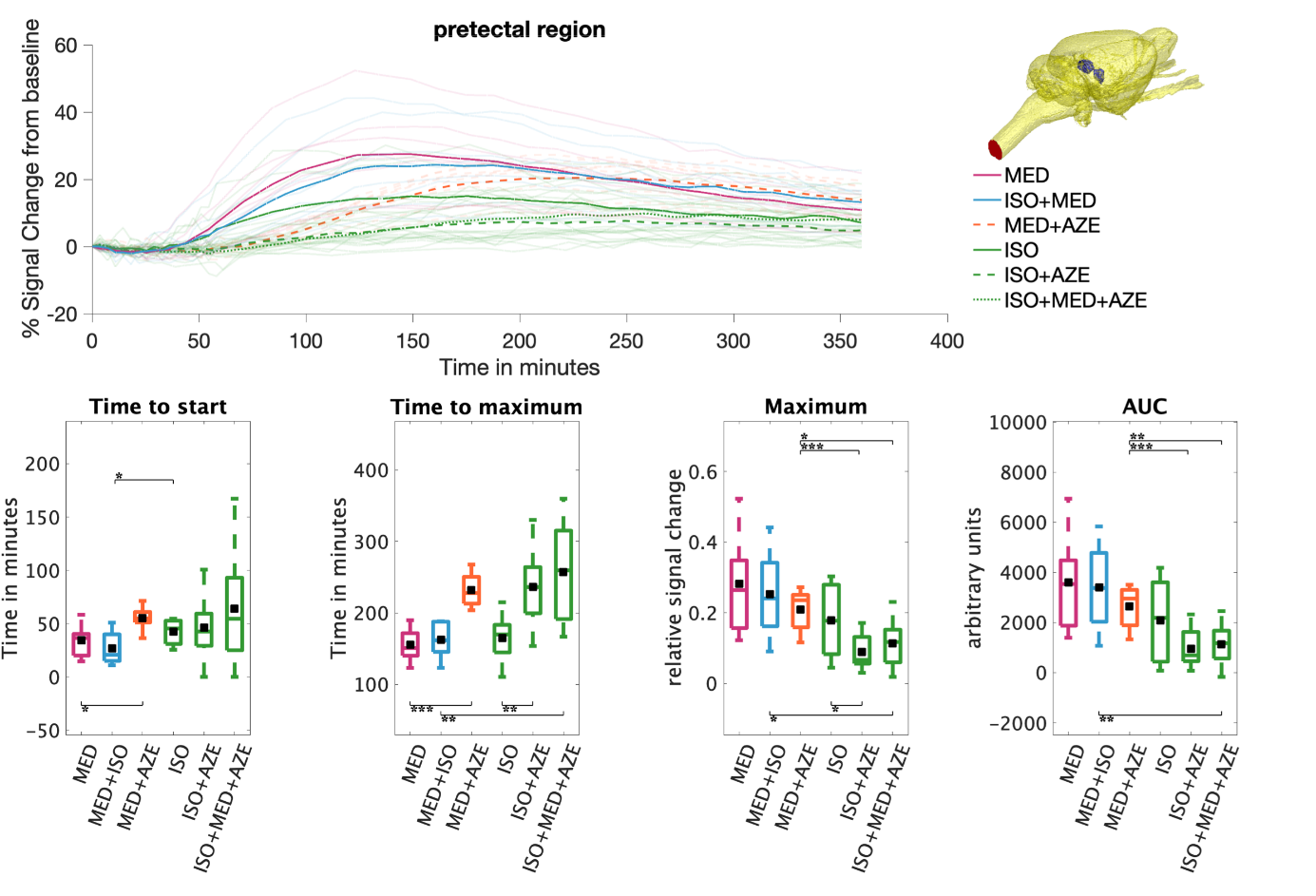

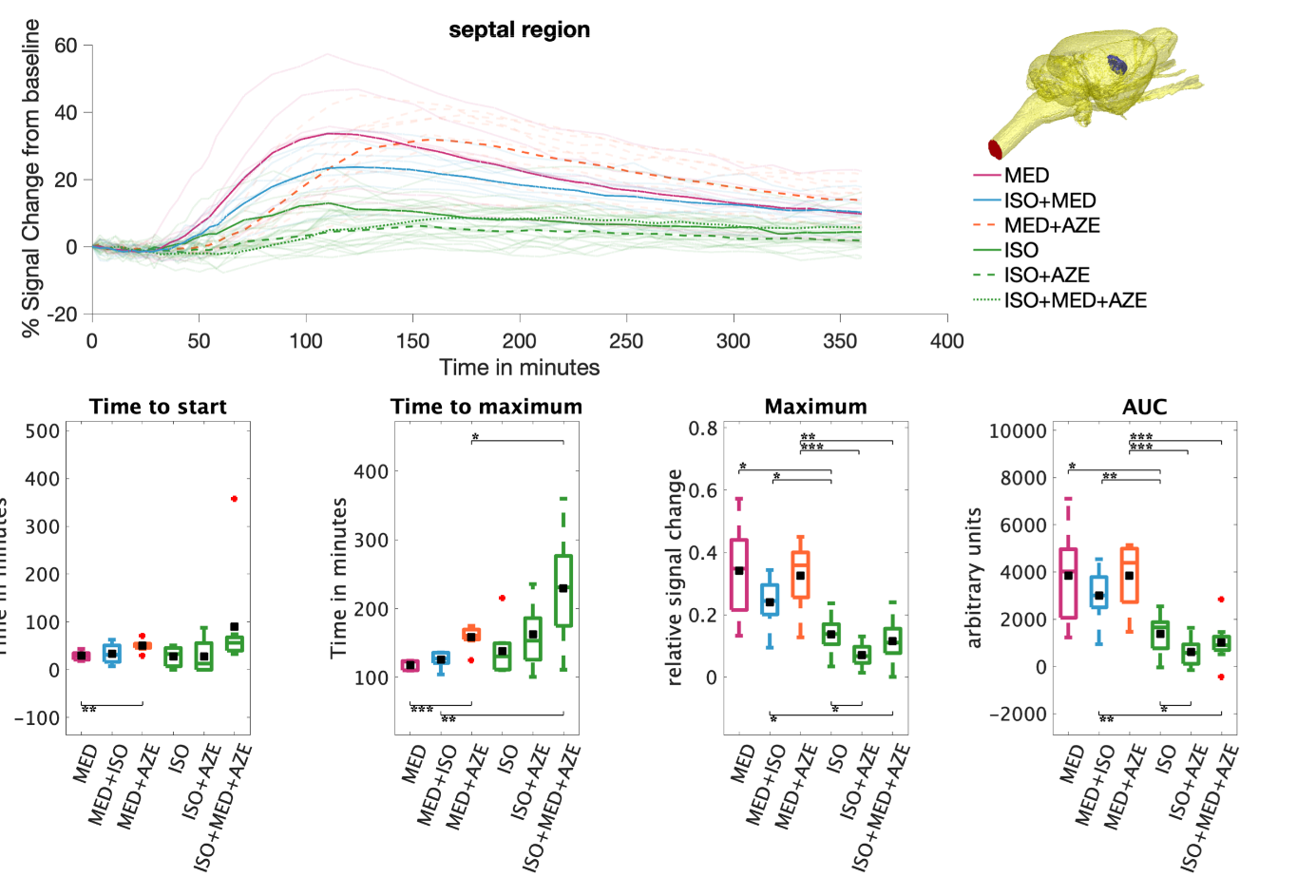

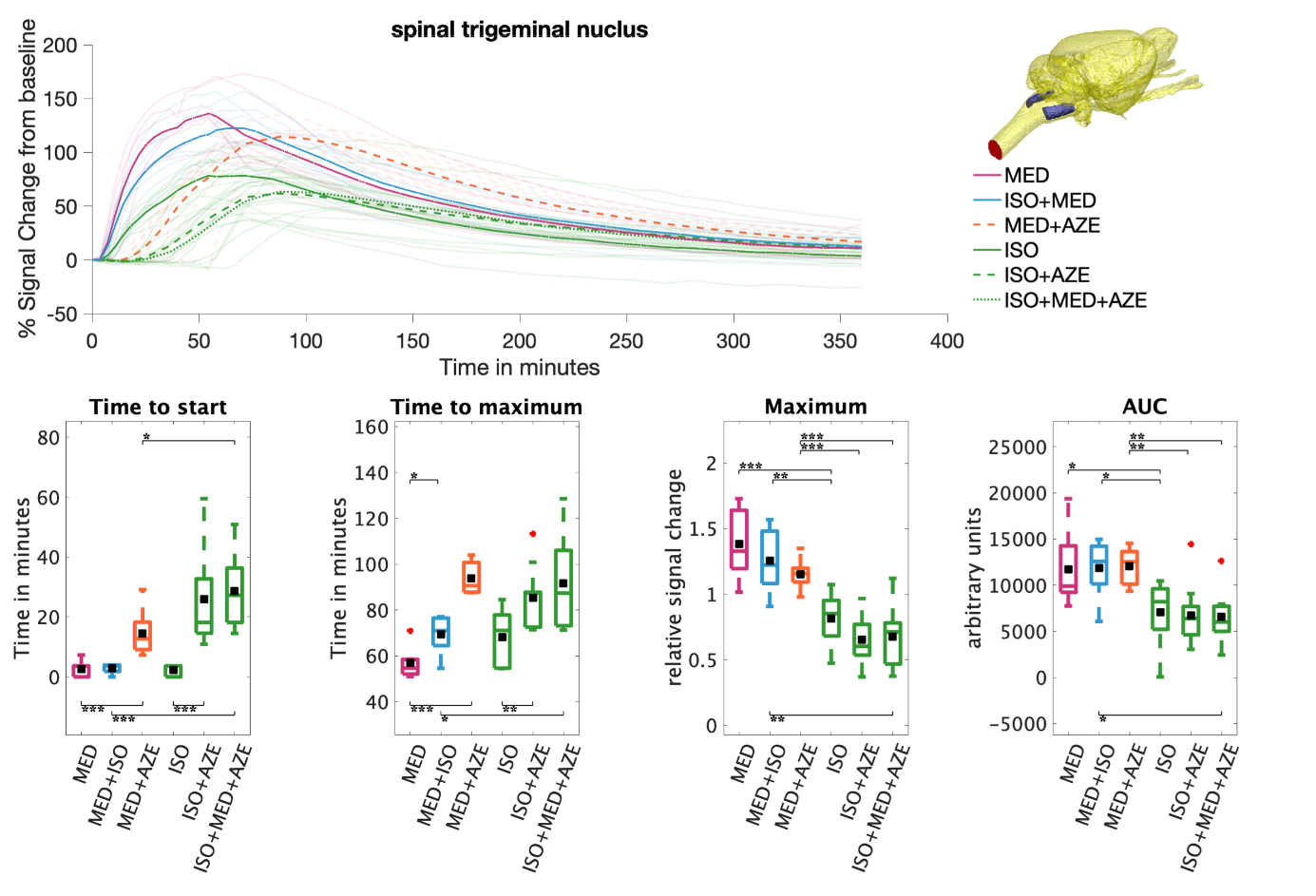

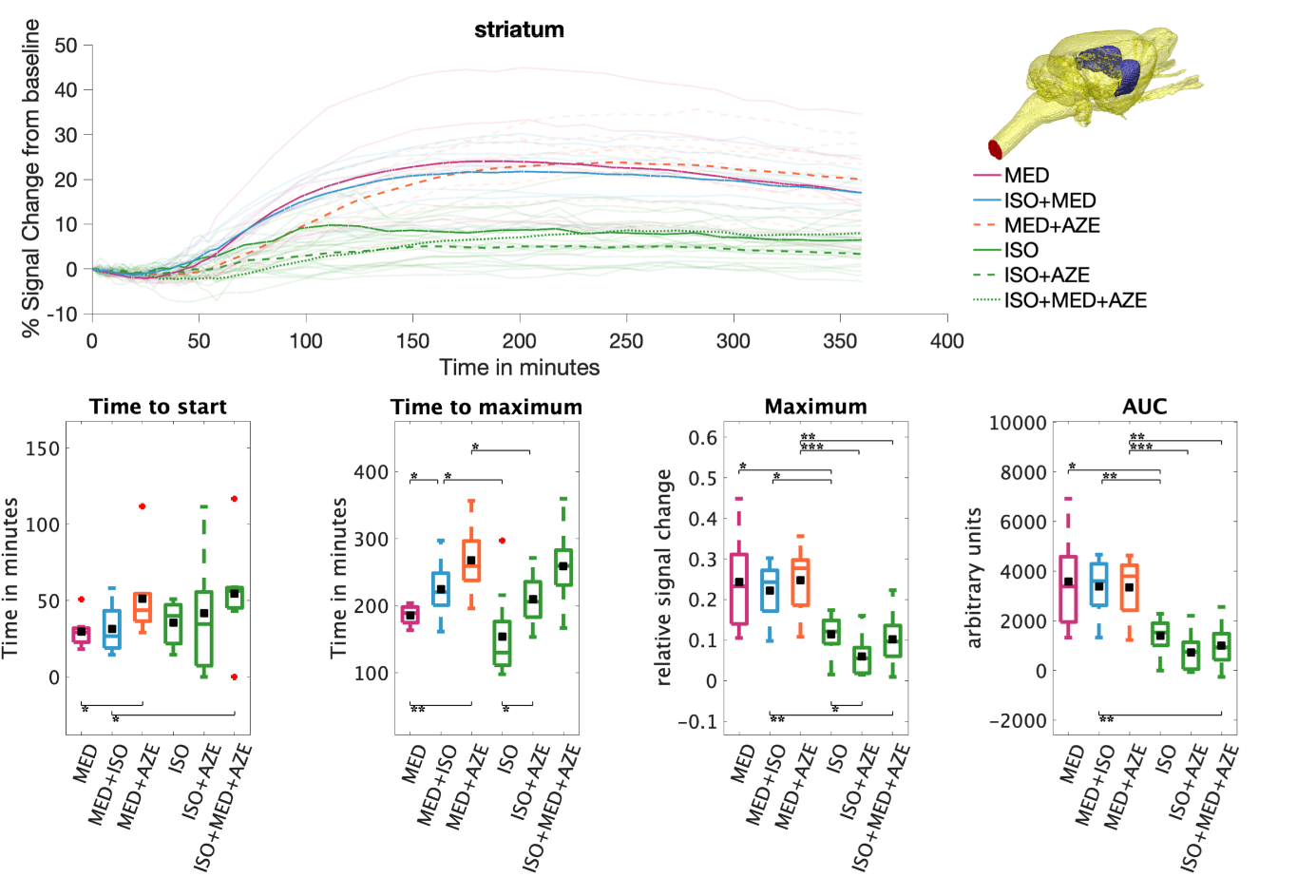

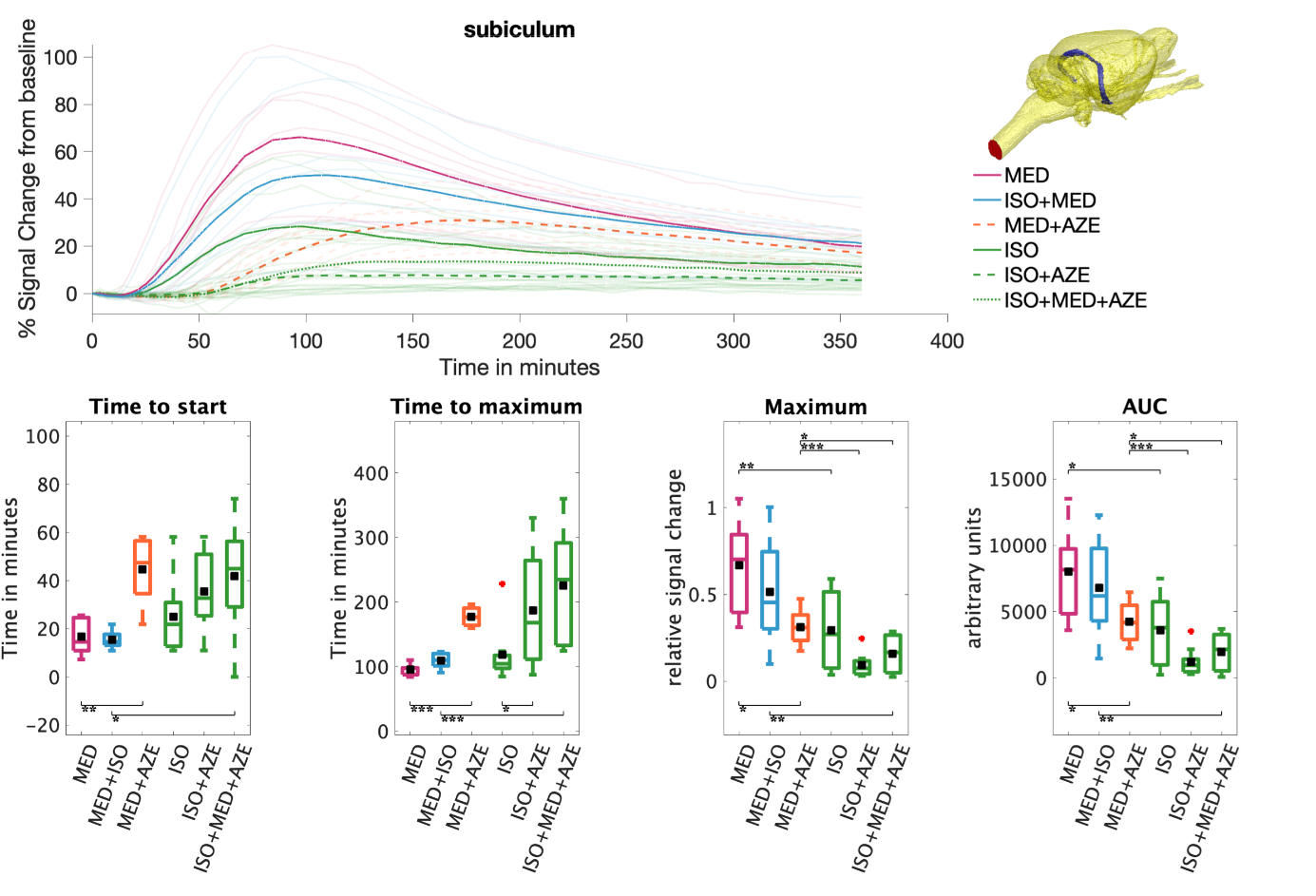

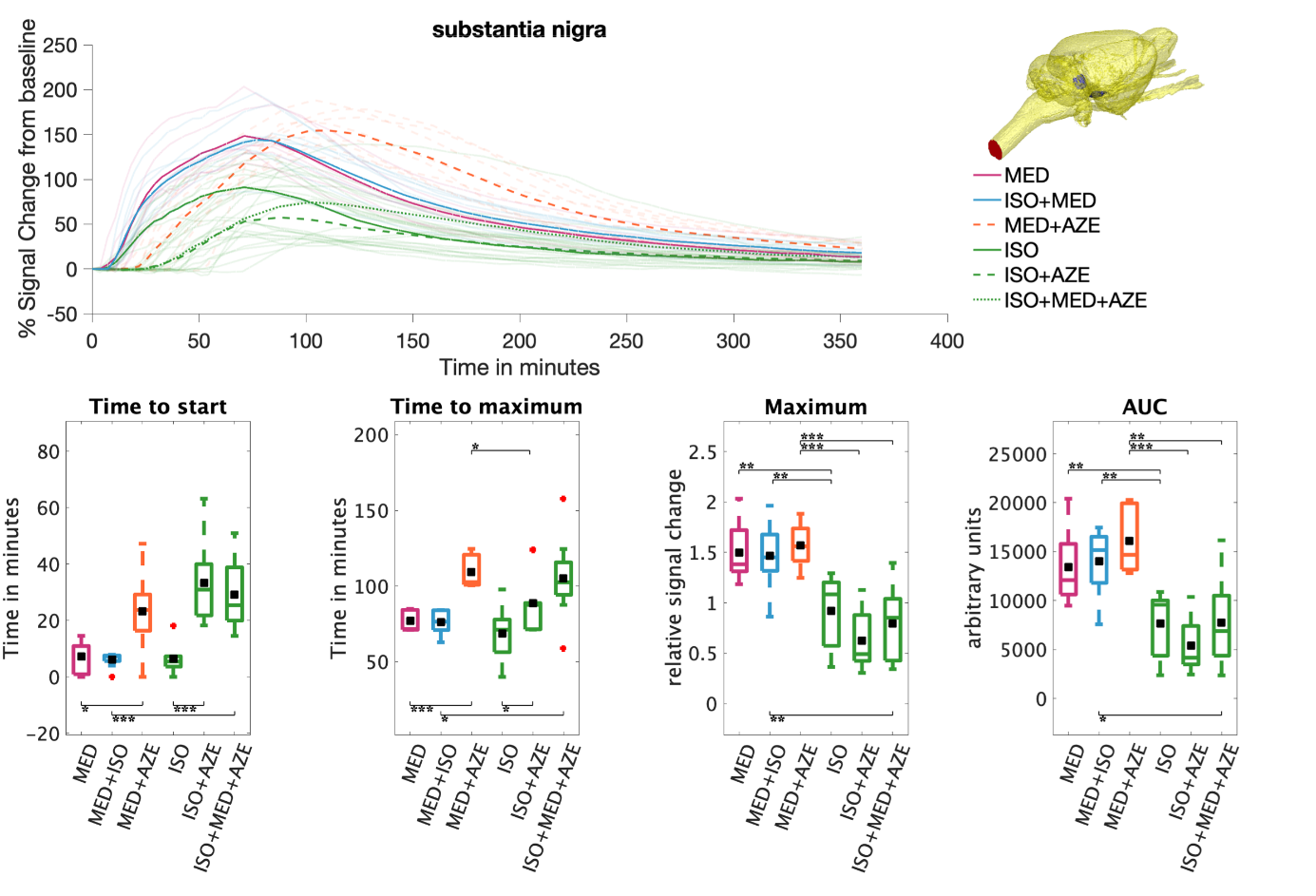

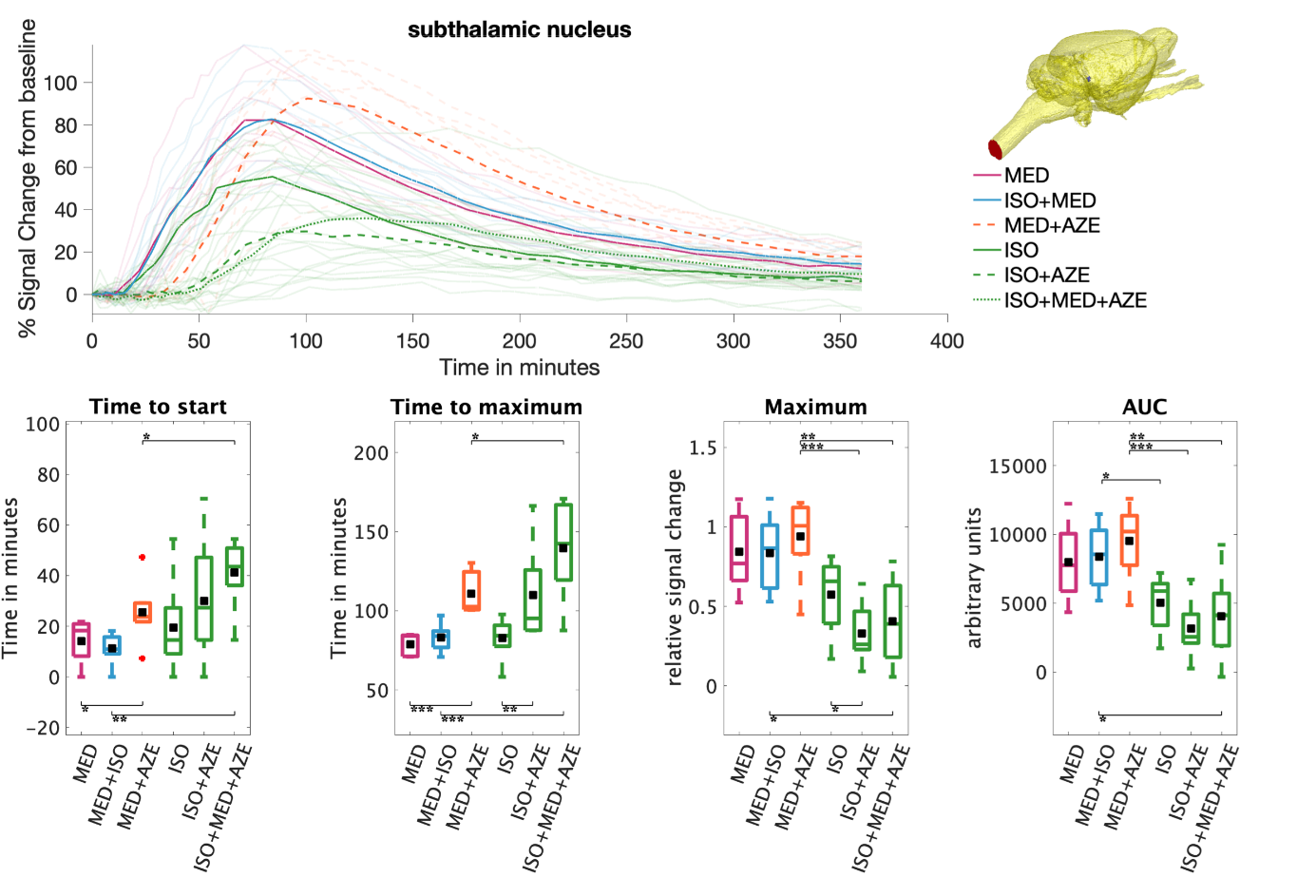

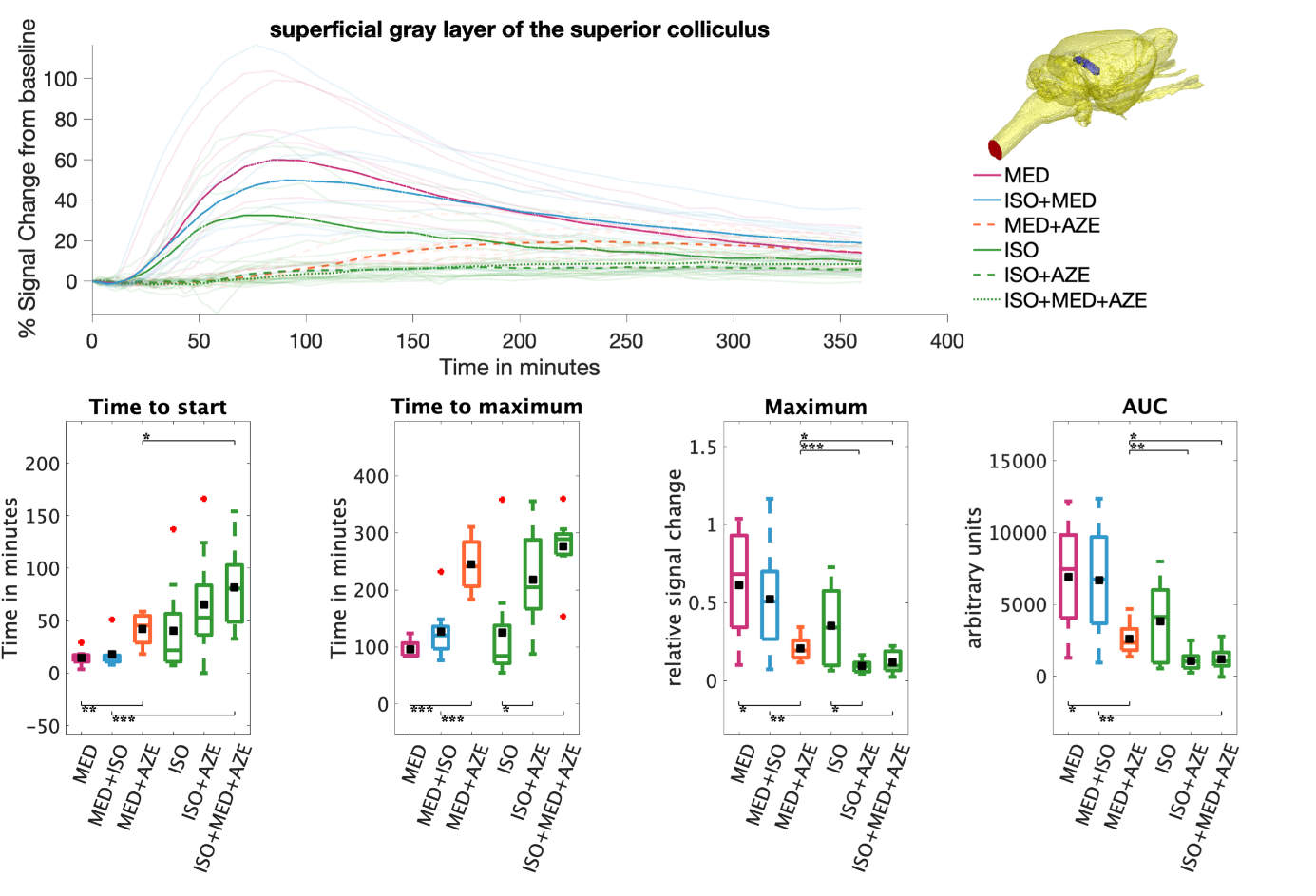

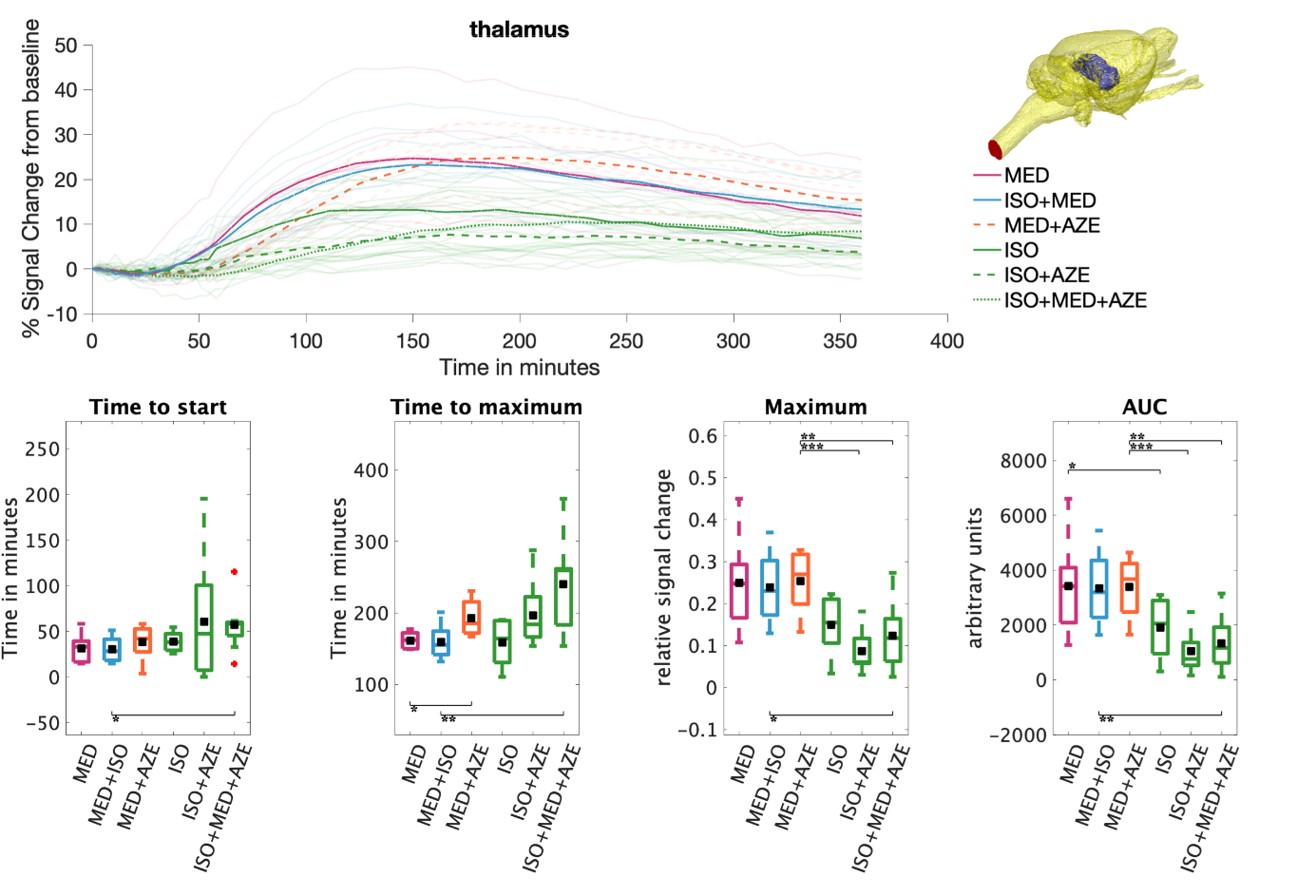


Other structures
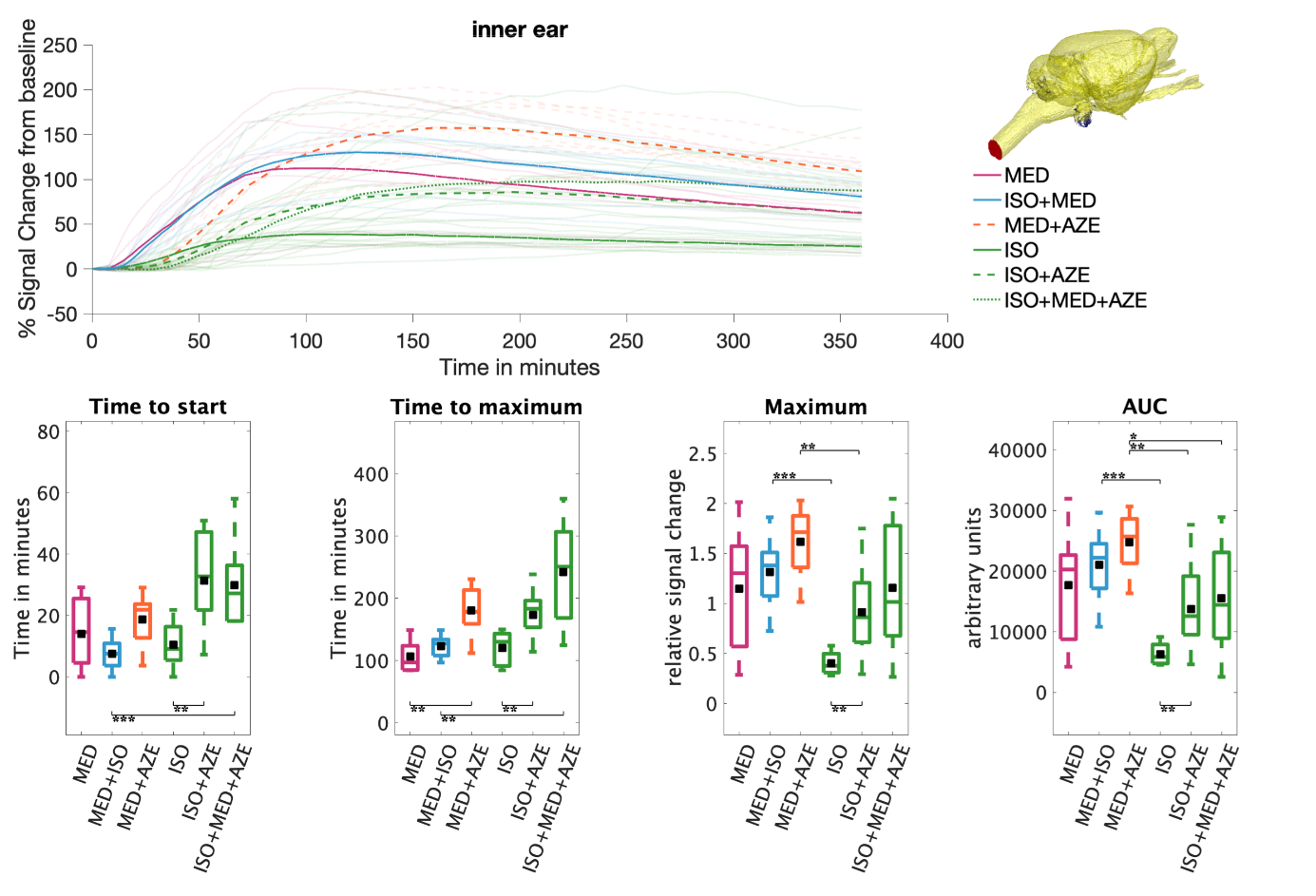

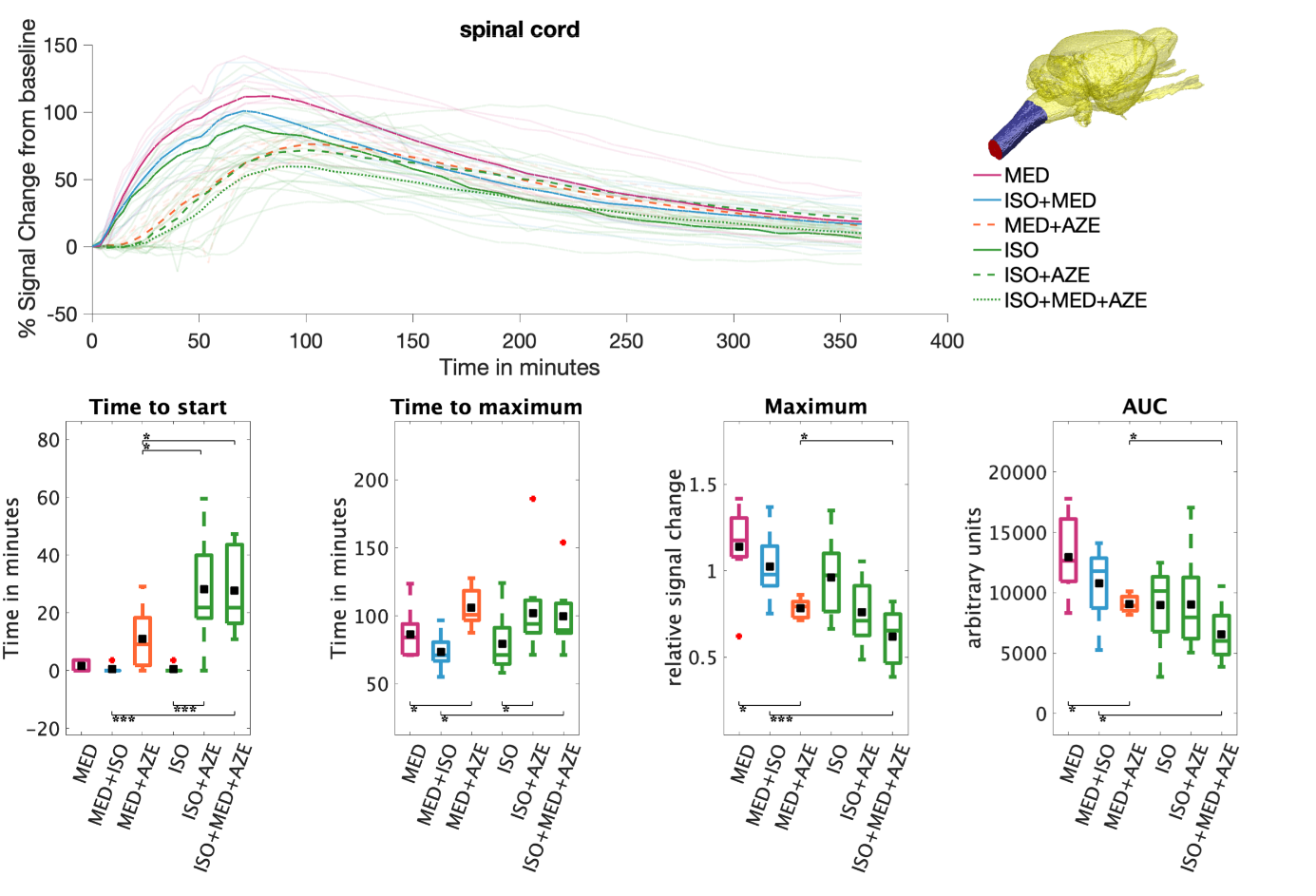

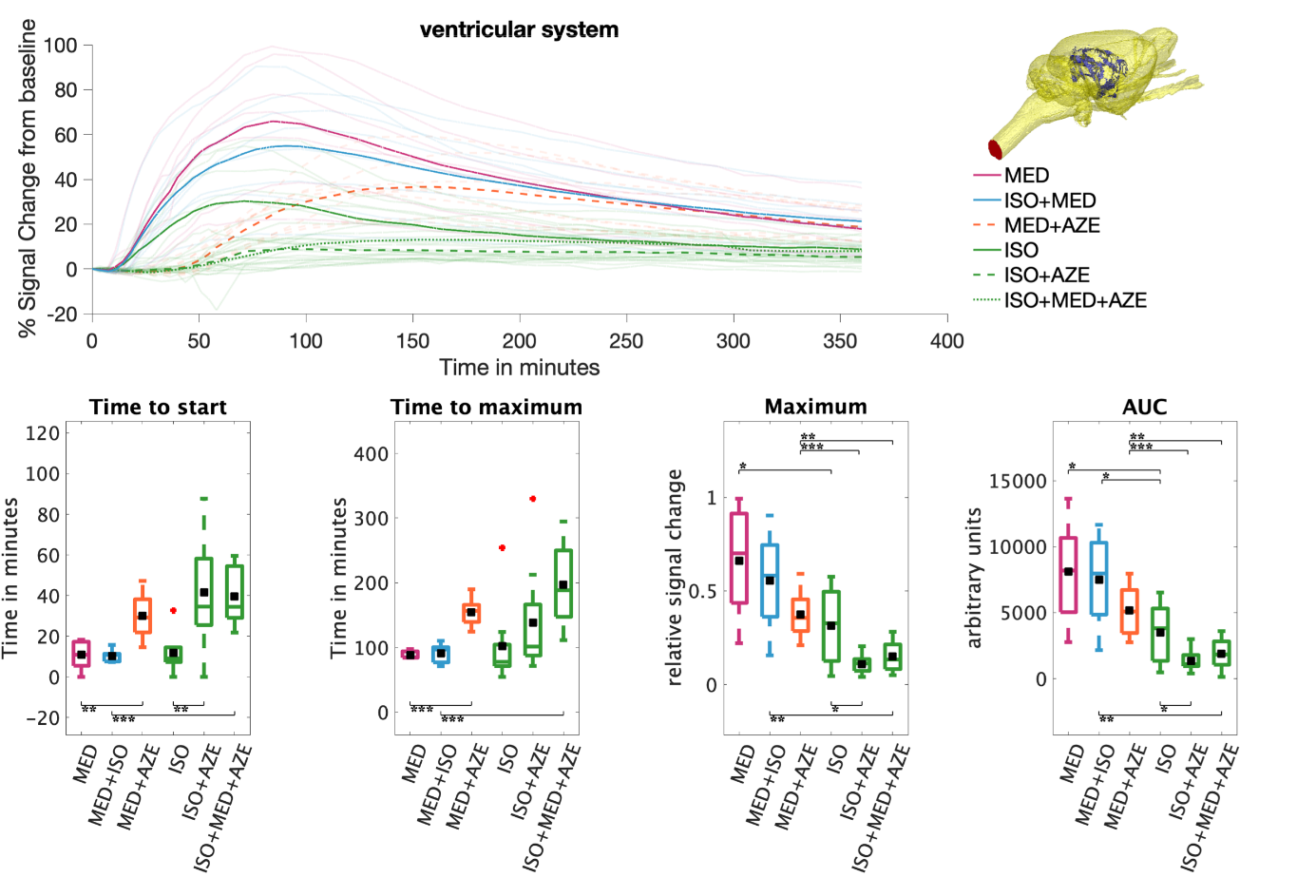


Additional file 3 – Brain region specific analysis

Illustrations of time signal curves, and boxplots for time to start, time to maximum, maximal signal and area under the curve for every anesthetic condition and every of 79 analyzed brain regions. The specific brain region is the respective title of the illustration and is illustrated in the 3D rendering. Time signal curves are represented in percent signal change from baseline and time in minutes for each anesthetic condition and each animal (thin curves) and mean (thick curves) (ISO n = 8; MED n = 7; ISO+MED n = 8; ISO+AZE n = 10; MED+AZE n = 8; ISO+MED+AZE n = 8). Concerning the Boxplots the central mark indicates the median, the bottom and top edges of the box indicate the 25th and 75th percentiles, respectively. The whiskers extend to the most extreme data points not considered outliers. Significant differences are marked with one star if p < 0.05, two stars if p < 0.01 and three if p < 0.001.

White matter
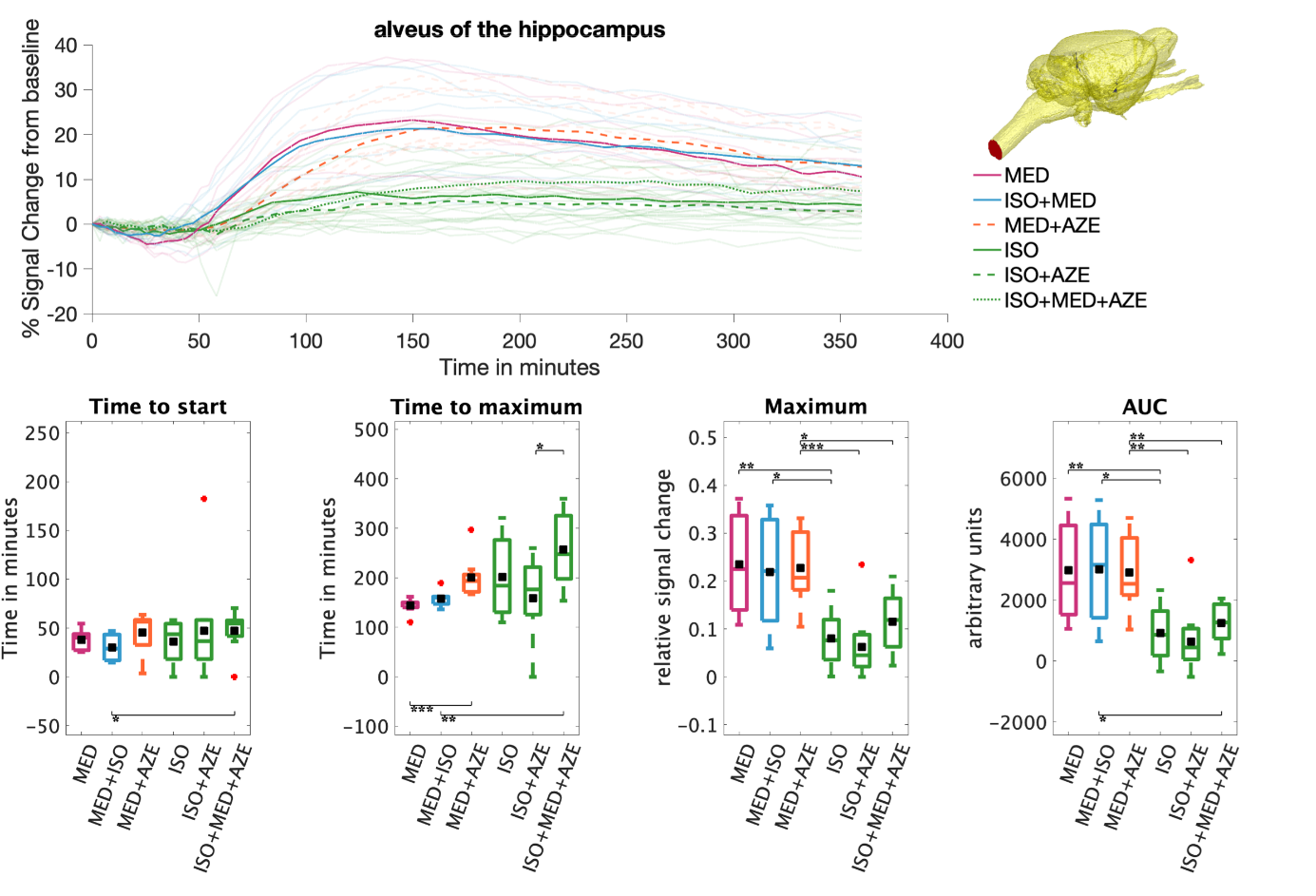

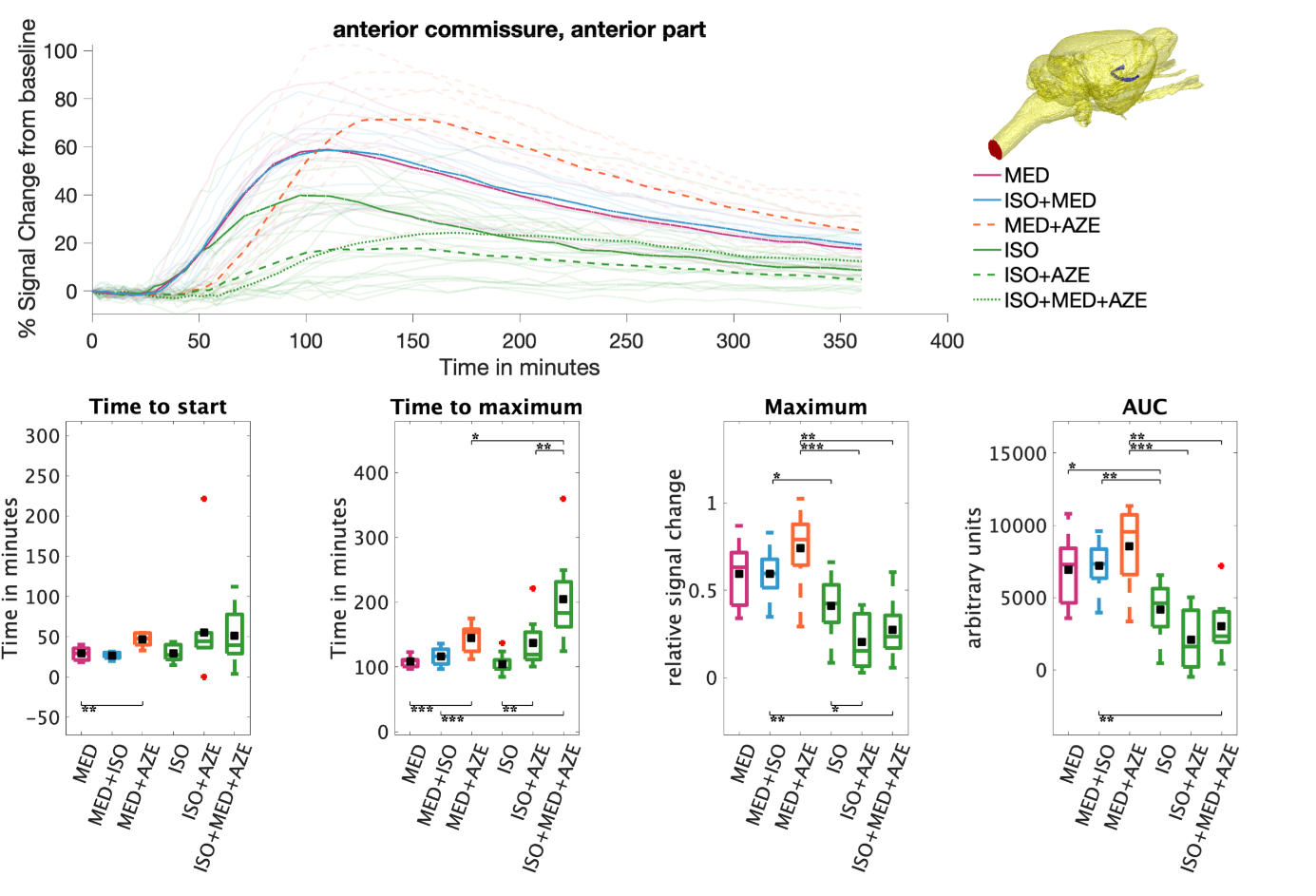

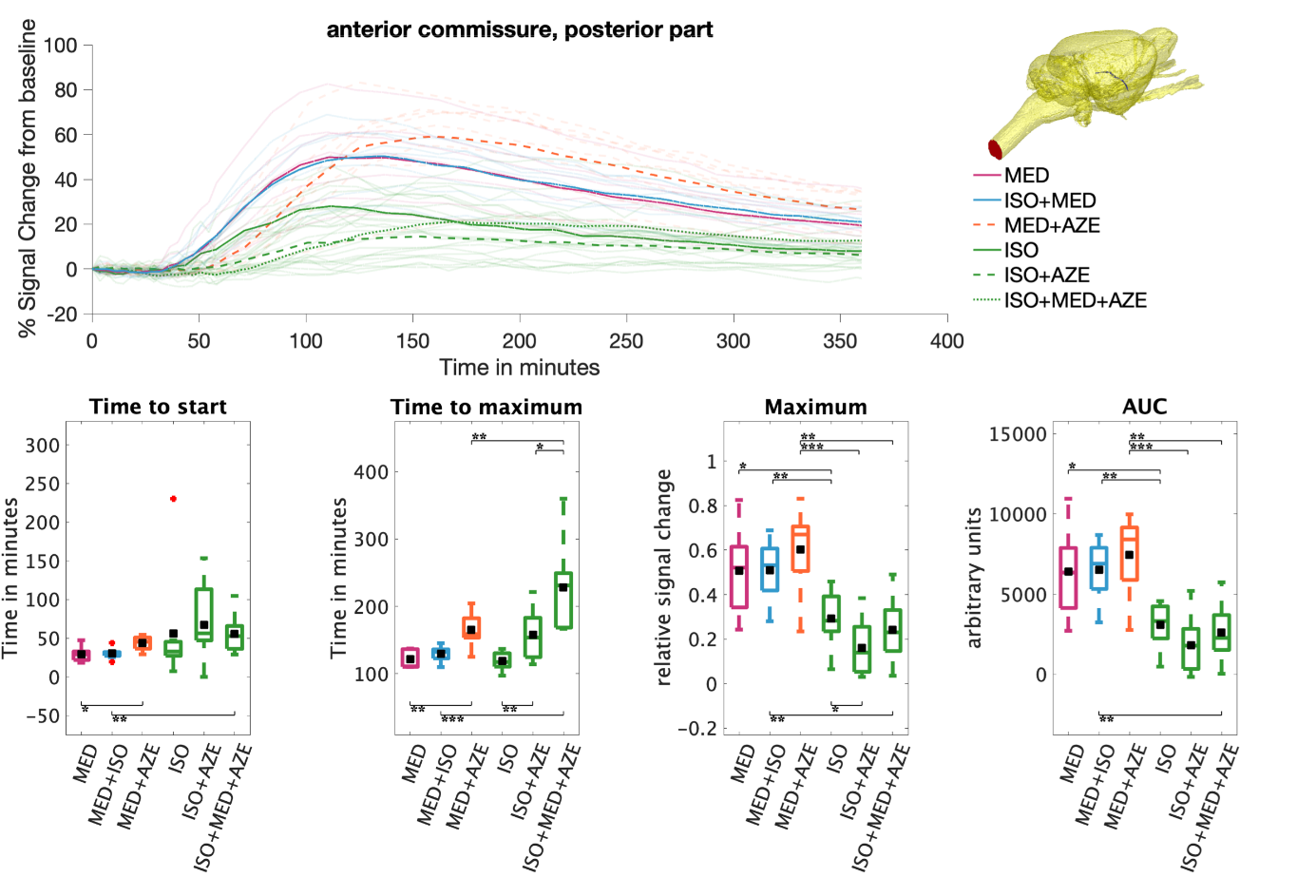

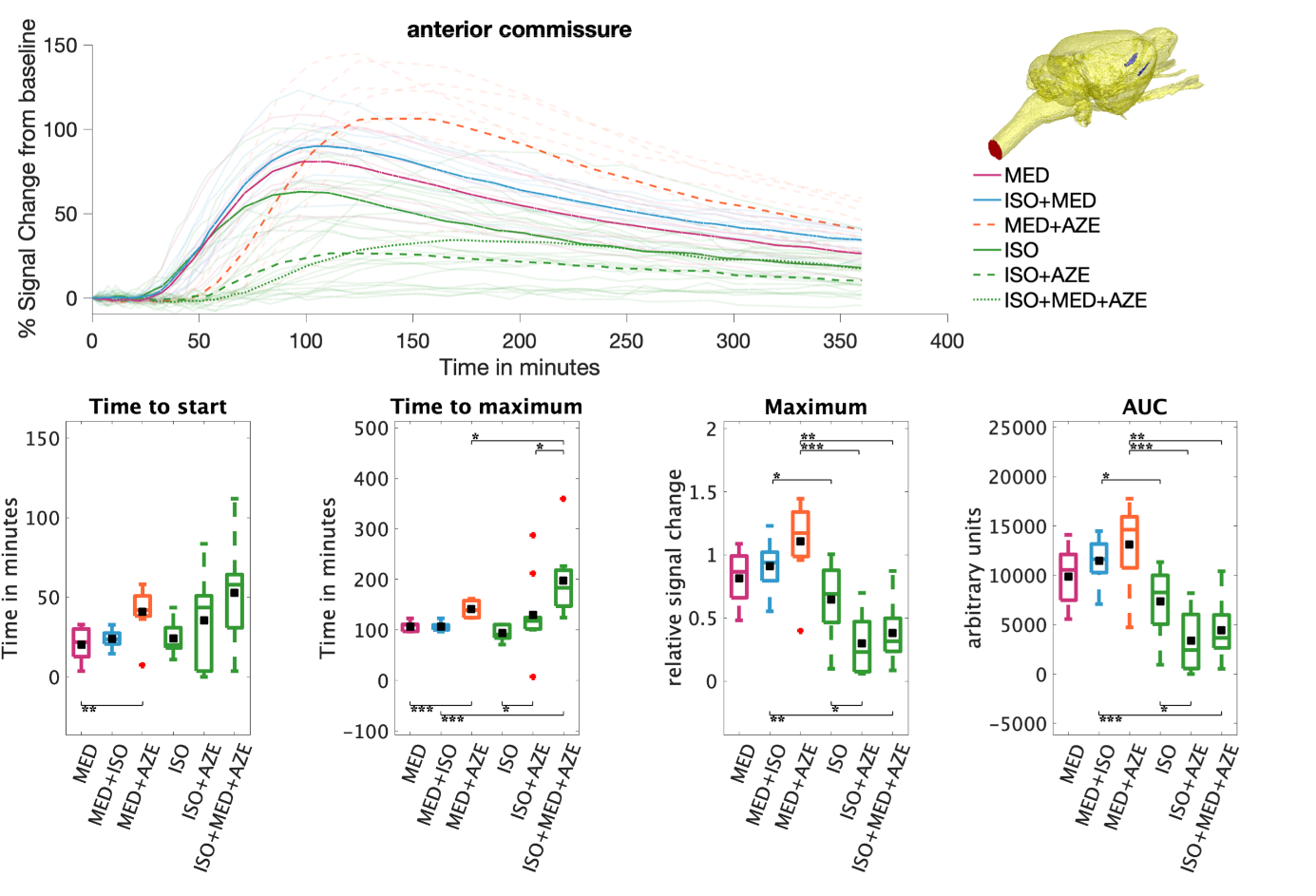

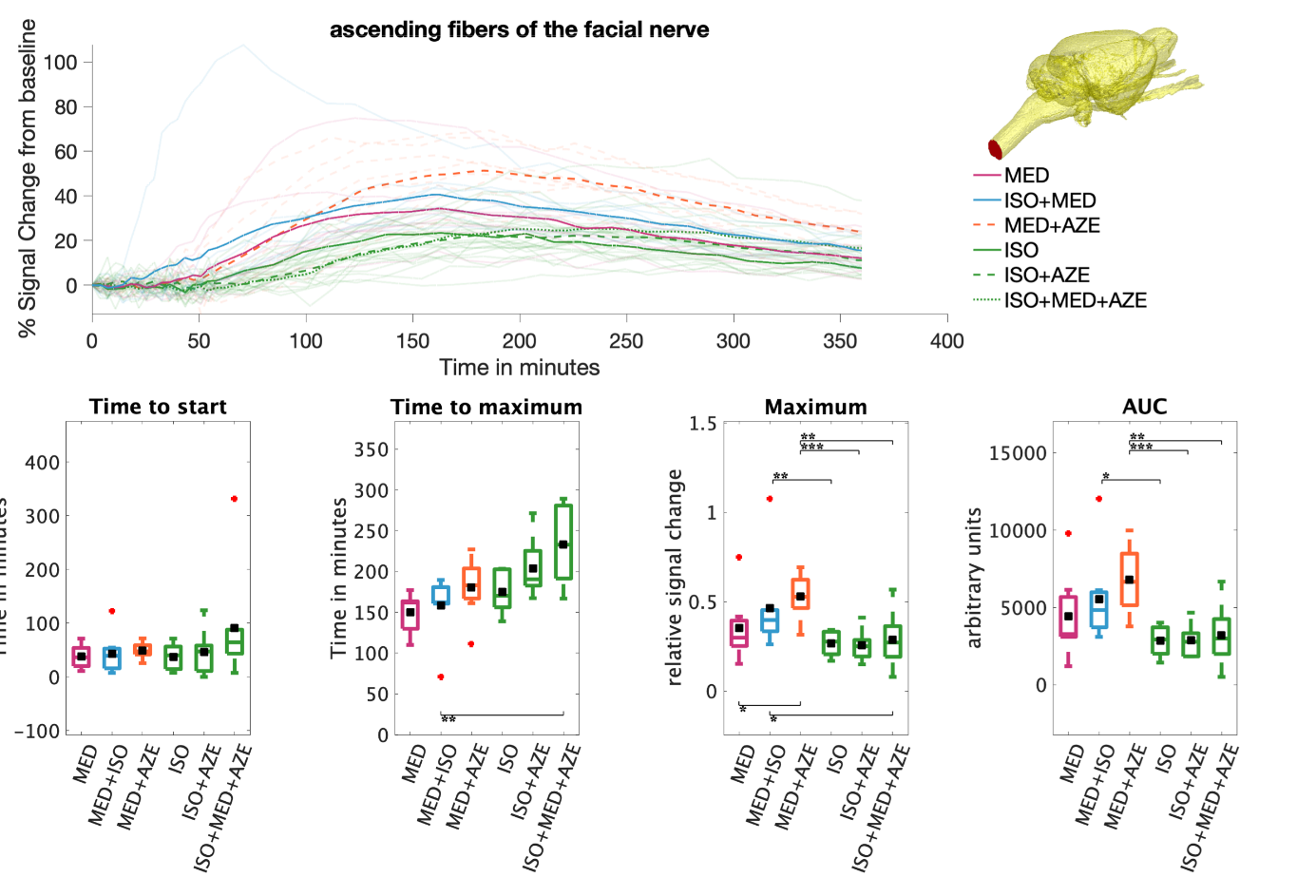

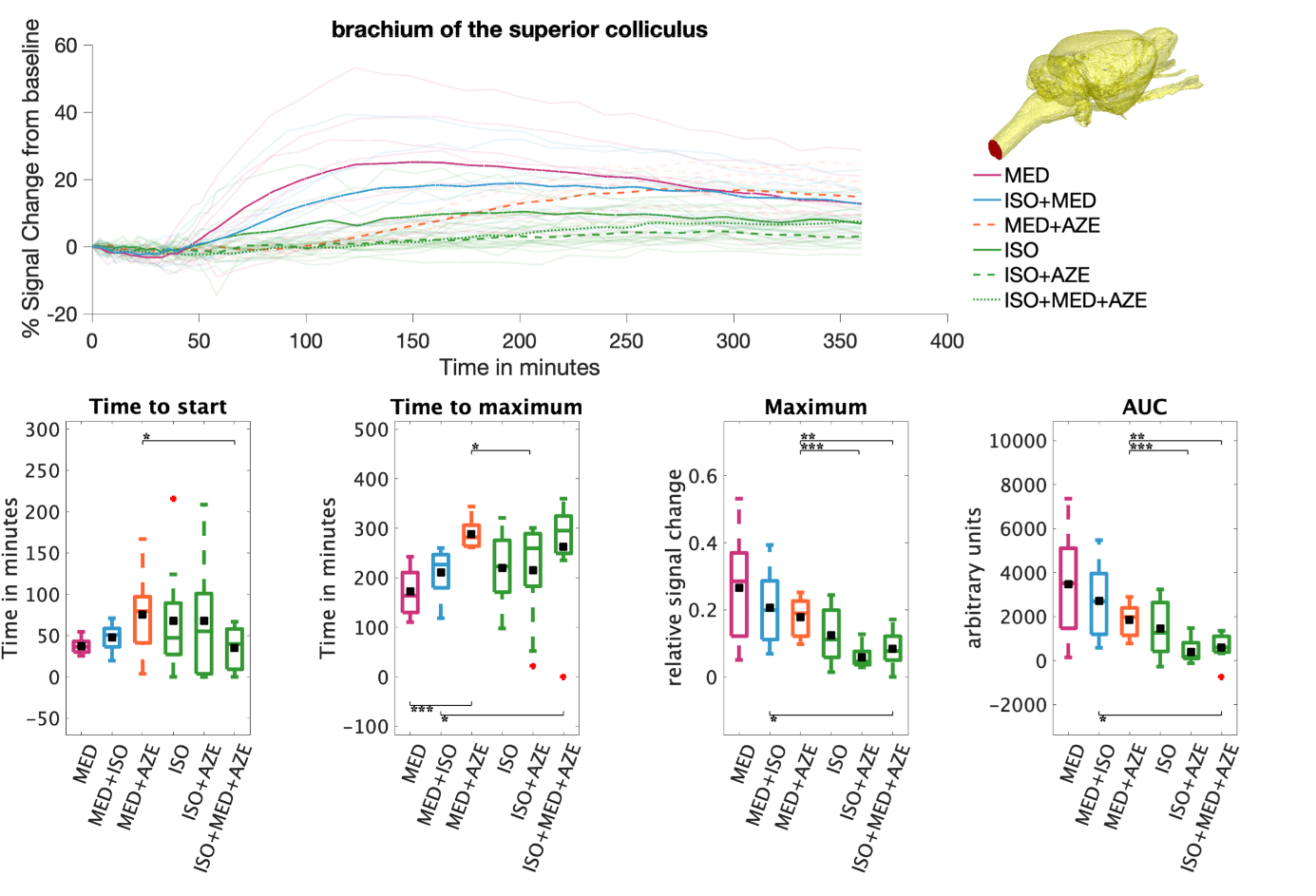

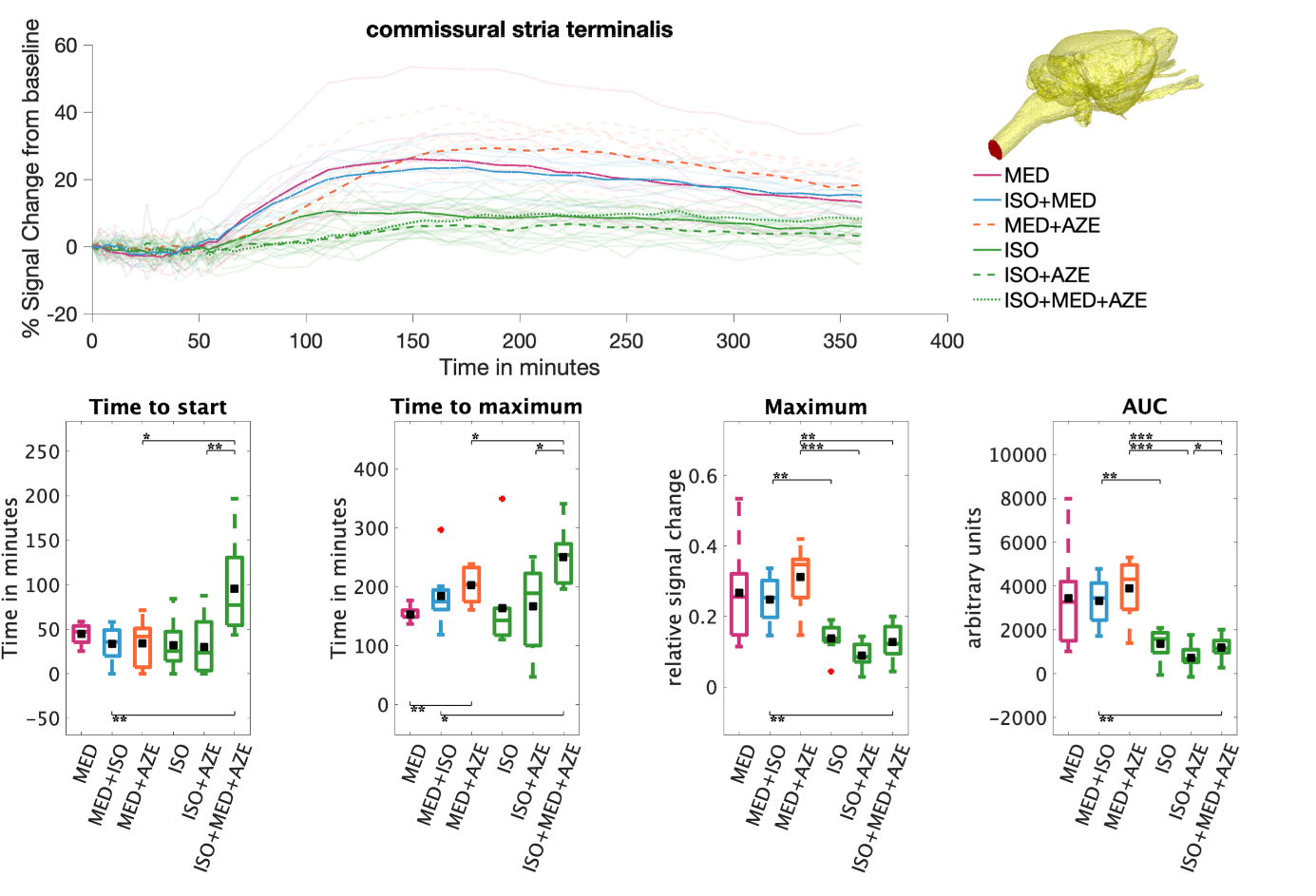

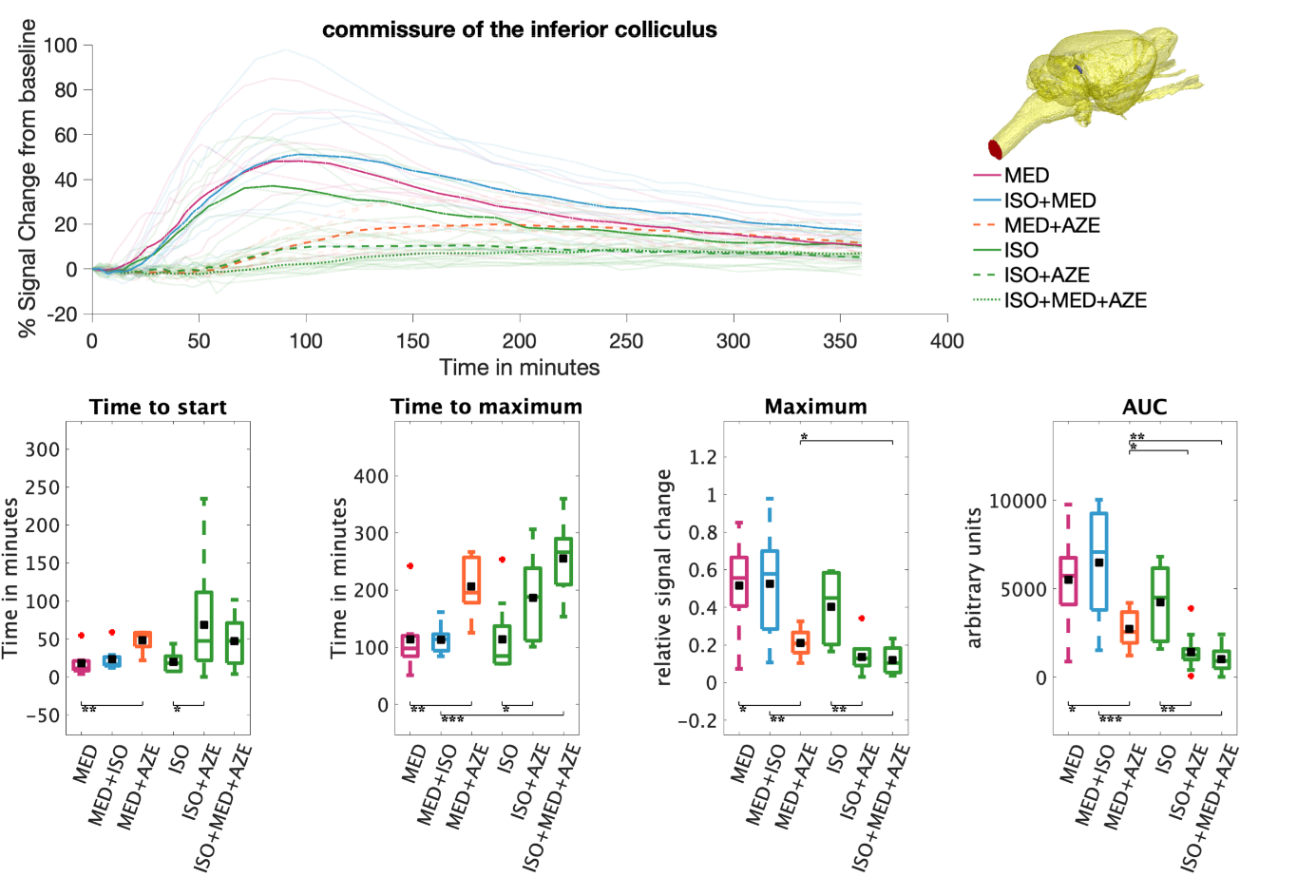

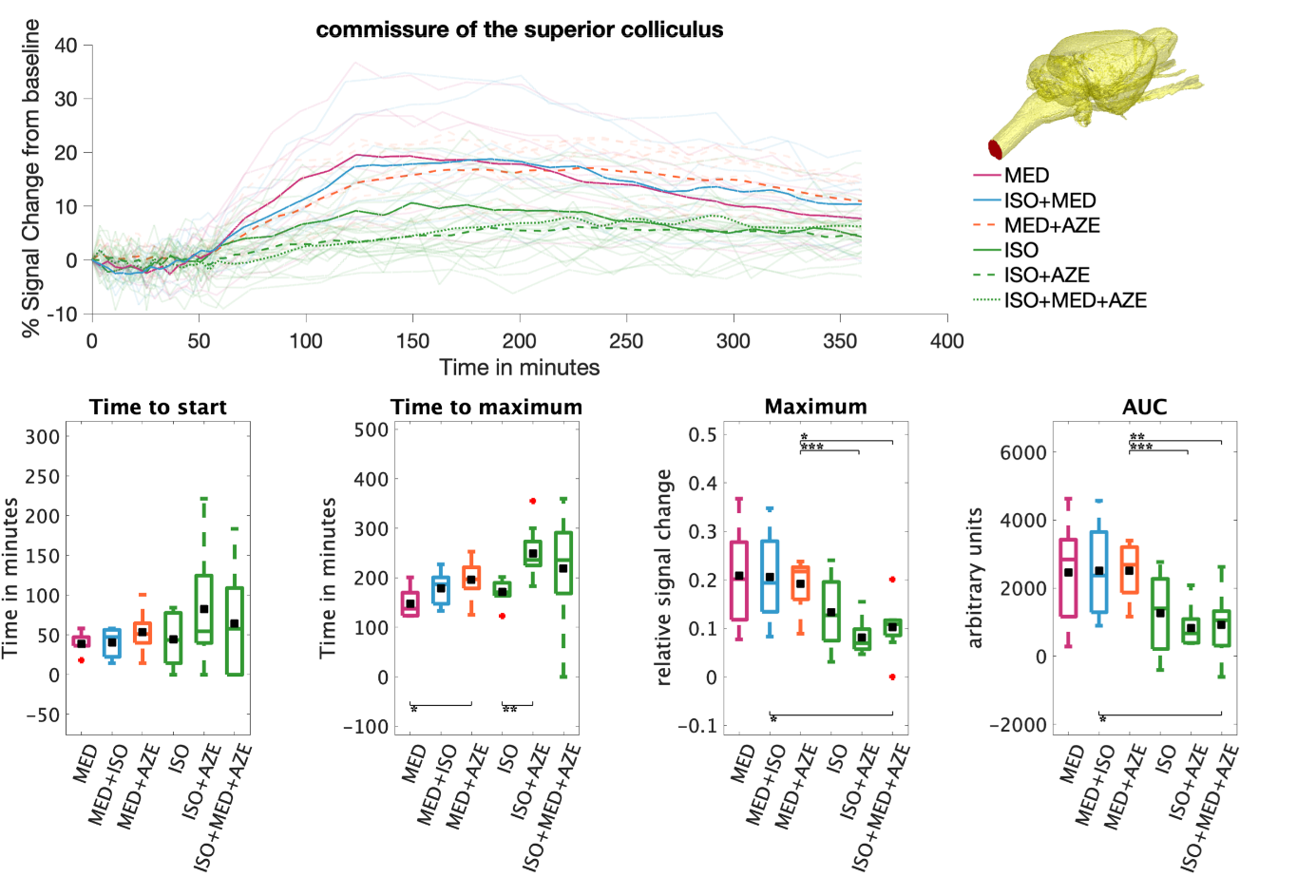

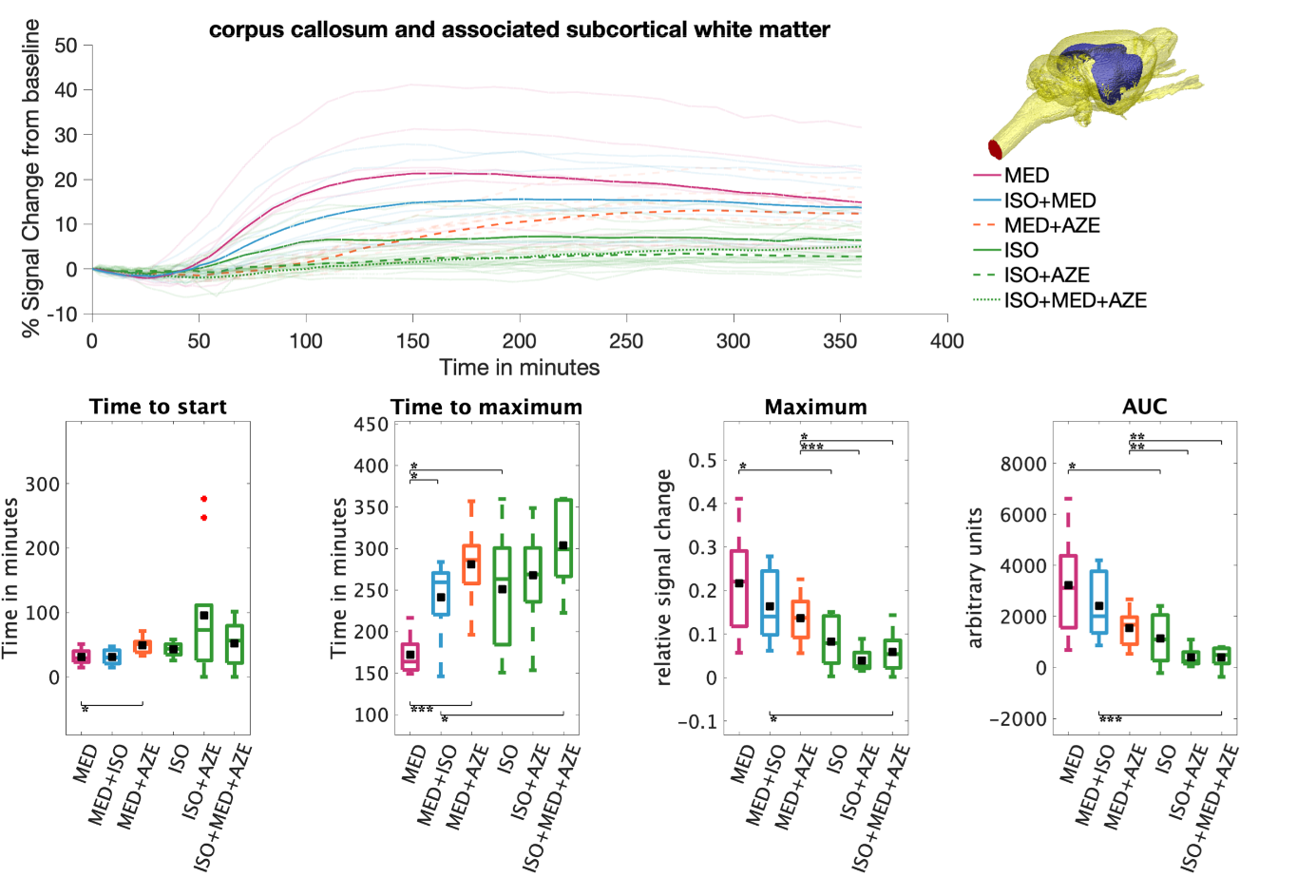

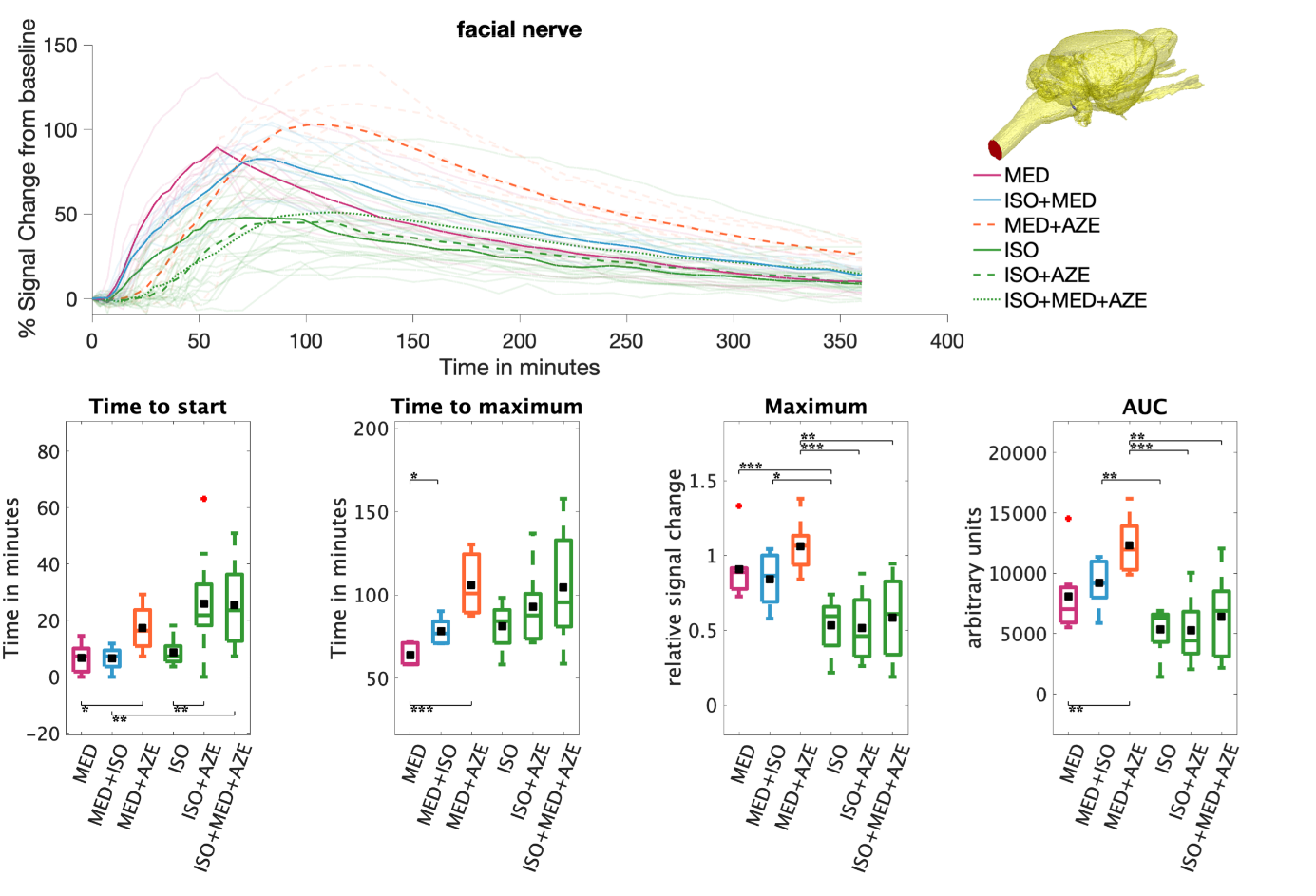

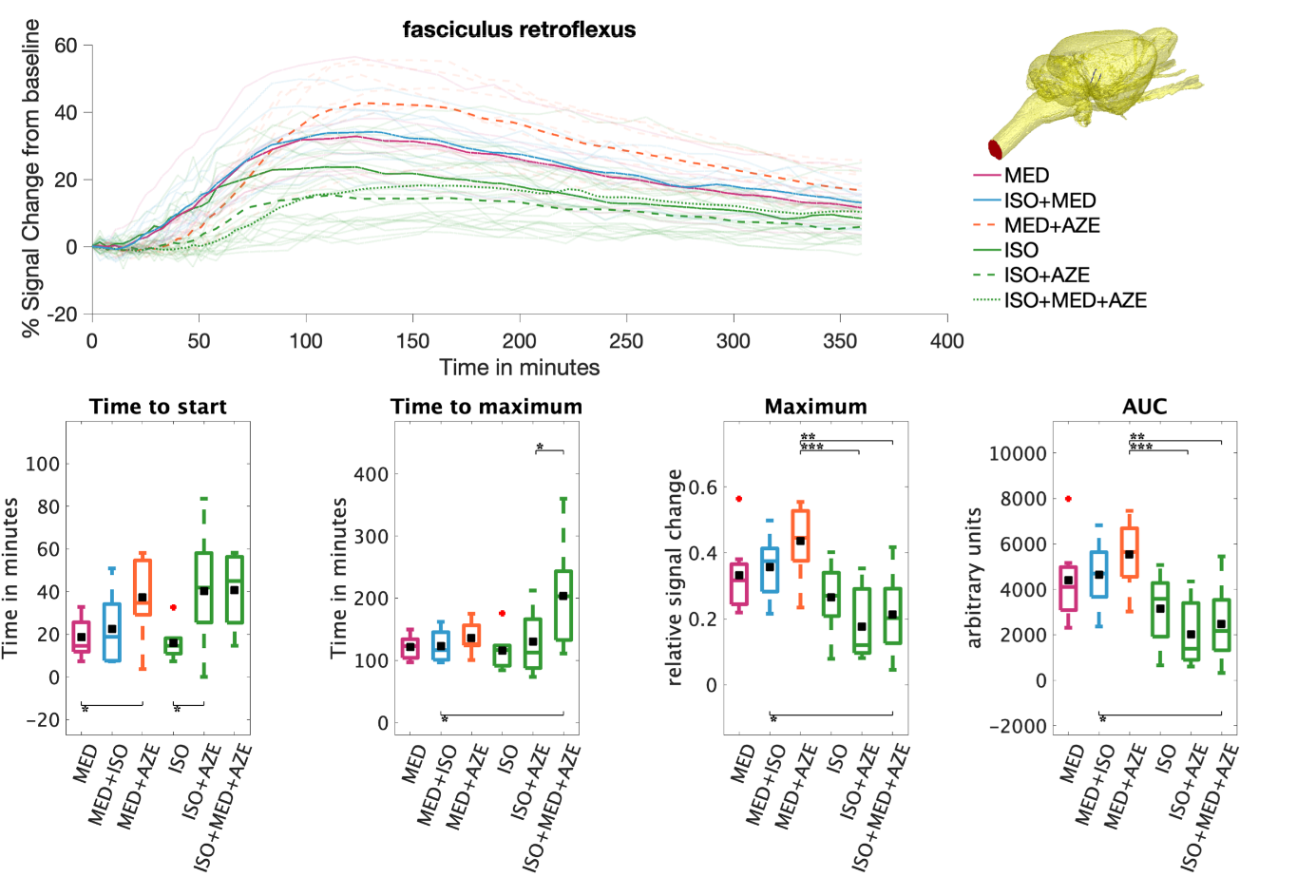

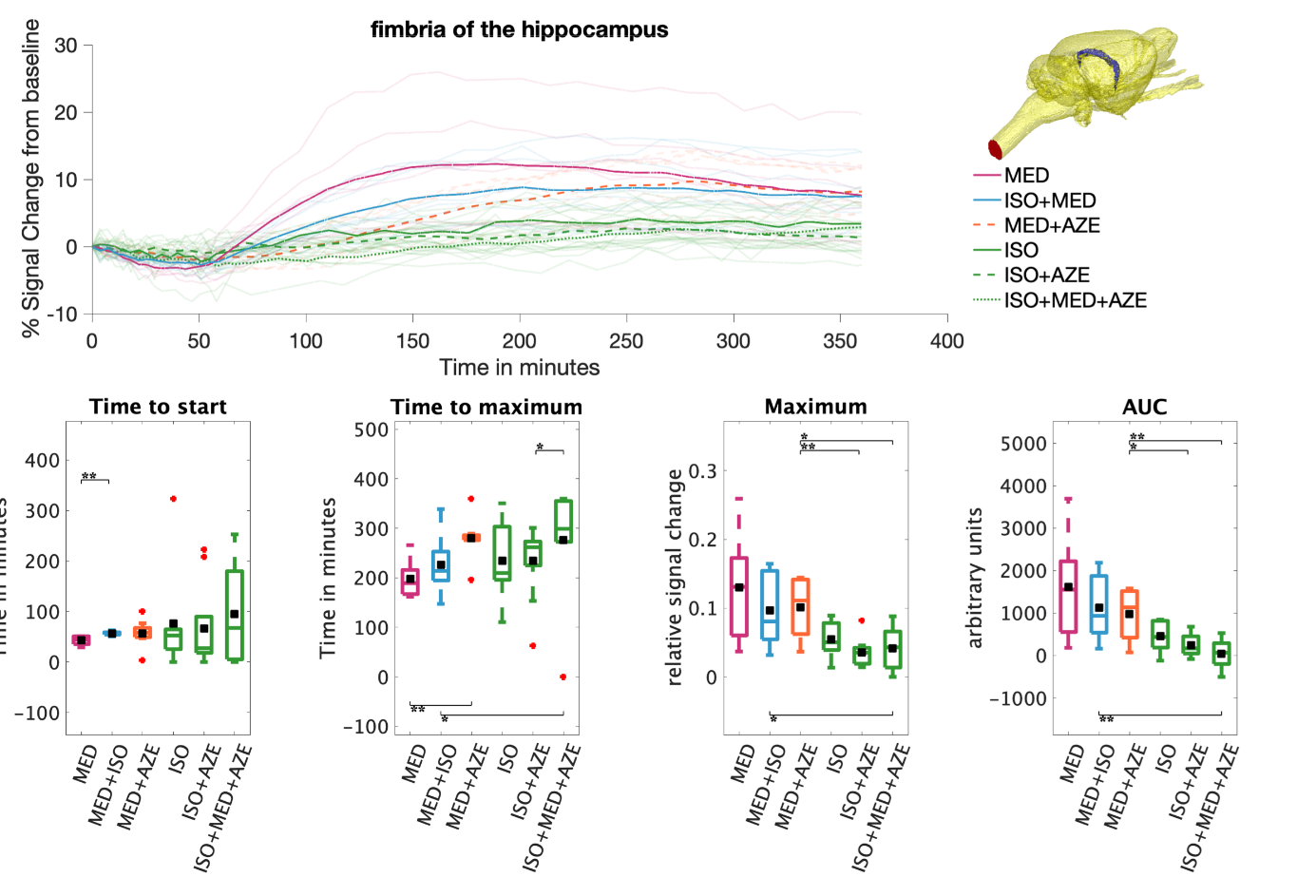

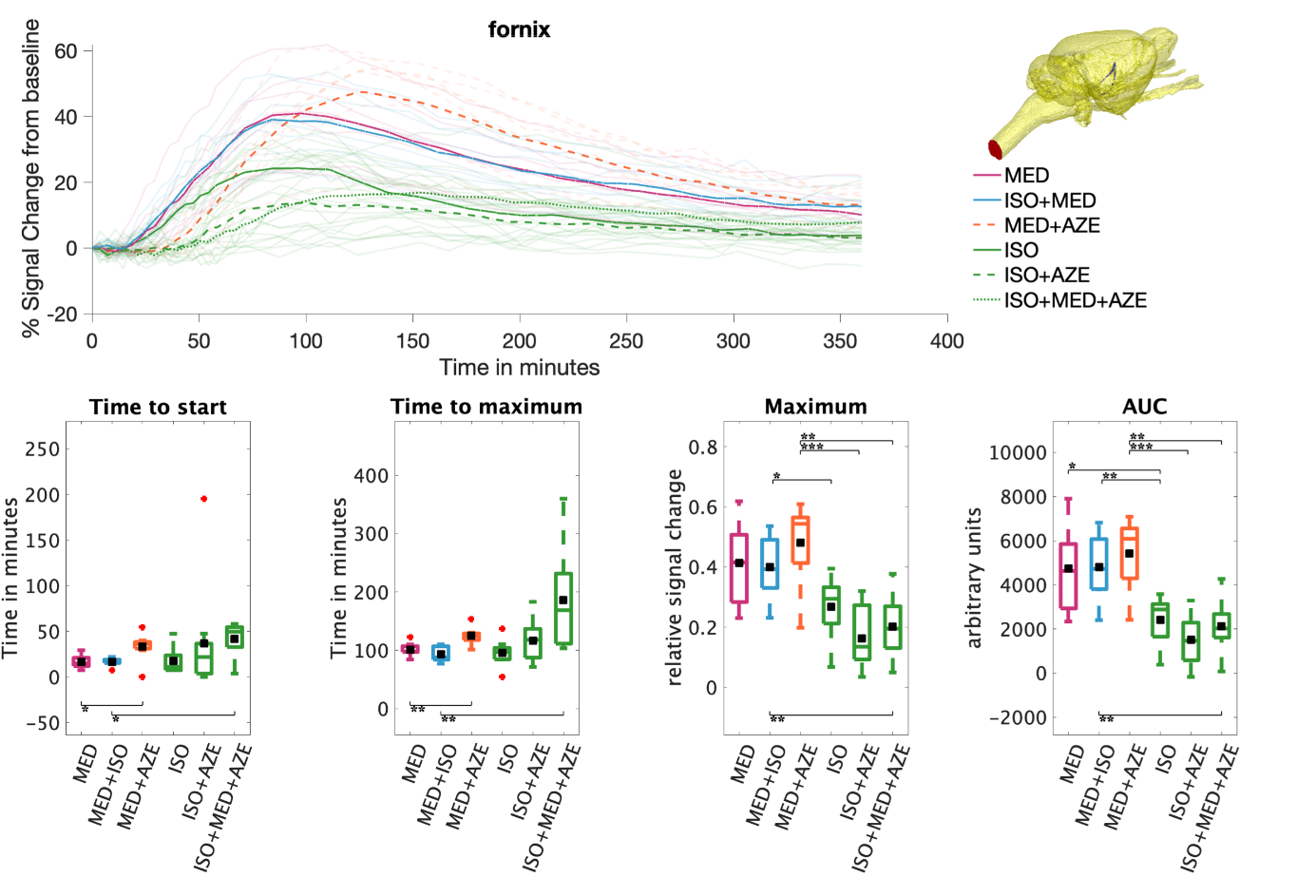

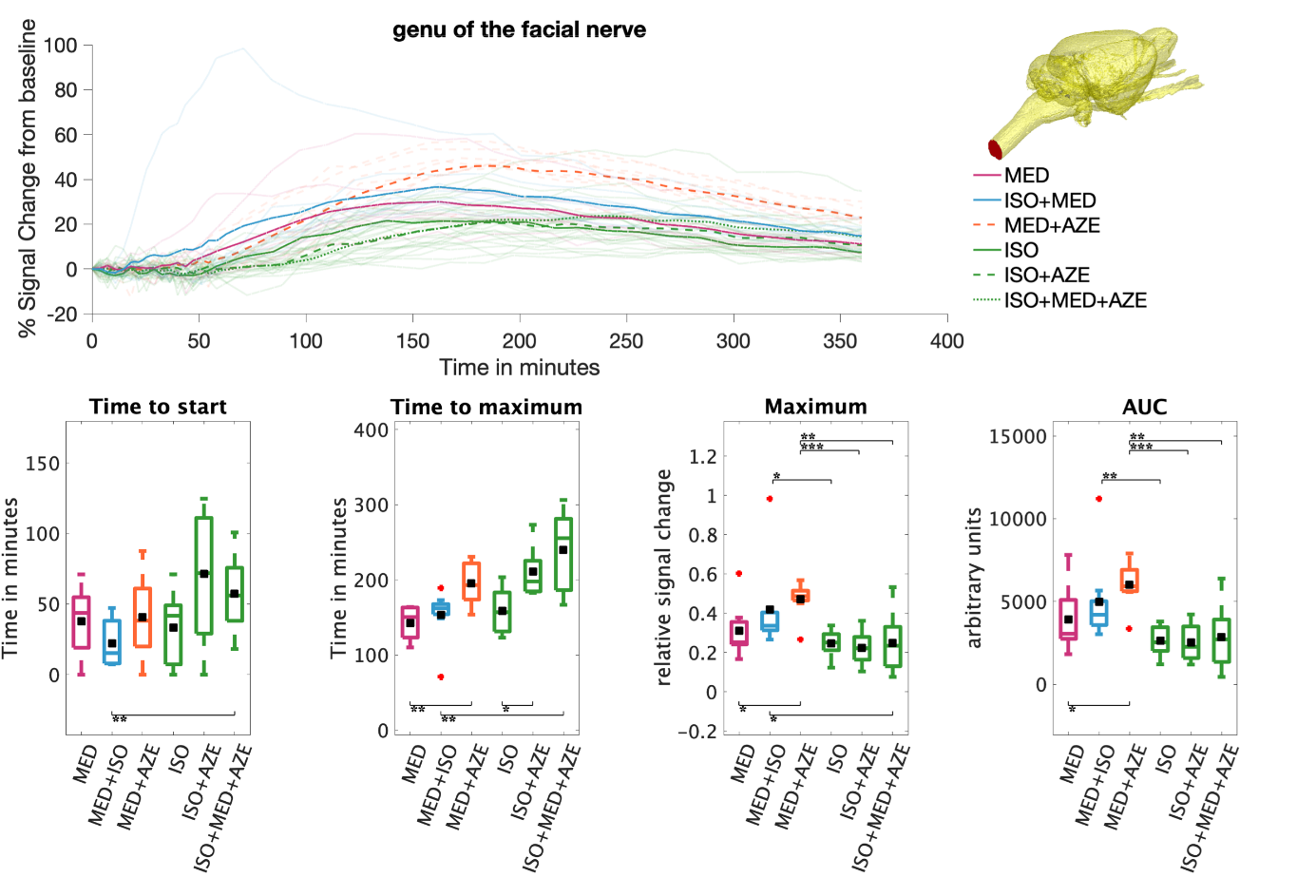

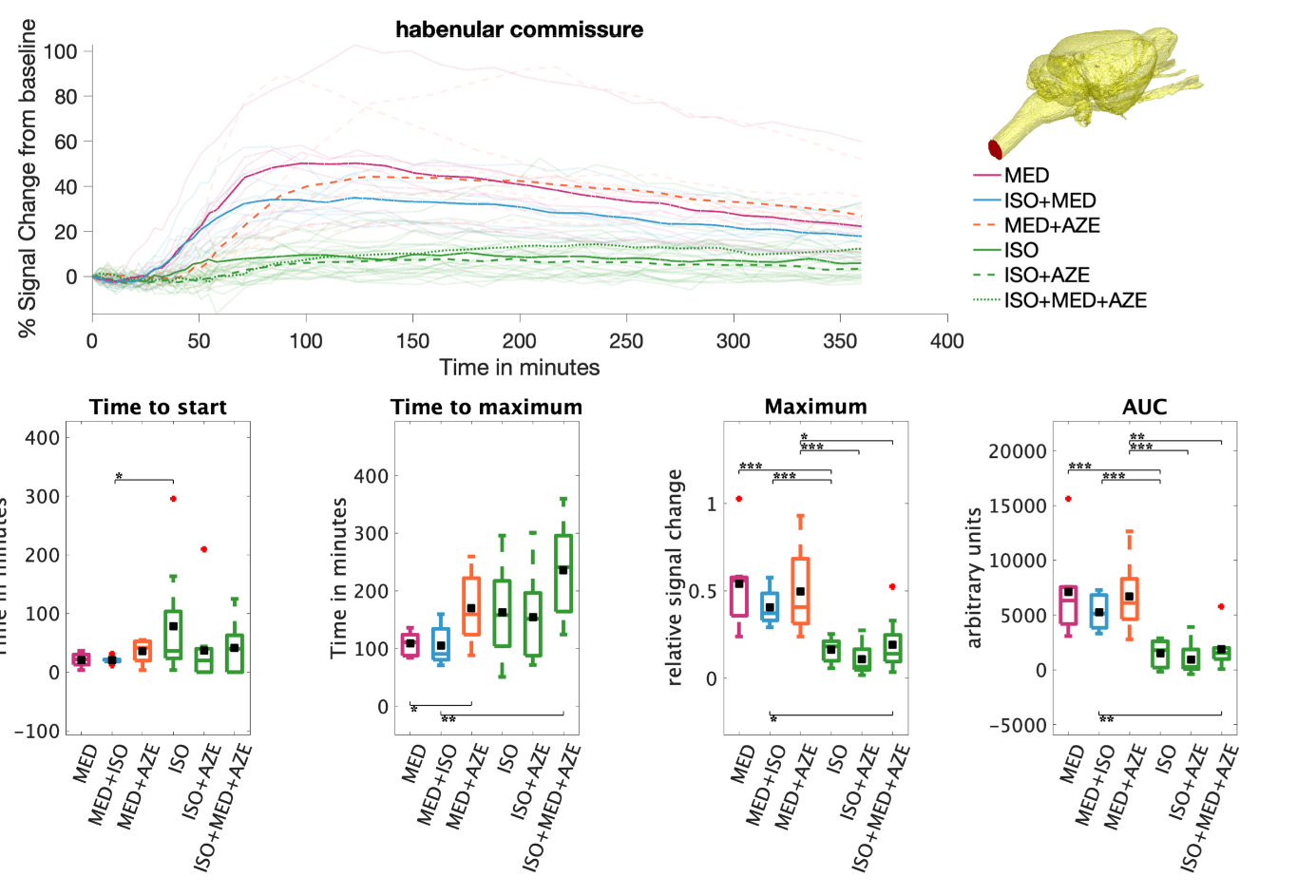

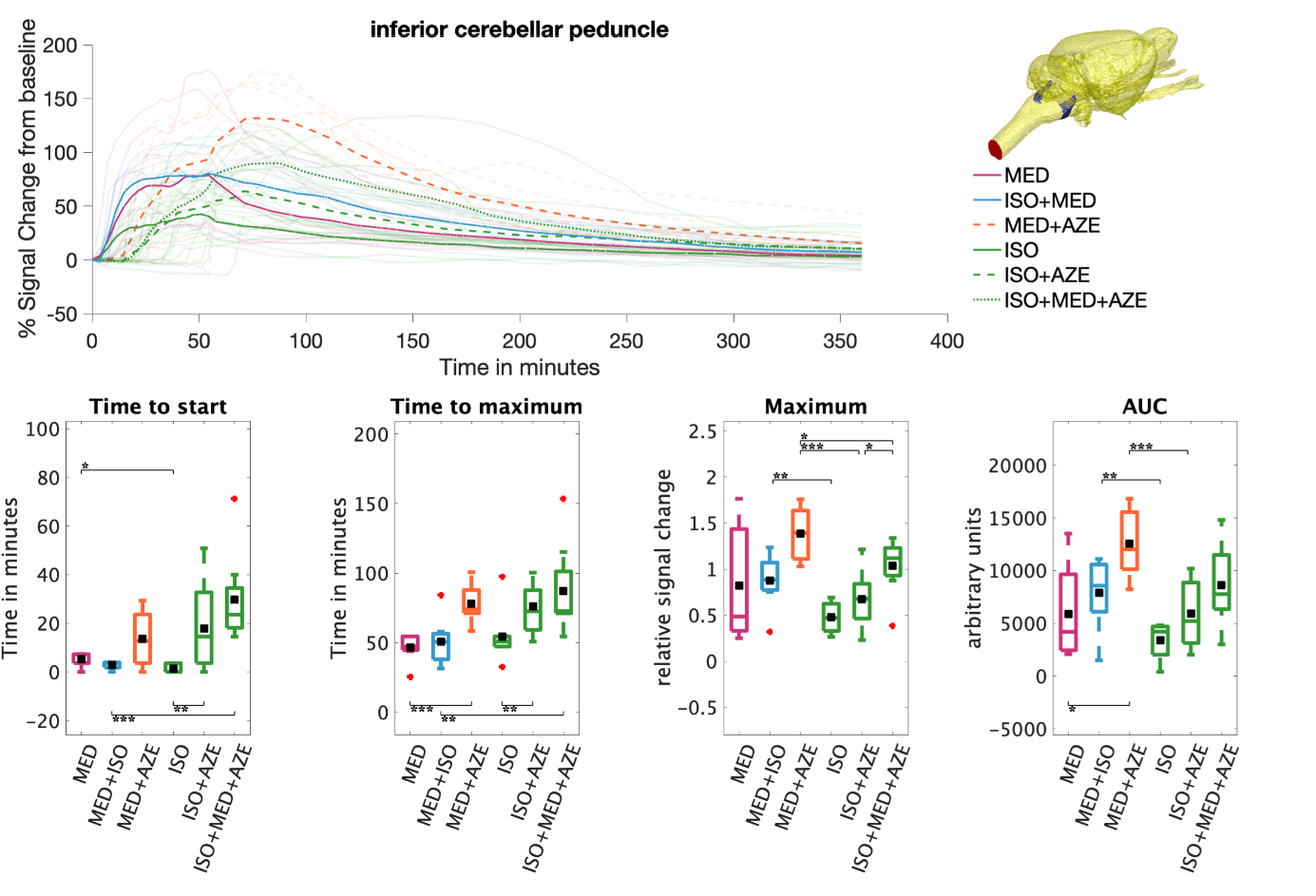

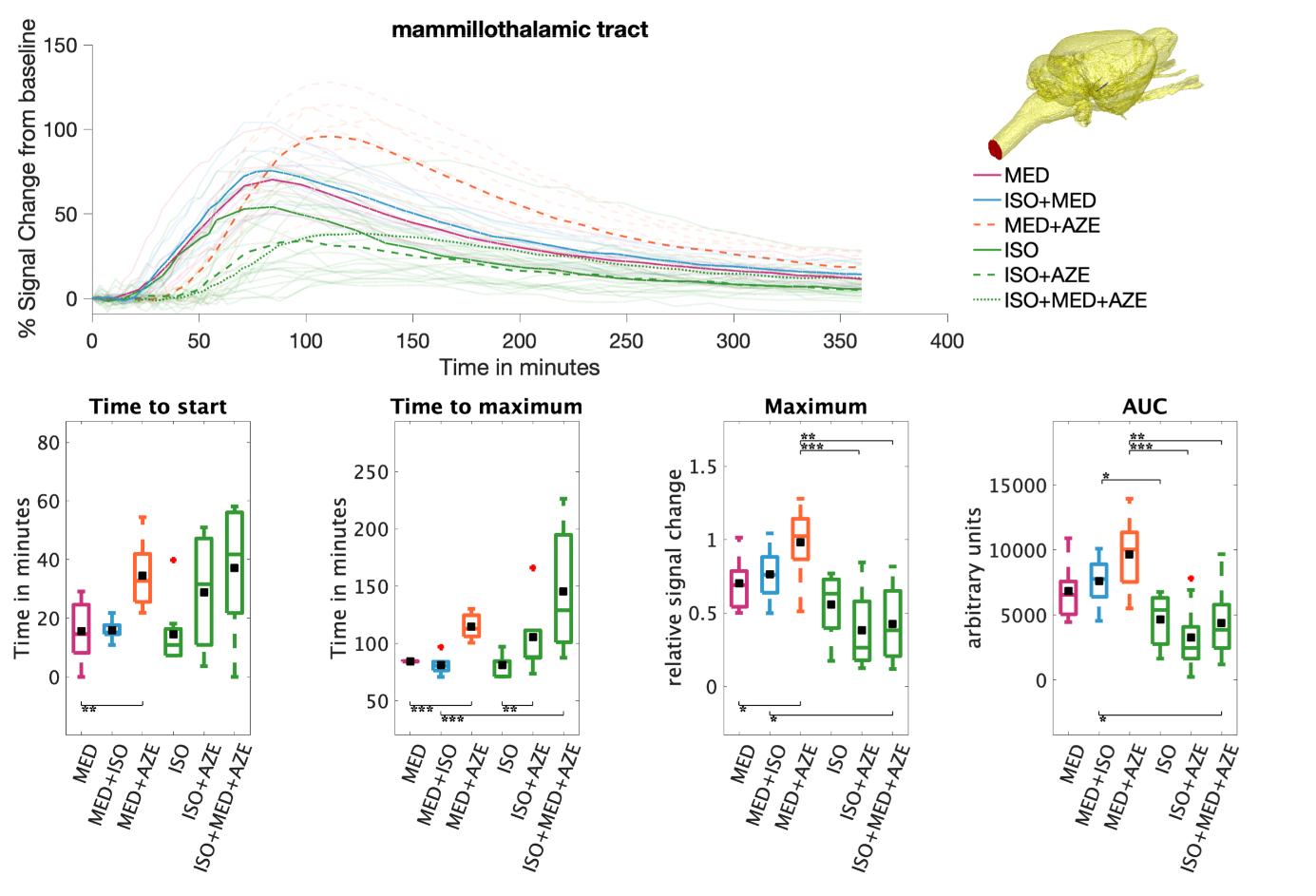

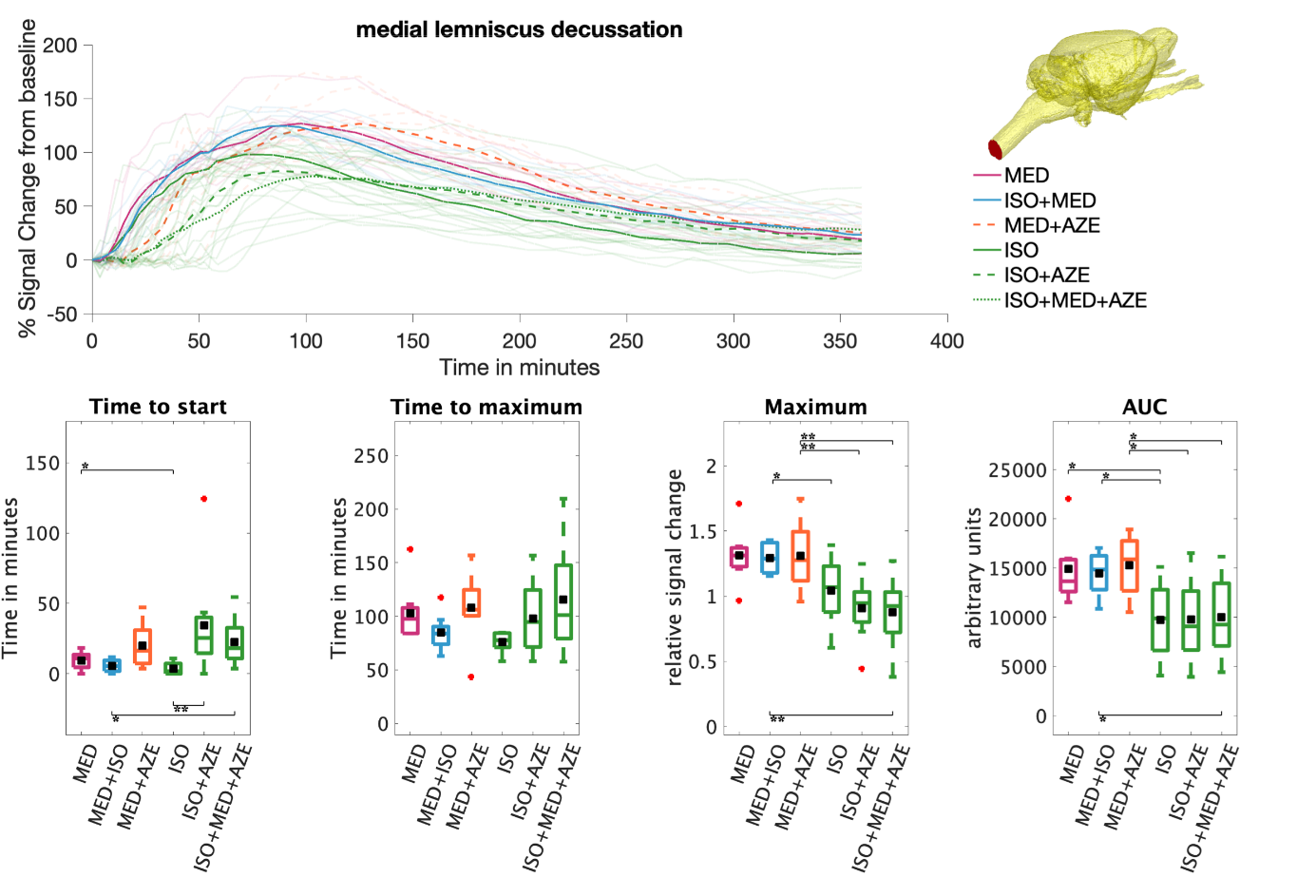

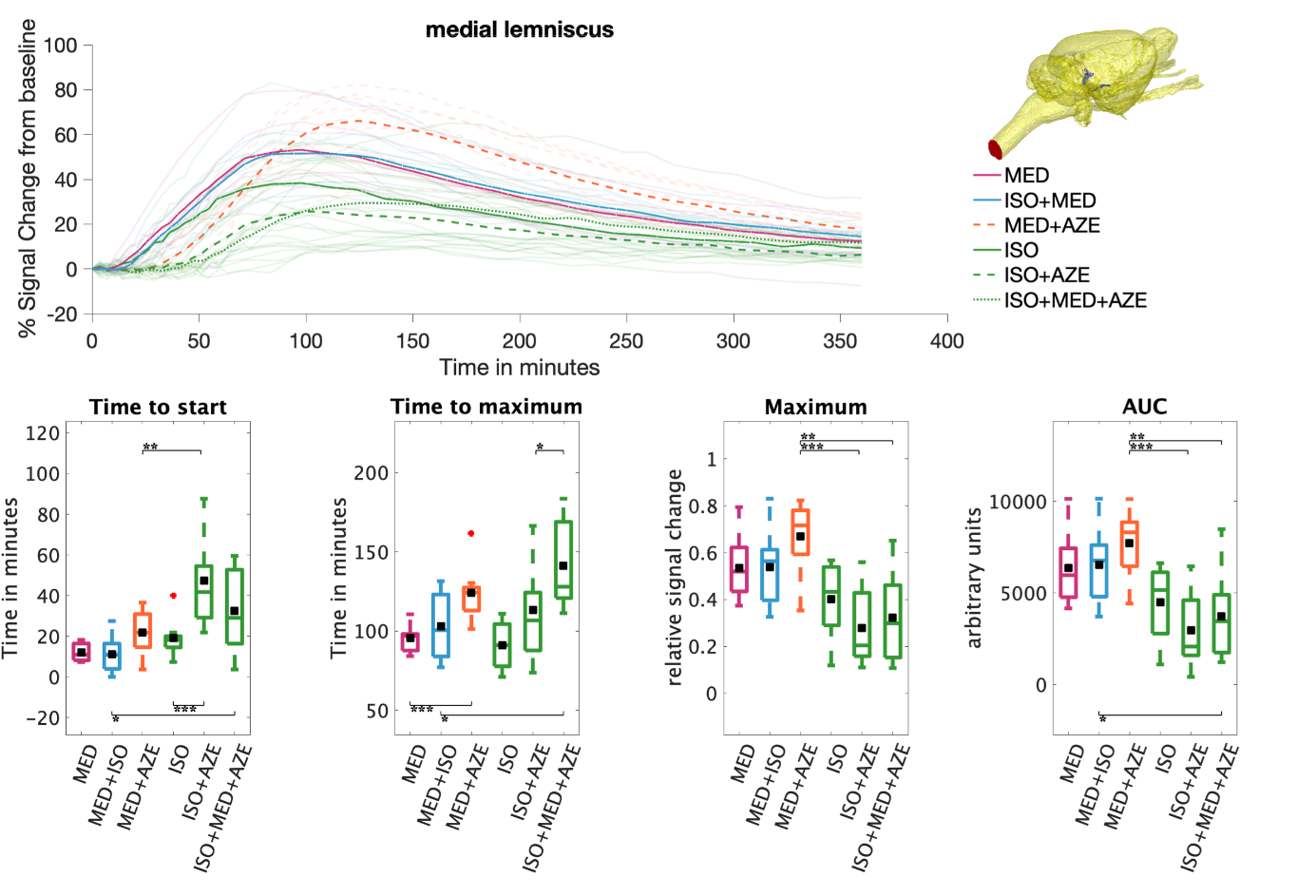

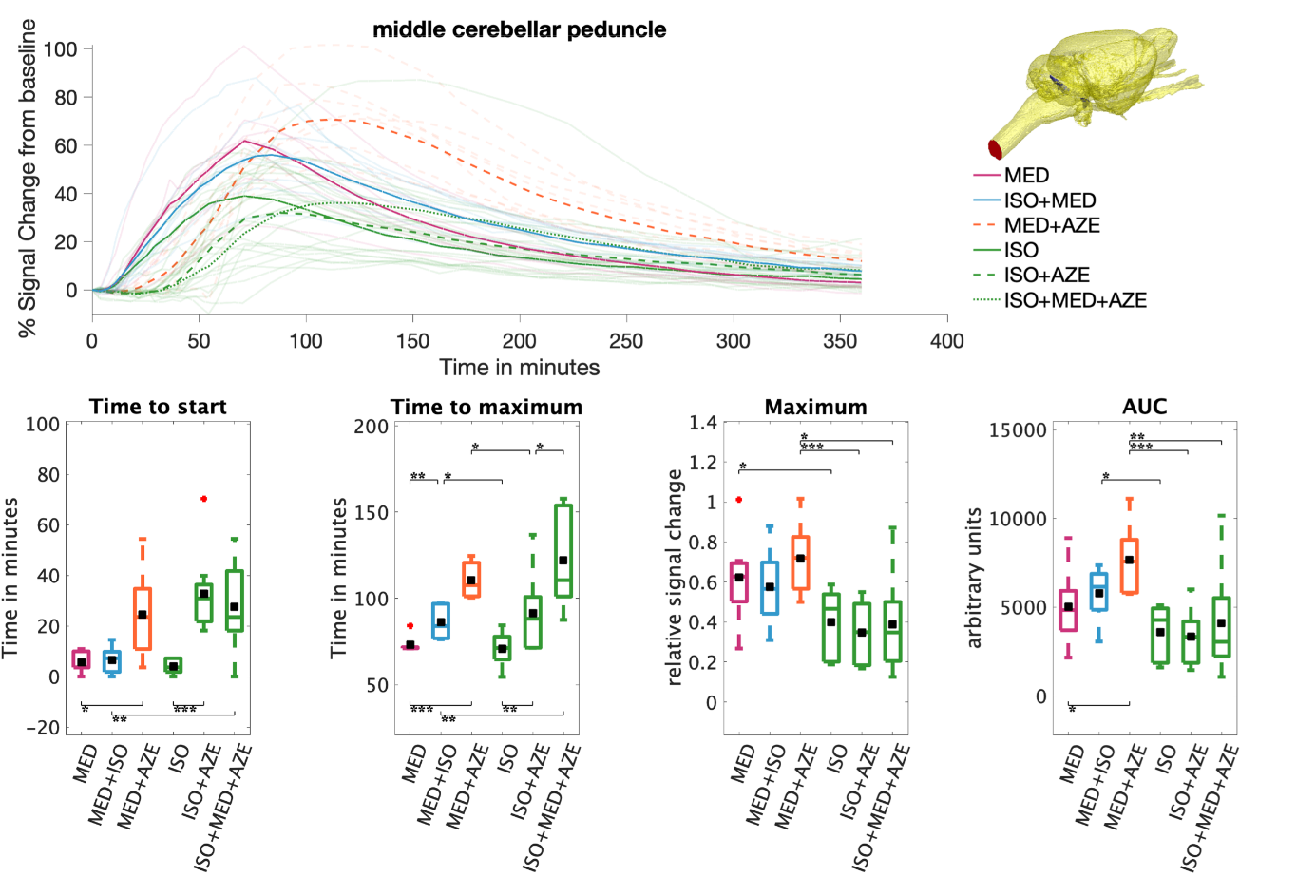

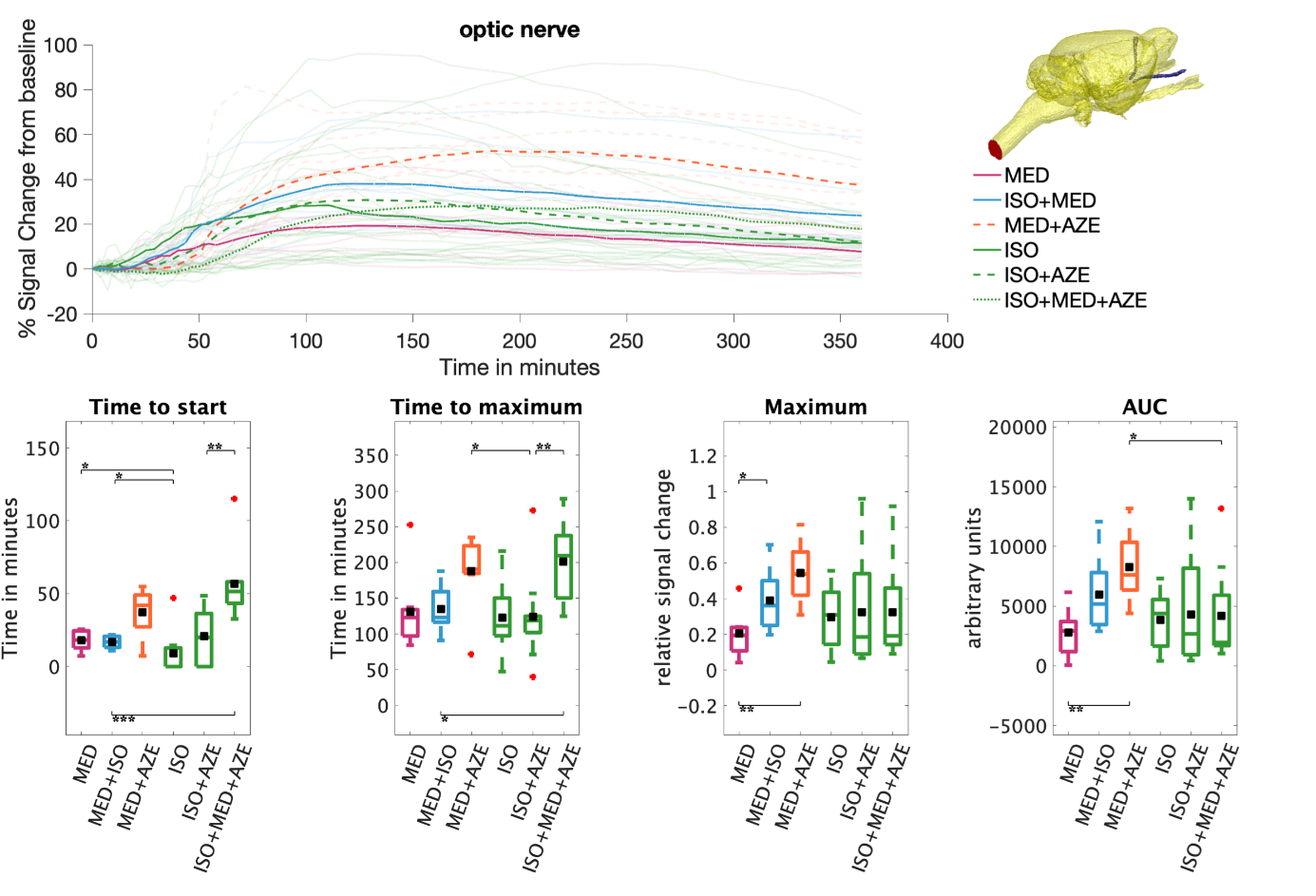

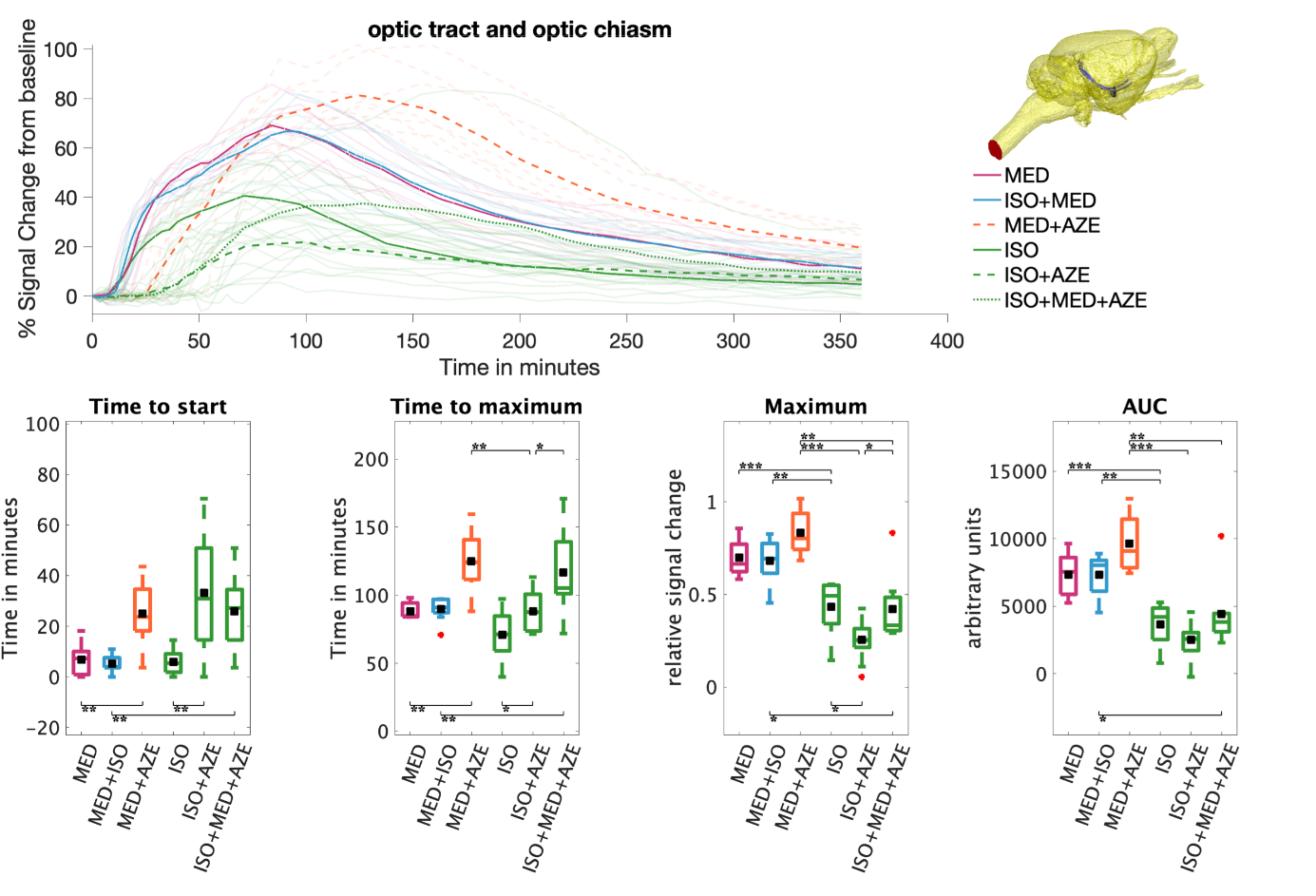

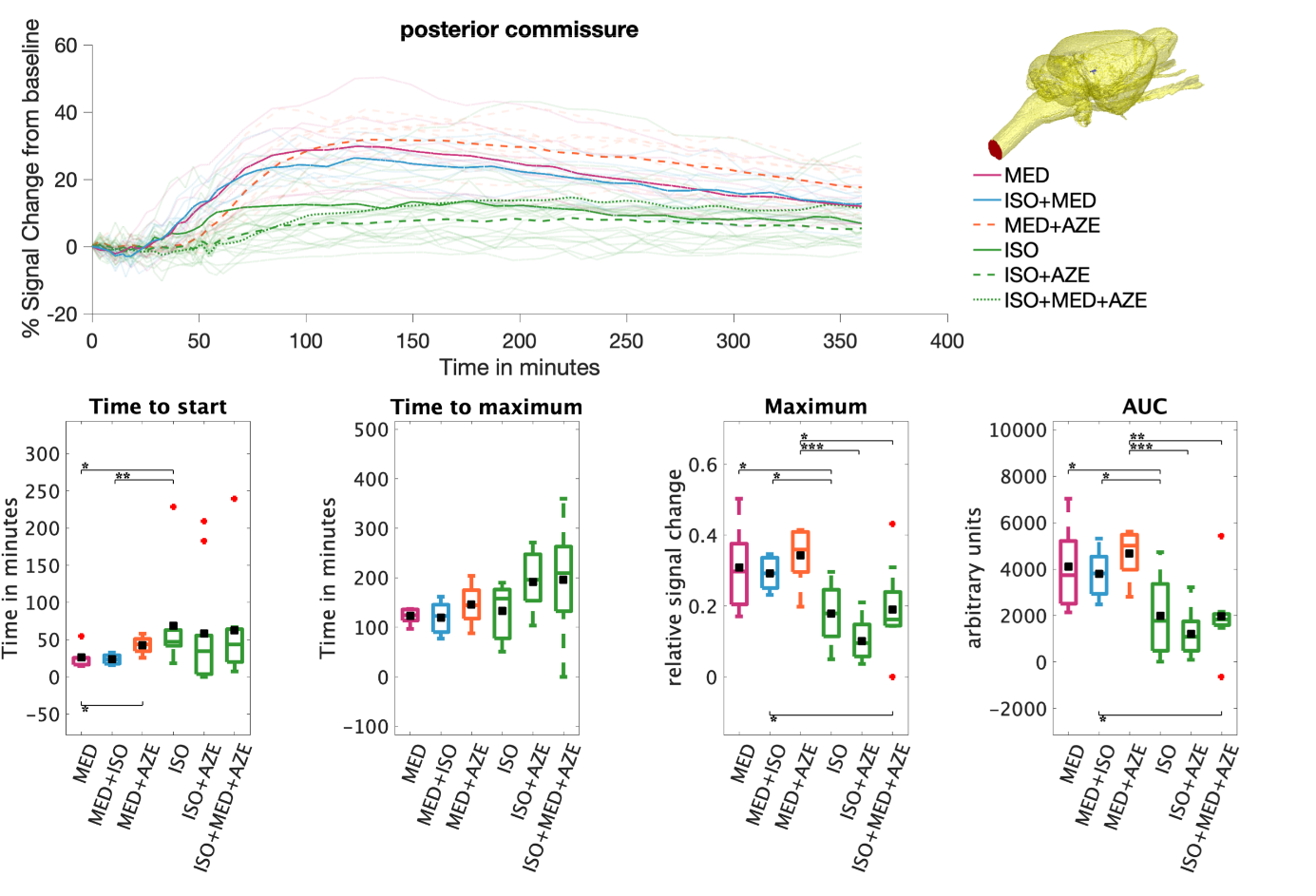

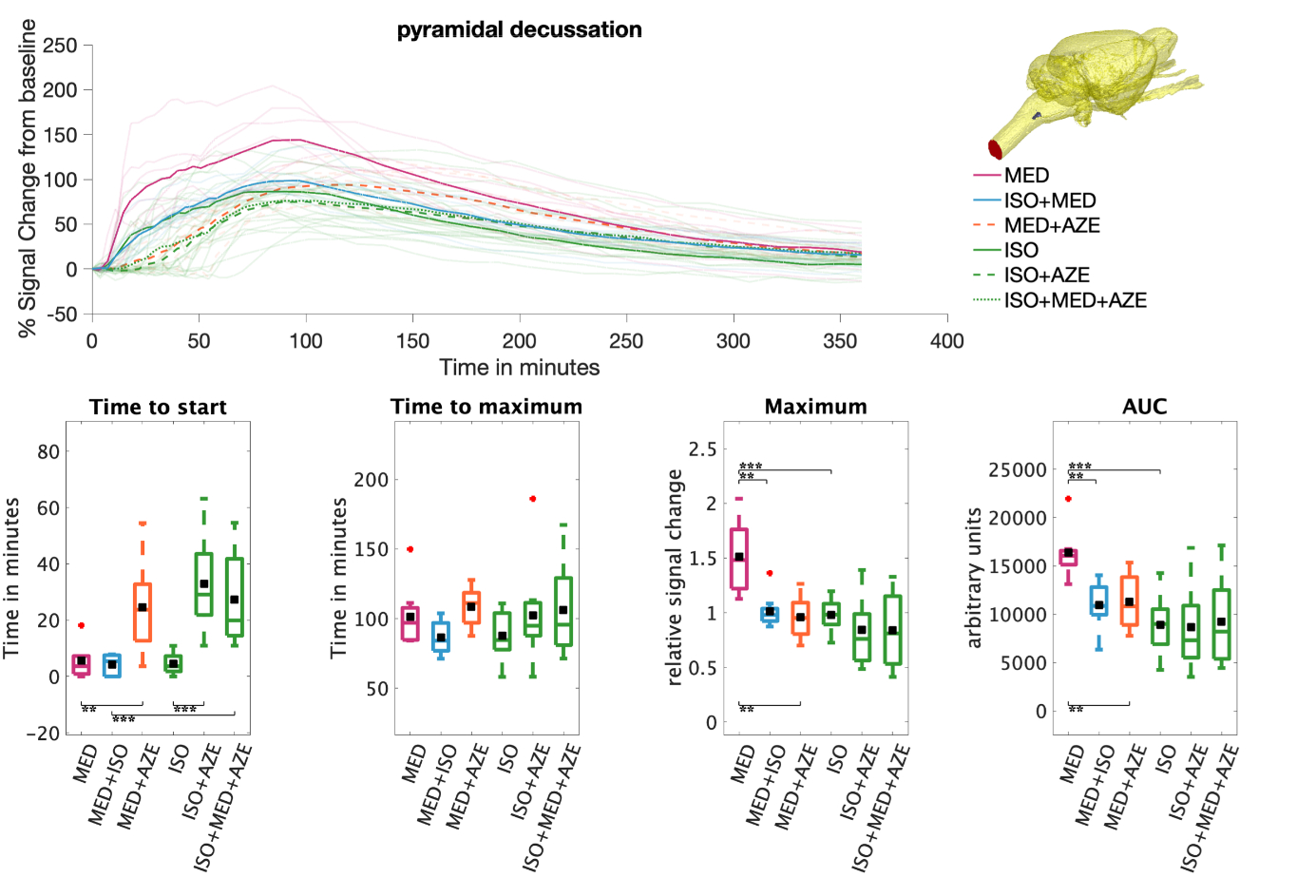

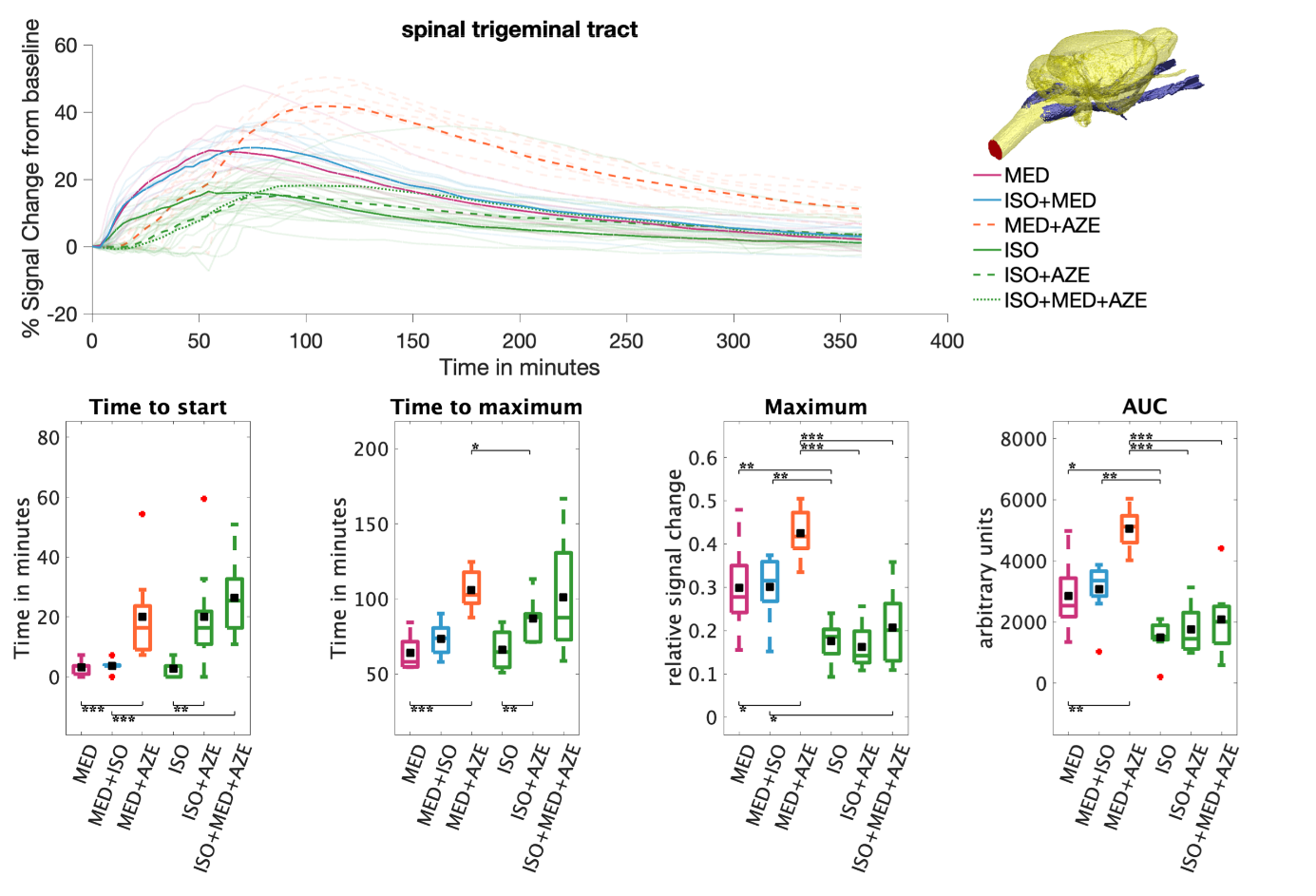

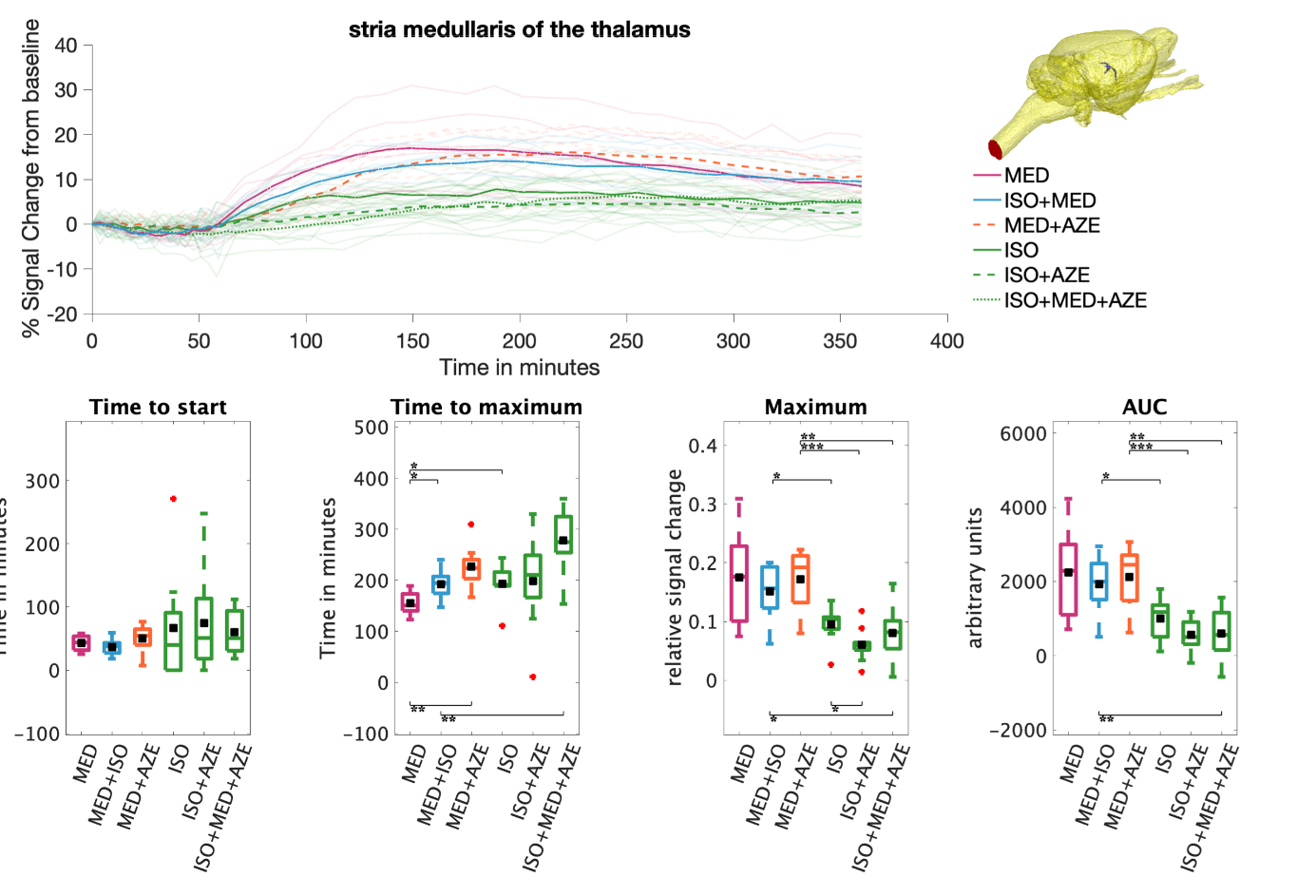

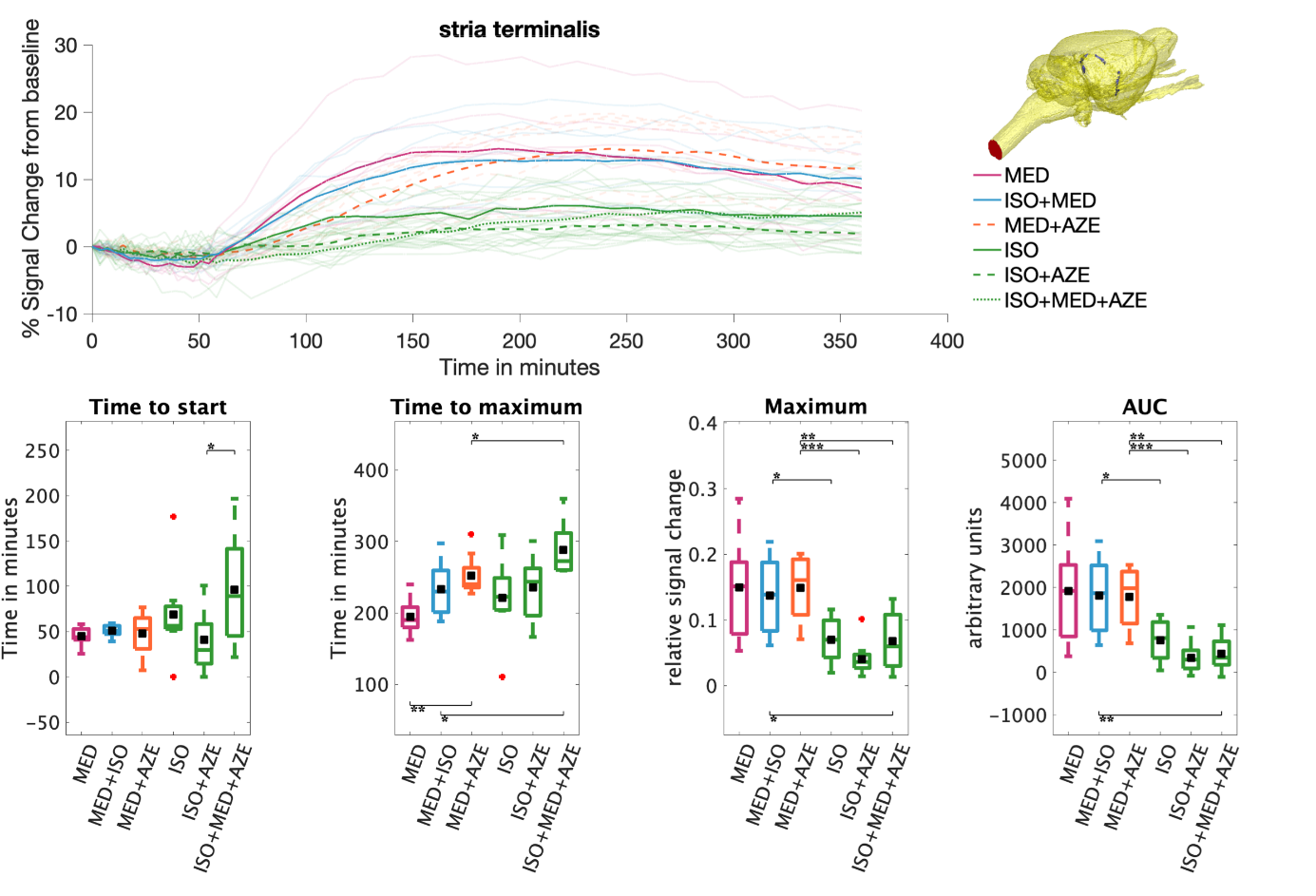

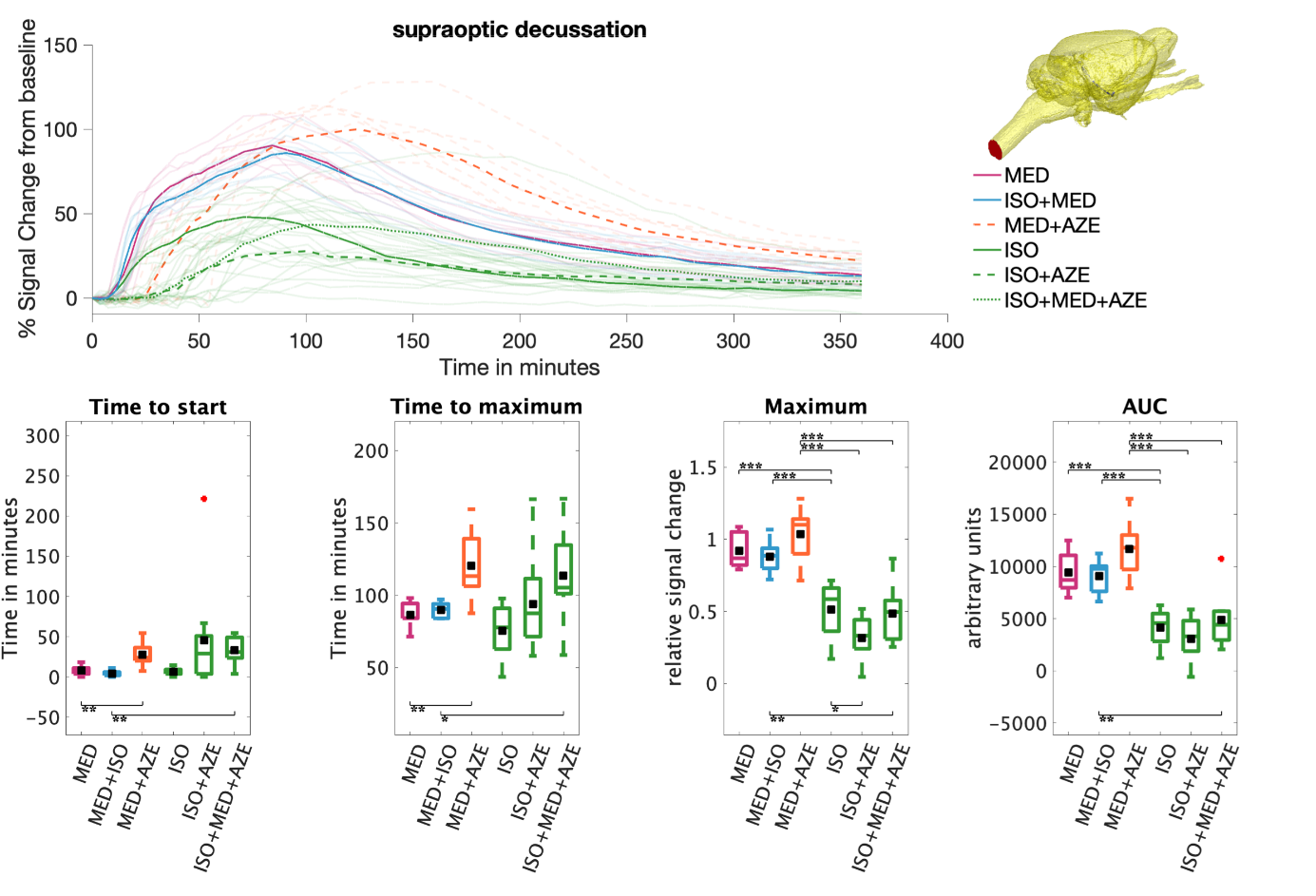

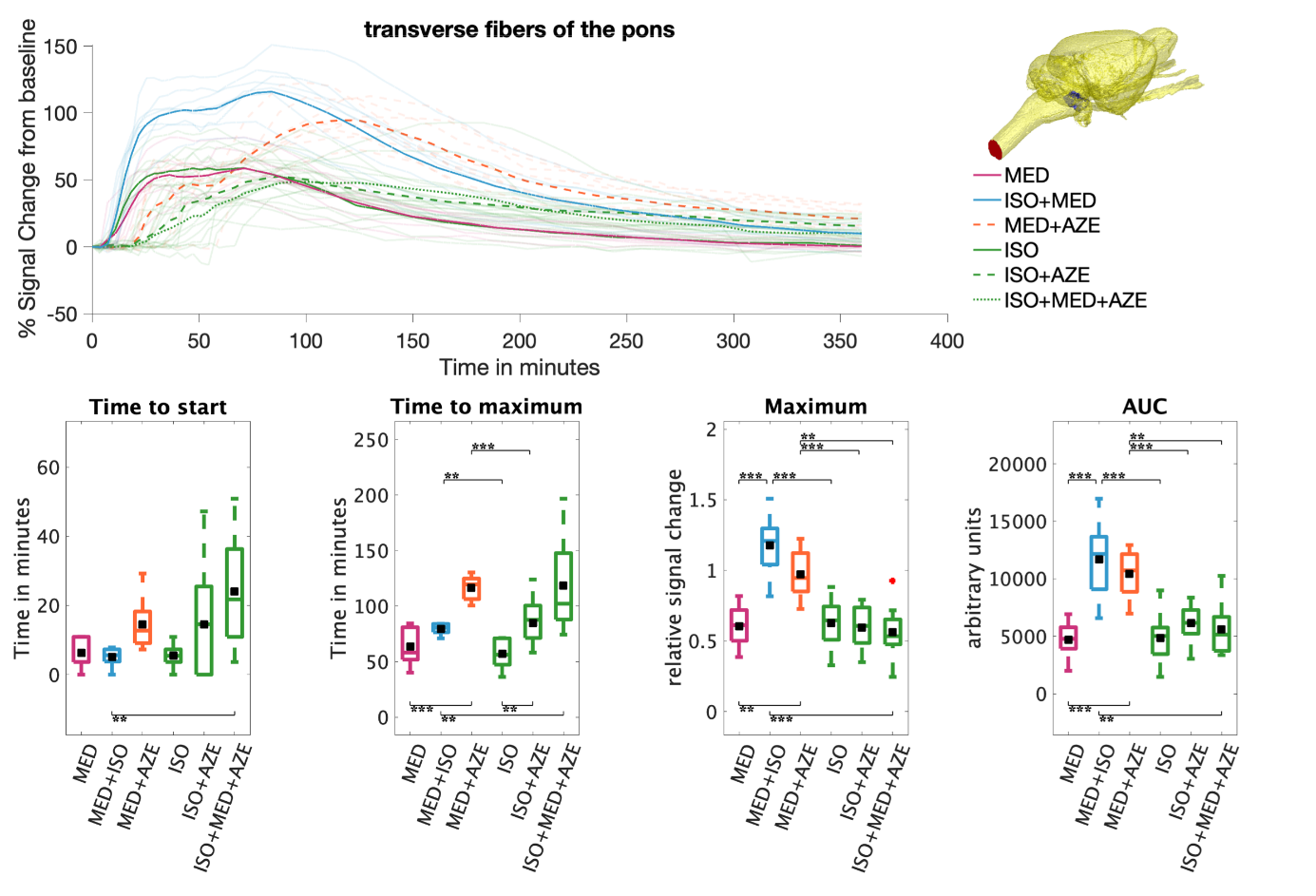

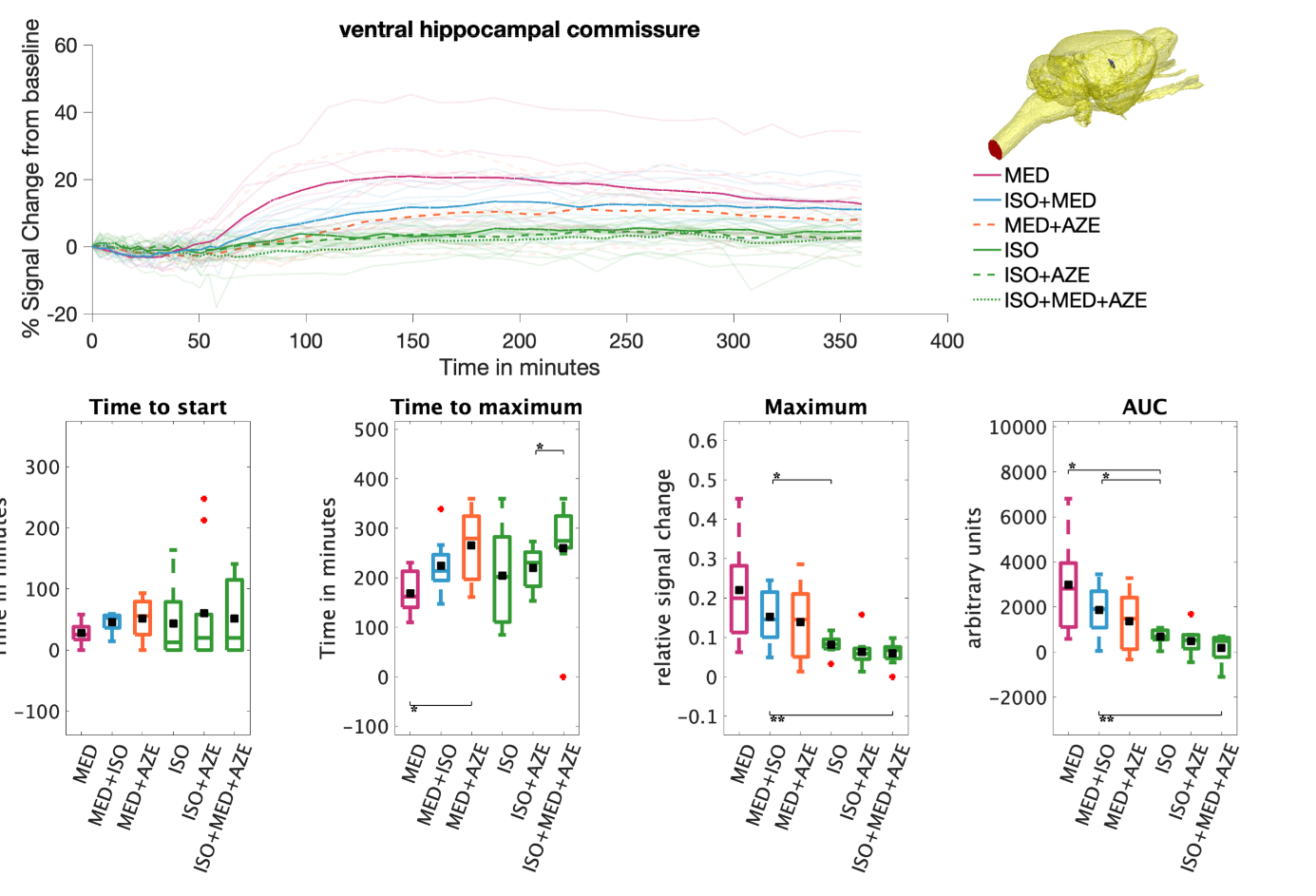

Supplement: Supplementary file 3 — Additionalfile 3. Brain region specific analysis. [file 12987_2023_443_MOESM3_ESM.docx]
