# Supplementary material for: Disentangling the impact of cerebrospinal fluid formation and neuronal activity on solute clearance from the brain
Source: Fluids Barriers CNS. 2023 Jun 14;20:43. doi: 10.1186/s12987-023-00443-2 (PMC10265831; doi:10.1186/s12987-023-00443-2)
Supplement: Supplementary file 4 — Additionalfile 4. Table of group comparison between the different anesthetic conditions for every brain region and for the four parameters (time to start; time to maximum; maximal signal; area under the curve) using Kruskal–Wallis test. [file 12987_2023_443_MOESM4_ESM.docx]

Additional file 4 – Table of group comparison between the different anesthetic conditions for every brain region and for the four parameters (Time to start; Time to maximum; Maximal signal; Area under the curve) using Kruskal-Wallis test.

| Region | Time to start | Time to maximum | Maximum signal | Area under curve |
| --- | --- | --- | --- | --- |
| descending corticofugal pathways | 0.0001 | 0.0001 | 0.0001 | 0.0001 |
| substantia nigra | < 0.0001 | 0.0001 | < 0.0001 | < 0.0001 |
| subthalamic nucleus | 0.0072 | < 0.0001 | 0.0001 | 0.0001 |
| molecular layer of the cerebellum | 0.0005 | 0.5158 | 0.0177 | 0.0245 |
| granule cell level of the cerebellum | 0.0007 | 0.1384 | 0.0194 | 0.0431 |
| alveus of the hippocampus | 0.2712 | 0.0035 | 0.0003 | 0.0003 |
| inferior cerebellar peduncle | 0.0002 | 0.0001 | 0.0007 | 0.0012 |
| cingulate cortex, area 2 | 0.0877 | 0.0132 | < 0.0001 | < 0.0001 |
| striatum | 0.1666 | 0.0015 | < 0.0001 | < 0.0001 |
| globus pallidus | 0.0828 | 0.0047 | < 0.0001 | < 0.0001 |
| entopeduncular nucleus | 0.3082 | 0.0314 | < 0.0001 | < 0.0001 |
| ventricular system | < 0.0001 | 0.0003 | < 0.0001 | < 0.0001 |
| medial lemniscus | 0.0004 | 0.0004 | 0.001 | 0.0014 |
| facial nerve | 0.0005 | 0.0002 | 0.0001 | 0.0002 |
| anterior commissure, anterior part | 0.0082 | < 0.0001 | 0.0001 | 0.0001 |
| anterior commissure, posterior part | 0.0197 | < 0.0001 | < 0.0001 | < 0.0001 |
| ventral hippocampal commissure | 0.6817 | 0.0479 | 0.0025 | 0.0034 |
| thalamus | 0.1799 | 0.0044 | 0.0003 | 0.0002 |
| septal region | 0.0371 | 0.0003 | < 0.0001 | < 0.0001 |
| optic nerve | 0.0003 | 0.0096 | 0.0407 | 0.0354 |
| optic tract and optic chiasm | 0.0002 | 0.0001 | < 0.0001 | < 0.0001 |
| pineal gland | < 0.0001 | 0.0015 | 0.0008 | 0.0009 |
| inner ear | 0.0012 | < 0.0001 | 0.0013 | 0.0008 |
| spinal cord | < 0.0001 | 0.0024 | 0.0007 | 0.0106 |
| commissure of the superior colliculus | 0.7198 | 0.0024 | 0.002 | 0.0036 |
| brainstem | < 0.0001 | < 0.0001 | 0.0001 | 0.0003 |
| hypothalamic region | 0.0002 | < 0.0001 | < 0.0001 | < 0.0001 |
| inferior colliculus | < 0.0001 | 0.0002 | 0.0002 | 0.0003 |
| superficial gray layer of the superior colliculus | 0.0005 | 0.0001 | 0.0003 | 0.0002 |
| periaqueductal gray | 0.2201 | < 0.0001 | 0.0005 | 0.0003 |
| fornix | 0.0318 | 0.0009 | 0.0001 | < 0.0001 |
| mammillothalamic tract | 0.0206 | < 0.0001 | 0.0006 | 0.0004 |
| commissural stria terminalis | 0.0217 | 0.0037 | < 0.0001 | < 0.0001 |
| deeper layers of the superior colliculus | 0.0775 | < 0.0001 | 0.0004 | 0.0004 |
| periventricular gray | 0.0001 | 0.0002 | 0.0024 | 0.004 |
| genu of the facial nerve | 0.0497 | 0.0001 | 0.0009 | 0.0011 |
| pontine nuclei | 0.0003 | < 0.0001 | < 0.0001 | < 0.0001 |
| fimbria of the hippocampus | 0.4705 | 0.0226 | 0.0042 | 0.0008 |
| fasciculus retroflexus | 0.013 | 0.0367 | 0.0012 | 0.0009 |
| stria medullaris of the thalamus | 0.7536 | 0.002 | 0.0002 | 0.0002 |
| stria terminalis | 0.2656 | 0.0042 | 0.0001 | 0.0001 |
| posterior commissure | 0.0416 | 0.038 | 0.0001 | 0.0001 |
| glomerular layer of the accessory olfactory bulb | < 0.0001 | 0.0004 | < 0.0001 | < 0.0001 |
| glomerular layer of the olfactory bulb | < 0.0001 | 0.0001 | < 0.0001 | < 0.0001 |
| olfactory bulb | < 0.0001 | < 0.0001 | < 0.0001 | < 0.0001 |
| corpus callosum and associated subcortical white matter | 0.0546 | 0.0033 | 0.0002 | 0.0001 |
| brachium of the superior colliculus | 0.4221 | 0.0033 | 0.0011 | 0.0009 |
| commissure of the inferior colliculus | 0.014 | 0.0002 | 0.0002 | 0.0001 |
| interpeduncular nucleus | 0.0001 | 0.0032 | 0.0003 | 0.0004 |
| ascending fibers of the facial nerve | 0.6303 | 0.0014 | 0.001 | 0.0017 |
| anterior commissure | 0.0428 | < 0.0001 | < 0.0001 | < 0.0001 |
| inferior olive | 0.0001 | 0.0004 | 0.0011 | 0.001 |
| spinal trigeminal nuclus | < 0.0001 | < 0.0001 | < 0.0001 | 0.0005 |
| spinal trigeminal tract | < 0.0001 | 0.0001 | < 0.0001 | < 0.0001 |
| frontal associiation cortex | 0.0023 | 0.007 | < 0.0001 | < 0.0001 |
| middle cerebellar peduncle | < 0.0001 | < 0.0001 | 0.0019 | 0.0009 |
| transverse fibers of the pons | 0.0138 | < 0.0001 | < 0.0001 | < 0.0001 |
| habenular commissure | 0.2908 | 0.005 | < 0.0001 | < 0.0001 |
| nucleus of the stria medullaris | 0.1302 | < 0.0001 | 0.0001 | < 0.0001 |
| basal forebrain region | 0.0001 | 0.0001 | < 0.0001 | < 0.0001 |
| supraoptic decussation | 0.001 | 0.0021 | < 0.0001 | < 0.0001 |
| medial lemniscus decussation | 0.0009 | 0.0766 | 0.0003 | 0.0056 |
| pyramidal decussation | < 0.0001 | 0.0726 | 0.0072 | 0.0057 |
| neocortex | 0.0108 | 0.0067 | < 0.0001 | < 0.0001 |
| bed nucleus of the stria terminalis | 0.0135 | < 0.0001 | 0.0001 | < 0.0001 |
| pretectal region | 0.048 | < 0.0001 | 0.0016 | 0.0013 |
| cornu ammonis 1 | 0.001 | 0.0002 | 0.0002 | 0.0004 |
| dentate gyrus | 0.0003 | 0.0029 | 0.0002 | 0.0001 |
| cornu ammonis 2 | 0.0838 | 0.001 | 0.0007 | 0.0009 |
| cornu ammonis 3 | 0.0338 | < 0.0001 | 0.0004 | 0.0003 |
| fasciola cinereum | 0.5464 | 0.1319 | 0.0015 | 0.0002 |
| subiculum | 0.0008 | < 0.0001 | 0.0001 | 0.0001 |
| postrhinal cortex | 0.3357 | 0.0006 | 0.0001 | 0.0001 |
| presubiculum | 0.0001 | < 0.0001 | < 0.0001 | < 0.0001 |
| parasubiculum | < 0.0001 | < 0.0001 | 0.0001 | 0.0001 |
| perirhinal area 35 | 0.265 | 0.0609 | < 0.0001 | < 0.0001 |
| perirhinal area 36 | 0.1789 | 0.0417 | 0.0001 | 0.0001 |
| entorhinal cortex | < 0.0001 | < 0.0001 | 0.0001 | 0.0001 |
| lateral entorhinal cortex | < 0.0001 | 0.0002 | 0.0001 | < 0.0001 |
