# Supplementary material for: Disentangling the impact of cerebrospinal fluid formation and neuronal activity on solute clearance from the brain
Source: Fluids Barriers CNS. 2023 Jun 14;20:43. doi: 10.1186/s12987-023-00443-2 (PMC10265831; doi:10.1186/s12987-023-00443-2)
Supplement: Supplementary file 5 — Additionalfile 5. Signal decay rate of TSCs under MED and ISO+MED. [file 12987_2023_443_MOESM5_ESM.docx]

Additional file 5 – Signal decay rate of TSCs under MED and ISO+MED


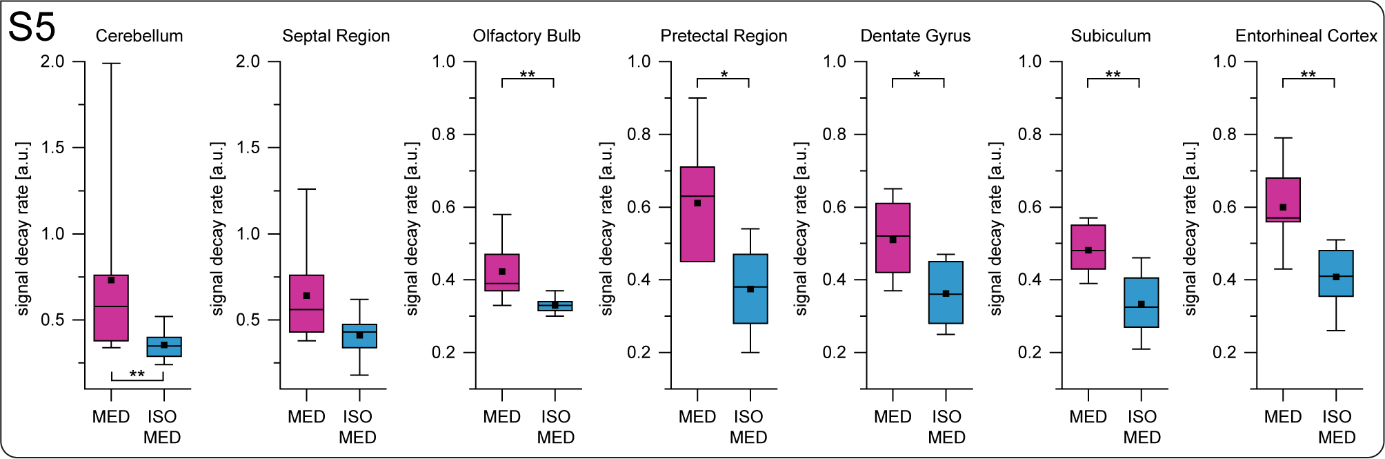


Signal decay rate of TSCs under MED and ISO+MED for exemplary brain regions (Cerebellum, Septal Region, Olfactory Bulb, Pretectal Region, Dentate Gyrus, Subiculum, Entorhineal Cortex), calculated by mono-exponentially fitting of the individual region-specific TSCs over the time period between 90 min after TSC maximum and end of data acquisition (MED n = 7; ISO+MED n = 8).
