# Supplementary material for: Disentangling the impact of cerebrospinal fluid formation and neuronal activity on solute clearance from the brain
Source: Fluids Barriers CNS. 2023 Jun 14;20:43. doi: 10.1186/s12987-023-00443-2 (PMC10265831; doi:10.1186/s12987-023-00443-2)
Supplement: Supplementary file 6 — Additionalfile 6. Solution of the differential equation. [file 12987_2023_443_MOESM6_ESM.docx]

Additional file 6 – Solution of the differential equation


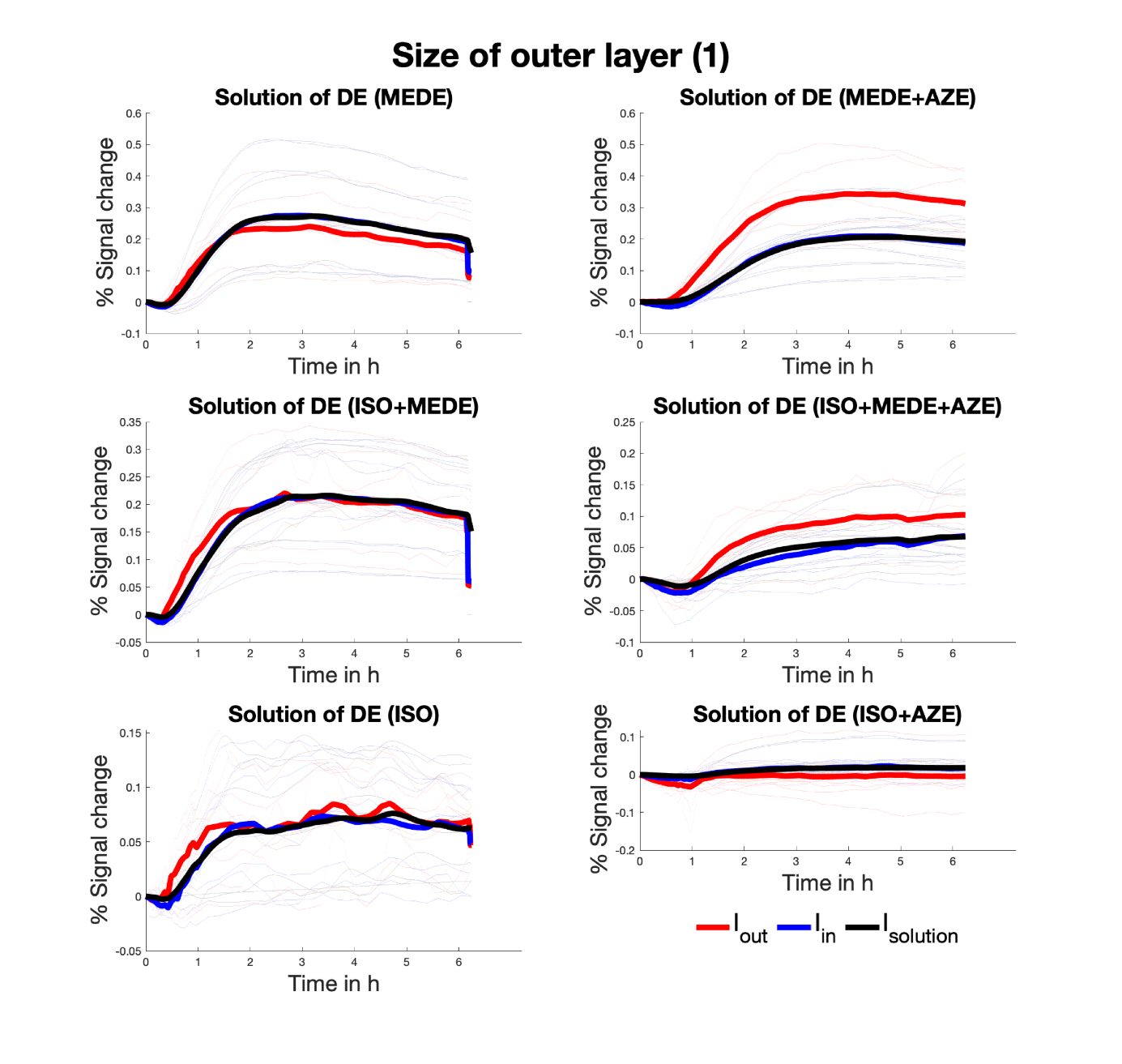


Illustration of the time signal curves of the inner (blue) and outer (red) layer and the solution of the differential equation (black) for each anesthetic condition with a thickness of the outer layer of one voxel for every animal (thin curves) and mean (thick curves). The signal change is displayed as percent and the time is illustrated in hours.

The solution of the differential equation ($I_{in}^{\mathrm{sol}}\left( t \right)$) is the calculated TSC for the inner layer from the TSC of outer layer (I_out_) and the exchange parameters k_1_ and k_2_.


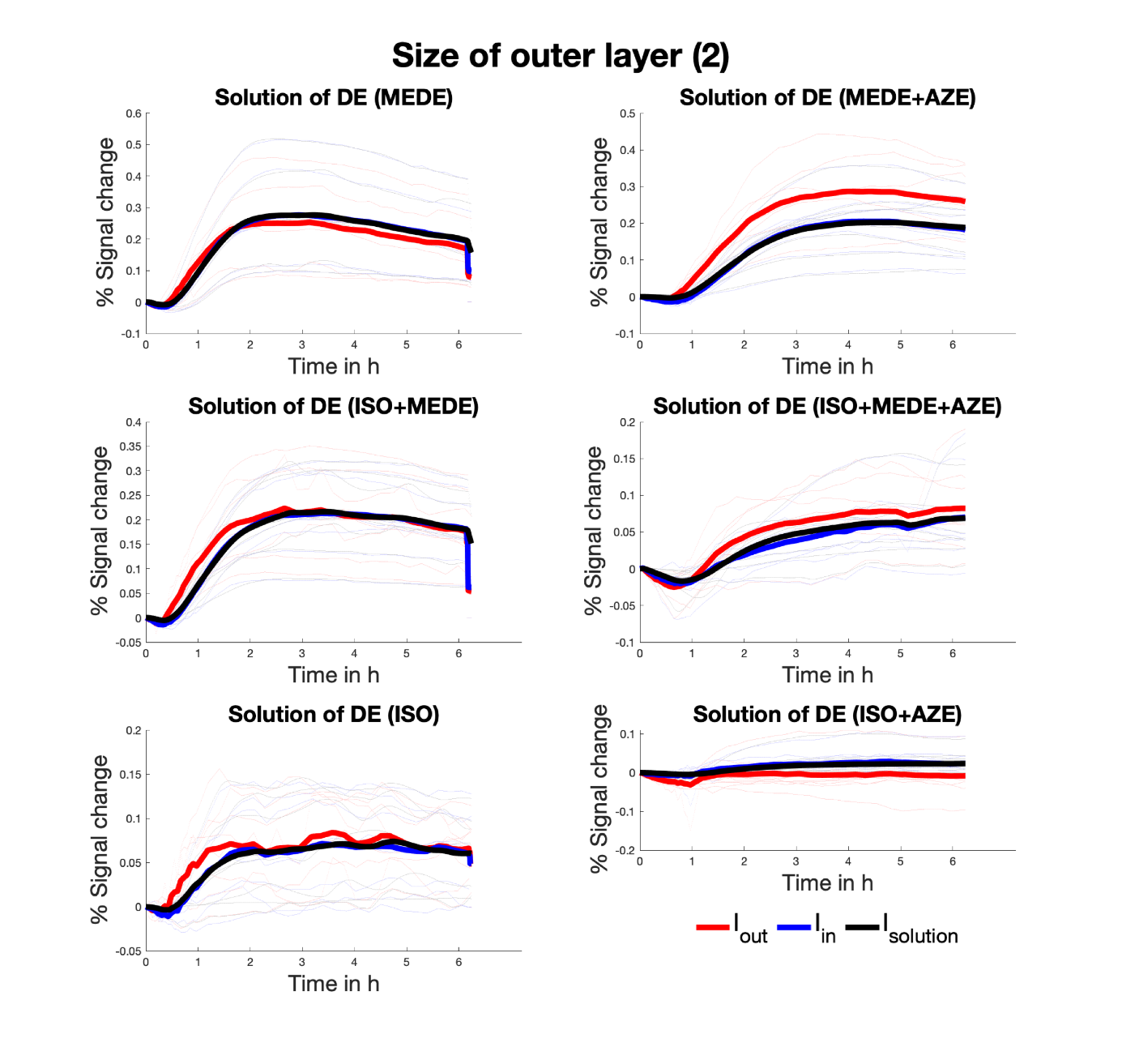


Illustration of the time signal curves of the inner (blue) and outer (red) layer and the solution of the differential equation (black) for each anesthetic condition with a thickness of the outer layer of two voxel for every animal (thin curves) and mean (thick curves). The signal change is displayed as percent and the time is illustrated in hours.

The solution of the differential equation ($I_{in}^{\mathrm{sol}}\left( t \right)$) is the calculated TSC for the inner layer from the TSC of outer layer (I_out_) and the exchange parameters k_1_ and k_2_.


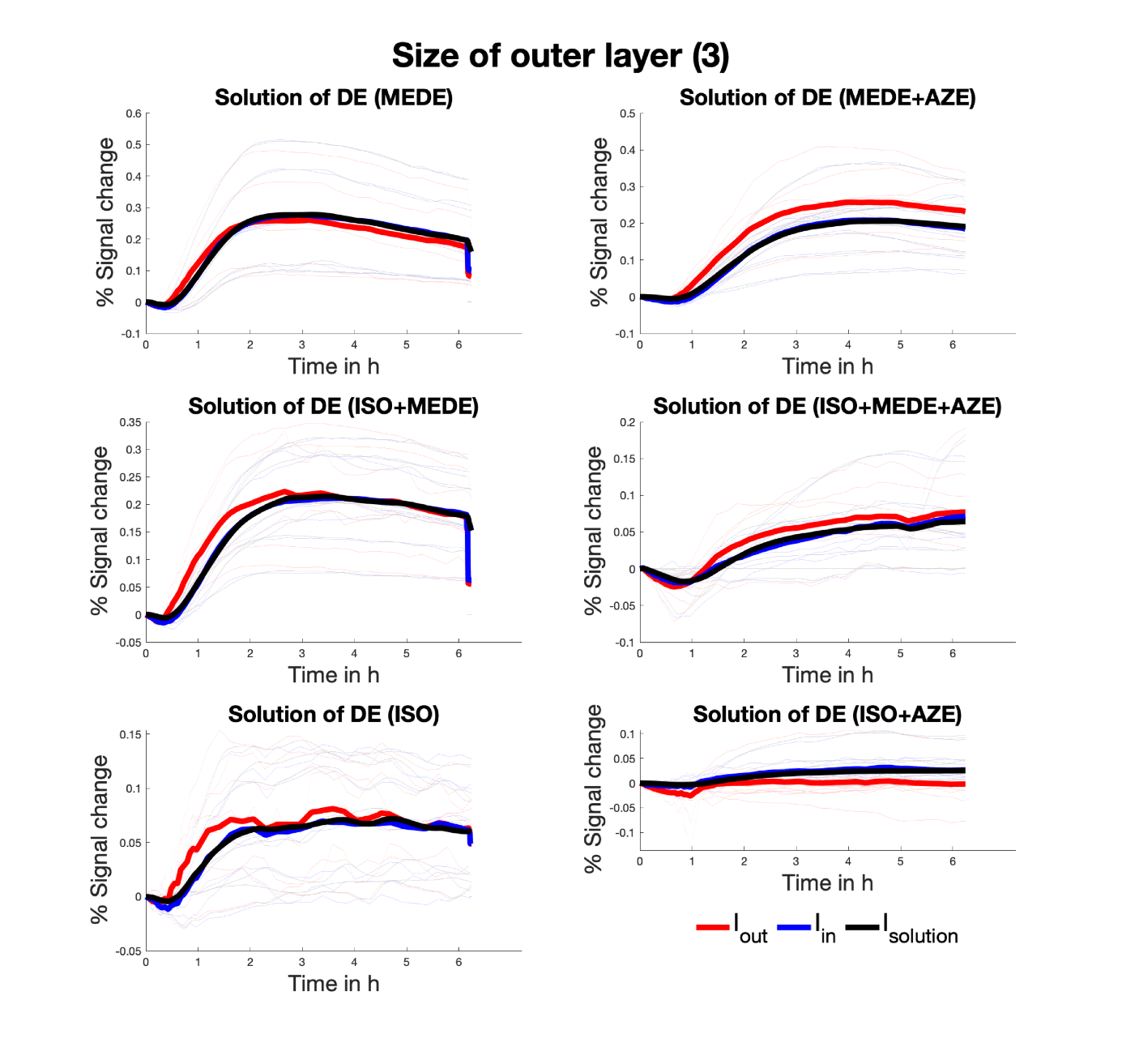


Illustration of the time signal curves of the inner (blue) and outer (red) layer and the solution of the differential equation (black) for each anesthetic condition with a thickness of the outer layer of three voxel for every animal (thin curves) and mean (thick curves). The signal change is displayed as percent and the time is illustrated in hours.

The solution of the differential equation ($I_{in}^{\mathrm{sol}}\left( t \right)$) is the calculated TSC for the inner layer from the TSC of outer layer (I_out_) and the exchange parameters k_1_ and k_2_.


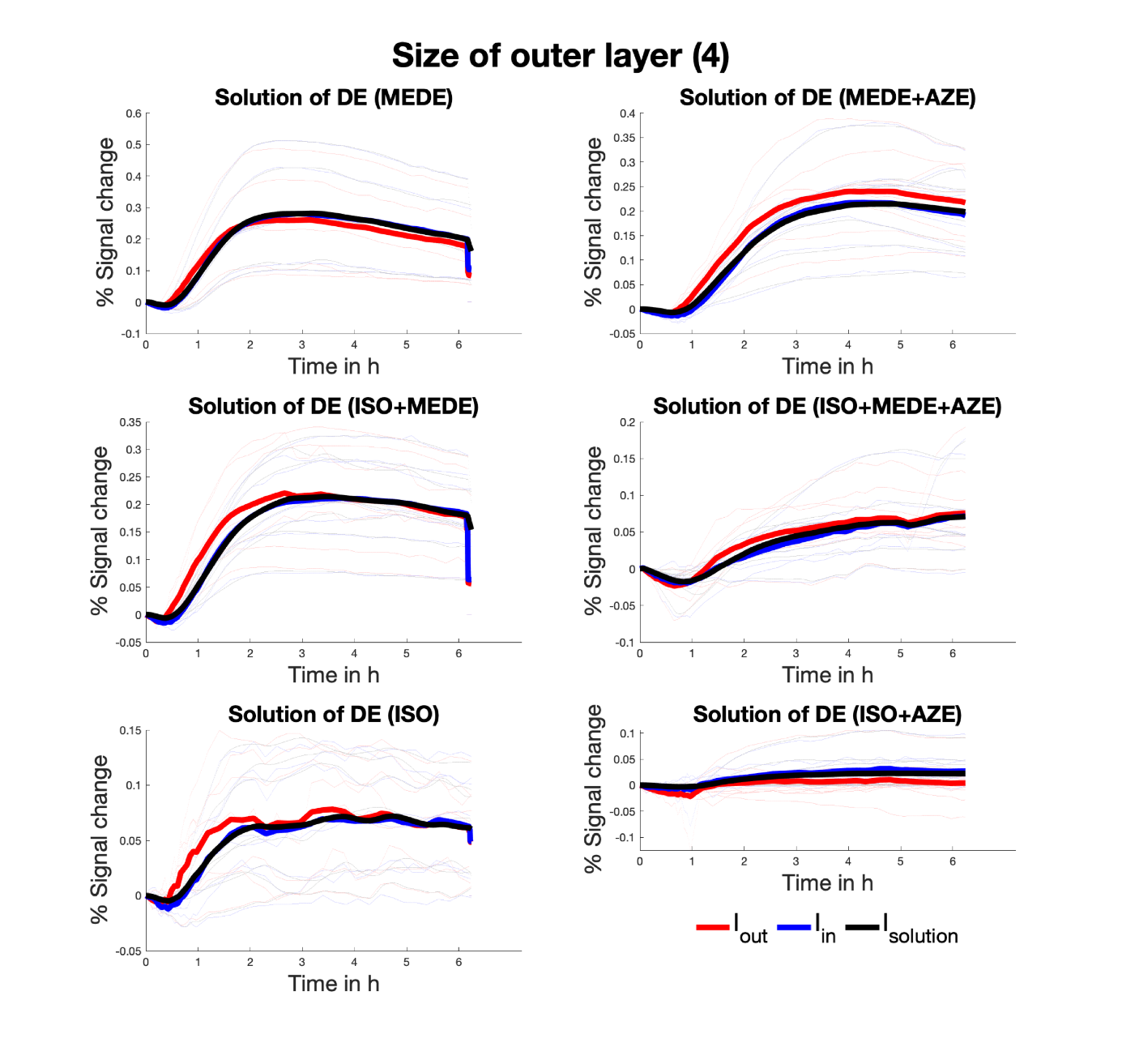


Illustration of the time signal curves of the inner (blue) and outer (red) layer and the solution of the differential equation (black) for each anesthetic condition with a thickness of the outer layer of four voxel for every animal (thin curves) and mean (thick curves). The signal change is displayed as percent and the time is illustrated in hours.

The solution of the differential equation ($I_{in}^{\mathrm{sol}}\left( t \right)$) is the calculated TSC for the inner layer from the TSC of outer layer (I_out_) and the exchange parameters k_1_ and k_2_.


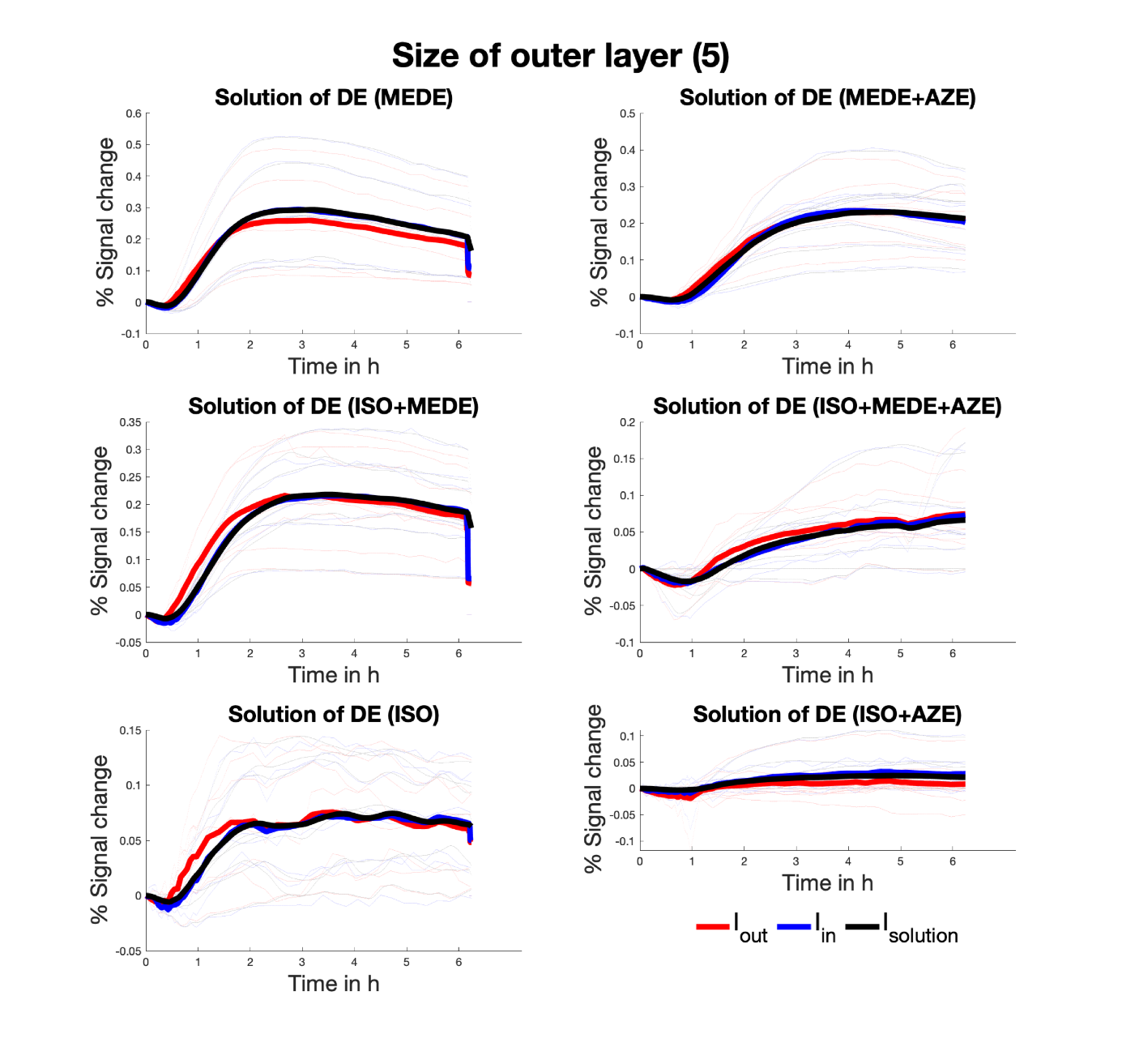


Illustration of the time signal curves of the inner (blue) and outer (red) layer and the solution of the differential equation (black) for each anesthetic condition with a thickness of the outer layer of five voxel for every animal (thin curves) and mean (thick curves). The signal change is displayed as percent and the time is illustrated in hours.

The solution of the differential equation ($I_{in}^{\mathrm{sol}}\left( t \right)$) is the calculated TSC for the inner layer from the TSC of outer layer (I_out_) and the exchange parameters k_1_ and k_2_.


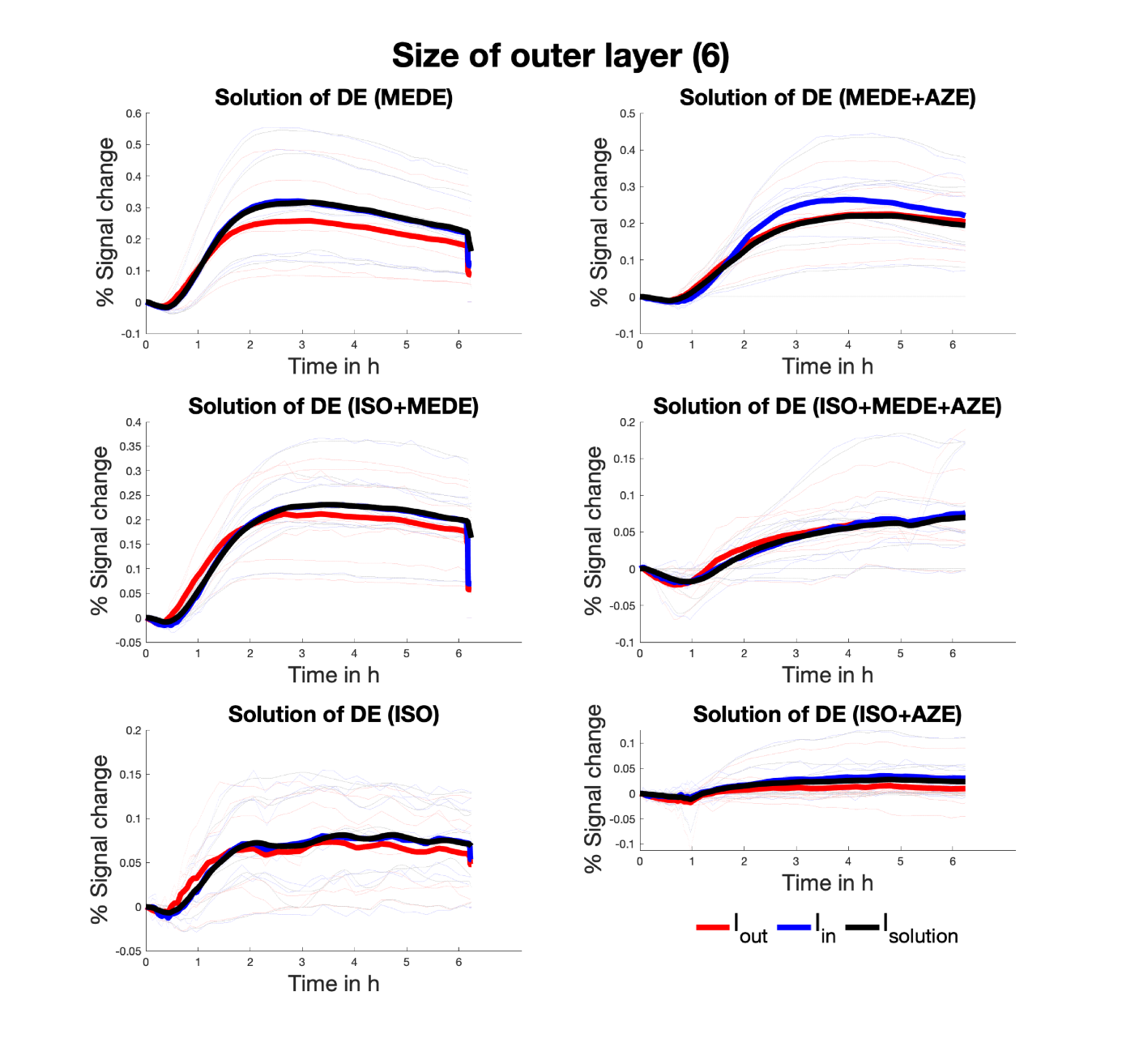


Illustration of the time signal curves of the inner (blue) and outer (red) layer and the solution of the differential equation (black) for each anesthetic condition with a thickness of the outer layer of six voxel for every animal (thin curves) and mean (thick curves). The signal change is displayed as percent and the time is illustrated in hours.

The solution of the differential equation ($I_{in}^{\mathrm{sol}}\left( t \right)$) is the calculated TSC for the inner layer from the TSC of outer layer (I_out_) and the exchange parameters k_1_ and k_2_.


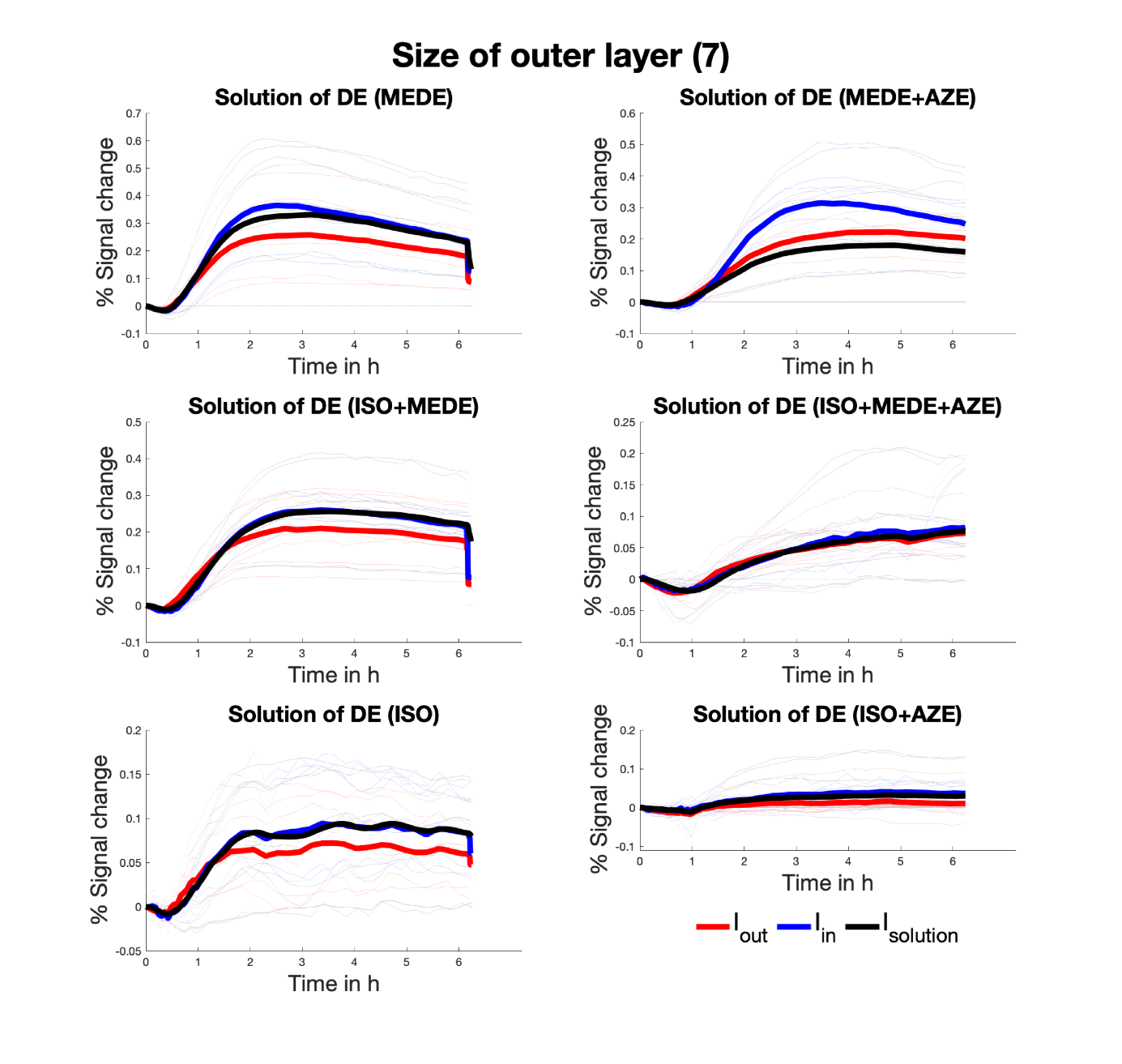


Illustration of the time signal curves of the inner (blue) and outer (red) layer and the solution of the differential equation (black) for each anesthetic condition with a thickness of the outer layer of seven voxel for every animal (thin curves) and mean (thick curves). The signal change is displayed as percent and the time is illustrated in hours.

The solution of the differential equation ($I_{in}^{\mathrm{sol}}\left( t \right)$) is the calculated TSC for the inner layer from the TSC of outer layer (I_out_) and the exchange parameters k_1_ and k_2_.


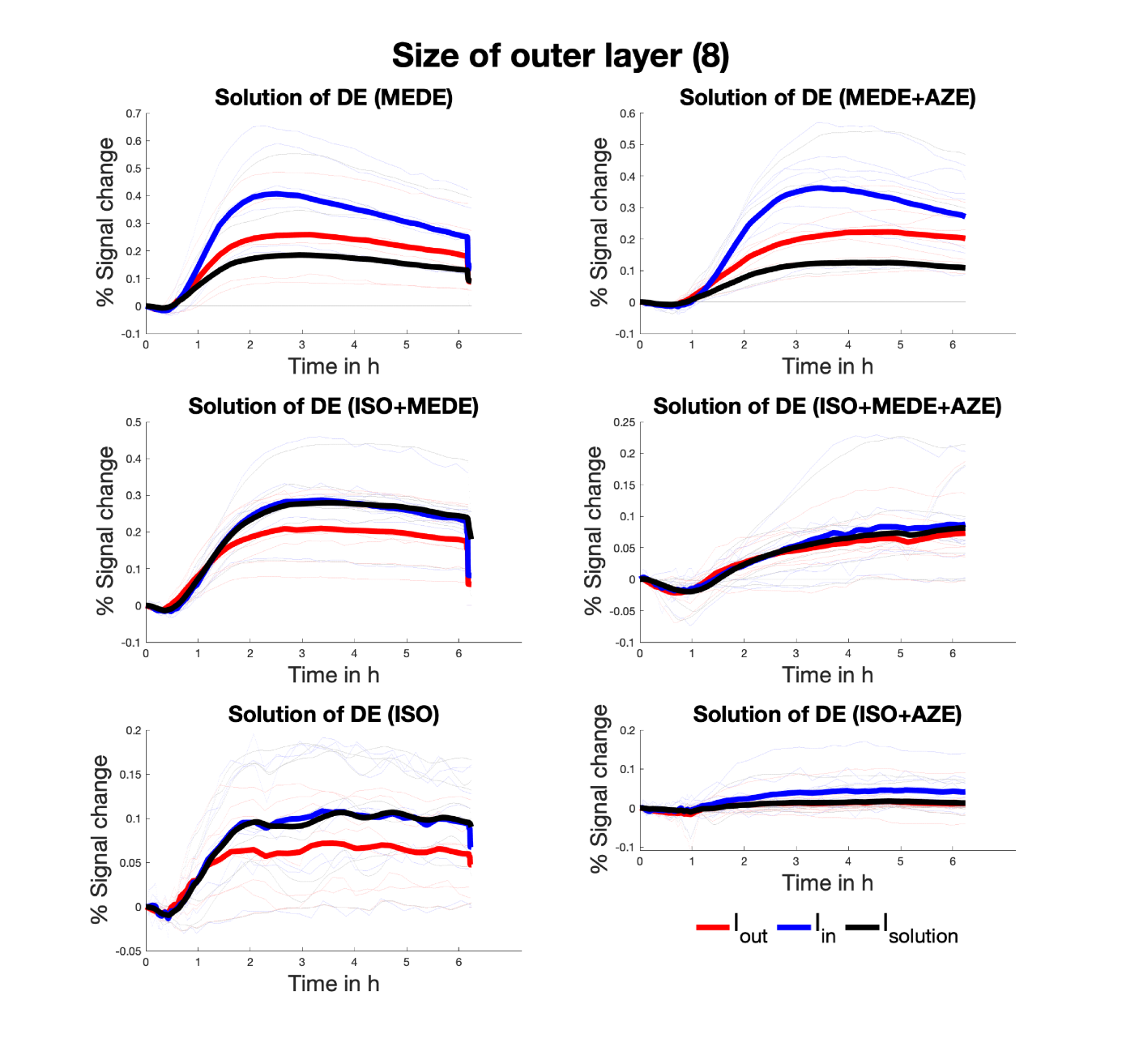


Illustration of the time signal curves of the inner (blue) and outer (red) layer and the solution of the differential equation (black) for each anesthetic condition with a thickness of the outer layer of eight voxel for every animal (thin curves) and mean (thick curves). The signal change is displayed as percent and the time is illustrated in hours.

The solution of the differential equation ($I_{in}^{\mathrm{sol}}\left( t \right)$) is the calculated TSC for the inner layer from the TSC of outer layer (I_out_) and the exchange parameters k_1_ and k_2_.


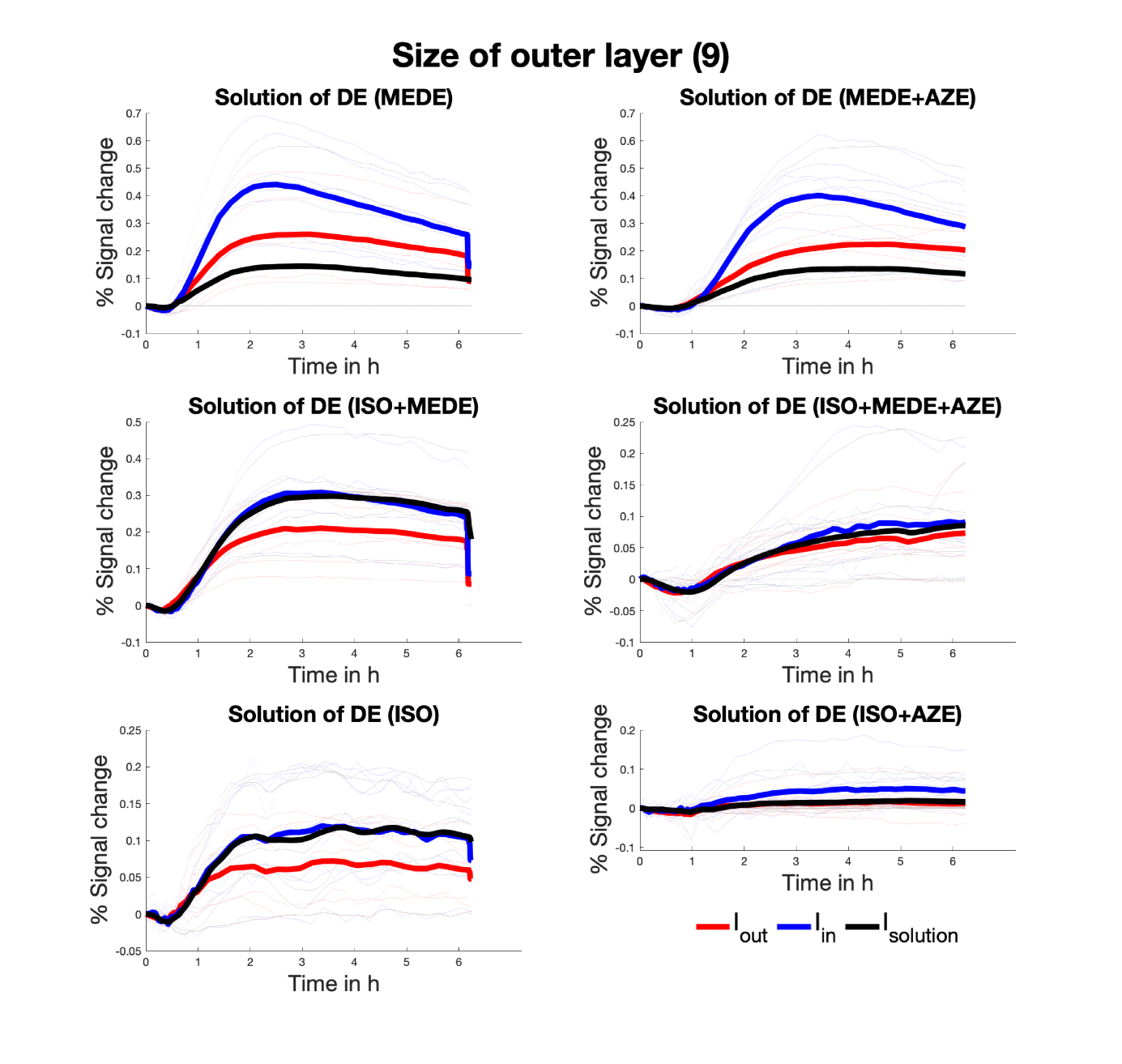


Illustration of the time signal curves of the inner (blue) and outer (red) layer and the solution of the differential equation (black) for each anesthetic condition with a thickness of the outer layer of nine voxel for every animal (thin curves) and mean (thick curves). The signal change is displayed as percent and the time is illustrated in hours.

The solution of the differential equation ($I_{in}^{\mathrm{sol}}\left( t \right)$) is the calculated TSC for the inner layer from the TSC of outer layer (I_out_) and the exchange parameters k_1_ and k_2_.


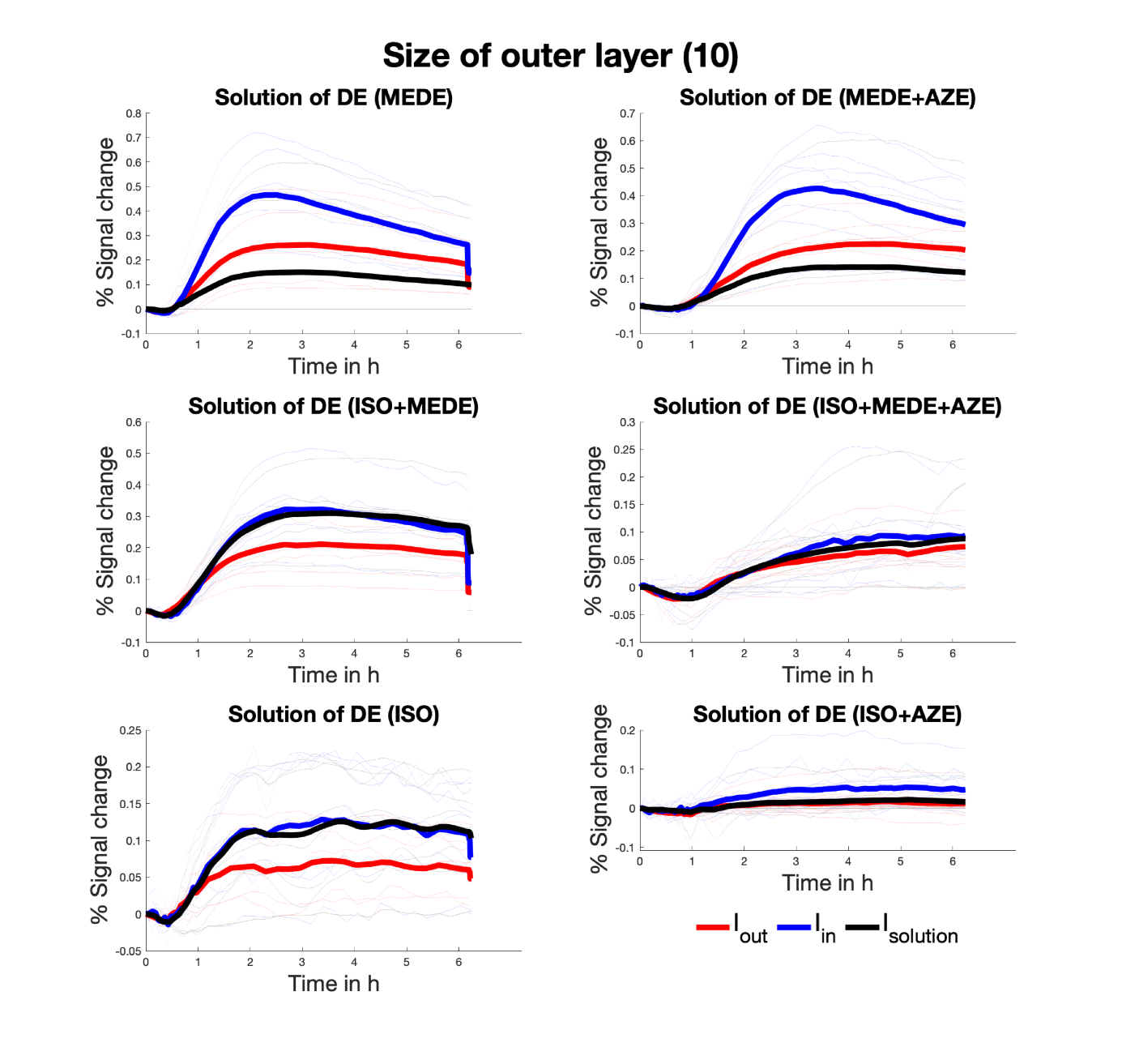


Illustration of the time signal curves of the inner (blue) and outer (red) layer and the solution of the differential equation (black) for each anesthetic condition with a thickness of the outer layer of ten voxel for every animal (thin curves) and mean (thick curves). The signal change is displayed as percent and the time is illustrated in hours.

The solution of the differential equation ($I_{in}^{\mathrm{sol}}\left( t \right)$) is the calculated TSC for the inner layer from the TSC of outer layer (I_out_) and the exchange parameters k_1_ and k_2_.


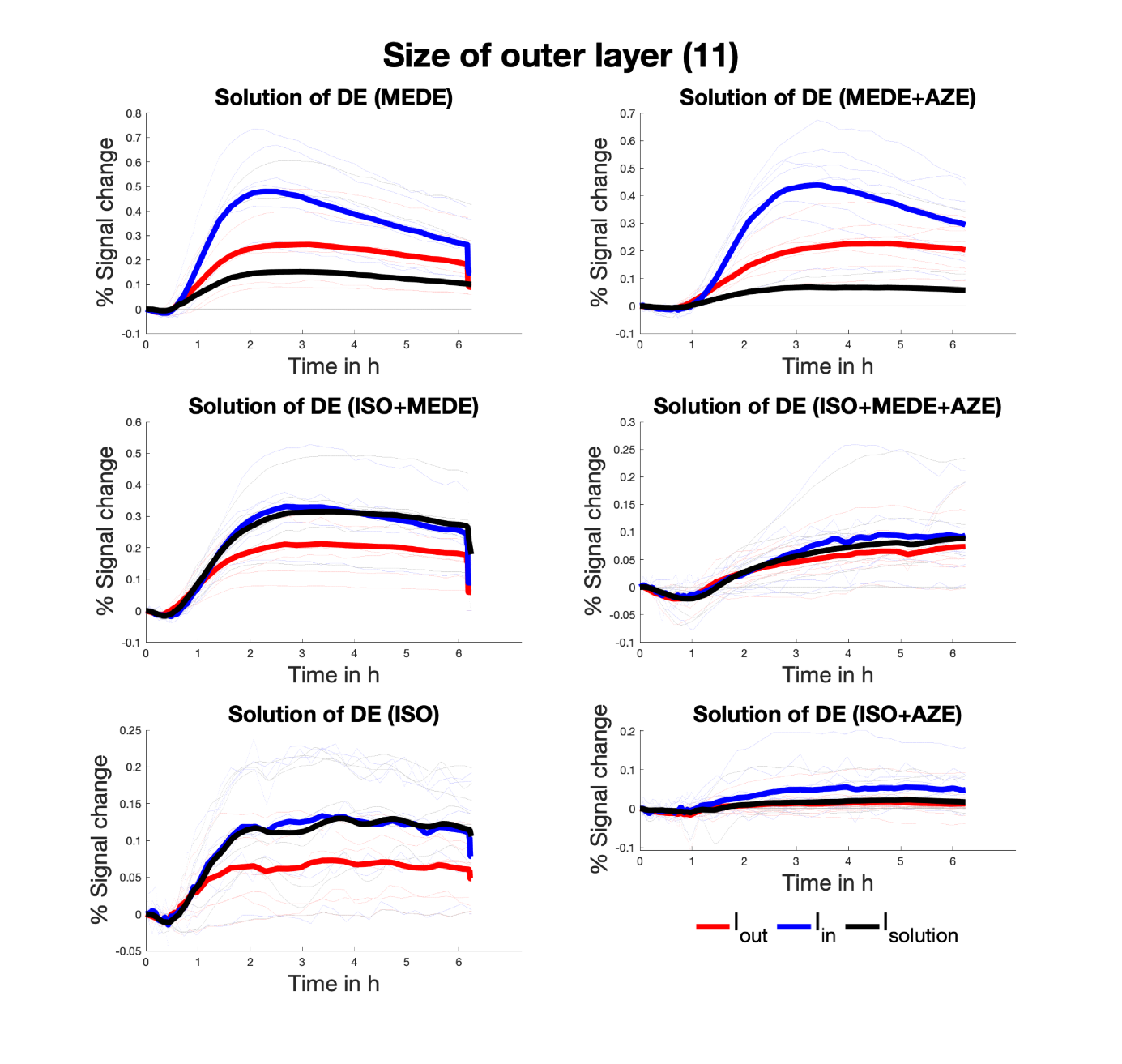


Illustration of the time signal curves of the inner (blue) and outer (red) layer and the solution of the differential equation (black) for each anesthetic condition with a thickness of the outer layer of eleven voxel for every animal (thin curves) and mean (thick curves). The signal change is displayed as percent and the time is illustrated in hours.

The solution of the differential equation ($I_{in}^{\mathrm{sol}}\left( t \right)$) is the calculated TSC for the inner layer from the TSC of outer layer (I_out_) and the exchange parameters k_1_ and k_2_.


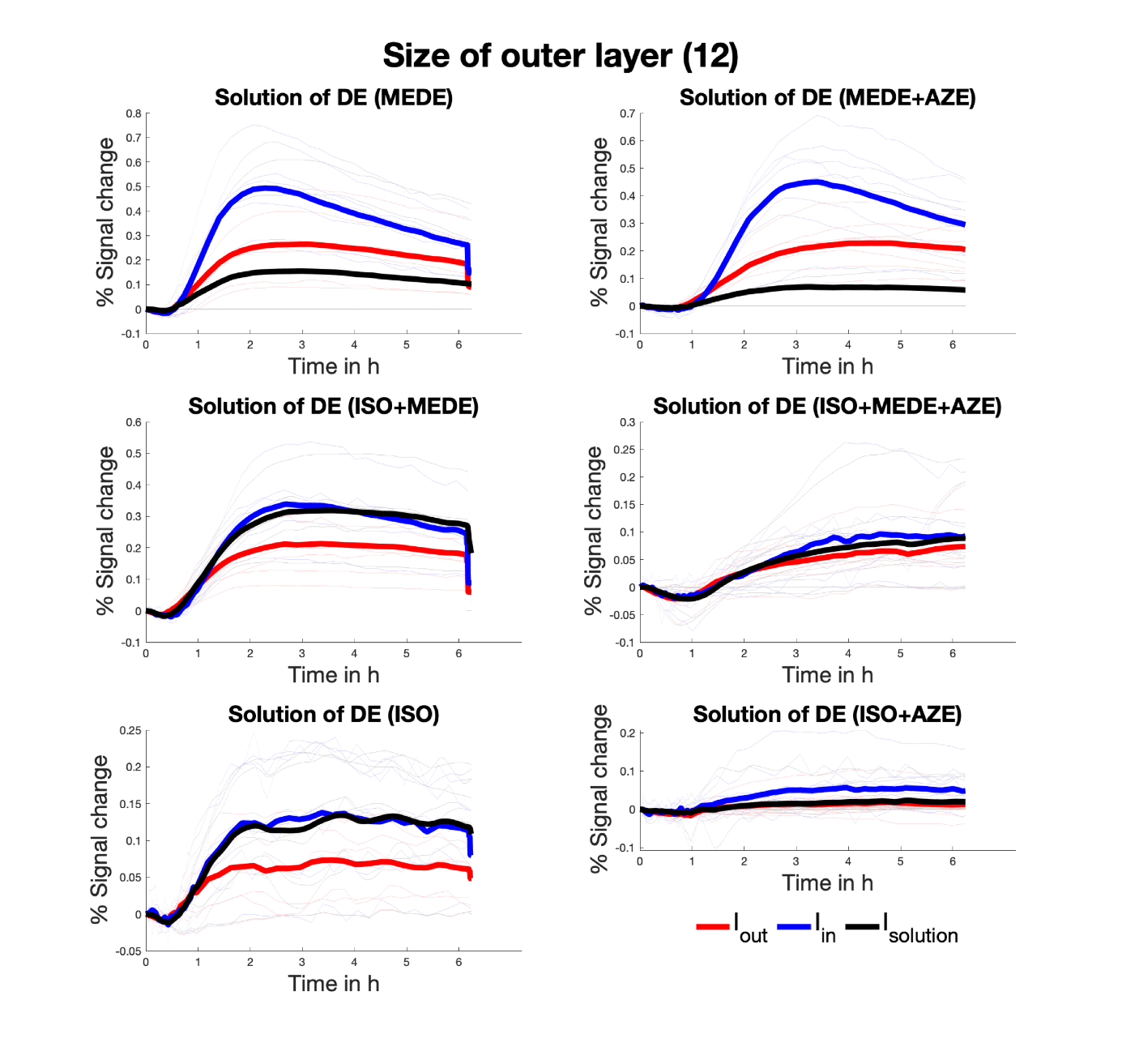


Illustration of the time signal curves of the inner (blue) and outer (red) layer and the solution of the differential equation (black) for each anesthetic condition with a thickness of the outer layer of twelve voxel for every animal (thin curves) and mean (thick curves). The signal change is displayed as percent and the time is illustrated in hours.

The solution of the differential equation ($I_{in}^{\mathrm{sol}}\left( t \right)$) is the calculated TSC for the inner layer from the TSC of outer layer (I_out_) and the exchange parameters k_1_ and k_2_.


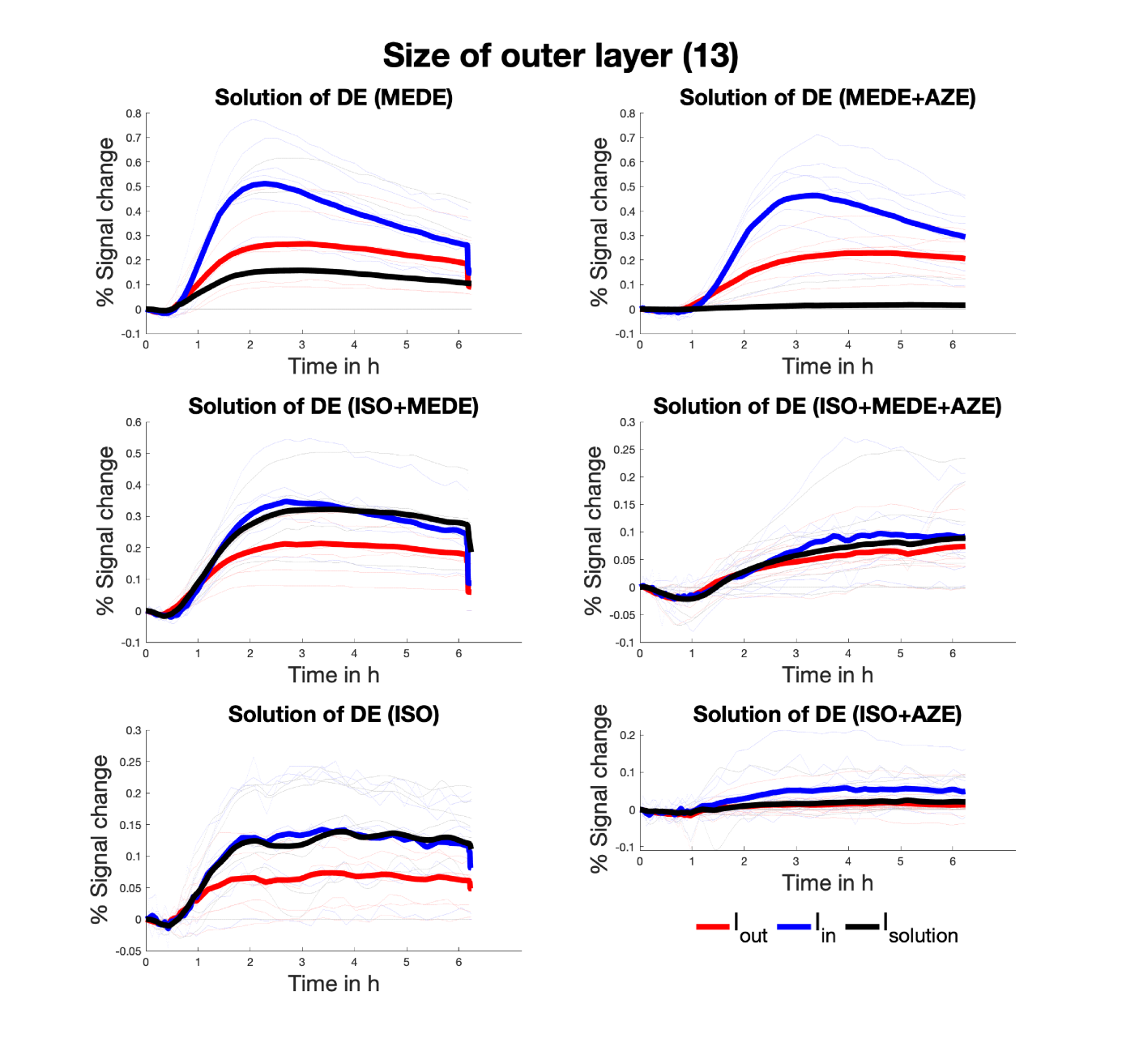


Illustration of the time signal curves of the inner (blue) and outer (red) layer and the solution of the differential equation (black) for each anesthetic condition with a thickness of the outer layer of thirteen voxel for every animal (thin curves) and mean (thick curves). The signal change is displayed as percent and the time is illustrated in hours.

The solution of the differential equation ($I_{in}^{\mathrm{sol}}\left( t \right)$) is the calculated TSC for the inner layer from the TSC of outer layer (I_out_) and the exchange parameters k_1_ and k_2_.


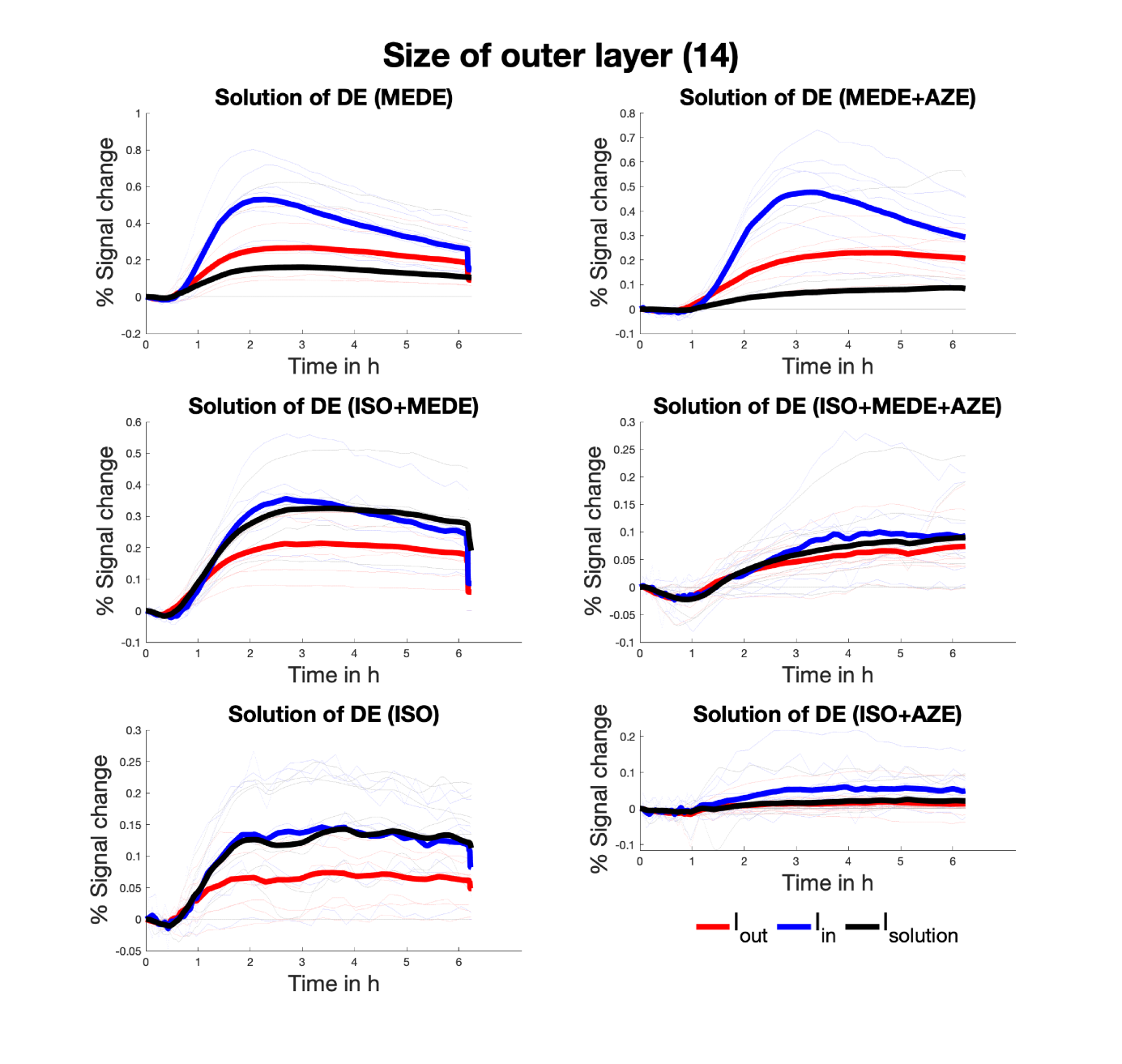


Illustration of the time signal curves of the inner (blue) and outer (red) layer and the solution of the differential equation (black) for each anesthetic condition with a thickness of the outer layer of fourteen voxel for every animal (thin curves) and mean (thick curves). The signal change is displayed as percent and the time is illustrated in hours.

The solution of the differential equation ($I_{in}^{\mathrm{sol}}\left( t \right)$) is the calculated TSC for the inner layer from the TSC of outer layer (I_out_) and the exchange parameters k_1_ and k_2_.


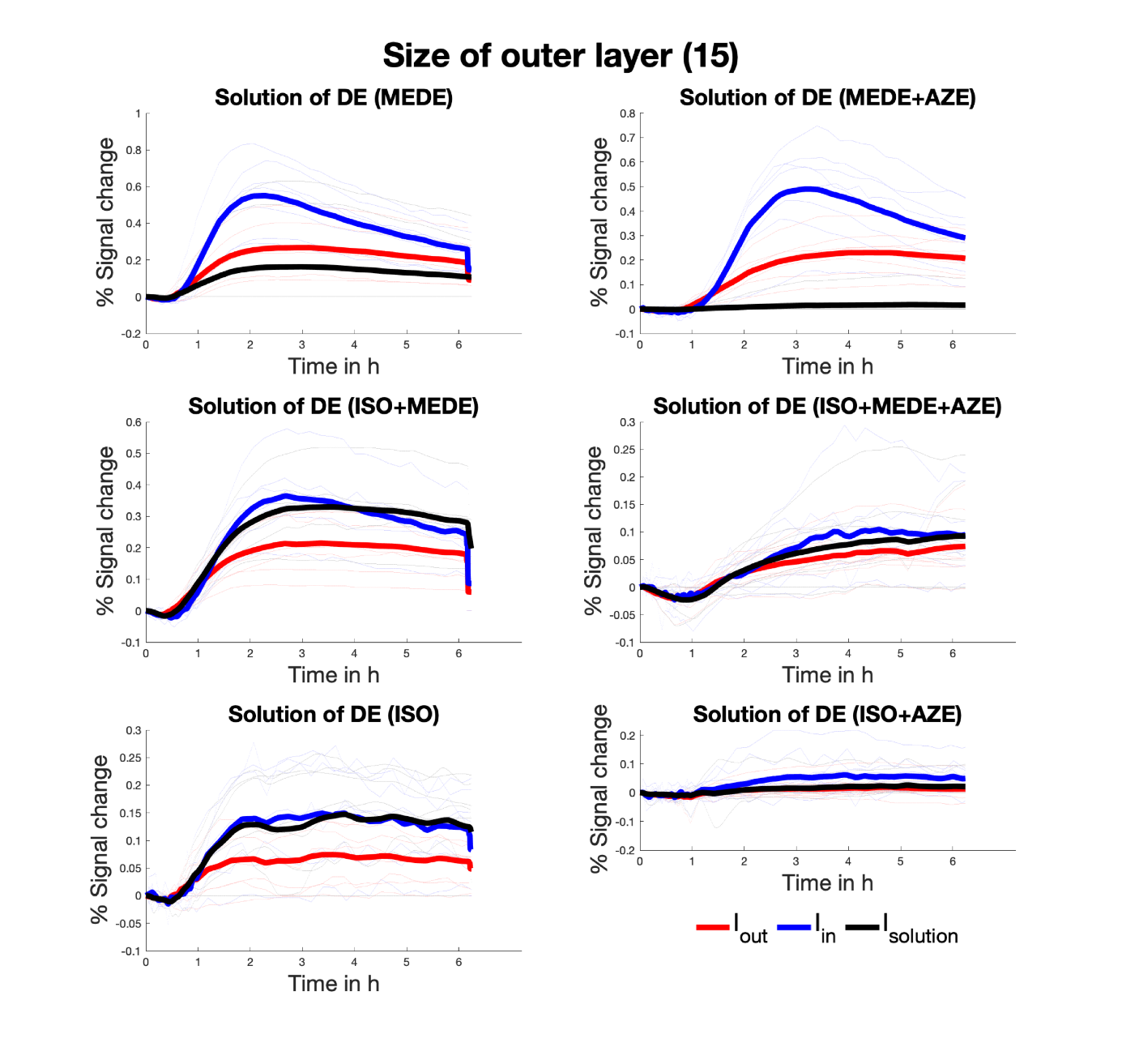


Illustration of the time signal curves of the inner (blue) and outer (red) layer and the solution of the differential equation (black) for each anesthetic condition with a thickness of the outer layer of fifteen voxel for every animal (thin curves) and mean (thick curves). The signal change is displayed as percent and the time is illustrated in hours.

The solution of the differential equation ($I_{in}^{\mathrm{sol}}\left( t \right)$) is the calculated TSC for the inner layer from the TSC of outer layer (I_out_) and the exchange parameters k_1_ and k_2_.


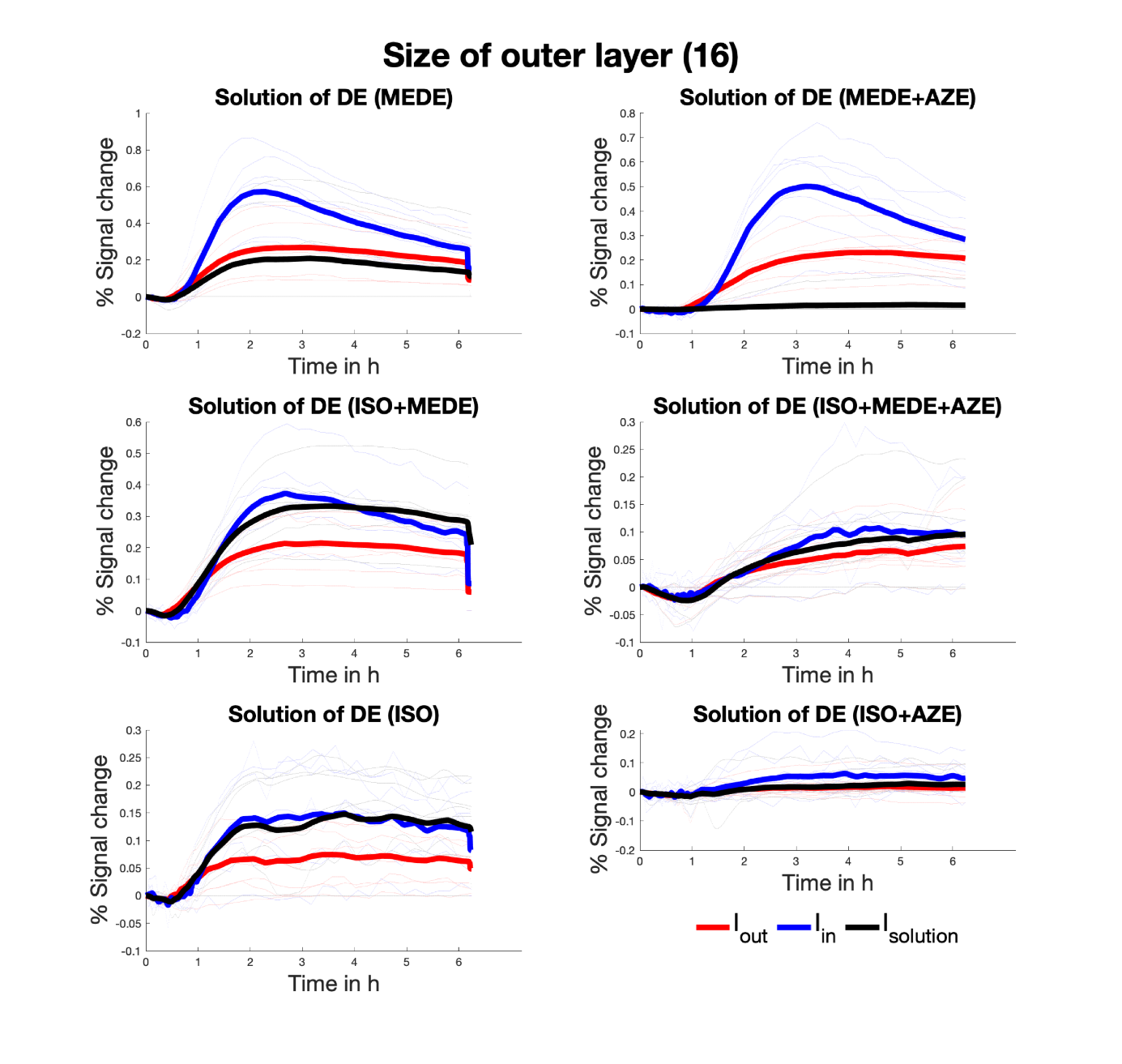


Illustration of the time signal curves of the inner (blue) and outer (red) layer and the solution of the differential equation (black) for each anesthetic condition with a thickness of the outer layer of sixteen voxel for every animal (thin curves) and mean (thick curves). The signal change is displayed as percent and the time is illustrated in hours.

The solution of the differential equation ($I_{in}^{\mathrm{sol}}\left( t \right)$) is the calculated TSC for the inner layer from the TSC of outer layer (I_out_) and the exchange parameters k_1_ and k_2_.


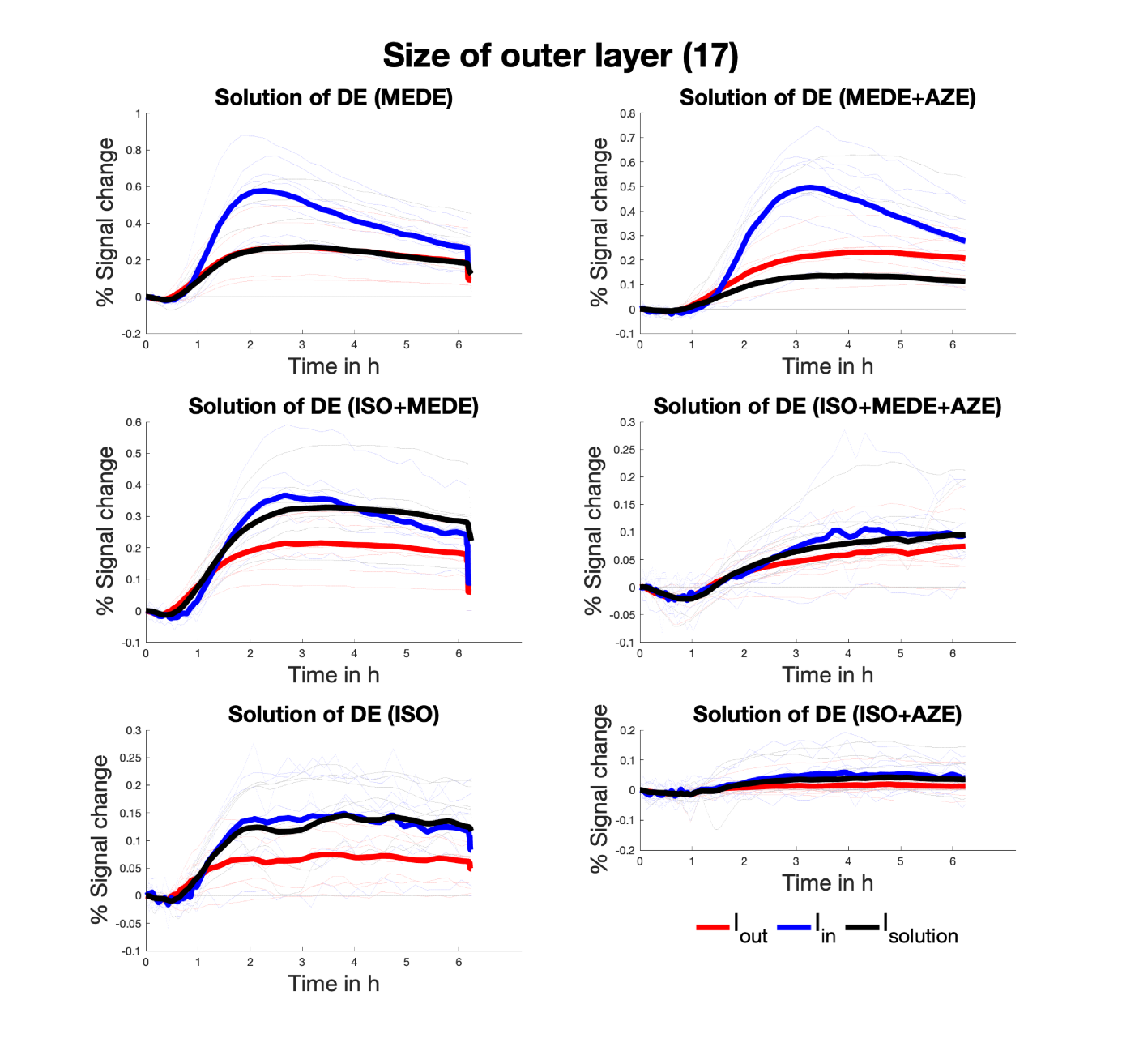


Illustration of the time signal curves of the inner (blue) and outer (red) layer and the solution of the differential equation (black) for each anesthetic condition with a thickness of the outer layer of seventeen voxel for every animal (thin curves) and mean (thick curves). The signal change is displayed as percent and the time is illustrated in hours.

The solution of the differential equation ($I_{in}^{\mathrm{sol}}\left( t \right)$) is the calculated TSC for the inner layer from the TSC of outer layer (I_out_) and the exchange parameters k_1_ and k_2_.
